# Advancing The Cancer Genome Atlas glioma MRI collections with expert segmentation labels and radiomic features

**DOI:** 10.1038/sdata.2017.117

**Published:** 2017-09-05

**Authors:** Spyridon Bakas, Hamed Akbari, Aristeidis Sotiras, Michel Bilello, Martin Rozycki, Justin S. Kirby, John B. Freymann, Keyvan Farahani, Christos Davatzikos

**Affiliations:** 1 Center for Biomedical Image Computing and Analytics (CBICA), Perelman School of Medicine, University of Pennsylvania, Richards Medical Research Laboratories, Floor 7, 3700 Hamilton Walk, Philadelphia, Pennsylvania 19104, USA; 2 Department of Radiology, Perelman School of Medicine, University of Pennsylvania, Richards Medical Research Laboratories, Floor 7, 3700 Hamilton Walk, Philadelphia, Pennsylvania 19104, USA; 3 Leidos Biomedical Research, Inc., Frederick National Laboratory for Cancer Research (FNLCR), Cancer Imaging Program (CIP), 8560 Progress Drive, Frederick, Maryland 21701, USA; 4 Cancer Imaging Program (CIP), National Cancer Institute (NCI), 9609 Medical Center Drive, Bethesda, Maryland 20892, USA

**Keywords:** CNS cancer, Cancer imaging, Image processing, Translational research, Computational models

## Abstract

Gliomas belong to a group of central nervous system tumors, and consist of various sub-regions. Gold standard labeling of these sub-regions in radiographic imaging is essential for both clinical and computational studies, including radiomic and radiogenomic analyses. Towards this end, we release segmentation labels and radiomic features for all pre-operative multimodal magnetic resonance imaging (MRI) (*n*=243) of the multi-institutional glioma collections of The Cancer Genome Atlas (TCGA), publicly available in The Cancer Imaging Archive (TCIA). Pre-operative scans were identified in both glioblastoma (TCGA-GBM, *n*=135) and low-grade-glioma (TCGA-LGG, *n*=108) collections via radiological assessment. The glioma sub-region labels were produced by an automated state-of-the-art method and manually revised by an expert board-certified neuroradiologist. An extensive panel of radiomic features was extracted based on the manually-revised labels. This set of labels and features should enable i) direct utilization of the TCGA/TCIA glioma collections towards repeatable, reproducible and comparative quantitative studies leading to new predictive, prognostic, and diagnostic assessments, as well as ii) performance evaluation of computer-aided segmentation methods, and comparison to our state-of-the-art method.

## Background & Summary

Gliomas are the most common primary central nervous system malignancies. These tumors, which exhibit highly variable clinical prognosis, usually contain various heterogeneous sub-regions (i.e., edema, enhancing and non-enhancing core), with variable histologic and genomic phenotypes. This intrinsic heterogeneity of gliomas is also portrayed in their radiographic phenotypes, as their sub-regions are depicted by different intensity profiles disseminated across multimodal MRI (mMRI) scans, reflecting differences in tumor biology. There is increasing evidence that quantitative analysis of imaging features^1–3^ extracted from mMRI (i.e., radiomic features), beyond traditionally used clinical measurements (e.g., the largest anterior-posterior, transverse, and inferior-superior tumor dimensions, measured on a subjectively-/arbitrarily-chosen slice), through advanced computational algorithms, leads to advanced image-based tumor phenotyping^4^. Such phenotyping may enable assessment of reflected biological processes and assist in surgical and treatment planning. Furthermore, its correlation with molecular characteristics established radiogenomic research^5–12^, leading to improved predictive, prognostic and diagnostic imaging biomarkers^9,12–32^, hence yielding the potential benefit towards non-invasive precision medicine^33^. However, it is clear from current literature^26,34–38^ that such advanced image-based phenotyping requires accurate annotations of the various tumor sub-regions.

Both clinical and computational studies focusing on such research require the availability of ample data to yield significant associations. Considering the value of big data and the potential of publicly available datasets for increased reproducibility of scientific findings, the National Cancer Institute (NCI) of the National Institutes of Health (NIH) created TCGA (cancergenome.nih.gov) and TCIA^39^ (www.cancerimagingarchive.net). TCGA is a multi-institutional comprehensive collection of various molecularly characterized tumor types, and its data are available in NCI’s Genomic Data Commons portal (gdc-portal.nci.nih.gov). Building upon NIH’s investment in TCGA, the NCI’s Cancer Imaging Program approached sites that contributed tissue samples, to obtain corresponding de-identified routine clinically-acquired radiological data and store them in TCIA. These repositories make available multi-institutional, high-dimensional, multi-parametric data of cancer patients, allowing for radiogenomic analysis. However, the data available in TCIA lack accompanying annotations allowing to fully exploit their potential in clinical and computational studies.

Towards addressing this limitation, this study provides segmentation labels and a panel of radiomic features for the glioma datasets included in the TCGA/TCIA repositories. The main goal is to enable imaging and non-imaging researchers to conduct their analyses and extract measurements in a reproducible and repeatable manner, while eventually allowing for comparison across studies. Specifically, the resources of this study provide i) imaging experts with benchmarks to debate their algorithms, and ii) non-imaging experts (e.g., bioinformaticians, clinicians), who do not have the background to interpret and/or appropriately process the raw images, with data helpful to conduct correlative genomic/clinical studies. Following radiological assessment of both the Glioblastoma Multiforme (TCGA-GBM^39^, *n*=262 [Data Citation 1]) and the Low-Grade-Glioma (TCGA-LGG^39^, *n*=199 [Data Citation 2]) collections, we identified 135 and 108 pre-operative mMRI scans, respectively. These scans include at least pre- and post-contrast T1-weighted, T2-weighted, and T2 Fluid-Attenuated Inversion Recovery (FLAIR) volumes. The segmentation labels provided for these scans are divided into two categories: a) computer-aided segmentation labels that could be mainly used for computational comparative studies, and b) manually corrected segmentation labels (approved by an expert board-certified neuroradiologist—M.B.) for use in clinically-oriented analyses, as well as for performance evaluation and training of computational models. The method employed to produce the computer-aided labels is named GLISTRboost^36,38^, which was awarded the 1st prize during the International Multimodal Brain Tumor Image Segmentation challenge 2015 (BraTS’15)^36,38,40–56^.

The generated data describe two independent datasets [Data Citation 3 and Data Citation 4], one for each glioma collection, and include the computer-aided and manually-revised segmentation labels, coupled with the corresponding co-registered and skull-stripped TCIA scans, in the Neuroimaging Informatics Technology Initiative (NIfTI^57^) format, allowing for direct analysis. Furthermore, a panel of radiomic features is included entailing intensity, volumetric, morphologic, histogram-based, and textural parameters, as well as spatial information and parameters extracted from glioma growth models^58–60^. In consistency with the FAIR (Findable, Accessible, Interoperable, Re-usable) principle, these data are made available through TCIA and should enable both clinical and computational quantitative analyses, as well as serve as a resource for i) educational training of neuroradiology and neurosurgery residents, and ii) performance evaluation of segmentation methods. Furthermore, it could potentially lead to predictive, prognostic, and diagnostic imaging markers suitable for enabling oncological treatment models customized on an individual patient basis (precision medicine), through non-invasive quantification of disease processes.

## Methods

### Data collection

**Figure 1:**
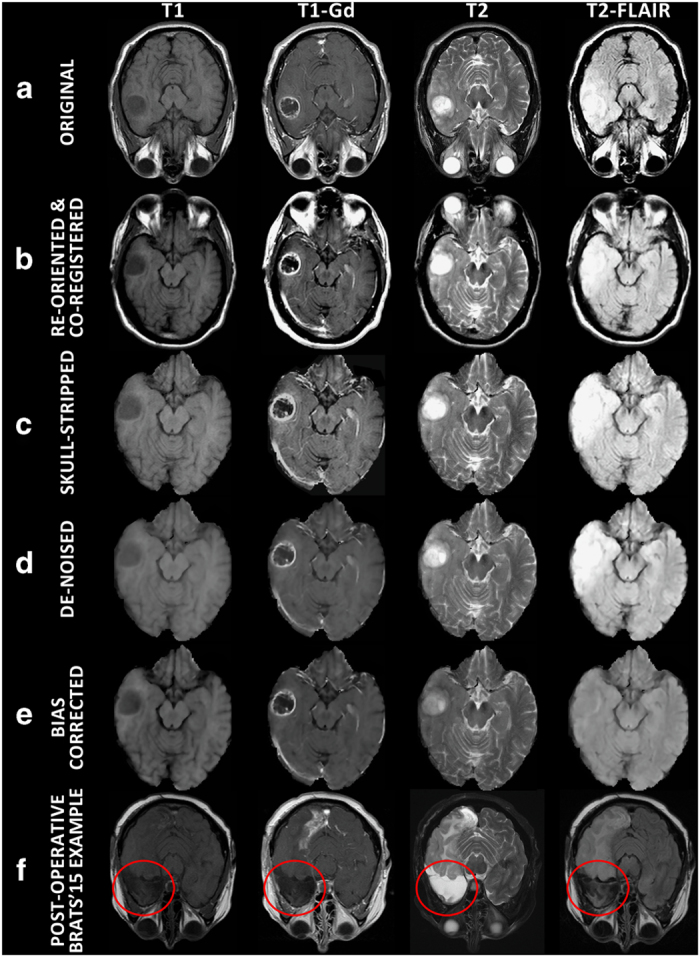
Single slice multimodal (T1, T1-Gd, T2, T2-FLAIR) MRI scans of example subjects. Examples are shown (**a**) in the original TCIA volume; (**b**–**e**) after application of various pre-processing steps; (**f**) for post-operative volumes in the BraTS ’15 data. Note that the step shown in (**e**), which is usually used to correct for intensity non-uniformities caused by the inhomogeneity of the scanner’s magnetic field during image acquisition, was not applied in the current data as it obliterated the T2-FLAIR signal.

The complete radiological data of the TCGA-GBM and TCGA-LGG collections consist of 262 [Data Citation 1] and 199 [Data Citation 2] mMRI scans provided from 8 and 5 institutions, respectively (Table 1). The data included in this study describe the subset of the pre-operative baseline scans of these collections, with available MRI modalities of at least T1-weighted pre-contrast (T1), T1-weighted post-contrast (T1-Gd), T2, and T2-FLAIR (Fig. 1a). Specifically, we considered 135 and 108 pre-operative baseline scans from the TCIA-GBM and TCIA-LGG collections, respectively. Further detailed information on the diversity of the imaging sequences used for this study is included in Table 2 (available online only). This table covers the TCIA institutional identifier, patient information (i.e., age, sex, weight), scanner information (i.e., manufacturer, model, magnetic field strength, station name), as well as specific imaging volume information extracted from the dicom headers (i.e., modality name, series number, accession number, acquisition/study/series date, scan sequence, type, slice thickness, slice spacing, repetition time, echo time, inversion time, imaging frequency, flip angle, specific absorption rate, numbers of slices, pixel dimensions, acquisition matrix rows/columns).

It should be noted that the diversity of the available scans in NCI/NIH/TCIA is driven by the fact that TCIA collected all available scans for subjects whose tissue specimens had passed the quality evaluation of the NCI/NIH/TCGA program. Due to this collection being retrospective all the MRI scans are considered ‘standard-of-care’, without following any uniform imaging protocol.

### Pre-processing

All pre-operative mMRI volumes were re-oriented to the LPS (left-posterior-superior) coordinate system (which is a requirement for GLISTRboost), co-registered to the same T1 anatomic template^61^ using affine registration through the Oxford center for Functional MRI of the Brain (FMRIB) Linear Image Registration Tool (FLIRT)^62,63^ of FMRIB Software Library (FSL)^64–66^, and resampled to 1 mm^3^ voxel resolution (Fig. 1b). The volumes of all the modalities for each patient were then skull-stripped using the Brain Extraction Tool (BET)^67,68^ from the FSL^64–66^ (Fig. 1c). Subsequent skull-stripping, on cases that BET produced insufficient results, was performed using a novel automated method based on a multi atlas registration and label fusion framework^69^. The template library for this task consisted of 216 MRI scans and their brain masks. This library was then used for target specific template selection and subsequent registrations using an existing strategy of MUlti-atlas Segmentation utilizing Ensembles (MUSE)^70^. A final region-growing based processing step, guided by T2, was applied to obtain a brain mask that includes the intra-cranial CSF. The resulted volumes are the ones provided in [Data Citation 3 and Data Citation 4].

For producing the computer-aided segmentation labels, further preprocessing steps included the smoothing of all volumes using a low-level image processing method, namely Smallest Univalue Segment Assimilating Nucleus (SUSAN)^71^, in order to reduce high frequency intensity variations (i.e., noise) in regions of uniform intensity profile while preserving the underlying structure (Fig. 1d). The intensity histograms of all modalities of all patients were then matched^72^ to the corresponding modality of a single reference patient, using the implemented version in ITK (HistogramMatchingImageFilter).

It should be noted that we did not use any non-parametric, non-uniform intensity normalization algorithm^73–75^ to correct for intensity non-uniformities caused by the inhomogeneity of the scanner’s magnetic field during image acquisition, as we observed that application of such algorithm obliterated the T2-FLAIR signal (Fig. 1e).

### Segmentation labels of glioma sub-regions

**Figure 2:**
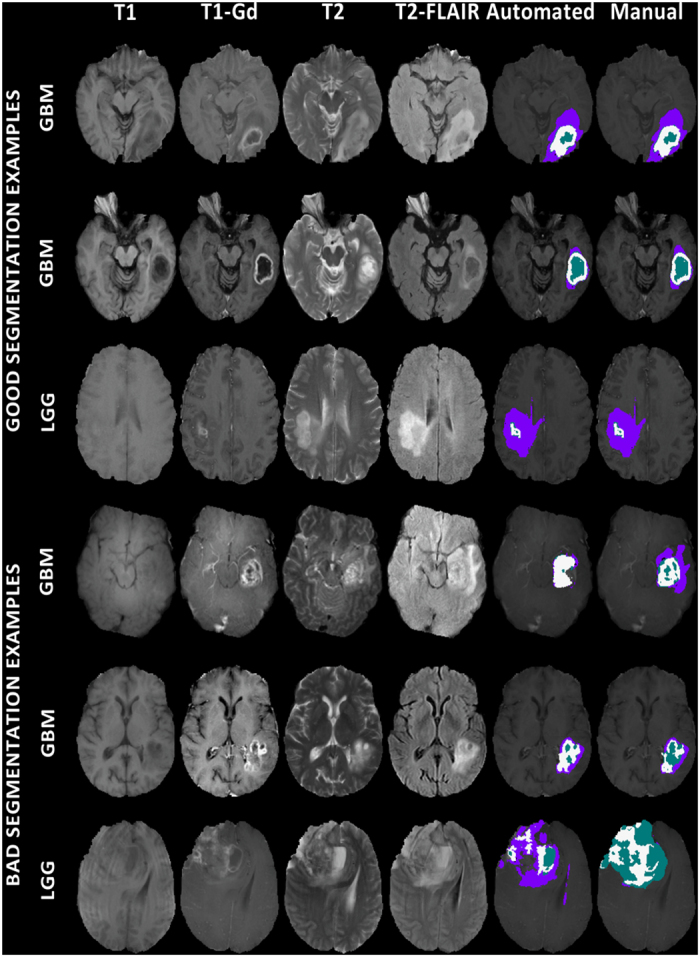
Single slice multimodal MRI scans of example subjects, illustrating all modalities used in GLISTRboost^36,38^ and example segmentation labels. The first three rows depict good segmentation examples, whereas the following three depict bad segmentation examples, produced by GLISTRboost^36,38^.

Consistent with the BraTS challenge^56^ the segmentation labels that we consider in the present study, and make available through TCIA [Data Citation 3 and Data Citation 4], delineate the enhancing part of the tumor core (ET), the non-enhancing part of the tumor core (NET), and the peritumoral edema (ED) (Fig. 2). The ET is described by areas that show hyper-intensity in T1-Gd when compared to T1, but also when compared to normal/healthy white matter (WM) in T1-Gd. Biologically, ET is felt to represent regions where there is leakage of contrast through a disrupted blood-brain barrier that is commonly seen in high grade gliomas. The NET represents non-enhancing tumor regions, as well as transitional/pre-necrotic and necrotic regions that belong to the non-enhancing part of the tumor core (TC), and are typically resected in addition to the ET. The appearance of the NET is typically hypo-intense in T1-Gd when compared to T1, but also when compared to normal/healthy WM in T1-Gd. Finally, the ED is described by hyper-intense signal on the T2-FLAIR volumes.

### Computer-aided segmentation approach

The method used in this study to produce the computer-aided segmentation labels for all pre-operative scans of both TCGA-GBM and TCGA-LGG collections is named GLISTRboost^36,38^ and it is based on a hybrid generative-discriminative model. The generative part incorporates a glioma growth model^58–60^, and is based on an Expectation-Maximization (EM) framework to segment the brain scans into tumor (i.e., ET, NET and ED), as well as healthy tissue labels (i.e., WM, gray matter, cerebrospinal fluid, vessels and cerebellum). The discriminative part is based on a gradient boosting^76,77^ multi-class classification scheme, which was trained on BraTS’15 data (www.virtualskeleton.ch/BRATS/Start2015), to refine tumor labels based on information from multiple patients. Lastly, a Bayesian strategy^78^ is employed to further refine and finalize the tumor segmentation based on patient-specific intensity statistics from the multiple modalities available. Example segmentation labels are illustrated in Fig. 2.

GLISTRboost^36,38^ is based on a modified version of the GLioma Image SegmenTation and Registration (GLISTR)^79^ software. GLISTR jointly performs a) the registration of a healthy population probabilistic atlas to brain scans of patients with gliomas using a tumor growth model to account for mass effects, and b) the segmentation of such scans into healthy and tumor tissues. The whole framework of GLISTR is based on a probabilistic generative model that relies on EM, to recursively refine the estimates of the posteriors for all tissue labels, the deformable mapping to the atlas, and the parameters of the incorporated brain tumor growth model^58–60^. GLISTR was originally designed to tackle cases with solitary GBMs^79–81^, and subsequently extended to handle multifocal masses and tumors of complex shapes with heterogeneous texture^82^. Furthermore, the original version of GLISTR^79–82^ was based on a single seed-point for each brain tissue label to represent its mean intensity value, while the variance was described by a fixed value for all labels. On the contrary, GLISTRboost incorporates multiple tissue seed-points for each label, to model more accurately the intensity distribution, i.e., mean and variance, for each tissue class. Note that both GLISTR and GLISTRboost take into account only the intensity value of the initialization tissue seed-points on each modality, while they discard spatial information regarding the coordinate position of the respective points. As a consequence, even if the initialized tissue seed-points during two independent segmentation attempts have different coordinates, the output sets of segmentation labels should be identical, given that the modeled intensity distributions during these attempts are the same. In addition to the tissue seed-points, GLISTR and on that account GLISTRboost, requires the definition of a single seed-point and a radius for approximating the center and the bulk volume of each apparent tumor by a sphere. All these seed-points are initialized using the ‘Cancer Imaging Phenomics Toolkit’ (CaPTk)^83^ (www.med.upenn.edu/sbia/captk.html), which has been primarily developed for this purpose, by the Center for Biomedical Image Computing and Analytics (CBICA) of the University of Pennsylvania. Given the tumor seed-point and radius for a tumor, a growth model is initiated by the parametric model of a sphere. This growth model is used to deform a healthy atlas into one with tumor and edema tissues matching the input scans, while approximating the deformations occurred to all brain tissues due to the mass effect of the tumors. A tumor shape prior is estimated by a random-walk-based generative model, which uses the tumor seed-points as initialization cues. This shape prior is systemically incorporated into the EM framework via an empirical Bayes model^82^. Furthermore, a minimum of three initialization seed-points is needed for each brain tissue label, in order to capture the intensity variation and model the intensity distribution across all modalities. Use of multiple seed-points improves the initialization of the EM framework, leading to more accurate segmentation labels, when compared to the single seed-point approach^82^. The output of GLISTR is a posterior probability map for each tissue label, as well as an integrative label map, which describes a very good ‘initial’ segmentation of all different tissues within a patient's brain.

This ‘initial’ segmentation is then refined by taking into account information from multiple patients via a discriminative machine-learning algorithm. Specifically, we used the gradient boosting algorithm^76^ to perform voxel-level multi-label classification. Gradient boosting produces a prediction model by combining weak learners in an ensemble. We used decision trees of maximum depth 3 as ‘weak learners’, which were trained in a sub-sample of the training set, in order to introduce randomness^77^. The sampling rate was set equal to 0.6, while additional randomness was introduced by sampling stochastically a subset of imaging (i.e., radiomic) features at each node. The number of sampled features was set equal to the square root of the total number of features. The algorithm was terminated after 100 iterations.

The set of features used for training our model was extracted volumetrically and consists of i) intensity information, ii) image derivative, iii) geodesic information, iv) texture features, and v) the GLISTR posterior probability maps. The intensity information is summarized by the raw intensity value, I, of each image voxel, v_i_, at each modality, m, (i.e., *I(v*_*i*_^*m*^)), as well as by the respective differences among all four modalities, i.e., I(v_i_^T1^)- I(v_i_^T1Gd^), I(v_i_^T1^)- I(v_i_^T2^), I(v_i_^T1^)- I(v_i_^T2FLAIR^), I(v_i_^T1Gd^)- I(v_i_^T1^), I(v_i_^T1Gd^)- I(v_i_^T2^), I(v_i_^T1Gd^)- I(v_i_^T2FLAIR^), I(v_i_^T2^)- I(v_i_^T1^), I(v_i_^T2^)- I(v_i_^T1Gd^), I(v_i_^T2^)- I(v_i_^T2FLAIR^), I(v_i_^T2FLAIR^)- I(v_i_^T1^), I(v_i_^T2FLAIR^)- I(v_i_^T1Gd^), I(v_i_^T2FLAIR^)- I(v_i_^T2^). The image derivative component consists of the Laplacian of Gaussians and the image gradient magnitude. Note that in order to ensure that the intensity-based features are comparable, intensity normalization was performed across subjects based on the median intensity value of the cerebrospinal fluid label, as provided by GLISTR. Geodesic information was used to introduce spatial context information. At any voxel *v*_*i*_ we calculated the geodesic distance from the seed-point at voxel *v*_*s*_, which was used in GLISTR as the tumor center. The geodesic distance between *v*_*i*_ and *v*_*s*_ was estimated using the fast marching method^84,85^ and by taking into account local image gradient magnitude^86^. Furthermore, we used texture features computed from a gray-level co-occurrence matrix (GLCM)^87^. Specifically, these texture features describe first-order statistics (i.e., mean and variance of each modality’s intensities within a radius of 2 voxels for each voxel), as well as second-order statistics. To obtain the latter, the image volumes were firstly normalized to 64 different gray levels, and then a bounding box of 5-by-5-by-5 voxels was used for all the voxels of each image as a sliding window. Then, a GLCM was populated by taking into account the intensity values within a radius of 2 pixels and for the 26 main 3D directions to extract the energy, entropy, dissimilarity, homogeneity (i.e., inverse difference moment of order 2), and inverse difference moment of order 1. These features were computed for each direction and their average was used. To avoid overfitting, the gradient boosting machine was trained using simultaneously both LGG and GBM training data of BraTS’15, in a 54-fold cross-validation setting (allowing for using a one out of the 54 available LGGs of the BraTS’15 training data, within each fold).

Finally, the segmentation results were further refined for each patient separately, by assessing the local intensity distribution of the segmentation labels and updating their spatial configuration based on a probabilistic model^78^. The intensity distributions of the WM, ED, NET and ET, were populated separately using the corresponding voxels of posterior probability equal to 1, as given by GLISTR. Histogram normalization was then performed for the 3 pair-wise distributions considered; ED versus WM in T2-FLAIR, ET versus ED in T1-Gd, and ET versus NET in T1-Gd. Maximum likelihood estimation was used to model the class-conditional probability densities (*Pr(I(v*_*i*_*)|Class*) by a distinct Gaussian model for each class. In all pair-wise comparisons described before, the former tissue is expected to be brighter than the latter. Voxels of each class with spatial proximity smaller than 4 voxels to the voxels of the paired class, were evaluated by assessing their intensity *I(v*_*i*_) and comparing the (‘*Pr(I(v*_*i*_*)|Class*_*1*_) with *Pr(I(v*_*i*_*)|Class*_*2*_). The voxel *v*_*i*_ was then classified into the tissue class with the larger conditional probability. This is equivalent to a classification based on Bayes' Theorem with equal priors for the two classes, i.e., *Pr(Class*_*1*_*)=Pr(Class*_*2*_*)=0.5*.

### Manual revision

**Figure 3:**
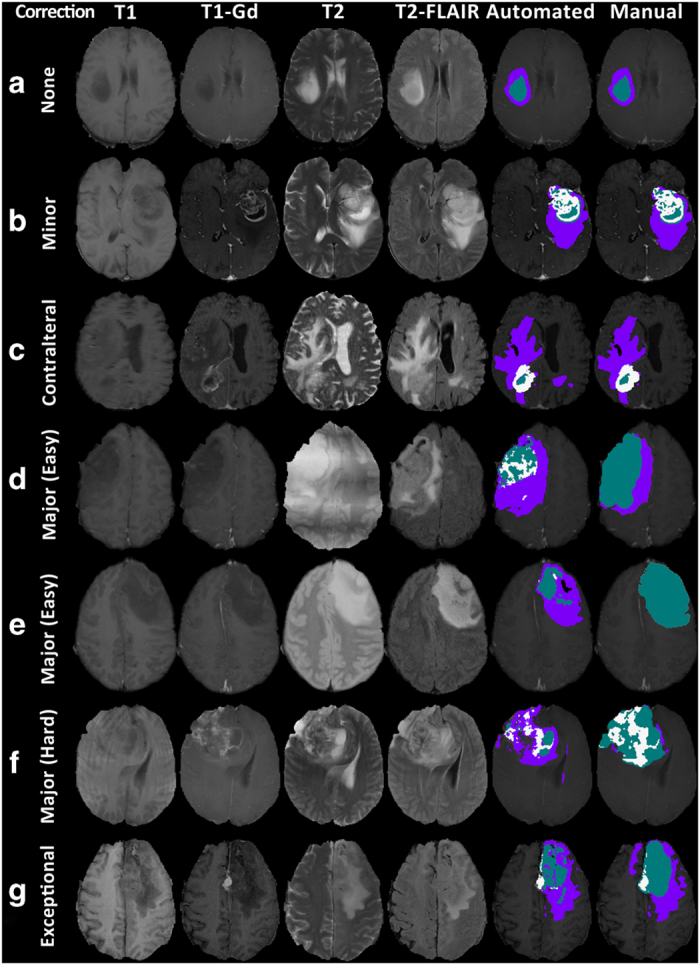
Single slice multimodal MRI scans of example subjects, illustrating all modalities used in GLISTRboost^36,38^ and examples of the computer-aided (automated) and the manually-revised (manual) segmentation labels. The type of corrections applied during the manual-revision of the segmentation labels is also shown in the left side of each row; (**a**) no correction, (**b**) minor corrections, (**c**) corrections in the contralateral edema, (**d**) major (easy) correction of LGG without ET, (**e**) major (easy) correction of LGG without ET or ED, (**f**) major (hard) corrections, (**g**) exceptional subject (TCGA-DU-7304) that could have a meningioma in the midline as the apparent lesion seems to raise from the dura.

The output of GLISTRboost segmentation is expected to yield labels for ET, NET, and ED. However, some gliomas, especially LGG, do not exhibit much contrast enhancement, or ED. Biologically, LGGs may have less blood-brain barrier disruption (leading to less leak of contrast during the scan), and may grow at a rate slow enough to avoid significant edema formation, which results from rapid disruption, irritation, and infiltration of normal brain parenchyma by tumor cells. As such, manual revision of the segmentation labels was performed, particularly for LGG cases lacking ET or ED regions. Specifically, after taking all the above into consideration, in scans of LGGs without an apparent ET area we consider only the NET and ED labels (Fig. 3a,d), whereas in LGG scans without ET and without obvious texture differences across modalities we consider only the NET label, allowing for distinguishing between normal and abnormal brain tissue (Fig. 3e). The difficulty in calculating the accurate boundaries between tumor and healthy tissue in the operating room is reflected in the segmentation labels as well; there is high uncertainty among neurosurgeons, neuroradiologists, and imaging scientists in delineating these boundaries. Therefore, small regions within the segmented labels that were ambiguous of their exact classification, were left as segmented by GLISTRboost.

Manual revisions/corrections applied in the computer-aided segmentation labels include: i) obvious under- or over-segmented ED/ET/NET regions (Fig. 3d–g), ii) voxels classified as ED within the tumor core (Fig. 3b,c,g), iii) unclassified voxels within the tumor core (Fig. 3c–g), iv) voxels classified as NET outside the tumor core. Contralateral and periventricular regions of T2-FLAIR hyper-intensity were excluded from the ED region (Fig. 3c,f), unless they were contiguous with peritumoral ED (Fig. 3g—addition of apparent contralateral ED), as these areas are generally considered to represent chronic microvascular changes, or age-associated demyelination, rather than tumor infiltration^88^.

### Radiomic features panel

An extensive panel of more than 700 radiomic features is extracted volumetrically (in 3D), based on the manually-revised labels of each tumor sub-region that comprised i) intensity, ii) volumetric^89^, iii) morphologic^90–93^, iv) histogram-based^31^, and v) textural parameters, including features based on wavelets^94^, GLCM^87^, Gray-Level Run-Length Matrix (GLRLM)^93,95–98^, Gray-Level Size Zone Matrix (GLSZM)^95–97,99^, and Neighborhood Gray-Tone Difference Matrix (NGTDM)^100^, as well as vi) spatial information^101^, and vii) glioma diffusion properties extracted from glioma growth models^58–60^, that are already evaluated as having predictive and prognostic value^30–32,102,103^. The specific features provided are all shown in Table 3 (available online only).

These radiomic features are provided on an ‘as-is’ basis, and are distinct from the panel of features used in GLISTRboost. The biological significance of these individual radiomic features remains unknown, but we include them here to facilitate research on their association with molecular markers, clinical outcomes, treatment responses, and other endpoints, by researchers without sufficient computational background to extract such features. Although researchers can derive their own radiomic features from our segmentation labels, and the corresponding images we included a collection of features that have been shown in various studies to relate to clinical outcome^31^ and underlying tumor molecular characteristics^30,32^. Note that the radiomic features we provide are extracted from the denoised images, and the users might also want to consider extracting features from the unsmoothed images provided in [Data Citation 3 and Data Citation 4].

### Code availability

All software tools used for pre-processing, initialization, and generation of the hereby described segmentation labels are based on publicly available tools. Specifically, the tools used for the pre-processing steps of skull-stripping (BET)^67,68^ and co-registration (FLIRT)^62,63^ are publicly available from the FMRIB Software Library (FSL)^64–66^, in: fsl.fmrib.ox.ac.uk. The software used for the further skull-stripping approaches, i.e., Multi-Atlas Skull-Stripping (MASS)^69^ and MUSE^70^, are publicly available in www.med.upenn.edu/sbia/mass.html and www.med.upenn.edu/sbia/muse.html, respectively.

We developed CaPTk^83^ as a toolkit to facilitate translation of complex research algorithms into clinical practice, by enabling operators to conduct quantitative analyses without requiring substantial computational background. Towards this end CaPTk is a dynamically growing software platform, with various integrated applications, allowing 1) interactive definition of coordinates and regions, 2) generic image analysis (e.g., registration, feature extraction), and 3) specialized analysis algorithms (e.g., identification of genetic mutation imaging markers^12^). Specifically for this study, CaPTk was used to 1) manually initialize seed-points required for the initialization of GLISTRboost^36,38^, 2) apply the de-noising approach (SUSAN)^71^ used for smoothing images before their input to GLISTRboost, as well as 3) to extract the radiomic features released in TCIA [Data Citation 3 and Data Citation 4]. The exact version used for initializing the required seed-points in this study was released on the 14th of October 2016 and the code source, as well as executable installers, are available in: www.med.upenn.edu/sbia/captk.html.

Finally, our segmentation approach, GLISTRboost^36,38^, has been made available for public use through the Online Image Processing Portal (IPP—ipp.cbica.upenn.edu) of the CBICA. CBICA's IPP allows users to perform their data analysis using integrated algorithms, without any software installation, whilst also using CBICA's High Performance Computing resources. It should be noted that we used the Python package scikit-learn^104^ for the implementation of the gradient boosting algorithm.

## Data Records

We selected only the pre-operative multimodal scans of the TCGA-GBM [Data Citation 1] and TCGA-LGG [Data Citation 2] glioma collections, from the publicly available TCIA repository. The generated data, which is made publicly available through TCIA’s Analysis Results Directory (wiki.cancerimagingarchive.net/x/sgH1) [Data Citation 3 and Data Citation 4], comprise pre-operative baseline re-oriented, co-registered and skull-stripped mMRI scans together with their corresponding computer-aided and manually-revised segmentation labels in NIfTI^57^ format. We have further enriched the file containers to include an extensive panel of radiomic features, which we hope may facilitate radiogenomic research using the TCGA portal, as well as comparison of segmentation methods, even among those scientists without image analysis resources.

A subset of the pre-operative scans included in the generated data [Data Citation 3 and Data Citation 4] was also part of the BraTS’15 dataset (Table 4 (available online only)), which were skull-stripped, co-registered to the same anatomical template and resampled to 1 mm^3^ voxel resolution by the challenge organizers. For this subset, we provide the identical MRI volumes as provided by the BraTS’15 challenge, allowing other researchers to compare their segmentation labels to the leaderboard of the BraTS’15 challenge. Furthermore, the manually-revised segmentation labels provided in [Data Citation 3 and Data Citation 4] are included in the datasets of the BraTS’17 challenge, for benchmarking computational segmentation algorithms against tumor delineation validated by expert neuroradiologists, allowing for repeatable research.

## Technical Validation

### Data collection

Our expert board-certified neuroradiologist (M.B.) identified 135 and 108 pre-operative baseline scans of the TCGA-GBM and the TCGA-LGG glioma collections, via radiological assessment and while blinded to the glioma grade. Since it is not always easy to determine if a scan is pre-operative or post-operative only by visually assessing MRI volumes, and the radiological reports were not available through the TCGA/TCIA repositories, whenever we mention ‘pre-operative scans’ in this study, we refer to those that radiographically do not have clear evidence of prior instrumentation. Specifically, the main evaluation criterion for classifying scans as pre-operative, was absence of obvious skull defect and of operative cavity through either biopsy or resection.

We note that a mixed (pre- and post-operative) subset of 223 and 59 scans from the TCIA-GBM and TCIA-LGG datasets, respectively, were included in the BraTS’15 challenge, as part of their training (n_GBM_=200, n_LGG_=44) and testing (n_GBM_=23, n_LGG_=15) datasets, via the Virtual Skeleton Database (VSD) platform^56,105^ (www.virtualskeleton.ch). Since an explicit distinction as pre- or post-operative was not provided for the BraTS’15 dataset, we conducted the radiological assessment of the complete TCIA collections, blind to whether a scan was part of the BraTS challenge, and only included the BraTS’15 volumes identified as pre-operative (Fig. 1f) (Table 4 (available online only)).

### Segmentation labels

The segmentation method we developed to produce the segmentation labels, GLISTRboost^36,38^, was ranked as the best performing method and awarded the 1st prize during the International Multimodal Brain Tumor Image Segmentation challenge 2015 (BraTS’15)^36,38,40–56^. Specifically, the performance of the computer-aided segmentation labels was assessed during the challenge for the test data, through the VSD platform, by comparing the voxel-level overlap between the segmentation labels produced by GLISTRboost and the ground truth labels provided by the BraTS organizers in three regions, i.e., the whole tumor (WT), the tumor core (TC) and the ET. The WT describes the union of the ET, NET and ED, whereas the TC describes the union of the ET and NET. The performance was quantitatively validated by the per-voxel overlap between respective regions, using the DICE coefficient and the robust Hausdorff distance (95% quantile), as suggested by the BraTS’15 organizers^56^. The former metric takes values between 0 and 1, with higher values corresponding to increased overlap, whereas lower values in the latter correspond to segmentation labels closer to the gold standard labels. Note that the quantitative results for the test data were not provided to the participants, until a manuscript summarizing the results of BraTS’14 and BraTS’15 is published. However, for reporting the performance of our method, we report here the cross-validated results of the same metrics used in BraTS’15 for the subset of GBM subjects included in the training set of BraTS’15 and identified as pre-operative in this study (Table 4 (available online only)). The median DICE values with their corresponding inter-quartile ranges (IQR) for the three evaluated regions, i.e., WT, TC, ET, were equal to 0.92 (IQR: 0.88–0.94), 0.88 (IQR: 0.81–0.93) and 0.88 (IQR: 0.81–0.91), respectively. Equivalently, the 95th percentile of the Hausdorff distance for WT, TC and ET were equal to 3.61 (IQR: 2.39–8.15), 4.06 (IQR: 2.39–7.29), and 2 (IQR: 1.41–2.83), respectively.

Furthermore, we used the Jaccard coefficient, in order to quantify the difference between the computer-aided segmentation labels produced for all the scans identified as pre-operative and all the manually-corrected labels that we provide in [Data Citation 3 and Data Citation 4]. The median (mean±std.dev) Jaccard values for the three regions of interest i.e., WT, TC, ET, were equal to 0.96 (0.93±0.1), 0.87 (0.78±0.23), and 0.86 (0.73±0.29), respectively.

### Manual correction

The classification scheme of segmentation labels considered for the manual corrections of the GBM and LGG cases describe all three segmentation labels (i.e., ET, NET, and ED) for both GBMs and LGGs with an apparent ET area. However, whenever we note LGG scans without an apparent ET area and not obvious texture differences, we considered only the NET label, allowing for distinguishing between normal and abnormal brain tissue, as slowly growing tumors are not expected to induce ED. Furthermore, due to high uncertainty (reported by neurosurgeons, neuroradiologists, and imaging scientists) on the exact boundaries between the various tumor labels, particularly between NET and ED, small regions that visual assessment was ambiguous of their exact classification, were left as segmented by GLISTRboost.

Manual revisions/corrections applied in the computer-aided segmentation labels comprise: i) obvious under- or over-segmented ED/ET/NET regions (Fig. 3d–g), ii) voxels classified as ED within the tumor core (Fig. 3b,c,g), iii) unclassified voxels within the tumor core (Fig. 3c–g), iv) voxels classified as NET outside the tumor core. Note that during the manual corrections only peritumoral ED was considered, and both contralateral, and periventricular ED was deleted (Fig. 3c,f), unless it was a clear continuation of the peritumoral ED, in which cases was added (Fig. 3g). The rationale for this is that contralateral and periventricular white matter hyper-intensities regions might be considered pre-existing conditions, related to small vessel ischemic disease, especially in older patients.

The scheme followed for the manual correction included two computational imaging scientists (S.B., A.S.) and a medical doctor (H.A.) working in medical image computing and analysis for 10, 12 and 8 years, respectively. These operators corrected mislabeled voxels following the rules set by our expert board-certified neuroradiologist (M.B.) with 14 years of experience. The corrected labels were then iteratively re-evaluated by the latter and re-iterated until they were satisfactory segmented.

## Additional Information

**How to cite this article:** Bakas, S. *et al.* Advancing The Cancer Genome Atlas glioma MRI collections with expert segmentation labels and radiomic features. *Sci. Data* 4:170117 doi: 10.1038/sdata.2017.117 (2017).

**Publisher’s note:** Springer Nature remains neutral with regard to jurisdictional claims in published maps and institutional affiliations.

## Supplementary Material



## Figures and Tables

**Table 1:** Source of radiographic data for patients (n) provided in TCIA.

**Collection**	***n***	**Institutions contributed data—(n)**	**TCGA ID**	**Scanner (strength in T)**
**TCGA-GBM**	262	Henry Ford Hospital, Detroit, MI—(74)	TCGA-06	GE (1.5, 3): Genesis Signa, Signa Excite
		CWRU School of Medicine, Cleveland, OH—(38)	TCGA-19	Siemens (1.5, 3): Avanto, Symphony, Verio
		University of California, San Francisco, CA—(32)	TCGA-08	GE (1.5, 3): Genesis Signa, Signa Excite
		Emory University, Atlanta, GA—(31)	TCGA-14	Philips (1.5): InteraSiemens (1.5, 3): Avanto, Trio
		MD Anderson Cancer Center, Houston, TX—(25)	TCGA-02	GE: Genesis Signa, Signa Excite
		Duke University School of Medicine, Durham, NC—(24)	TCGA-12	GE (1.5): Genesis Signa, Signa HDx,Signa ExciteSiemens (1.5, 3): Avanto, Trio, Symphony
		Thomas Jefferson University, Philadelphia, PA—(22)	TCGA-76	Philips (1.5, 3): AchievaSiemens (1.5): Magnetom Vision
		Fondazione IRCCS Instituto Neuroligico C. Besta, Milan, Italy—(16)	TCGA-27	Philips (0.5): Intera Siemens (1.5): Avanto
**TCGA-LGG**	199	St Joseph Hospital/Medical Center, Phoenix, AZ—(98)	TCGA-HT	GE (1.5, 3): Signa Excite, Signa HDx, Signa HDxt
		Henry Ford Hospital, Detroit, MI—(57)	TCGA-DU	Hitachi (1.16): OasisGE (1.5, 3): Genesis, Signa Excite, Signa HDxt,Philips (1.5, 3): Intera, Ingenia
		Case Western Reserve University, Cleveland, OH—(22)	TCGA-FG	Siemens (1.5, 3): Avanto, Symphony, Skyra, Verio
		Thomas Jefferson University, Philadelphia, PA—(20)	TCGA-CS	GE (1.5): Genesis Signa, Signa HDxtPhilips (1.5, 3): AchievaSiemens (1.5): Magnetom Vision
		University of North Carolina, Chapel Hill, NC—(2)	TCGA-EZ	Siemens (3): TrioTim

**Table 2:** Detailed information capturing the diversity of the used images

**Collection**	**TCGA ID**	**Included in BraTS'15**	**BraTS'15 suggested a baseline pre-op scan**	**We Identified Baseline Pre-operative scans**
TCGA-GBM	TCGA-02-0003	Yes (Testing data)	Yes	Yes
TCGA-GBM	TCGA-02-0006	Yes (Training data)	Yes	Yes
TCGA-GBM	TCGA-02-0009	Yes (Training data)	Yes	Yes
TCGA-GBM	TCGA-02-0011	Yes (Training data)	Yes	Yes
TCGA-GBM	TCGA-02-0027	Yes (Training data)	Yes	Yes
TCGA-GBM	TCGA-02-0033	Yes (Training data)	Yes	Yes
TCGA-GBM	TCGA-02-0034	Yes (Training data)	Yes	Yes
TCGA-GBM	TCGA-02-0037	Yes (Training data)	Yes	Yes
TCGA-GBM	TCGA-02-0046	Yes (Training data)	Yes	Yes
TCGA-GBM	TCGA-02-0047	Yes (Training data)	Yes	Yes
TCGA-GBM	TCGA-02-0048	No	N/A	Yes
TCGA-GBM	TCGA-02-0054	Yes (Training data)	Yes	Yes
TCGA-GBM	TCGA-02-0059	Yes (Training data)	Yes	Yes
TCGA-GBM	TCGA-02-0060	No	N/A	Yes
TCGA-GBM	TCGA-02-0064	Yes (Training data)	Yes	Yes
TCGA-GBM	TCGA-02-0068	Yes (Training data)	Yes	Yes
TCGA-GBM	TCGA-02-0069	Yes (Training data)	Yes	Yes
TCGA-GBM	TCGA-02-0070	Yes (Training data)	Yes	Yes
TCGA-GBM	TCGA-02-0075	Yes (Training data)	Yes	Yes
TCGA-GBM	TCGA-02-0085	Yes (Training data)	Yes	Yes
TCGA-GBM	TCGA-02-0086	Yes (Training data)	Yes	Yes
TCGA-GBM	TCGA-02-0087	Yes (Training data)	Yes	Yes
TCGA-GBM	TCGA-02-0102	Yes (Training data)	Yes	Yes
TCGA-GBM	TCGA-02-0106	Yes (Training data)	Yes	Yes
TCGA-GBM	TCGA-02-0116	Yes (Training data)	Yes	Yes
TCGA-GBM	TCGA-06-0119	Yes (Training data)	Yes	Yes
TCGA-GBM	TCGA-06-0121	No	N/A	No
TCGA-GBM	TCGA-06-0122	Yes (Training data)	Yes	Yes
TCGA-GBM	TCGA-06-0125	No	N/A	No
TCGA-GBM	TCGA-06-0127	Yes (Testing data)	Yes	No
TCGA-GBM	TCGA-06-0128	No	N/A	Yes
TCGA-GBM	TCGA-06-0129	No	N/A	No
TCGA-GBM	TCGA-06-0130	Yes (Training data)	Yes	Yes
TCGA-GBM	TCGA-06-0132	Yes (Training data)	No	Yes
TCGA-GBM	TCGA-06-0133	Yes (Testing data)	No	Yes
TCGA-GBM	TCGA-06-0137	Yes (Training data)	Yes	Yes
TCGA-GBM	TCGA-06-0138	Yes (Training data)	Yes	Yes
TCGA-GBM	TCGA-06-0139	Yes (Training data)	Yes	Yes
TCGA-GBM	TCGA-06-0141	Yes (Training data)	Yes	No
TCGA-GBM	TCGA-06-0142	Yes (Training data)	Yes	Yes
TCGA-GBM	TCGA-06-0143	Yes (Training data)	No	No
TCGA-GBM	TCGA-06-0145	Yes (Training data)	Yes	Yes
TCGA-GBM	TCGA-06-0147	Yes (Training data)	Yes	No
TCGA-GBM	TCGA-06-0148	No	N/A	No
TCGA-GBM	TCGA-06-0149	Yes (Training data)	Yes	Yes
TCGA-GBM	TCGA-06-0154	Yes (Training data)	Yes	Yes
TCGA-GBM	TCGA-06-0156	Yes (Testing data)	No	No
TCGA-GBM	TCGA-06-0157	Yes (Training data)	Yes	No
TCGA-GBM	TCGA-06-0158	Yes (Training data)	Yes	Yes
TCGA-GBM	TCGA-06-0160	No	N/A	No
TCGA-GBM	TCGA-06-0162	Yes (Training data)	Yes	Yes
TCGA-GBM	TCGA-06-0164	Yes (Training data)	Yes	Yes
TCGA-GBM	TCGA-06-0165	Yes (Training data)	No	No
TCGA-GBM	TCGA-06-0166	Yes (Testing data)	No	Yes
TCGA-GBM	TCGA-06-0168	Yes (Testing data)	Yes	Yes
TCGA-GBM	TCGA-06-0171	Yes (Training data)	No	No
TCGA-GBM	TCGA-06-0173	No	N/A	No
TCGA-GBM	TCGA-06-0174	No	N/A	No
TCGA-GBM	TCGA-06-0175	No	N/A	Yes
TCGA-GBM	TCGA-06-0176	Yes (Training data)	Yes	Yes
TCGA-GBM	TCGA-06-0177	Yes (Training data)	Yes	Yes
TCGA-GBM	TCGA-06-0178	Yes (Testing data)	Yes	No
TCGA-GBM	TCGA-06-0179	Yes (Training data)	Yes	Yes
TCGA-GBM	TCGA-06-0182	Yes (Training data)	Yes	Yes
TCGA-GBM	TCGA-06-0184	Yes (Training data)	Yes	Yes
TCGA-GBM	TCGA-06-0185	Yes (Training data)	Yes	Yes
TCGA-GBM	TCGA-06-0187	Yes (Training data)	Yes	Yes
TCGA-GBM	TCGA-06-0188	Yes (Training data)	Yes	Yes
TCGA-GBM	TCGA-06-0189	No	N/A	Yes
TCGA-GBM	TCGA-06-0190	Yes (Training data)	Yes	Yes
TCGA-GBM	TCGA-06-0192	No	N/A	Yes
TCGA-GBM	TCGA-06-0210	No	N/A	No
TCGA-GBM	TCGA-06-0213	Yes (Testing data)	Yes	Yes
TCGA-GBM	TCGA-06-0214	Yes (Training data)	Yes	No
TCGA-GBM	TCGA-06-0216	No	N/A	No
TCGA-GBM	TCGA-06-0221	No	N/A	No
TCGA-GBM	TCGA-06-0237	Yes (Training data)	Yes	No
TCGA-GBM	TCGA-06-0238	Yes (Training data)	Yes	Yes
TCGA-GBM	TCGA-06-0240	Yes (Training data)	Yes	Yes
TCGA-GBM	TCGA-06-0241	Yes (Testing data)	Yes	Yes
TCGA-GBM	TCGA-06-0644	Yes (Training data)	Yes	Yes
TCGA-GBM	TCGA-06-0645	Yes (Testing data)	Yes	No
TCGA-GBM	TCGA-06-0646	Yes (Training data)	Yes	Yes
TCGA-GBM	TCGA-06-0648	No	N/A	Yes
TCGA-GBM	TCGA-06-0649	Yes (Testing data)	Yes	Yes
TCGA-GBM	TCGA-06-0878	Yes (Testing data)	No	No
TCGA-GBM	TCGA-06-0881	No	N/A	No
TCGA-GBM	TCGA-06-1084	Yes (Training data)	Yes	Yes
TCGA-GBM	TCGA-06-1801	Yes (Training data)	No	No
TCGA-GBM	TCGA-06-1802	Yes (Training data)	No	Yes
TCGA-GBM	TCGA-06-1806	No	N/A	No
TCGA-GBM	TCGA-06-2570	No	N/A	Yes
TCGA-GBM	TCGA-06-5408	Yes (Training data)	Yes	Yes
TCGA-GBM	TCGA-06-5410	No	N/A	No
TCGA-GBM	TCGA-06-5412	Yes (Testing data)	Yes	Yes
TCGA-GBM	TCGA-06-5413	Yes (Training data)	Yes	Yes
TCGA-GBM	TCGA-06-5417	No	N/A	Yes
TCGA-GBM	TCGA-06-6389	No	N/A	Yes
TCGA-GBM	TCGA-06-6701	No	N/A	No
TCGA-GBM	TCGA-08-0244	No	N/A	No
TCGA-GBM	TCGA-08-0246	No	N/A	No
TCGA-GBM	TCGA-08-0348	No	N/A	No
TCGA-GBM	TCGA-08-0349	No	N/A	No
TCGA-GBM	TCGA-08-0350	No	N/A	Yes
TCGA-GBM	TCGA-08-0351	No	N/A	No
TCGA-GBM	TCGA-08-0352	No	N/A	Yes
TCGA-GBM	TCGA-08-0353	No	N/A	Yes
TCGA-GBM	TCGA-08-0354	No	N/A	Yes
TCGA-GBM	TCGA-08-0355	Yes (Training data)	Yes	Yes
TCGA-GBM	TCGA-08-0356	Yes (Training data)	Yes	Yes
TCGA-GBM	TCGA-08-0357	No	N/A	Yes
TCGA-GBM	TCGA-08-0358	No	N/A	Yes
TCGA-GBM	TCGA-08-0359	Yes (Training data)	Yes	Yes
TCGA-GBM	TCGA-08-0360	Yes (Training data)	Yes	Yes
TCGA-GBM	TCGA-08-0380	No	N/A	No
TCGA-GBM	TCGA-08-0385	Yes (Training data)	Yes	Yes
TCGA-GBM	TCGA-08-0389	Yes (Training data)	Yes	Yes
TCGA-GBM	TCGA-08-0390	Yes (Training data)	Yes	Yes
TCGA-GBM	TCGA-08-0392	Yes (Training data)	Yes	Yes
TCGA-GBM	TCGA-08-0509	Yes (Training data)	Yes	Yes
TCGA-GBM	TCGA-08-0510	No	N/A	Yes
TCGA-GBM	TCGA-08-0511	No	N/A	No
TCGA-GBM	TCGA-08-0512	Yes (Training data)	Yes	Yes
TCGA-GBM	TCGA-08-0514	No	N/A	No
TCGA-GBM	TCGA-08-0516	No	N/A	No
TCGA-GBM	TCGA-08-0518	No	N/A	No
TCGA-GBM	TCGA-08-0520	Yes (Training data)	Yes	Yes
TCGA-GBM	TCGA-08-0521	No	N/A	Yes
TCGA-GBM	TCGA-08-0522	Yes (Training data)	Yes	Yes
TCGA-GBM	TCGA-08-0524	No	N/A	Yes
TCGA-GBM	TCGA-08-0529	Yes (Testing data)	Yes	Yes
TCGA-GBM	TCGA-12-0616	Yes (Training data)	Yes	Yes
TCGA-GBM	TCGA-12-0620	No	N/A	No
TCGA-GBM	TCGA-12-0769	No	N/A	No
TCGA-GBM	TCGA-12-0772	No	N/A	No
TCGA-GBM	TCGA-12-0773	No	N/A	No
TCGA-GBM	TCGA-12-0775	No	N/A	No
TCGA-GBM	TCGA-12-0776	Yes (Training data)	Yes	Yes
TCGA-GBM	TCGA-12-0829	Yes (Training data)	Yes	Yes
TCGA-GBM	TCGA-12-1092	No	N/A	No
TCGA-GBM	TCGA-12-1093	No	N/A	Yes
TCGA-GBM	TCGA-12-1094	Yes (Training data)	Yes	Yes
TCGA-GBM	TCGA-12-1096	Yes (Training data)	Yes	No
TCGA-GBM	TCGA-12-1097	No	N/A	No
TCGA-GBM	TCGA-12-1098	Yes (Training data)	Yes	Yes
TCGA-GBM	TCGA-12-1099	No	N/A	No
TCGA-GBM	TCGA-12-1598	Yes (Training data)	Yes	Yes
TCGA-GBM	TCGA-12-1599	No	N/A	No
TCGA-GBM	TCGA-12-1600	No	N/A	No
TCGA-GBM	TCGA-12-1601	Yes (Training data)	Yes	Yes
TCGA-GBM	TCGA-12-1602	No	N/A	Yes
TCGA-GBM	TCGA-12-3646	No	N/A	No
TCGA-GBM	TCGA-12-3648	No	N/A	No
TCGA-GBM	TCGA-12-3649	No	N/A	No
TCGA-GBM	TCGA-12-3650	Yes (Training data)	Yes	Yes
TCGA-GBM	TCGA-14-0736	No	N/A	No
TCGA-GBM	TCGA-14-0783	Yes (Training data)	Yes	No
TCGA-GBM	TCGA-14-0789	Yes (Testing data)	No	Yes
TCGA-GBM	TCGA-14-0790	Yes (Training data)	Yes	No
TCGA-GBM	TCGA-14-0812	Yes (Training data)	Yes	No
TCGA-GBM	TCGA-14-0813	Yes (Training data)	Yes	No
TCGA-GBM	TCGA-14-0817	Yes (Testing data)	No	No
TCGA-GBM	TCGA-14-0865	Yes (Training data)	Yes	No
TCGA-GBM	TCGA-14-0866	No	N/A	No
TCGA-GBM	TCGA-14-0871	Yes (Testing data)	Yes	No
TCGA-GBM	TCGA-14-1034	No	N/A	No
TCGA-GBM	TCGA-14-1037	Yes (Testing data)	No	No
TCGA-GBM	TCGA-14-1043	Yes (Training data)	Yes	No
TCGA-GBM	TCGA-14-1395	No	N/A	No
TCGA-GBM	TCGA-14-1396	No	N/A	No
TCGA-GBM	TCGA-14-1401	Yes (Training data)	No	No
TCGA-GBM	TCGA-14-1402	Yes (Testing data)	No	No
TCGA-GBM	TCGA-14-1452	Yes (Testing data)	No	No
TCGA-GBM	TCGA-14-1453	Yes (Training data)	No	No
TCGA-GBM	TCGA-14-1454	Yes (Training data)	No	No
TCGA-GBM	TCGA-14-1456	Yes (Training data)	Yes	Yes
TCGA-GBM	TCGA-14-1458	Yes (Training data)	No	No
TCGA-GBM	TCGA-14-1459	Yes (Training data)	No	No
TCGA-GBM	TCGA-14-1794	Yes (Training data)	Yes	Yes
TCGA-GBM	TCGA-14-1795	Yes (Testing data)	No	No
TCGA-GBM	TCGA-14-1821	Yes (Testing data)	No	No
TCGA-GBM	TCGA-14-1823	Yes (Training data)	Yes	No
TCGA-GBM	TCGA-14-1825	Yes (Training data)	Yes	Yes
TCGA-GBM	TCGA-14-1829	Yes (Testing data)	No	Yes
TCGA-GBM	TCGA-14-2555	Yes (Training data)	No	No
TCGA-GBM	TCGA-14-3477	Yes (Training data)	Yes	Yes
TCGA-GBM	TCGA-19-0955	Yes (Training data)	Yes	No
TCGA-GBM	TCGA-19-0957	No	N/A	No
TCGA-GBM	TCGA-19-0960	No	N/A	No
TCGA-GBM	TCGA-19-0962	No	N/A	No
TCGA-GBM	TCGA-19-0963	Yes (Training data)	Yes	Yes
TCGA-GBM	TCGA-19-0964	No	N/A	No
TCGA-GBM	TCGA-19-1385	No	N/A	No
TCGA-GBM	TCGA-19-1386	No	N/A	No
TCGA-GBM	TCGA-19-1387	No	N/A	No
TCGA-GBM	TCGA-19-1388	Yes (Training data)	Yes	No
TCGA-GBM	TCGA-19-1389	No	N/A	No
TCGA-GBM	TCGA-19-1390	No	N/A	Yes
TCGA-GBM	TCGA-19-1392	No	N/A	No
TCGA-GBM	TCGA-19-1787	No	N/A	No
TCGA-GBM	TCGA-19-1788	No	N/A	No
TCGA-GBM	TCGA-19-1789	Yes (Training data)	Yes	Yes
TCGA-GBM	TCGA-19-1791	No	N/A	No
TCGA-GBM	TCGA-19-2619	No	N/A	No
TCGA-GBM	TCGA-19-2620	No	N/A	No
TCGA-GBM	TCGA-19-2621	No	N/A	No
TCGA-GBM	TCGA-19-2623	No	N/A	No
TCGA-GBM	TCGA-19-2624	Yes (Training data)	Yes	Yes
TCGA-GBM	TCGA-19-2625	No	N/A	No
TCGA-GBM	TCGA-19-2629	No	N/A	No
TCGA-GBM	TCGA-19-2631	Yes (Training data)	Yes	Yes
TCGA-GBM	TCGA-19-4065	No	N/A	No
TCGA-GBM	TCGA-19-4068	No	N/A	No
TCGA-GBM	TCGA-19-5947	No	N/A	No
TCGA-GBM	TCGA-19-5950	Yes (Training data)	Yes	No
TCGA-GBM	TCGA-19-5951	Yes (Training data)	Yes	Yes
TCGA-GBM	TCGA-19-5952	No	N/A	No
TCGA-GBM	TCGA-19-5953	No	N/A	No
TCGA-GBM	TCGA-19-5954	Yes (Training data)	Yes	Yes
TCGA-GBM	TCGA-19-5955	No	N/A	No
TCGA-GBM	TCGA-19-5956	No	N/A	No
TCGA-GBM	TCGA-19-5958	No	N/A	Yes
TCGA-GBM	TCGA-19-5959	No	N/A	No
TCGA-GBM	TCGA-19-5960	Yes (Training data)	Yes	Yes
TCGA-GBM	TCGA-27-1830	No	N/A	No
TCGA-GBM	TCGA-27-1831	No	N/A	No
TCGA-GBM	TCGA-27-1833	No	N/A	No
TCGA-GBM	TCGA-27-1834	No	N/A	Yes
TCGA-GBM	TCGA-27-1835	No	N/A	No
TCGA-GBM	TCGA-27-1836	No	N/A	No
TCGA-GBM	TCGA-27-1837	No	N/A	No
TCGA-GBM	TCGA-27-1838	No	N/A	Yes
TCGA-GBM	TCGA-27-2518	No	N/A	No
TCGA-GBM	TCGA-27-2519	No	N/A	No
TCGA-GBM	TCGA-27-2521	No	N/A	No
TCGA-GBM	TCGA-27-2523	No	N/A	No
TCGA-GBM	TCGA-27-2524	No	N/A	No
TCGA-GBM	TCGA-27-2526	No	N/A	Yes
TCGA-GBM	TCGA-27-2527	No	N/A	No
TCGA-GBM	TCGA-27-2528	No	N/A	No
TCGA-GBM	TCGA-76-4925	No	N/A	No
TCGA-GBM	TCGA-76-4926	No	N/A	No
TCGA-GBM	TCGA-76-4927	No	N/A	No
TCGA-GBM	TCGA-76-4928	No	N/A	No
TCGA-GBM	TCGA-76-4929	No	N/A	No
TCGA-GBM	TCGA-76-4931	No	N/A	No
TCGA-GBM	TCGA-76-4932	Yes (Training data)	Yes	Yes
TCGA-GBM	TCGA-76-4934	Yes (Training data)	Yes	Yes
TCGA-GBM	TCGA-76-4935	Yes (Training data)	Yes	Yes
TCGA-GBM	TCGA-76-6191	Yes (Training data)	Yes	Yes
TCGA-GBM	TCGA-76-6192	No	N/A	No
TCGA-GBM	TCGA-76-6193	Yes (Training data)	Yes	Yes
TCGA-GBM	TCGA-76-6280	Yes (Training data)	Yes	Yes
TCGA-GBM	TCGA-76-6282	Yes (Training data)	Yes	Yes
TCGA-GBM	TCGA-76-6285	Yes (Training data)	Yes	Yes
TCGA-GBM	TCGA-76-6286	No	N/A	No
TCGA-GBM	TCGA-76-6656	Yes (Training data)	Yes	Yes
TCGA-GBM	TCGA-76-6657	Yes (Training data)	Yes	Yes
TCGA-GBM	TCGA-76-6661	Yes (Training data)	Yes	Yes
TCGA-GBM	TCGA-76-6662	Yes (Training data)	Yes	Yes
TCGA-GBM	TCGA-76-6663	Yes (Training data)	Yes	Yes
TCGA-GBM	TCGA-76-6664	Yes (Training data)	Yes	Yes
TCGA-LGG	TCGA-CS-4938	No	N/A	No
TCGA-LGG	TCGA-CS-4941	Yes (Testing data)	Yes	Yes
TCGA-LGG	TCGA-CS-4942	Yes (Training data)	Yes	Yes
TCGA-LGG	TCGA-CS-4943	No	N/A	Yes
TCGA-LGG	TCGA-CS-4944	Yes (Training data)	Yes	Yes
TCGA-LGG	TCGA-CS-5390	No	N/A	No
TCGA-LGG	TCGA-CS-5393	Yes (Training data)	Yes	Yes
TCGA-LGG	TCGA-CS-5394	No	N/A	No
TCGA-LGG	TCGA-CS-5395	Yes (Testing data)	Yes	Yes
TCGA-LGG	TCGA-CS-5396	Yes (Training data)	Yes	Yes
TCGA-LGG	TCGA-CS-5397	Yes (Training data)	Yes	Yes
TCGA-LGG	TCGA-CS-6186	Yes (Training data)	Yes	Yes
TCGA-LGG	TCGA-CS-6188	No	N/A	Yes
TCGA-LGG	TCGA-CS-6290	Yes (Testing data)	Yes	Yes
TCGA-LGG	TCGA-CS-6665	Yes (Training data)	Yes	Yes
TCGA-LGG	TCGA-CS-6666	Yes (Training data)	Yes	Yes
TCGA-LGG	TCGA-CS-6667	Yes (Testing data)	Yes	Yes
TCGA-LGG	TCGA-CS-6668	Yes (Training data)	Yes	Yes
TCGA-LGG	TCGA-CS-6669	Yes (Training data)	Yes	Yes
TCGA-LGG	TCGA-CS-6670	Yes (Training data)	Yes	No
TCGA-LGG	TCGA-DU-5849	Yes (Testing data)	Yes	Yes
TCGA-LGG	TCGA-DU-5851	Yes (Training data)	Yes	Yes
TCGA-LGG	TCGA-DU-5852	Yes (Testing data)	Yes	Yes
TCGA-LGG	TCGA-DU-5853	Yes (Testing data)	Yes	Yes
TCGA-LGG	TCGA-DU-5854	Yes (Training data)	Yes	Yes
TCGA-LGG	TCGA-DU-5855	Yes (Training data)	Yes	Yes
TCGA-LGG	TCGA-DU-5871	Yes (Testing data)	Yes	Yes
TCGA-LGG	TCGA-DU-5872	Yes (Training data)	Yes	Yes
TCGA-LGG	TCGA-DU-5874	Yes (Training data)	Yes	Yes
TCGA-LGG	TCGA-DU-6395	No	N/A	No
TCGA-LGG	TCGA-DU-6397	Yes (Testing data)	Yes	Yes
TCGA-LGG	TCGA-DU-6399	Yes (Testing data)	Yes	Yes
TCGA-LGG	TCGA-DU-6400	Yes (Testing data)	Yes	Yes
TCGA-LGG	TCGA-DU-6401	Yes (Testing data)	Yes	Yes
TCGA-LGG	TCGA-DU-6402	No	N/A	No
TCGA-LGG	TCGA-DU-6404	Yes (Training data)	Yes	Yes
TCGA-LGG	TCGA-DU-6405	No	N/A	Yes
TCGA-LGG	TCGA-DU-6407	No	N/A	Yes
TCGA-LGG	TCGA-DU-6408	No	N/A	Yes
TCGA-LGG	TCGA-DU-6410	No	N/A	Yes
TCGA-LGG	TCGA-DU-6542	Yes (Training data)	Yes	Yes
TCGA-LGG	TCGA-DU-7008	Yes (Training data)	Yes	Yes
TCGA-LGG	TCGA-DU-7010	Yes (Training data)	Yes	Yes
TCGA-LGG	TCGA-DU-7013	No	N/A	No
TCGA-LGG	TCGA-DU-7014	Yes (Training data)	Yes	Yes
TCGA-LGG	TCGA-DU-7015	Yes (Training data)	Yes	Yes
TCGA-LGG	TCGA-DU-7018	Yes (Training data)	Yes	Yes
TCGA-LGG	TCGA-DU-7019	Yes (Training data)	Yes	Yes
TCGA-LGG	TCGA-DU-7294	Yes (Training data)	Yes	Yes
TCGA-LGG	TCGA-DU-7298	Yes (Training data)	Yes	Yes
TCGA-LGG	TCGA-DU-7299	Yes (Training data)	Yes	Yes
TCGA-LGG	TCGA-DU-7300	Yes (Training data)	Yes	Yes
TCGA-LGG	TCGA-DU-7301	Yes (Training data)	Yes	Yes
TCGA-LGG	TCGA-DU-7302	Yes (Training data)	Yes	Yes
TCGA-LGG	TCGA-DU-7304	Yes (Training data)	Yes	Yes
TCGA-LGG	TCGA-DU-7306	Yes (Training data)	Yes	Yes
TCGA-LGG	TCGA-DU-7309	Yes (Training data)	Yes	Yes
TCGA-LGG	TCGA-DU-8158	No	N/A	No
TCGA-LGG	TCGA-DU-8162	No	N/A	Yes
TCGA-LGG	TCGA-DU-8163	Yes (Training data)	Yes	No
TCGA-LGG	TCGA-DU-8164	Yes (Training data)	Yes	Yes
TCGA-LGG	TCGA-DU-8165	No	N/A	Yes
TCGA-LGG	TCGA-DU-8166	Yes (Training data)	Yes	Yes
TCGA-LGG	TCGA-DU-8167	Yes (Training data)	Yes	Yes
TCGA-LGG	TCGA-DU-8168	Yes (Training data)	Yes	Yes
TCGA-LGG	TCGA-DU-A5TP	No	N/A	Yes
TCGA-LGG	TCGA-DU-A5TR	No	N/A	Yes
TCGA-LGG	TCGA-DU-A5TS	No	N/A	Yes
TCGA-LGG	TCGA-DU-A5TT	No	N/A	Yes
TCGA-LGG	TCGA-DU-A5TU	No	N/A	Yes
TCGA-LGG	TCGA-DU-A5TW	No	N/A	Yes
TCGA-LGG	TCGA-DU-A5TY	No	N/A	Yes
TCGA-LGG	TCGA-DU-A6S2	No	N/A	Yes
TCGA-LGG	TCGA-DU-A6S3	No	N/A	Yes
TCGA-LGG	TCGA-DU-A6S6	No	N/A	Yes
TCGA-LGG	TCGA-DU-A6S7	No	N/A	Yes
TCGA-LGG	TCGA-DU-A6S8	No	N/A	Yes
TCGA-LGG	TCGA-EZ-7264A	No	N/A	No
TCGA-LGG	TCGA-EZ-7265A	No	N/A	Yes
TCGA-LGG	TCGA-FG-5962	No	N/A	No
TCGA-LGG	TCGA-FG-5963	No	N/A	No
TCGA-LGG	TCGA-FG-5964	Yes (Training data)	Yes	Yes
TCGA-LGG	TCGA-FG-5965	No	N/A	No
TCGA-LGG	TCGA-FG-6688	No	N/A	Yes
TCGA-LGG	TCGA-FG-6689	Yes (Training data)	Yes	Yes
TCGA-LGG	TCGA-FG-6690	Yes (Testing data)	Yes	No
TCGA-LGG	TCGA-FG-6691	Yes (Training data)	Yes	Yes
TCGA-LGG	TCGA-FG-6692	Yes (Training data)	Yes	Yes
TCGA-LGG	TCGA-FG-7634	Yes (Training data)	Yes	Yes
TCGA-LGG	TCGA-FG-7637	Yes (Testing data)	Yes	No
TCGA-LGG	TCGA-FG-7641	No	N/A	No
TCGA-LGG	TCGA-FG-7643	Yes (Testing data)	Yes	Yes
TCGA-LGG	TCGA-FG-8186	No	N/A	No
TCGA-LGG	TCGA-FG-8189	No	N/A	No
TCGA-LGG	TCGA-FG-A4MT	Yes (Training data)	Yes	Yes
TCGA-LGG	TCGA-FG-A4MU	No	N/A	No
TCGA-LGG	TCGA-FG-A60K	No	N/A	No
TCGA-LGG	TCGA-FG-A6IZ	No	N/A	Yes
TCGA-LGG	TCGA-FG-A6J1	No	N/A	No
TCGA-LGG	TCGA-FG-A713	No	N/A	Yes
TCGA-LGG	TCGA-FG-A87N	No	N/A	No
TCGA-LGG	TCGA-HT-7467	No	N/A	No
TCGA-LGG	TCGA-HT-7468	No	N/A	No
TCGA-LGG	TCGA-HT-7469	No	N/A	No
TCGA-LGG	TCGA-HT-7470	No	N/A	No
TCGA-LGG	TCGA-HT-7471	No	N/A	No
TCGA-LGG	TCGA-HT-7472	No	N/A	No
TCGA-LGG	TCGA-HT-7473	No	N/A	Yes
TCGA-LGG	TCGA-HT-7474	No	N/A	No
TCGA-LGG	TCGA-HT-7475	No	N/A	Yes
TCGA-LGG	TCGA-HT-7476	No	N/A	No
TCGA-LGG	TCGA-HT-7477	No	N/A	No
TCGA-LGG	TCGA-HT-7478	No	N/A	No
TCGA-LGG	TCGA-HT-7479	No	N/A	No
TCGA-LGG	TCGA-HT-7480	No	N/A	No
TCGA-LGG	TCGA-HT-7481	No	N/A	No
TCGA-LGG	TCGA-HT-7482	No	N/A	No
TCGA-LGG	TCGA-HT-7483	No	N/A	No
TCGA-LGG	TCGA-HT-7485	No	N/A	No
TCGA-LGG	TCGA-HT-7601	No	N/A	No
TCGA-LGG	TCGA-HT-7602	No	N/A	Yes
TCGA-LGG	TCGA-HT-7603	No	N/A	No
TCGA-LGG	TCGA-HT-7604	No	N/A	No
TCGA-LGG	TCGA-HT-7605	No	N/A	No
TCGA-LGG	TCGA-HT-7606	No	N/A	No
TCGA-LGG	TCGA-HT-7607	No	N/A	No
TCGA-LGG	TCGA-HT-7608	No	N/A	No
TCGA-LGG	TCGA-HT-7609	No	N/A	No
TCGA-LGG	TCGA-HT-7610	No	N/A	No
TCGA-LGG	TCGA-HT-7611	No	N/A	No
TCGA-LGG	TCGA-HT-7616	No	N/A	Yes
TCGA-LGG	TCGA-HT-7620	No	N/A	No
TCGA-LGG	TCGA-HT-7676	No	N/A	No
TCGA-LGG	TCGA-HT-7677	No	N/A	No
TCGA-LGG	TCGA-HT-7680	No	N/A	Yes
TCGA-LGG	TCGA-HT-7681	No	N/A	No
TCGA-LGG	TCGA-HT-7684	No	N/A	Yes
TCGA-LGG	TCGA-HT-7686	No	N/A	Yes
TCGA-LGG	TCGA-HT-7687	No	N/A	No
TCGA-LGG	TCGA-HT-7688	No	N/A	No
TCGA-LGG	TCGA-HT-7689	No	N/A	No
TCGA-LGG	TCGA-HT-7690	No	N/A	Yes
TCGA-LGG	TCGA-HT-7691	No	N/A	No
TCGA-LGG	TCGA-HT-7692	No	N/A	Yes
TCGA-LGG	TCGA-HT-7693	No	N/A	Yes
TCGA-LGG	TCGA-HT-7694	No	N/A	Yes
TCGA-LGG	TCGA-HT-7695	No	N/A	No
TCGA-LGG	TCGA-HT-7854	No	N/A	No
TCGA-LGG	TCGA-HT-7855	No	N/A	Yes
TCGA-LGG	TCGA-HT-7856	No	N/A	Yes
TCGA-LGG	TCGA-HT-7857	No	N/A	No
TCGA-LGG	TCGA-HT-7858	No	N/A	No
TCGA-LGG	TCGA-HT-7860	No	N/A	Yes
TCGA-LGG	TCGA-HT-7873	No	N/A	No
TCGA-LGG	TCGA-HT-7874	No	N/A	Yes
TCGA-LGG	TCGA-HT-7875	No	N/A	No
TCGA-LGG	TCGA-HT-7877	No	N/A	No
TCGA-LGG	TCGA-HT-7879	No	N/A	Yes
TCGA-LGG	TCGA-HT-7880	No	N/A	No
TCGA-LGG	TCGA-HT-7881	No	N/A	No
TCGA-LGG	TCGA-HT-7882	No	N/A	Yes
TCGA-LGG	TCGA-HT-7884	No	N/A	Yes
TCGA-LGG	TCGA-HT-7902	No	N/A	No
TCGA-LGG	TCGA-HT-8010	No	N/A	No
TCGA-LGG	TCGA-HT-8011	No	N/A	No
TCGA-LGG	TCGA-HT-8012	No	N/A	No
TCGA-LGG	TCGA-HT-8013	No	N/A	No
TCGA-LGG	TCGA-HT-8015	No	N/A	No
TCGA-LGG	TCGA-HT-8018	No	N/A	Yes
TCGA-LGG	TCGA-HT-8019	No	N/A	No
TCGA-LGG	TCGA-HT-8104	No	N/A	No
TCGA-LGG	TCGA-HT-8105	No	N/A	Yes
TCGA-LGG	TCGA-HT-8106	No	N/A	Yes
TCGA-LGG	TCGA-HT-8107	No	N/A	Yes
TCGA-LGG	TCGA-HT-8108	No	N/A	No
TCGA-LGG	TCGA-HT-8109	No	N/A	No
TCGA-LGG	TCGA-HT-8110	No	N/A	No
TCGA-LGG	TCGA-HT-8111	No	N/A	Yes
TCGA-LGG	TCGA-HT-8113	No	N/A	Yes
TCGA-LGG	TCGA-HT-8114	No	N/A	Yes
TCGA-LGG	TCGA-HT-8558	No	N/A	No
TCGA-LGG	TCGA-HT-8563	No	N/A	Yes
TCGA-LGG	TCGA-HT-8564	No	N/A	No
TCGA-LGG	TCGA-HT-A4DS	No	N/A	No
TCGA-LGG	TCGA-HT-A4DV	No	N/A	No
TCGA-LGG	TCGA-HT-A5R5	No	N/A	No
TCGA-LGG	TCGA-HT-A5R7	No	N/A	No
TCGA-LGG	TCGA-HT-A5RA	No	N/A	No
TCGA-LGG	TCGA-HT-A5RB	No	N/A	No
TCGA-LGG	TCGA-HT-A5RC	No	N/A	Yes
TCGA-LGG	TCGA-HT-A614	No	N/A	Yes
TCGA-LGG	TCGA-HT-A615	No	N/A	No
TCGA-LGG	TCGA-HT-A616	No	N/A	No
TCGA-LGG	TCGA-HT-A617	No	N/A	No
TCGA-LGG	TCGA-HT-A618	No	N/A	No
TCGA-LGG	TCGA-HT-A619	No	N/A	No
TCGA-LGG	TCGA-HT-A61A	No	N/A	Yes
TCGA-LGG	TCGA-HT-A61B	No	N/A	No
TCGA-LGG	TCGA-HT-A61C	No	N/A	No

**Table 3:** Generated radiomic features and their description

**Collection**	**Site**	**Patient ID**	**GivenModalityForThisStudy**	**Sex**	**Age**	**Weight**	**ModalityName**	**SeriesNum**	**AccessionNum**	**Acq/Study/SeriesDate**	**Manufacturer**	**StationName**	**ManufacturerModelName**	**ScanSequence**	**AcquisitionType**	**SliceThickness**	**RepetitionTime**	**EchoTime**	**InversionTime**	**ImagingFreq**	**MagneticFieldStrength**	**SliceSpacing**	**FlipAngle**	**SpecificAbsorptionRate**	**Slices**	**AcqMatRows**	**AcqMatCols**	**PixDim**	**Series/Modalities_ID**
TCGA-GBM	TCGA-02	TCGA-02-0003	flair	M	50	99.79	AX_FLAIR	11	2.8195E+15	6/8/1997	GE MEDICAL SYSTEMS		GENESIS_SIGNA	IR	2D	5	10002	147	2200	6.39E+08	1.5	6.5	90	0.932588	25	256	160	0.781250\0.781250	TCGA-02-0003/1.3.6.1.4.1.14519.5.2.1.1706.4001.145725991542758792340793681239/1.3.6.1.4.1.14519.5.2.1.1706.4001.166909646801864710106680106577
TCGA-GBM	TCGA-02	TCGA-02-0003	t1Gd	M	50	99.79	AX_T1_POST	13	2.8195E+15	6/8/1997	GE MEDICAL SYSTEMS		GENESIS_SIGNA	SE	2D	5	650	9		6.39E+08	1.5	6.5	90	1.972841	25	256	192	0.781250\0.781250	TCGA-02-0003/1.3.6.1.4.1.14519.5.2.1.1706.4001.145725991542758792340793681239/1.3.6.1.4.1.14519.5.2.1.1706.4001.204054528317611873722753919694
TCGA-GBM	TCGA-02	TCGA-02-0003	t1	M	50	99.79	AX_T1	12	2.8195E+15	6/8/1997	GE MEDICAL SYSTEMS		GENESIS_SIGNA	SE	2D	5	533.332	8		6.39E+08	1.5	6.5	90	1.959656	25	256	128	0.781250\0.781250	TCGA-02-0003/1.3.6.1.4.1.14519.5.2.1.1706.4001.145725991542758792340793681239/1.3.6.1.4.1.14519.5.2.1.1706.4001.769579767788431533139923490444
TCGA-GBM	TCGA-02	TCGA-02-0003	t2	M	50	99.79	AX_T2_FSE	10	2.8195E+15	6/8/1997	GE MEDICAL SYSTEMS		GENESIS_SIGNA	SE	2D	5	3500	105		6.39E+08	1.5	6.5	90	1.697364	25	256	224	0.781250\0.781250	TCGA-02-0003/1.3.6.1.4.1.14519.5.2.1.1706.4001.145725991542758792340793681239/1.3.6.1.4.1.14519.5.2.1.1706.4001.643840351201993277528058523822
TCGA-GBM	TCGA-02	TCGA-02-0006	flair	F	56	70.307	AX_FLAIR	4	2.8195E+15	8/23/1996	GE MEDICAL SYSTEMS		GENESIS_SIGNA	IR	2D	5	10002	147	2200	6.38E+08	1.5	6.5	90	0.023404	23	256	160	0.781250\0.781250	TCGA-02-0006/1.3.6.1.4.1.14519.5.2.1.1706.4001.149500105036523046215258942545/1.3.6.1.4.1.14519.5.2.1.1706.4001.315980779833795710131738723922
TCGA-GBM	TCGA-02	TCGA-02-0006	t1Gd	F	56	70.307	AX_T1_POST	6	2.8195E+15	8/23/1996	GE MEDICAL SYSTEMS		GENESIS_SIGNA	SE	2D	5	583.332	8		6.38E+08	1.5	6.5	90	0.059235	23	256	192	0.781250\0.781250	TCGA-02-0006/1.3.6.1.4.1.14519.5.2.1.1706.4001.149500105036523046215258942545/1.3.6.1.4.1.14519.5.2.1.1706.4001.170251499565901046332998726504
TCGA-GBM	TCGA-02	TCGA-02-0006	t1	F	56	70.307	AX_T1	5	2.8195E+15	8/23/1996	GE MEDICAL SYSTEMS		GENESIS_SIGNA	SE	2D	5	416.664	8		6.38E+08	1.5	6.5	90	0.067638	23	256	128	0.781250\0.781250	TCGA-02-0006/1.3.6.1.4.1.14519.5.2.1.1706.4001.149500105036523046215258942545/1.3.6.1.4.1.14519.5.2.1.1706.4001.283912726091613148226144162419
TCGA-GBM	TCGA-02	TCGA-02-0006	t2	F	56	70.307	AX_T2_FSE	3	2.8195E+15	8/23/1996	GE MEDICAL SYSTEMS		GENESIS_SIGNA	SE	2D	5	3500	98		6.38E+08	1.5	6.5	90	0.04572	23	256	224	0.781250\0.781250	TCGA-02-0006/1.3.6.1.4.1.14519.5.2.1.1706.4001.149500105036523046215258942545/1.3.6.1.4.1.14519.5.2.1.1706.4001.929166576410729967676059227597
TCGA-GBM	TCGA-02	TCGA-02-0009	flair	F	61	63.503	AX_FLAIR	4	2.8195E+15	6/14/1997	GE MEDICAL SYSTEMS		GENESIS_SIGNA	IR	2D	5	10002	147	2200	6.39E+08	1.5	6.5	90	0.023788	23	256	160	0.781250\0.781250	TCGA-02-0009/1.3.6.1.4.1.14519.5.2.1.1706.4001.743358002952086773602945013452/1.3.6.1.4.1.14519.5.2.1.1706.4001.779013211760175351323192631290
TCGA-GBM	TCGA-02	TCGA-02-0009	t1Gd	F	61	63.503	AX_T1_POST	6	2.8195E+15	6/14/1997	GE MEDICAL SYSTEMS		GENESIS_SIGNA	SE	2D	5	583.332	8		6.39E+08	1.5	6.5	90	0.060207	23	256	192	0.781250\0.781250	TCGA-02-0009/1.3.6.1.4.1.14519.5.2.1.1706.4001.743358002952086773602945013452/1.3.6.1.4.1.14519.5.2.1.1706.4001.150346522548097750188958570502
TCGA-GBM	TCGA-02	TCGA-02-0009	t1	F	61	63.503	AX_T1	5	2.8195E+15	6/14/1997	GE MEDICAL SYSTEMS		GENESIS_SIGNA	SE	2D	5	416.664	8		6.39E+08	1.5	6.5	90	0.068749	23	256	128	0.781250\0.781250	TCGA-02-0009/1.3.6.1.4.1.14519.5.2.1.1706.4001.743358002952086773602945013452/1.3.6.1.4.1.14519.5.2.1.1706.4001.507509430627489084746877138458
TCGA-GBM	TCGA-02	TCGA-02-0009	t2	F	61	63.503	AX_T2_FSE	3	2.8195E+15	6/14/1997	GE MEDICAL SYSTEMS		GENESIS_SIGNA	SE	2D	5	3500	98		6.39E+08	1.5	6.5	90	0.04647	23	256	224	0.781250\0.781250	TCGA-02-0009/1.3.6.1.4.1.14519.5.2.1.1706.4001.743358002952086773602945013452/1.3.6.1.4.1.14519.5.2.1.1706.4001.466873465067673546718804101423
TCGA-GBM	TCGA-02	TCGA-02-0011	flair	F	18	51.256	AX_FLAIR	4	2.8195E+15	2/1/1998	GE MEDICAL SYSTEMS		GENESIS_SIGNA	IR	2D	5	10002	147	2200	6.39E+08	1.5	6.5	90	0.024617	23	256	160	0.781250\0.781250	TCGA-02-0011/1.3.6.1.4.1.14519.5.2.1.1706.4001.338073323505507625300877831709/1.3.6.1.4.1.14519.5.2.1.1706.4001.245399232039099875265266953905
TCGA-GBM	TCGA-02	TCGA-02-0011	t1Gd	F	18	51.256	AX_T1_POST	6	2.8195E+15	2/1/1998	GE MEDICAL SYSTEMS		GENESIS_SIGNA	SE	2D	5	583.332	8		6.39E+08	1.5	6.5	90	0.062307	23	256	192	0.781250\0.781250	TCGA-02-0011/1.3.6.1.4.1.14519.5.2.1.1706.4001.338073323505507625300877831709/1.3.6.1.4.1.14519.5.2.1.1706.4001.209353288390362628068712523284
TCGA-GBM	TCGA-02	TCGA-02-0011	t1	F	18	51.256	AX_T1	5	2.8195E+15	2/1/1998	GE MEDICAL SYSTEMS		GENESIS_SIGNA	SE	2D	5	416.664	8		6.39E+08	1.5	6.5	90	0.071146	23	256	128	0.781250\0.781250	TCGA-02-0011/1.3.6.1.4.1.14519.5.2.1.1706.4001.338073323505507625300877831709/1.3.6.1.4.1.14519.5.2.1.1706.4001.101129210060381531266477375594
TCGA-GBM	TCGA-02	TCGA-02-0011	t2	F	18	51.256	AX_T2_FSE	3	2.8195E+15	2/1/1998	GE MEDICAL SYSTEMS		GENESIS_SIGNA	SE	2D	5	3500	98		6.39E+08	1.5	6.5	90	0.048091	23	256	224	0.781250\0.781250	TCGA-02-0011/1.3.6.1.4.1.14519.5.2.1.1706.4001.338073323505507625300877831709/1.3.6.1.4.1.14519.5.2.1.1706.4001.222837342553572147643639077488
TCGA-GBM	TCGA-02	TCGA-02-0027	flair	F	33	61.689	T2_FLAIR	4	2.8195E+15	3/28/1999	GE MEDICAL SYSTEMS		SIGNA EXCITE	IR	2D	5	10002	140.244	2250	127.7415	3	6.5	90	0.5753	23	320	224	0.46875\0.46875	TCGA-02-0027/1.3.6.1.4.1.14519.5.2.1.1706.4001.190188151913002985587952372782/1.3.6.1.4.1.14519.5.2.1.1706.4001.218172129878062618773517653321
TCGA-GBM	TCGA-02	TCGA-02-0027	t1Gd	F	33	61.689	FSPGR_3D	10	2.8195E+15	3/28/1999	GE MEDICAL SYSTEMS		SIGNA EXCITE	GR	3D	1.5	5.872	2.1	400	127.7415	3	1.5	20	0.709214	124	320	224	0.488281\0.488281	TCGA-02-0027/1.3.6.1.4.1.14519.5.2.1.1706.4001.190188151913002985587952372782/1.3.6.1.4.1.14519.5.2.1.1706.4001.227653997506797488566901323094
TCGA-GBM	TCGA-02	TCGA-02-0027	t1	F	33	61.689	SE_T1	5	2.8195E+15	3/28/1999	GE MEDICAL SYSTEMS		SIGNA EXCITE	SE	2D	5	816.664	12	0	127.7415	3	6.5	90	1.755603	23	320	224	0.46875\0.46875	TCGA-02-0027/1.3.6.1.4.1.14519.5.2.1.1706.4001.190188151913002985587952372782/1.3.6.1.4.1.14519.5.2.1.1706.4001.206413929564700806222369874836
TCGA-GBM	TCGA-02	TCGA-02-0027	t2	F	33	61.689	FSE_T2	3	2.8195E+15	3/28/1999	GE MEDICAL SYSTEMS		SIGNA EXCITE	SE	2D	5	5200	99	0	127.7415	3	6.5	90	1.2148	23	320	256	0.46875\0.46875	TCGA-02-0027/1.3.6.1.4.1.14519.5.2.1.1706.4001.190188151913002985587952372782/1.3.6.1.4.1.14519.5.2.1.1706.4001.198656437193522367812015571836
TCGA-GBM	TCGA-02	TCGA-02-0033	flair	M	54	88.904	AX_FLAIR	4	2.8195E+15	5/26/1997	GE MEDICAL SYSTEMS		GENESIS_SIGNA	IR	2D	5	10002	147	2200	6.39E+08	1.5	6.5	90	0.022541	23	256	160	0.781250\0.781250	TCGA-02-0033/1.3.6.1.4.1.14519.5.2.1.1706.4001.211135800355624542661804589744/1.3.6.1.4.1.14519.5.2.1.1706.4001.116289242878713280880004720679
TCGA-GBM	TCGA-02	TCGA-02-0033	t1Gd	M	54	88.904	AX_T1_POST	6	2.8195E+15	5/26/1997	GE MEDICAL SYSTEMS		GENESIS_SIGNA	SE	2D	5	583.332	8		6.39E+08	1.5	6.5	90	0.057052	23	256	192	0.781250\0.781250	TCGA-02-0033/1.3.6.1.4.1.14519.5.2.1.1706.4001.211135800355624542661804589744/1.3.6.1.4.1.14519.5.2.1.1706.4001.287753826073472752590065451465
TCGA-GBM	TCGA-02	TCGA-02-0033	t1	M	54	88.904	AX_T1	5	2.8195E+15	5/26/1997	GE MEDICAL SYSTEMS		GENESIS_SIGNA	SE	2D	5	416.664	8		6.39E+08	1.5	6.5	90	0.065145	23	256	128	0.781250\0.781250	TCGA-02-0033/1.3.6.1.4.1.14519.5.2.1.1706.4001.211135800355624542661804589744/1.3.6.1.4.1.14519.5.2.1.1706.4001.103174687731052142735983046836
TCGA-GBM	TCGA-02	TCGA-02-0033	t2	M	54	88.904	AX_T2_FSE	3	2.8195E+15	5/26/1997	GE MEDICAL SYSTEMS		GENESIS_SIGNA	SE	2D	5	3500	98		6.39E+08	1.5	6.5	90	0.044035	23	256	224	0.781250\0.781250	TCGA-02-0033/1.3.6.1.4.1.14519.5.2.1.1706.4001.211135800355624542661804589744/1.3.6.1.4.1.14519.5.2.1.1706.4001.261091685370401963952512175444
TCGA-GBM	TCGA-02	TCGA-02-0034	flair	M	60	91.626	AX_FLAIR	11	2.8195E+15	7/27/1997	GE MEDICAL SYSTEMS		GENESIS_SIGNA	IR	2D	5	10002	147	2200	6.39E+08	1.5	6.5	90	0.769412	24	256	160	0.781250\0.781250	TCGA-02-0034/1.3.6.1.4.1.14519.5.2.1.1706.4001.278960043528371206673900239956/1.3.6.1.4.1.14519.5.2.1.1706.4001.287132853761458303765658181489
TCGA-GBM	TCGA-02	TCGA-02-0034	t1Gd	M	60	91.626	AX_T1_POST	13	2.8195E+15	7/27/1997	GE MEDICAL SYSTEMS		GENESIS_SIGNA	SE	2D	5	616.664	9		6.39E+08	1.5	6.5	90	1.921836	24	256	192	0.781250\0.781250	TCGA-02-0034/1.3.6.1.4.1.14519.5.2.1.1706.4001.278960043528371206673900239956/1.3.6.1.4.1.14519.5.2.1.1706.4001.497428918591782710551794403821
TCGA-GBM	TCGA-02	TCGA-02-0034	t1	M	60	91.626	AX_T1	12	2.8195E+15	7/27/1997	GE MEDICAL SYSTEMS		GENESIS_SIGNA	SE	2D	5	483.332	8		6.39E+08	1.5	6.5	90	1.99891	24	256	128	0.781250\0.781250	TCGA-02-0034/1.3.6.1.4.1.14519.5.2.1.1706.4001.278960043528371206673900239956/1.3.6.1.4.1.14519.5.2.1.1706.4001.135129750377370879091413746175
TCGA-GBM	TCGA-02	TCGA-02-0034	t2	M	60	91.626	AX_T2_FSE	10	2.8195E+15	7/27/1997	GE MEDICAL SYSTEMS		GENESIS_SIGNA	SE	2D	5	3500	105		6.39E+08	1.5	6.5	90	1.568419	24	256	224	0.781250\0.781250	TCGA-02-0034/1.3.6.1.4.1.14519.5.2.1.1706.4001.278960043528371206673900239956/1.3.6.1.4.1.14519.5.2.1.1706.4001.185929810886679504848373001030
TCGA-GBM	TCGA-02	TCGA-02-0037	flair	F	74	109.769	AX_FLAIR	4	2.8195E+15	1/13/1998	GE MEDICAL SYSTEMS		GENESIS_SIGNA	IR	2D	5	10002	147	2200	6.39E+08	1.5	6.5	90	0.021793	23	256	160	0.781250\0.781250	TCGA-02-0037/1.3.6.1.4.1.14519.5.2.1.1706.4001.170054238455666525856624861567/1.3.6.1.4.1.14519.5.2.1.1706.4001.178667019197289195525849495660
TCGA-GBM	TCGA-02	TCGA-02-0037	t1Gd	F	74	109.769	AX_T1_POST	6	2.8195E+15	1/13/1998	GE MEDICAL SYSTEMS		GENESIS_SIGNA	SE	2D	5	583.332	8		6.39E+08	1.5	6.5	90	0.055159	23	256	192	0.781250\0.781250	TCGA-02-0037/1.3.6.1.4.1.14519.5.2.1.1706.4001.170054238455666525856624861567/1.3.6.1.4.1.14519.5.2.1.1706.4001.417797301325658887813420764479
TCGA-GBM	TCGA-02	TCGA-02-0037	t1	F	74	109.769	AX_T1	5	2.8195E+15	1/13/1998	GE MEDICAL SYSTEMS		GENESIS_SIGNA	SE	2D	5	416.664	8		6.39E+08	1.5	6.5	90	0.062985	23	256	128	0.781250\0.781250	TCGA-02-0037/1.3.6.1.4.1.14519.5.2.1.1706.4001.170054238455666525856624861567/1.3.6.1.4.1.14519.5.2.1.1706.4001.134108057579177395146913192259
TCGA-GBM	TCGA-02	TCGA-02-0037	t2	F	74	109.769	AX_T2_FSE	3	2.8195E+15	1/13/1998	GE MEDICAL SYSTEMS		GENESIS_SIGNA	SE	2D	5	3500	98		6.39E+08	1.5	6.5	90	0.042574	23	256	224	0.781250\0.781250	TCGA-02-0037/1.3.6.1.4.1.14519.5.2.1.1706.4001.170054238455666525856624861567/1.3.6.1.4.1.14519.5.2.1.1706.4001.242653383084895972538145369544
TCGA-GBM	TCGA-02	TCGA-02-0046	flair	M	61	86.183	AX_FLAIR	4	2.8195E+15	11/28/1998	GE MEDICAL SYSTEMS		SIGNA EXCITE	IR	2D	5	10002	141.54	2200	63.88013	1.5	6.5	90	0.0226	23	256	160	0.78125\0.78125	TCGA-02-0046/1.3.6.1.4.1.14519.5.2.1.1706.4001.667069087302553300405296434177/1.3.6.1.4.1.14519.5.2.1.1706.4001.509588674123932722082406945181
TCGA-GBM	TCGA-02	TCGA-02-0046	t1Gd	M	61	86.183	AX_T1_POST	6	2.8195E+15	11/28/1998	GE MEDICAL SYSTEMS		SIGNA EXCITE	SE	2D	5	583.332	8	0	63.88013	1.5	6.5	90	0.057336	23	256	192	0.78125\0.78125	TCGA-02-0046/1.3.6.1.4.1.14519.5.2.1.1706.4001.667069087302553300405296434177/1.3.6.1.4.1.14519.5.2.1.1706.4001.229600217589805337233000276642
TCGA-GBM	TCGA-02	TCGA-02-0046	t1	M	61	86.183	AX_T1	5	2.8195E+15	11/28/1998	GE MEDICAL SYSTEMS		SIGNA EXCITE	SE	2D	5	416.664	8	0	63.88013	1.5	6.5	90	0.06547	23	256	128	0.78125\0.78125	TCGA-02-0046/1.3.6.1.4.1.14519.5.2.1.1706.4001.667069087302553300405296434177/1.3.6.1.4.1.14519.5.2.1.1706.4001.266305079892504466765664273320
TCGA-GBM	TCGA-02	TCGA-02-0046	t2	M	61	86.183	AX_T2_FSE	3	2.8195E+15	11/28/1998	GE MEDICAL SYSTEMS		SIGNA EXCITE	SE	2D	5	3000	96.512	0	63.88013	1.5	6.5	90	0.086	23	256	224	0.78125\0.78125	TCGA-02-0046/1.3.6.1.4.1.14519.5.2.1.1706.4001.667069087302553300405296434177/1.3.6.1.4.1.14519.5.2.1.1706.4001.321860854252522153610965271371
TCGA-GBM	TCGA-02	TCGA-02-0047	flair	M	78	71.668	AX_FLAIR	4	2.8195E+15	12/15/1998	GE MEDICAL SYSTEMS		GENESIS_SIGNA	IR	2D	5	10002	147	2200	6.38E+08	1.5	6.5	90	0.023332	23	256	160	0.78125\0.78125	TCGA-02-0047/1.3.6.1.4.1.14519.5.2.1.1706.4001.246202953409591552042046870492/1.3.6.1.4.1.14519.5.2.1.1706.4001.123447841249545695967914613161
TCGA-GBM	TCGA-02	TCGA-02-0047	t1Gd	M	78	71.668	AX_T1_POST	6	2.8195E+15	12/15/1998	GE MEDICAL SYSTEMS		GENESIS_SIGNA	SE	2D	5	583.332	8		6.38E+08	1.5	6.5	90	0.059053	23	256	192	0.78125\0.78125	TCGA-02-0047/1.3.6.1.4.1.14519.5.2.1.1706.4001.246202953409591552042046870492/1.3.6.1.4.1.14519.5.2.1.1706.4001.245568253298410645742658166184
TCGA-GBM	TCGA-02	TCGA-02-0047	t1	M	78	71.668	AX_T1	5	2.8195E+15	12/15/1998	GE MEDICAL SYSTEMS		GENESIS_SIGNA	SE	2D	5	416.664	8		6.38E+08	1.5	6.5	90	0.067431	23	256	128	0.78125\0.78125	TCGA-02-0047/1.3.6.1.4.1.14519.5.2.1.1706.4001.246202953409591552042046870492/1.3.6.1.4.1.14519.5.2.1.1706.4001.238166248172920183561871538756
TCGA-GBM	TCGA-02	TCGA-02-0047	t2	M	78	71.668	AX_T2_FSE	3	2.8195E+15	12/15/1998	GE MEDICAL SYSTEMS		GENESIS_SIGNA	SE	2D	5	3500	98		6.38E+08	1.5	6.5	90	0.04558	23	256	224	0.78125\0.78125	TCGA-02-0047/1.3.6.1.4.1.14519.5.2.1.1706.4001.246202953409591552042046870492/1.3.6.1.4.1.14519.5.2.1.1706.4001.489474944906343317155982879920
TCGA-GBM	TCGA-02	TCGA-02-0048	flair	M	80	90.718	AX_FLAIR	4	2.8195E+15	1/29/1999	GE MEDICAL SYSTEMS		GENESIS_SIGNA	IR	2D	5	10002	147	2200	6.39E+08	1.5	6.5	90	0.022468	23	256	160	0.78125\0.78125	TCGA-02-0048/1.3.6.1.4.1.14519.5.2.1.1706.4001.271759313632030441078606502900/1.3.6.1.4.1.14519.5.2.1.1706.4001.226227114463442396869459184631
TCGA-GBM	TCGA-02	TCGA-02-0048	t1Gd	M	80	90.718	AX_T1_POST	6	2.8195E+15	1/29/1999	GE MEDICAL SYSTEMS		GENESIS_SIGNA	SE	2D	5	583.332	8		6.39E+08	1.5	6.5	90	0.056867	23	256	192	0.78125\0.78125	TCGA-02-0048/1.3.6.1.4.1.14519.5.2.1.1706.4001.271759313632030441078606502900/1.3.6.1.4.1.14519.5.2.1.1706.4001.208892423578919751306703554190
TCGA-GBM	TCGA-02	TCGA-02-0048	t1	M	80	90.718	AX_T1	5	2.8195E+15	1/29/1999	GE MEDICAL SYSTEMS		GENESIS_SIGNA	SE	2D	5	416.664	8		6.39E+08	1.5	6.5	90	0.064935	23	256	128	0.78125\0.78125	TCGA-02-0048/1.3.6.1.4.1.14519.5.2.1.1706.4001.271759313632030441078606502900/1.3.6.1.4.1.14519.5.2.1.1706.4001.232765711441628100940557835018
TCGA-GBM	TCGA-02	TCGA-02-0048	t2	M	80	90.718	AX_T2_FSE	3	2.8195E+15	1/29/1999	GE MEDICAL SYSTEMS		GENESIS_SIGNA	SE	2D	5	3500	98		6.39E+08	1.5	6.5	90	0.043893	23	256	224	0.78125\0.78125	TCGA-02-0048/1.3.6.1.4.1.14519.5.2.1.1706.4001.271759313632030441078606502900/1.3.6.1.4.1.14519.5.2.1.1706.4001.249496727867125014749061516941
TCGA-GBM	TCGA-02	TCGA-02-0054	flair	F	44	57.153	AX_FLAIR	5	2.8195E+15	5/16/1999	GE MEDICAL SYSTEMS		GENESIS_SIGNA	IR	2D	5	10002	147	2200	6.39E+08	1.5	6.5	90	0.024192	23	256	160	0.78125\0.78125	TCGA-02-0054/1.3.6.1.4.1.14519.5.2.1.1706.4001.151634660376289017402983968633/1.3.6.1.4.1.14519.5.2.1.1706.4001.285561797133963558047987589982
TCGA-GBM	TCGA-02	TCGA-02-0054	t1Gd	F	44	57.153	AX_T1_POST	9	2.8195E+15	5/16/1999	GE MEDICAL SYSTEMS		GENESIS_SIGNA	SE	2D	5	583.332	8		6.39E+08	1.5	6.5	90	0.061231	23	256	192	0.78125\0.78125	TCGA-02-0054/1.3.6.1.4.1.14519.5.2.1.1706.4001.151634660376289017402983968633/1.3.6.1.4.1.14519.5.2.1.1706.4001.276414996547218243402040733611
TCGA-GBM	TCGA-02	TCGA-02-0054	t1	F	44	57.153	AX_T1	6	2.8195E+15	5/16/1999	GE MEDICAL SYSTEMS		GENESIS_SIGNA	SE	2D	5	416.664	8		6.39E+08	1.5	6.5	90	0.069917	23	256	128	0.78125\0.78125	TCGA-02-0054/1.3.6.1.4.1.14519.5.2.1.1706.4001.151634660376289017402983968633/1.3.6.1.4.1.14519.5.2.1.1706.4001.281967755247405389888355967057
TCGA-GBM	TCGA-02	TCGA-02-0054	t2	F	44	57.153	AX_T2_FSE	4	2.8195E+15	5/16/1999	GE MEDICAL SYSTEMS		GENESIS_SIGNA	SE	2D	5	3500	98		6.39E+08	1.5	6.5	90	0.04726	23	256	224	0.78125\0.78125	TCGA-02-0054/1.3.6.1.4.1.14519.5.2.1.1706.4001.151634660376289017402983968633/1.3.6.1.4.1.14519.5.2.1.1706.4001.324132997720244415771309925529
TCGA-GBM	TCGA-02	TCGA-02-0059	flair	M	68	66.224	T2_FLAIR	12	2.8195E+15	8/29/1999	GE MEDICAL SYSTEMS		SIGNA EXCITE	IR	2D	5	10002	140.244	2250	127.7374	3	6.5	90	0.5816	23	320	224	0.46875\0.46875	TCGA-02-0059/1.3.6.1.4.1.14519.5.2.1.1706.4001.231755935407944517150217116610/1.3.6.1.4.1.14519.5.2.1.1706.4001.152913535526794168392822098633
TCGA-GBM	TCGA-02	TCGA-02-0059	t1Gd	M	68	66.224	Ax_SE_T1_Post	14	2.8195E+15	8/29/1999	GE MEDICAL SYSTEMS		SIGNA EXCITE	SE	2D	5	850	12	0	127.7374	3	6.5	90	1.705132	23	320	224	0.46875\0.46875	TCGA-02-0059/1.3.6.1.4.1.14519.5.2.1.1706.4001.231755935407944517150217116610/1.3.6.1.4.1.14519.5.2.1.1706.4001.389966548228828975637733047437
TCGA-GBM	TCGA-02	TCGA-02-0059	t1	M	68	66.224	FSPGR_3D	7	2.8195E+15	8/29/1999	GE MEDICAL SYSTEMS		SIGNA EXCITE	GR	3D	1.2	7.02	2.1	400	127.7374	3	1.2	20	0.369349	124	256	224	0.976562\0.976562	TCGA-02-0059/1.3.6.1.4.1.14519.5.2.1.1706.4001.231755935407944517150217116610/1.3.6.1.4.1.14519.5.2.1.1706.4001.104793971301967645892117754002
TCGA-GBM	TCGA-02	TCGA-02-0059	t2	M	68	66.224	FSE_T2	11	2.8195E+15	8/29/1999	GE MEDICAL SYSTEMS		SIGNA EXCITE	SE	2D	5	4000	99	0	127.7374	3	6.5	90	0.8331	23	320	256	0.46875\0.46875	TCGA-02-0059/1.3.6.1.4.1.14519.5.2.1.1706.4001.231755935407944517150217116610/1.3.6.1.4.1.14519.5.2.1.1706.4001.126408749477332517415027496534
TCGA-GBM	TCGA-02	TCGA-02-0060	flair	F	65	48.534	T2_FLAIR2MM25FOV	6	2.8195E+15	2/28/2000	GE MEDICAL SYSTEMS		SIGNA EXCITE	IR	2D	2	10002	140.778	2250	127.7412	3	2	90	0.6233	75	256	224	0.976562\0.976562	TCGA-02-0060/1.3.6.1.4.1.14519.5.2.1.1706.4001.247522006211308616726493960307/1.3.6.1.4.1.14519.5.2.1.1706.4001.875691179108572562890034722734
TCGA-GBM	TCGA-02	TCGA-02-0060	t1Gd	F	65	48.534	3D_AX_FSPGR_POST	14	2.8195E+15	2/28/2000	GE MEDICAL SYSTEMS		SIGNA EXCITE	GR	3D	1.4	4.944	2.1	400	127.7412	3	0.699999	20	0.880176	216	256	224	0.859375\0.859375	TCGA-02-0060/1.3.6.1.4.1.14519.5.2.1.1706.4001.247522006211308616726493960307/1.3.6.1.4.1.14519.5.2.1.1706.4001.203293857391455403037115181939
TCGA-GBM	TCGA-02	TCGA-02-0060	t1	F	65	48.534	FSPGR_3D_25FOV	5	2.8195E+15	2/28/2000	GE MEDICAL SYSTEMS		SIGNA EXCITE	GR	3D	1.2	6.644	2.1	400	127.7412	3	1.2	20	0.426227	124	256	224	0.976562\0.976562	TCGA-02-0060/1.3.6.1.4.1.14519.5.2.1.1706.4001.247522006211308616726493960307/1.3.6.1.4.1.14519.5.2.1.1706.4001.219609462264761522732587573237
TCGA-GBM	TCGA-02	TCGA-02-0060	t2	F	65	48.534	FSE_T2	8	2.8195E+15	2/28/2000	GE MEDICAL SYSTEMS		SIGNA EXCITE	SE	2D	5	5500	99.268	0	127.7412	3	6.5	90	1.155	24	320	224	0.429688\0.429688	TCGA-02-0060/1.3.6.1.4.1.14519.5.2.1.1706.4001.247522006211308616726493960307/1.3.6.1.4.1.14519.5.2.1.1706.4001.275488470620879346473604871467
TCGA-GBM	TCGA-02	TCGA-02-0064	flair	M	49	88.904	T2_FLAIR	12	2.8195E+15	8/8/1999	GE MEDICAL SYSTEMS		SIGNA EXCITE	IR	2D	5	10002	148.118	2250	127.7414	3	6.5	90	0.4916	23	320	224	0.46875\0.46875	TCGA-02-0064/1.3.6.1.4.1.14519.5.2.1.1706.4001.329011203940755039589411572116/1.3.6.1.4.1.14519.5.2.1.1706.4001.289547124248265878668833441740
TCGA-GBM	TCGA-02	TCGA-02-0064	t1Gd	M	49	88.904	FSPGR_3D	17	2.8195E+15	8/8/1999	GE MEDICAL SYSTEMS		SIGNA EXCITE	GR	3D	1.5	6.24	2.1	400	127.7413	3	1.5	20	0.697716	124	320	224	0.488281\0.488281	TCGA-02-0064/1.3.6.1.4.1.14519.5.2.1.1706.4001.329011203940755039589411572116/1.3.6.1.4.1.14519.5.2.1.1706.4001.120605412029452199210260324433
TCGA-GBM	TCGA-02	TCGA-02-0064	t1	M	49	88.904	FSPGR_3D	6	2.8195E+15	8/8/1999	GE MEDICAL SYSTEMS		SIGNA EXCITE	GR	3D	1.2	6.636	2.1	400	127.7414	3	1.2	20	0.468168	124	256	224	0.976562\0.976562	TCGA-02-0064/1.3.6.1.4.1.14519.5.2.1.1706.4001.329011203940755039589411572116/1.3.6.1.4.1.14519.5.2.1.1706.4001.908751550085828850580923426669
TCGA-GBM	TCGA-02	TCGA-02-0064	t2	M	49	88.904	FSE_T2	11	2.8195E+15	8/8/1999	GE MEDICAL SYSTEMS		SIGNA EXCITE	SE	2D	5	4000	98.076	0	127.7414	3	6.5	90	0.8718	23	320	256	0.46875\0.46875	TCGA-02-0064/1.3.6.1.4.1.14519.5.2.1.1706.4001.329011203940755039589411572116/1.3.6.1.4.1.14519.5.2.1.1706.4001.305032039524820143164998539810
TCGA-GBM	TCGA-02	TCGA-02-0068	flair	M	56	79.379	AX_FLAIR	4	2.8195E+15	5/16/2000	GE MEDICAL SYSTEMS		SIGNA EXCITE	IR	2D	5	10002	147.112	2200	63.8772	1.5	6.5	90	0.578	23	256	192	0.859375\0.859383	TCGA-02-0068/1.3.6.1.4.1.14519.5.2.1.1706.4001.239251012204750851180008114061/1.3.6.1.4.1.14519.5.2.1.1706.4001.169273732875851383844768405016
TCGA-GBM	TCGA-02	TCGA-02-0068	t1Gd	M	56	79.379	AX_T1_POST	9	2.8195E+15	5/16/2000	GE MEDICAL SYSTEMS		SIGNA EXCITE	SE	2D	5	550	9	0	63.8772	1.5	6.5	90	1.34066	23	256	192	0.859375\0.859383	TCGA-02-0068/1.3.6.1.4.1.14519.5.2.1.1706.4001.239251012204750851180008114061/1.3.6.1.4.1.14519.5.2.1.1706.4001.202828227420706130840978676909
TCGA-GBM	TCGA-02	TCGA-02-0068	t1	M	56	79.379	AX_T1	6	2.8195E+15	5/16/2000	GE MEDICAL SYSTEMS		SIGNA EXCITE	SE	2D	5	550	9	0	63.8772	1.5	6.5	90	1.34066	23	256	192	0.859375\0.859383	TCGA-02-0068/1.3.6.1.4.1.14519.5.2.1.1706.4001.239251012204750851180008114061/1.3.6.1.4.1.14519.5.2.1.1706.4001.217327932936754121521503964949
TCGA-GBM	TCGA-02	TCGA-02-0068	t2	M	56	79.379	AX_T2_FSE	3	2.8195E+15	5/16/2000	GE MEDICAL SYSTEMS		SIGNA EXCITE	SE	2D	5	3500	93.76	0	63.8772	1.5	6.5	90	0.6985	23	320	224	0.429688\0.429692	TCGA-02-0068/1.3.6.1.4.1.14519.5.2.1.1706.4001.239251012204750851180008114061/1.3.6.1.4.1.14519.5.2.1.1706.4001.164698594795106801737202685813
TCGA-GBM	TCGA-02	TCGA-02-0069	flair	F	31	65.771	T2_FLAIR	4	2.8195E+15	5/28/2000	GE MEDICAL SYSTEMS		SIGNA EXCITE	IR	2D	5	10002	140.778	2250	127.7411	3	6.5	90	0.4898	24	256	192	0.859375\0.859375	TCGA-02-0069/1.3.6.1.4.1.14519.5.2.1.1706.4001.380770062553415313882886843557/1.3.6.1.4.1.14519.5.2.1.1706.4001.476121618290067004679503813189
TCGA-GBM	TCGA-02	TCGA-02-0069	t1Gd	F	31	65.771	FSPGR_3D	12	2.8195E+15	5/28/2000	GE MEDICAL SYSTEMS		SIGNA EXCITE	GR	3D	1.5	6.116	2.1	400	127.7411	3	1.5	20	0.771899	124	320	224	0.488281\0.488281	TCGA-02-0069/1.3.6.1.4.1.14519.5.2.1.1706.4001.380770062553415313882886843557/1.3.6.1.4.1.14519.5.2.1.1706.4001.787868618042894085065532704205
TCGA-GBM	TCGA-02	TCGA-02-0069	t1	F	31	65.771	SE_T1	5	2.8195E+15	5/28/2000	GE MEDICAL SYSTEMS		SIGNA EXCITE	SE	2D	5	700	10	0	127.7411	3	6.5	90	2.15934	24	256	192	0.859375\0.859375	TCGA-02-0069/1.3.6.1.4.1.14519.5.2.1.1706.4001.380770062553415313882886843557/1.3.6.1.4.1.14519.5.2.1.1706.4001.249010498119891546309777700223
TCGA-GBM	TCGA-02	TCGA-02-0069	t2	F	31	65.771	FSE_T2	3	2.8195E+15	5/28/2000	GE MEDICAL SYSTEMS		SIGNA EXCITE	SE	2D	5	5500	99.268	0	127.7411	3	6.5	90	1.2104	24	320	224	0.429688\0.429688	TCGA-02-0069/1.3.6.1.4.1.14519.5.2.1.1706.4001.380770062553415313882886843557/1.3.6.1.4.1.14519.5.2.1.1706.4001.202260155195797715412373698690
TCGA-GBM	TCGA-02	TCGA-02-0070	flair	M	70	99.79	T2_FLAIR	4	2.8195E+15	7/10/2000	GE MEDICAL SYSTEMS		SIGNA EXCITE	IR	2D	5	10002	146.002	2250	127.7411	3	6.5	90	0.6703	24	256	192	0.859371\0.859375	TCGA-02-0070/1.3.6.1.4.1.14519.5.2.1.1706.4001.288983046313649223240718848290/1.3.6.1.4.1.14519.5.2.1.1706.4001.327171371276055674791400905248
TCGA-GBM	TCGA-02	TCGA-02-0070	t1Gd	M	70	99.79	AXIAL_T1_+C	10	2.8195E+15	7/10/2000	GE MEDICAL SYSTEMS		SIGNA EXCITE	SE	2D	5	700	10	0	127.7411	3	6.5	90	1.98231	24	256	192	0.859371\0.859375	TCGA-02-0070/1.3.6.1.4.1.14519.5.2.1.1706.4001.288983046313649223240718848290/1.3.6.1.4.1.14519.5.2.1.1706.4001.333477048103776358657330624985
TCGA-GBM	TCGA-02	TCGA-02-0070	t1	M	70	99.79	SE_T1	5	2.8195E+15	7/10/2000	GE MEDICAL SYSTEMS		SIGNA EXCITE	SE	2D	5	700	10	0	127.7411	3	6.5	90	1.7422	24	256	192	0.859371\0.859375	TCGA-02-0070/1.3.6.1.4.1.14519.5.2.1.1706.4001.288983046313649223240718848290/1.3.6.1.4.1.14519.5.2.1.1706.4001.223969978304636924462725206105
TCGA-GBM	TCGA-02	TCGA-02-0070	t2	M	70	99.79	FSE_T2	3	2.8195E+15	7/10/2000	GE MEDICAL SYSTEMS		SIGNA EXCITE	SE	2D	5	5500	99.268	0	127.7411	3	6.5	90	1.2906	24	320	224	0.429686\0.429688	TCGA-02-0070/1.3.6.1.4.1.14519.5.2.1.1706.4001.288983046313649223240718848290/1.3.6.1.4.1.14519.5.2.1.1706.4001.231105368610379750268505671981
TCGA-GBM	TCGA-02	TCGA-02-0075	flair	M	63	73.482	T2_FLAIR	11	2.8195E+15	9/24/1999	GE MEDICAL SYSTEMS		SIGNA EXCITE	IR	2D	5	10002	140.778	2250	127.7412	3	6.5	90	0.4983	23	256	192	0.859375\0.859375	TCGA-02-0075/1.3.6.1.4.1.14519.5.2.1.1706.4001.199028178384634677629049332875/1.3.6.1.4.1.14519.5.2.1.1706.4001.327311884902359009396424776096
TCGA-GBM	TCGA-02	TCGA-02-0075	t1Gd	M	63	73.482	Ax_SE_T1_Post	17	2.8195E+15	9/24/1999	GE MEDICAL SYSTEMS		SIGNA EXCITE	SE	2D	5	700	10	0	127.7412	3	6.5	90	2.09587	23	256	192	0.859375\0.859375	TCGA-02-0075/1.3.6.1.4.1.14519.5.2.1.1706.4001.199028178384634677629049332875/1.3.6.1.4.1.14519.5.2.1.1706.4001.269081075850064364799501008723
TCGA-GBM	TCGA-02	TCGA-02-0075	t1	M	63	73.482	FSPGR_3D	14	2.8195E+15	9/24/1999	GE MEDICAL SYSTEMS		SIGNA EXCITE	GR	3D	1.5	4.984	2.1	400	127.7412	3	1.5	20	0.786819	124	256	224	0.859375\0.859375	TCGA-02-0075/1.3.6.1.4.1.14519.5.2.1.1706.4001.199028178384634677629049332875/1.3.6.1.4.1.14519.5.2.1.1706.4001.149622591468669601675018790164
TCGA-GBM	TCGA-02	TCGA-02-0075	t2	M	63	73.482	FSE_T2	10	2.8195E+15	9/24/1999	GE MEDICAL SYSTEMS		SIGNA EXCITE	SE	2D	5	5500	102.596	0	127.7412	3	6.5	90	1.1799	23	256	192	0.859375\0.859375	TCGA-02-0075/1.3.6.1.4.1.14519.5.2.1.1706.4001.199028178384634677629049332875/1.3.6.1.4.1.14519.5.2.1.1706.4001.317391215776965396207339135736
TCGA-GBM	TCGA-02	TCGA-02-0085	flair	F	65	90.265	AX_FLAIR	4	2.8195E+15	1/30/1999	GE MEDICAL SYSTEMS		GENESIS_SIGNA	IR	2D	5	10002	147	2200	6.39E+08	1.5	6.5	90	0.022486	22	256	160	0.78125\0.78125	TCGA-02-0085/1.3.6.1.4.1.14519.5.2.1.1706.4001.289458647018511940499719529853/1.3.6.1.4.1.14519.5.2.1.1706.4001.108905473702454736524787314351
TCGA-GBM	TCGA-02	TCGA-02-0085	t1Gd	F	65	90.265	AX_T1_POST	6	2.8195E+15	1/30/1999	GE MEDICAL SYSTEMS		GENESIS_SIGNA	SE	2D	5	583.332	8		6.39E+08	1.5	6.5	90	0.054438	22	256	192	0.78125\0.78125	TCGA-02-0085/1.3.6.1.4.1.14519.5.2.1.1706.4001.289458647018511940499719529853/1.3.6.1.4.1.14519.5.2.1.1706.4001.179485994007439679254001323335
TCGA-GBM	TCGA-02	TCGA-02-0085	t1	F	65	90.265	AX_T1	5	2.8195E+15	1/30/1999	GE MEDICAL SYSTEMS		GENESIS_SIGNA	SE	2D	5	416.664	8		6.39E+08	1.5	6.5	90	0.06216	22	256	128	0.78125\0.78125	TCGA-02-0085/1.3.6.1.4.1.14519.5.2.1.1706.4001.289458647018511940499719529853/1.3.6.1.4.1.14519.5.2.1.1706.4001.288895680883982235784348384008
TCGA-GBM	TCGA-02	TCGA-02-0085	t2	F	65	90.265	AX_T2_FSE	3	2.8195E+15	1/30/1999	GE MEDICAL SYSTEMS		GENESIS_SIGNA	SE	2D	5	3500	98		6.39E+08	1.5	6.5	90	0.042018	22	256	224	0.78125\0.78125	TCGA-02-0085/1.3.6.1.4.1.14519.5.2.1.1706.4001.289458647018511940499719529853/1.3.6.1.4.1.14519.5.2.1.1706.4001.278352104503675092135094187118
TCGA-GBM	TCGA-02	TCGA-02-0086	flair	F	45	96.162	AX_FLAIR	4	2.8195E+15	7/25/1999	GE MEDICAL SYSTEMS		SIGNA EXCITE	IR	2D	5	10002	141.54	2200	63.87812	1.5	6.5	90	0.0222	23	256	160	0.78125\0.78125	TCGA-02-0086/1.3.6.1.4.1.14519.5.2.1.1706.4001.189302178457617024065570049848/1.3.6.1.4.1.14519.5.2.1.1706.4001.223838339019946872254464308895
TCGA-GBM	TCGA-02	TCGA-02-0086	t1Gd	F	45	96.162	AX_T1_POST	11	2.8195E+15	7/25/1999	GE MEDICAL SYSTEMS		SIGNA EXCITE	SE	2D	5	416.664	8	0	63.87817	1.5	6.5	90	0.064333	23	256	192	0.78125\0.78125	TCGA-02-0086/1.3.6.1.4.1.14519.5.2.1.1706.4001.189302178457617024065570049848/1.3.6.1.4.1.14519.5.2.1.1706.4001.327450908011286130960738686265
TCGA-GBM	TCGA-02	TCGA-02-0086	t1	F	45	96.162	AX_T1	6	2.8195E+15	7/25/1999	GE MEDICAL SYSTEMS		SIGNA EXCITE	SE	2D	5	416.664	8	0	63.87812	1.5	6.5	90	0.064333	23	256	128	0.78125\0.78125	TCGA-02-0086/1.3.6.1.4.1.14519.5.2.1.1706.4001.189302178457617024065570049848/1.3.6.1.4.1.14519.5.2.1.1706.4001.300182168244910270765145894367
TCGA-GBM	TCGA-02	TCGA-02-0086	t2	F	45	96.162	AX_T2_FSE	3	2.8195E+15	7/25/1999	GE MEDICAL SYSTEMS		SIGNA EXCITE	SE	2D	5	3500	96.512	0	63.87812	1.5	6.5	90	0.0724	23	256	224	0.78125\0.78125	TCGA-02-0086/1.3.6.1.4.1.14519.5.2.1.1706.4001.189302178457617024065570049848/1.3.6.1.4.1.14519.5.2.1.1706.4001.130793598392145152904761943139
TCGA-GBM	TCGA-02	TCGA-02-0087	flair	F	27	81.647	T2_FLAIR	10	2.8195E+15	12/13/1999	GE MEDICAL SYSTEMS		SIGNA EXCITE	IR	2D	5	10002	140.778	2250	127.7412	3	6.5	90	0.5064	23	256	192	0.859375\0.859375	TCGA-02-0087/1.3.6.1.4.1.14519.5.2.1.1706.4001.197977742428665924369847662702/1.3.6.1.4.1.14519.5.2.1.1706.4001.188891971026185310471464105645
TCGA-GBM	TCGA-02	TCGA-02-0087	t1Gd	F	27	81.647	FSPGR_3D	18	2.8195E+15	12/13/1999	GE MEDICAL SYSTEMS		SIGNA EXCITE	GR	3D	1.5	6.176	2.1	400	127.7411	3	1.5	20	0.741433	124	320	224	0.488281\0.488281	TCGA-02-0087/1.3.6.1.4.1.14519.5.2.1.1706.4001.197977742428665924369847662702/1.3.6.1.4.1.14519.5.2.1.1706.4001.130023584613816858008662430540
TCGA-GBM	TCGA-02	TCGA-02-0087	t1	F	27	81.647	FSPGR_3D_25FOV	5	2.8195E+15	12/13/1999	GE MEDICAL SYSTEMS		SIGNA EXCITE	GR	3D	1.2	6.644	2.1	400	127.7412	3	1.2	20	0.461773	124	256	224	0.976562\0.976562	TCGA-02-0087/1.3.6.1.4.1.14519.5.2.1.1706.4001.197977742428665924369847662702/1.3.6.1.4.1.14519.5.2.1.1706.4001.153352528048877621623084394547
TCGA-GBM	TCGA-02	TCGA-02-0087	t2	F	27	81.647	FSE_T2	9	2.8195E+15	12/13/1999	GE MEDICAL SYSTEMS		SIGNA EXCITE	SE	2D	5	5500	99.268	0	127.7412	3	6.5	90	1.1992	23	320	224	0.429688\0.429688	TCGA-02-0087/1.3.6.1.4.1.14519.5.2.1.1706.4001.197977742428665924369847662702/1.3.6.1.4.1.14519.5.2.1.1706.4001.297280847905859204769837917360
TCGA-GBM	TCGA-02	TCGA-02-0102	flair	M	42	74.843	AX_FLAIR	4	2.8195E+15	12/15/1997	GE MEDICAL SYSTEMS		GENESIS_SIGNA	IR	2D	5	10002	147	2200	6.39E+08	1.5	6.5	90	0.023171	23	256	160	0.78125\0.78125	TCGA-02-0102/1.3.6.1.4.1.14519.5.2.1.1706.4001.240465490805420927209667546432/1.3.6.1.4.1.14519.5.2.1.1706.4001.580630653491671454692169960256
TCGA-GBM	TCGA-02	TCGA-02-0102	t1Gd	M	42	74.843	AX_T1_POST	6	2.8195E+15	12/15/1997	GE MEDICAL SYSTEMS		GENESIS_SIGNA	SE	2D	5	583.332	9		6.39E+08	1.5	6.5	90	0.058645	23	256	192	0.78125\0.78125	TCGA-02-0102/1.3.6.1.4.1.14519.5.2.1.1706.4001.240465490805420927209667546432/1.3.6.1.4.1.14519.5.2.1.1706.4001.958674936724987421213786894928
TCGA-GBM	TCGA-02	TCGA-02-0102	t1	M	42	74.843	AX_T1	5	2.8195E+15	12/15/1997	GE MEDICAL SYSTEMS		GENESIS_SIGNA	SE	2D	5	416.664	8		6.39E+08	1.5	6.5	90	0.066965	23	256	128	0.78125\0.78125	TCGA-02-0102/1.3.6.1.4.1.14519.5.2.1.1706.4001.240465490805420927209667546432/1.3.6.1.4.1.14519.5.2.1.1706.4001.321931575003689210541138128522
TCGA-GBM	TCGA-02	TCGA-02-0102	t2	M	42	74.843	AX_T2_FSE	3	2.8195E+15	12/15/1997	GE MEDICAL SYSTEMS		GENESIS_SIGNA	SE	2D	5	3500	105		6.39E+08	1.5	6.5	90	0.045265	23	256	224	0.78125\0.78125	TCGA-02-0102/1.3.6.1.4.1.14519.5.2.1.1706.4001.240465490805420927209667546432/1.3.6.1.4.1.14519.5.2.1.1706.4001.846686946556146675536736249954
TCGA-GBM	TCGA-02	TCGA-02-0106	flair	M	54	70.307	AX_FLAIR	4	2.8195E+15	10/30/1998	GE MEDICAL SYSTEMS		GENESIS_SIGNA	IR	2D	5	10002	147	2200	6.38E+08	1.5	6.5	90	0.023404	23	256	160	0.78125\0.78125	TCGA-02-0106/1.3.6.1.4.1.14519.5.2.1.1706.4001.204859028463062009490599344146/1.3.6.1.4.1.14519.5.2.1.1706.4001.142741494614831385536214775922
TCGA-GBM	TCGA-02	TCGA-02-0106	t1Gd	M	54	70.307	AX_T1_POST	6	2.8195E+15	10/30/1998	GE MEDICAL SYSTEMS		GENESIS_SIGNA	SE	2D	5	583.332	8		6.38E+08	1.5	6.5	90	0.059235	23	256	192	0.78125\0.78125	TCGA-02-0106/1.3.6.1.4.1.14519.5.2.1.1706.4001.204859028463062009490599344146/1.3.6.1.4.1.14519.5.2.1.1706.4001.407761314964431500733064308177
TCGA-GBM	TCGA-02	TCGA-02-0106	t1	M	54	70.307	AX_T1	5	2.8195E+15	10/30/1998	GE MEDICAL SYSTEMS		GENESIS_SIGNA	SE	2D	5	416.664	8		6.38E+08	1.5	6.5	90	0.067638	23	256	128	0.78125\0.78125	TCGA-02-0106/1.3.6.1.4.1.14519.5.2.1.1706.4001.204859028463062009490599344146/1.3.6.1.4.1.14519.5.2.1.1706.4001.257637810777885113989358890547
TCGA-GBM	TCGA-02	TCGA-02-0106	t2	M	54	70.307	AX_T2_FSE	3	2.8195E+15	10/30/1998	GE MEDICAL SYSTEMS		GENESIS_SIGNA	SE	2D	5	3500	98		6.38E+08	1.5	6.5	90	0.04572	23	256	224	0.78125\0.78125	TCGA-02-0106/1.3.6.1.4.1.14519.5.2.1.1706.4001.204859028463062009490599344146/1.3.6.1.4.1.14519.5.2.1.1706.4001.189172039998441866861864803373
TCGA-GBM	TCGA-02	TCGA-02-0116	flair	M	51	99.79	AX_FLAIR	4	2.8195E+15	3/22/1997	GE MEDICAL SYSTEMS		GENESIS_SIGNA	IR	2D	5	10002	147	2200	6.39E+08	1.5	6.5	90	0.022128	24	256	160	0.781250\0.781250	TCGA-02-0116/1.3.6.1.4.1.14519.5.2.1.1706.4001.333801404495028917226888170515/1.3.6.1.4.1.14519.5.2.1.1706.4001.511107585415671993882365147439
TCGA-GBM	TCGA-02	TCGA-02-0116	t1Gd	M	51	99.79	AX_T1_POST	6	2.8195E+15	3/22/1997	GE MEDICAL SYSTEMS		GENESIS_SIGNA	SE	2D	5	633.332	8		6.39E+08	1.5	6.5	90	0.053814	24	256	192	0.781250\0.781250	TCGA-02-0116/1.3.6.1.4.1.14519.5.2.1.1706.4001.333801404495028917226888170515/1.3.6.1.4.1.14519.5.2.1.1706.4001.324271664399244452369200352648
TCGA-GBM	TCGA-02	TCGA-02-0116	t1	M	51	99.79	AX_T1	5	2.8195E+15	3/22/1997	GE MEDICAL SYSTEMS		GENESIS_SIGNA	SE	2D	5	433.332	8		6.39E+08	1.5	6.5	90	0.064152	24	256	128	0.781250\0.781250	TCGA-02-0116/1.3.6.1.4.1.14519.5.2.1.1706.4001.333801404495028917226888170515/1.3.6.1.4.1.14519.5.2.1.1706.4001.119332675140408493379026119335
TCGA-GBM	TCGA-02	TCGA-02-0116	t2	M	51	99.79	AX_T2_FSE	3	2.8195E+15	3/22/1997	GE MEDICAL SYSTEMS		GENESIS_SIGNA	SE	2D	5	3500	98		6.39E+08	1.5	6.5	90	0.045108	24	256	224	0.781250\0.781250	TCGA-02-0116/1.3.6.1.4.1.14519.5.2.1.1706.4001.333801404495028917226888170515/1.3.6.1.4.1.14519.5.2.1.1706.4001.181707635044752935161529942524
TCGA-GBM	TCGA-06	TCGA-06-0119	flair	F			AxFLAIR-thin_for_surgery	7	2.27963E+15	12/26/2003		BAY2OC0		IR	2D	2.5	10004	155	2200	6.38E+08	1.5	2.5	90	0.035398	71	256	192	0.9375\0.9375	TCGA-06-0119/1.3.6.1.4.1.14519.5.2.1.4591.4001.283171458436570880779969690543/1.3.6.1.4.1.14519.5.2.1.4591.4001.190846624143899319203062806923
TCGA-GBM	TCGA-06	TCGA-06-0119	t1Gd	F			=+COR_T1	12	2.27963E+15	12/26/2003		BAY2OC0		SE	2D	5	500	14		6.38E+08	1.5	5	90	0.040717	37	256	192	0.9375\0.9375	TCGA-06-0119/1.3.6.1.4.1.14519.5.2.1.4591.4001.283171458436570880779969690543/1.3.6.1.4.1.14519.5.2.1.4591.4001.130997813040669753794699573293
TCGA-GBM	TCGA-06	TCGA-06-0119	t1	F			AXIAL_T1	6	2.27963E+15	12/26/2003		BAY2OC0		SE	2D	5	500	14		6.38E+08	1.5	5	90	0.037584	36	256	192	0.9375\0.9375	TCGA-06-0119/1.3.6.1.4.1.14519.5.2.1.4591.4001.283171458436570880779969690543/1.3.6.1.4.1.14519.5.2.1.4591.4001.175712352031583034594446384562
TCGA-GBM	TCGA-06	TCGA-06-0119	t2	F			AXIAL_FSE	5	2.27963E+15	12/26/2003		BAY2OC0		SE	2D	5	3500	20		6.38E+08	1.5	5	90	0.039045	72	256	192	0.9375\0.9375	TCGA-06-0119/1.3.6.1.4.1.14519.5.2.1.4591.4001.283171458436570880779969690543/1.3.6.1.4.1.14519.5.2.1.4591.4001.970644432299498858379456667200
TCGA-GBM	TCGA-06	TCGA-06-0122	flair	F			AXIAL_FLAIR	5	1.89289E+15	9/14/2004		MR3T		IR	2D	2.5	10002	125.4	2250	127.7323	3	2.5	90	0.6054	72	320	224	0.4688\0.4688	TCGA-06-0122/1.3.6.1.4.1.14519.5.2.1.4591.4001.545613483524570609928056355494/1.3.6.1.4.1.14519.5.2.1.4591.4001.128296557660865063844963462837
TCGA-GBM	TCGA-06	TCGA-06-0122	t1Gd	F			AX_T1_POST_GD_FLAIR	10	1.89289E+15	9/14/2004		MR3T		IR	2D	2.5	3181.81	6.356	1238	127.7322	3	2.5	90	1.4511	72	320	224	0.4688\0.4688	TCGA-06-0122/1.3.6.1.4.1.14519.5.2.1.4591.4001.545613483524570609928056355494/1.3.6.1.4.1.14519.5.2.1.4591.4001.151947201227980965067378635297
TCGA-GBM	TCGA-06	TCGA-06-0122	t1	F			AX_T1_pre_gd	8	1.89289E+15	9/14/2004		MR3T		IR	2D	5	3000.01	6.356	1238	127.7323	3	5	90	1.1543	36	320	224	0.4688\0.4688	TCGA-06-0122/1.3.6.1.4.1.14519.5.2.1.4591.4001.545613483524570609928056355494/1.3.6.1.4.1.14519.5.2.1.4591.4001.976676789917195046100044055071
TCGA-GBM	TCGA-06	TCGA-06-0122	t2	F			AX_T2_FR-FSE_RF2_150	6	1.89289E+15	9/14/2004		MR3T		SE	2D	5	3000	103.464	0	127.7323	3	5	90	1.3382	36	320	224	0.4688\0.4688	TCGA-06-0122/1.3.6.1.4.1.14519.5.2.1.4591.4001.545613483524570609928056355494/1.3.6.1.4.1.14519.5.2.1.4591.4001.284098509532271800980320718400
TCGA-GBM	TCGA-06	TCGA-06-0128	flair	M		77	AXIAL_FLAIR	3	2.38081E+15	2/18/1999	General Electric	BAY	GENESIS	flair		3	10004	155	2200			3	90		53			0.938\0.938	TCGA-06-0128/1.3.6.1.4.1.14519.5.2.1.4591.4001.197146947506099366832803736438/1.3.6.1.4.1.14519.5.2.1.4591.4001.651359394442711367070939266432
TCGA-GBM	TCGA-06	TCGA-06-0128	t1Gd	M		77	AXIAL_T1_POST_GD	9	2.38081E+15	2/18/1999	General Electric	BAY	GENESIS	memp		3	500	14	0			3	90		53			0.938\0.938	TCGA-06-0128/1.3.6.1.4.1.14519.5.2.1.4591.4001.197146947506099366832803736438/1.3.6.1.4.1.14519.5.2.1.4591.4001.155987932372490417929540843368
TCGA-GBM	TCGA-06	TCGA-06-0128	t1	M		77	AXIAL_T1_PRE_GD	7	2.38081E+15	2/18/1999	General Electric	BAY	GENESIS	memp		3	500	14	0			3	90		53			0.938\0.938	TCGA-06-0128/1.3.6.1.4.1.14519.5.2.1.4591.4001.197146947506099366832803736438/1.3.6.1.4.1.14519.5.2.1.4591.4001.619151632501848799260529175220
TCGA-GBM	TCGA-06	TCGA-06-0128	t2	M		77	AXIAL__FSE	2	2.38081E+15	2/18/1999	General Electric	BAY	GENESIS	fse		3	3500	22	0			3	90		106			0.938\0.938	TCGA-06-0128/1.3.6.1.4.1.14519.5.2.1.4591.4001.197146947506099366832803736438/1.3.6.1.4.1.14519.5.2.1.4591.4001.120673894695124200101185494671
TCGA-GBM	TCGA-06	TCGA-06-0130	flair	M			AXIAL_FLAIR_RF2__150	7	2.70782E+15	9/11/2001		MR3T		IR	2D	2.5	10002	123.5	2250	1.28E+09	3	2.5	90	0.090741	78	256	192	0.937500\0.937500	TCGA-06-0130/1.3.6.1.4.1.14519.5.2.1.4591.4001.123247425694007311600825493062/1.3.6.1.4.1.14519.5.2.1.4591.4001.296118904200952959225417317679
TCGA-GBM	TCGA-06	TCGA-06-0130	t1Gd	M			AX_T1_POST_GD_FLAIR	11	2.70782E+15	9/11/2001		MR3T		RM	2D	2.5	3252.816	8.104	860	1.28E+09	3	2.5	90	0.100896	80	288	192	0.468750\0.468750	TCGA-06-0130/1.3.6.1.4.1.14519.5.2.1.4591.4001.123247425694007311600825493062/1.3.6.1.4.1.14519.5.2.1.4591.4001.128853861285611731507706018829
TCGA-GBM	TCGA-06	TCGA-06-0130	t1	M			AX_T1_pre_gd	8	2.70782E+15	9/11/2001		MR3T		RM	2D	5	2366.264	8.104	860	1.28E+09	3	5	90	0.107373	42	288	192	0.468750\0.468750	TCGA-06-0130/1.3.6.1.4.1.14519.5.2.1.4591.4001.123247425694007311600825493062/1.3.6.1.4.1.14519.5.2.1.4591.4001.838991966145071683848655126039
TCGA-GBM	TCGA-06	TCGA-06-0130	t2	M			AX_T2_FR-FSE_RF2_150	6	2.70782E+15	9/11/2001		MR3T		RM	2D	5	3766.664	104.972		1.28E+09	3	5	90	0.0939	40	352	192	0.468750\0.468750	TCGA-06-0130/1.3.6.1.4.1.14519.5.2.1.4591.4001.123247425694007311600825493062/1.3.6.1.4.1.14519.5.2.1.4591.4001.305701459061918935894190837157
TCGA-GBM	TCGA-06	TCGA-06-0132	flair	M			AXIAL_FLAIR	5	2.64669E+15	5/17/2005		BAY2OC0		IR	2D	2.5	10004	155	2200	6.39E+08	1.5	2.5	90	0.031318	71	256	192	0.9375\0.9375	TCGA-06-0132/1.3.6.1.4.1.14519.5.2.1.4591.4001.156068272915163991923280385588/1.3.6.1.4.1.14519.5.2.1.4591.4001.288861537619222119588401224045
TCGA-GBM	TCGA-06	TCGA-06-0132	t1Gd	M			AXIAL_T1_GD	15	2.64669E+15	5/17/2005		BAY2OC0		SE	2D	2.5	500	13		6.39E+08	1.5	2.5	90	0.044335	76	256	192	0.9375\0.9375	TCGA-06-0132/1.3.6.1.4.1.14519.5.2.1.4591.4001.156068272915163991923280385588/1.3.6.1.4.1.14519.5.2.1.4591.4001.662318710003756037574079830102
TCGA-GBM	TCGA-06	TCGA-06-0132	t1	M			AXIAL_T1	7	2.64669E+15	5/17/2005		BAY2OC0		SE	2D	5	500	14		6.39E+08	1.5	5	90	0.036023	37	256	192	0.9375\0.9375	TCGA-06-0132/1.3.6.1.4.1.14519.5.2.1.4591.4001.156068272915163991923280385588/1.3.6.1.4.1.14519.5.2.1.4591.4001.249844598065449004919997881569
TCGA-GBM	TCGA-06	TCGA-06-0132	t2	M			AXIAL_FSE	4	2.64669E+15	5/17/2005		BAY2OC0		SE	2D	5	3500	20		6.39E+08	1.5	5	90	0.036463	74	256	192	0.9375\0.9375	TCGA-06-0132/1.3.6.1.4.1.14519.5.2.1.4591.4001.156068272915163991923280385588/1.3.6.1.4.1.14519.5.2.1.4591.4001.148730121880670249624667054880
TCGA-GBM	TCGA-06	TCGA-06-0133	flair	M			AXIAL_FLAIR	5	1.48959E+15	5/10/2005		MR3T		IR	2D	2.5	10002	125.4	2250	127.7322	3	2.5	90	1.0485	71	320	224	0.4688\0.4688	TCGA-06-0133/1.3.6.1.4.1.14519.5.2.1.4591.4001.200432657521696433544702327867/1.3.6.1.4.1.14519.5.2.1.4591.4001.915421561452489655072317720028
TCGA-GBM	TCGA-06	TCGA-06-0133	t1Gd	M			AX_T1_POST_GD_FLAIR	11	1.48959E+15	5/10/2005		MR3T		IR	2D	2.5	3072.51	6.356	1238	127.7322	3	2.5	90	1.5929	71	320	224	0.4688\0.4688	TCGA-06-0133/1.3.6.1.4.1.14519.5.2.1.4591.4001.200432657521696433544702327867/1.3.6.1.4.1.14519.5.2.1.4591.4001.312563117951495282981965119560
TCGA-GBM	TCGA-06	TCGA-06-0133	t1	M			AX_T1_pre_gd	8	1.48959E+15	5/10/2005		MR3T		IR	2D	5	3072.58	6.356	1238	127.7323	3	5	90	1.5929	36	320	224	0.4688\0.4688	TCGA-06-0133/1.3.6.1.4.1.14519.5.2.1.4591.4001.200432657521696433544702327867/1.3.6.1.4.1.14519.5.2.1.4591.4001.188613876158978626356924469119
TCGA-GBM	TCGA-06	TCGA-06-0133	t2	M			AX_T2_FR-FSE_RF2_150	6	1.48959E+15	5/10/2005		MR3T		SE	2D	5	3000	103.464	0	127.7322	3	5	90	1.4532	36	320	224	0.4688\0.4688	TCGA-06-0133/1.3.6.1.4.1.14519.5.2.1.4591.4001.200432657521696433544702327867/1.3.6.1.4.1.14519.5.2.1.4591.4001.165459028026483730012969806104
TCGA-GBM	TCGA-06	TCGA-06-0137	flair	F			AXIAL_FLAIR	4	1.55442E+15	12/24/2001		BAY2OC0		IR	2D	3	10004	155	2200	6.38E+08	1.5	3	90	0.028464	50	256	192	0.9375\0.9375	TCGA-06-0137/1.3.6.1.4.1.14519.5.2.1.4591.4001.326662079066250663678557696078/1.3.6.1.4.1.14519.5.2.1.4591.4001.109697696341289807648805483606
TCGA-GBM	TCGA-06	TCGA-06-0137	t1Gd	F			AXIAL_T1_GD	9	1.55442E+15	12/24/2001		BAY2OC0		SE	2D	3	500	14		6.38E+08	1.5	3	90	0.039289	50	256	192	0.9375\0.9375	TCGA-06-0137/1.3.6.1.4.1.14519.5.2.1.4591.4001.326662079066250663678557696078/1.3.6.1.4.1.14519.5.2.1.4591.4001.202223923499982894907965771887
TCGA-GBM	TCGA-06	TCGA-06-0137	t1	F			AXIAL_T1	6	1.55442E+15	12/24/2001		BAY2OC0		SE	2D	3	500	14		6.38E+08	1.5	3	90	0.039289	50	256	192	0.9375\0.9375	TCGA-06-0137/1.3.6.1.4.1.14519.5.2.1.4591.4001.326662079066250663678557696078/1.3.6.1.4.1.14519.5.2.1.4591.4001.305227854284090555965781589764
TCGA-GBM	TCGA-06	TCGA-06-0137	t2	F			AXIAL_FSE	3	1.55442E+15	12/24/2001		BAY2OC0		SE	2D	3	3500	20		6.38E+08	1.5	3	90	0.052326	100	256	192	0.9375\0.9375	TCGA-06-0137/1.3.6.1.4.1.14519.5.2.1.4591.4001.326662079066250663678557696078/1.3.6.1.4.1.14519.5.2.1.4591.4001.157809556490045867232259259088
TCGA-GBM	TCGA-06	TCGA-06-0138	flair	M			AXIAL_T2_FLAIR	6	5.979E+15	11/25/2002		MR3T		IR	2D	2.5	10002	124.9	2250	127.7324	3	2.5	90	1.0139	72	320	224	0.46875\0.46875	TCGA-06-0138/1.3.6.1.4.1.14519.5.2.1.4591.4001.296601565633699364447385491840/1.3.6.1.4.1.14519.5.2.1.4591.4001.312547018558825125677081232576
TCGA-GBM	TCGA-06	TCGA-06-0138	t1Gd	M			AX_T1_POST_GD_FLAIR	10	5.979E+15	11/25/2002		MR3T		IR	2D	2.5	3285.62	6.568	1238	127.7324	3	2.5	90	1.5327	69	320	224	0.46875\0.46875	TCGA-06-0138/1.3.6.1.4.1.14519.5.2.1.4591.4001.296601565633699364447385491840/1.3.6.1.4.1.14519.5.2.1.4591.4001.973243150706573836060823787338
TCGA-GBM	TCGA-06	TCGA-06-0138	t1	M			AX_T1_pre_gd	8	5.979E+15	11/25/2002		MR3T		IR	2D	5	3379.57	6.584	1238	127.7324	3	5	90	1.2772	36	320	224	0.46875\0.46875	TCGA-06-0138/1.3.6.1.4.1.14519.5.2.1.4591.4001.296601565633699364447385491840/1.3.6.1.4.1.14519.5.2.1.4591.4001.208761379736355493761543330080
TCGA-GBM	TCGA-06	TCGA-06-0138	t2	M			AX_T2_FR-FSE_RF2_150	5	5.979E+15	11/25/2002		MR3T		SE	2D	5	3000	104.436	0	127.7324	3	5	90	1.3877	36	320	224	0.46875\0.46875	TCGA-06-0138/1.3.6.1.4.1.14519.5.2.1.4591.4001.296601565633699364447385491840/1.3.6.1.4.1.14519.5.2.1.4591.4001.251176902091719343107685682166
TCGA-GBM	TCGA-06	TCGA-06-0139	flair	M			AXIAL_FLAIR	5	3.34682E+15	6/10/2004		MR3T		IR	2D	2.5	10002	125.4	2250	127.7323	3	2.5	90	0.8274	71	320	224	0.46875\0.46875	TCGA-06-0139/1.3.6.1.4.1.14519.5.2.1.4591.4001.751927611731877561558954106150/1.3.6.1.4.1.14519.5.2.1.4591.4001.191924528389804940209560191592
TCGA-GBM	TCGA-06	TCGA-06-0139	t1Gd	M			AX_T1_POST_GD_FLAIR	10	3.34682E+15	6/10/2004		MR3T		IR	2D	2.5	3062.5	6.356	1238	127.7323	3	2.5	90	1.5454	71	320	224	0.46875\0.46875	TCGA-06-0139/1.3.6.1.4.1.14519.5.2.1.4591.4001.751927611731877561558954106150/1.3.6.1.4.1.14519.5.2.1.4591.4001.229090327759241505815737823916
TCGA-GBM	TCGA-06	TCGA-06-0139	t1	M			AX_T1_pre_gd	8	3.34682E+15	6/10/2004		MR3T		IR	2D	5	3062.58	6.356	1238	127.7323	3	5	90	1.5453	36	320	224	0.46875\0.46875	TCGA-06-0139/1.3.6.1.4.1.14519.5.2.1.4591.4001.751927611731877561558954106150/1.3.6.1.4.1.14519.5.2.1.4591.4001.332362461346917707181060064705
TCGA-GBM	TCGA-06	TCGA-06-0139	t2	M			AX_T2_FR-FSE_RF2_150	6	3.34682E+15	6/10/2004		MR3T		SE	2D	5	3000	103.464	0	127.7323	3	5	90	1.8289	36	320	224	0.46875\0.46875	TCGA-06-0139/1.3.6.1.4.1.14519.5.2.1.4591.4001.751927611731877561558954106150/1.3.6.1.4.1.14519.5.2.1.4591.4001.209789345577718778695745727517
TCGA-GBM	TCGA-06	TCGA-06-0142	flair	M			AXIAL_FLAIR	7	2.8195E+15	3/11/2000		BAY2BAY2	IR	2D	3	10004	155	2200	6.38E+08	1.5	3	90	0.991425	56	256	192	0.937500\0.937500	TCGA-06-0142/1.3.6.1.4.1.14519.5.2.1.4591.4001.140537559994289172851244415813/1.3.6.1.4.1.14519.5.2.1.4591.4001.265250330319551296448339505489	
TCGA-GBM	TCGA-06	TCGA-06-0142	t1Gd	M			AXIAL_T1_GD	11	2.8195E+15	3/11/2000		BAY2BAY2	SE	2D	3	500	14		6.38E+08	1.5	3	90	1.228103	56	256	192	0.937500\0.937500	TCGA-06-0142/1.3.6.1.4.1.14519.5.2.1.4591.4001.140537559994289172851244415813/1.3.6.1.4.1.14519.5.2.1.4591.4001.278231207410707039093733128546	
TCGA-GBM	TCGA-06	TCGA-06-0142	t1	M			AXIAL_T1	9	2.8195E+15	3/11/2000		BAY2BAY2	SE	2D	3	500	14		6.38E+08	1.5	3	90	1.228103	56	256	192	0.937500\0.937500	TCGA-06-0142/1.3.6.1.4.1.14519.5.2.1.4591.4001.140537559994289172851244415813/1.3.6.1.4.1.14519.5.2.1.4591.4001.160473331117007298735566672719	
TCGA-GBM	TCGA-06	TCGA-06-0142	t2	M			AXIAL_FSE	3	2.8195E+15	3/11/2000		BAY2BAY2	SE	2D	3	3500	20		6.38E+08	1.5	3	90	1.701089	112	256	192	0.937500\0.937500	TCGA-06-0142/1.3.6.1.4.1.14519.5.2.1.4591.4001.140537559994289172851244415813/1.3.6.1.4.1.14519.5.2.1.4591.4001.246556599071909674613941779916	
TCGA-GBM	TCGA-06	TCGA-06-0145	flair	F	52	73.482	AXIAL_FLAIR	4	6.78517E+15	11/6/2000	GE MEDICAL SYSTEMS	BAY2BAY2	GENESIS_SIGNA	IR	2D	3	10004	155	2200	6.38E+08	1.5	3	90	0.033739	54	256	192	0.937500\0.937500	TCGA-06-0145/1.3.6.1.4.1.14519.5.2.1.4591.4001.312057250385941099348613546751/1.3.6.1.4.1.14519.5.2.1.4591.4001.100984269677348148072147090714
TCGA-GBM	TCGA-06	TCGA-06-0145	t1Gd	F	52	73.482	AXIAL_T1_GD	10	6.78517E+15	11/6/2000	GE MEDICAL SYSTEMS	BAY2BAY2	GENESIS_SIGNA	SE	2D	3	500	14		6.38E+08	1.5	3	90	0.041793	54	256	192	0.937500\0.937500	TCGA-06-0145/1.3.6.1.4.1.14519.5.2.1.4591.4001.312057250385941099348613546751/1.3.6.1.4.1.14519.5.2.1.4591.4001.332902950048400716837387278761
TCGA-GBM	TCGA-06	TCGA-06-0145	t1	F	52	73.482	AXIAL_T1	8	6.78517E+15	11/6/2000	GE MEDICAL SYSTEMS	BAY2BAY2	GENESIS_SIGNA	SE	2D	3	500	14		6.38E+08	1.5	3	90	0.041793	54	256	192	0.937500\0.937500	TCGA-06-0145/1.3.6.1.4.1.14519.5.2.1.4591.4001.312057250385941099348613546751/1.3.6.1.4.1.14519.5.2.1.4591.4001.315639171460581681490878614022
TCGA-GBM	TCGA-06	TCGA-06-0145	t2	F	52	73.482	AXIAL_FSE	3	6.78517E+15	11/6/2000	GE MEDICAL SYSTEMS	BAY2BAY2	GENESIS_SIGNA	SE	2D	3	3500	20		6.38E+08	1.5	3	90	0.055821	108	256	192	0.937500\0.937500	TCGA-06-0145/1.3.6.1.4.1.14519.5.2.1.4591.4001.312057250385941099348613546751/1.3.6.1.4.1.14519.5.2.1.4591.4001.124843654110488034987706152331
TCGA-GBM	TCGA-06	TCGA-06-0149	flair	F			AXIAL_T2_FLAIR	5	1.82811E+15	3/25/2003		MR3T		IR	2D	2.5	10002	124.9	2250	127.7325	3	2.5	90	0.5979	70	320	224	0.46875\0.46875	TCGA-06-0149/1.3.6.1.4.1.14519.5.2.1.4591.4001.118072519901339114398931787536/1.3.6.1.4.1.14519.5.2.1.4591.4001.112272323130683081580304494682
TCGA-GBM	TCGA-06	TCGA-06-0149	t1Gd	F			AX_T1_POST_GD_FLAIR	9	1.82811E+15	3/25/2003		MR3T		IR	2D	2.5	3222.99	6.568	1238	127.7325	3	2.5	90	1.6875	70	320	224	0.46875\0.46875	TCGA-06-0149/1.3.6.1.4.1.14519.5.2.1.4591.4001.118072519901339114398931787536/1.3.6.1.4.1.14519.5.2.1.4591.4001.136467249644763011967454408425
TCGA-GBM	TCGA-06	TCGA-06-0149	t1	F			AX_T1_pre_gd	7	1.82811E+15	3/25/2003		MR3T		IR	2D	5	3042.13	6.584	1238	127.7325	3	5	90	1.3409	36	320	224	0.46875\0.46875	TCGA-06-0149/1.3.6.1.4.1.14519.5.2.1.4591.4001.118072519901339114398931787536/1.3.6.1.4.1.14519.5.2.1.4591.4001.105210083102263310494367161428
TCGA-GBM	TCGA-06	TCGA-06-0149	t2	F			AX_T2_FR-FSE_RF2_150	4	1.82811E+15	3/25/2003		MR3T		SE	2D	5	3000	104.436	0	127.7325	3	5	90	1.0799	36	320	224	0.46875\0.46875	TCGA-06-0149/1.3.6.1.4.1.14519.5.2.1.4591.4001.118072519901339114398931787536/1.3.6.1.4.1.14519.5.2.1.4591.4001.250187065726873055656948737218
TCGA-GBM	TCGA-06	TCGA-06-0154	flair	M				3	3.23894E+15	4/5/1996		BAY		flair		3	1000	155	2200						56			0.94\0.94	TCGA-06-0154/1.3.6.1.4.1.14519.5.2.1.4591.4001.163246911709429038918826429573/1.3.6.1.4.1.14519.5.2.1.4591.4001.105686167309383153415308758602
TCGA-GBM	TCGA-06	TCGA-06-0154	t1Gd	M			AXIAL_T1_PRE/POST_GD	8	3.23894E+15	4/5/1996		BAY		memp		3	500	8	0			3	90		56			0.938\0.938	TCGA-06-0154/1.3.6.1.4.1.14519.5.2.1.4591.4001.163246911709429038918826429573/1.3.6.1.4.1.14519.5.2.1.4591.4001.145285442414651713189537273383
TCGA-GBM	TCGA-06	TCGA-06-0154	t1	M			AXIAL_T1_PRE/POST_GD	6	3.23894E+15	4/5/1996		BAY		memp		3	500	8	0			3	90		56			0.938\0.938	TCGA-06-0154/1.3.6.1.4.1.14519.5.2.1.4591.4001.163246911709429038918826429573/1.3.6.1.4.1.14519.5.2.1.4591.4001.352404364946080402874319891442
TCGA-GBM	TCGA-06	TCGA-06-0154	t2	M			AXIAL_FSE	2	3.23894E+15	4/5/1996		BAY		fse		3	3500	22	0			3	90		112			0.938\0.938	TCGA-06-0154/1.3.6.1.4.1.14519.5.2.1.4591.4001.163246911709429038918826429573/1.3.6.1.4.1.14519.5.2.1.4591.4001.693437937502992071234421184617
TCGA-GBM	TCGA-06	TCGA-06-0158	flair	M			AXIAL_FLAIR	3	7.69267E+15	9/5/1996		BAY		flair		3	10004	155	2200			3	90		53			0.938\0.938	TCGA-06-0158/1.3.6.1.4.1.14519.5.2.1.4591.4001.222356408466975831011403030968/1.3.6.1.4.1.14519.5.2.1.4591.4001.274441692198883693078259097052
TCGA-GBM	TCGA-06	TCGA-06-0158	t1Gd	M			AXIAL_T1_POST_GD	5	7.69267E+15	9/5/1996		BAY		memp		3	500	14	0			3	90		53			0.938\0.938	TCGA-06-0158/1.3.6.1.4.1.14519.5.2.1.4591.4001.222356408466975831011403030968/1.3.6.1.4.1.14519.5.2.1.4591.4001.173289171872890553883773995785
TCGA-GBM	TCGA-06	TCGA-06-0158	t1	M			AXIAL_T1_PRE_GD	2	7.69267E+15	9/5/1996		BAY		memp		3	500	14	0			3	90		53			0.938\0.938	TCGA-06-0158/1.3.6.1.4.1.14519.5.2.1.4591.4001.222356408466975831011403030968/1.3.6.1.4.1.14519.5.2.1.4591.4001.518026839168479419278007929122
TCGA-GBM	TCGA-06	TCGA-06-0158	t2	M			AXIAL_FSE	2	7.69267E+15	9/5/1996		BAY		fse		3	3500	22	0			3	90		102			0.938\0.938	TCGA-06-0158/1.3.6.1.4.1.14519.5.2.1.4591.4001.222356408466975831011403030968/1.3.6.1.4.1.14519.5.2.1.4591.4001.139278893498918379783151781430
TCGA-GBM	TCGA-06	TCGA-06-0162	flair	F			AXIAL_FLAIR	3	2.58526E+15	12/7/1998		BAY		flair		3	10004	155	2200			3	90		51			0.938\0.938	TCGA-06-0162/1.3.6.1.4.1.14519.5.2.1.4591.4001.680171398593107658050490549032/1.3.6.1.4.1.14519.5.2.1.4591.4001.125093007120701827268088161895
TCGA-GBM	TCGA-06	TCGA-06-0162	t1Gd	F			AXIAL_T1_POST_GD	7	2.58526E+15	12/7/1998		BAY		memp		3	500	14	0			3	90		51			0.938\0.938	TCGA-06-0162/1.3.6.1.4.1.14519.5.2.1.4591.4001.680171398593107658050490549032/1.3.6.1.4.1.14519.5.2.1.4591.4001.235424499795225610529331169998
TCGA-GBM	TCGA-06	TCGA-06-0162	t1	F			AXIAL_T1_PRE_GD	5	2.58526E+15	12/7/1998		BAY		memp		3	500	14	0			3	90		51			0.938\0.938	TCGA-06-0162/1.3.6.1.4.1.14519.5.2.1.4591.4001.680171398593107658050490549032/1.3.6.1.4.1.14519.5.2.1.4591.4001.198868156954238484147941810221
TCGA-GBM	TCGA-06	TCGA-06-0162	t2	F			AXIAL__FSE	2	2.58526E+15	12/7/1998		BAY		fse		3	3500	22	0			3	90		102			0.938\0.938	TCGA-06-0162/1.3.6.1.4.1.14519.5.2.1.4591.4001.680171398593107658050490549032/1.3.6.1.4.1.14519.5.2.1.4591.4001.211482278670119430307242095853
TCGA-GBM	TCGA-06	TCGA-06-0164	flair	M			AXIAL_FLAIR	3	1.74001E+15	2/17/1999		BAY1OC0		RM	2D	3	10004	155		63.78557	1.5	3	90	0.039235	55	256	192	0.9375000000\0.9375000000	TCGA-06-0164/1.3.6.1.4.1.14519.5.2.1.4591.4001.278844795194298910084884570122/1.3.6.1.4.1.14519.5.2.1.4591.4001.598245930322114156315750780139
TCGA-GBM	TCGA-06	TCGA-06-0164	t1Gd	M			AXIAL_T1_POST_GD	11	1.74001E+15	2/17/1999		BAY1OC0		SE	2D	3	500	14		63.78555	1.5	3	90	0.041659	55	256	192	0.9375000000\0.9375000000	TCGA-06-0164/1.3.6.1.4.1.14519.5.2.1.4591.4001.278844795194298910084884570122/1.3.6.1.4.1.14519.5.2.1.4591.4001.325638225787739653579401254779
TCGA-GBM	TCGA-06	TCGA-06-0164	t1	M			AXIAL_T1_PRE	5	1.74001E+15	2/17/1999		BAY1OC0		SE	2D	3	500	14		63.78557	1.5	3	90	0.041659	55	256	192	0.9375000000\0.9375000000	TCGA-06-0164/1.3.6.1.4.1.14519.5.2.1.4591.4001.278844795194298910084884570122/1.3.6.1.4.1.14519.5.2.1.4591.4001.248569781958585320389571424266
TCGA-GBM	TCGA-06	TCGA-06-0164	t2	M			AXIAL__FSE	2	1.74001E+15	2/17/1999		BAY1OC0		SE	2D	3	3500	22		63.78558	1.5	3	90	0.05729	110	256	192	0.9375000000\0.9375000000	TCGA-06-0164/1.3.6.1.4.1.14519.5.2.1.4591.4001.278844795194298910084884570122/1.3.6.1.4.1.14519.5.2.1.4591.4001.187337638416414033994910403506
TCGA-GBM	TCGA-06	TCGA-06-0166	flair	M			AXIAL_FLAIR	3	1.46824E+15	6/1/1999		BAY		flair		3	10004	155	2200			3	90		57			0.938\0.938	TCGA-06-0166/1.3.6.1.4.1.14519.5.2.1.4591.4001.312918919617224104082765445485/1.3.6.1.4.1.14519.5.2.1.4591.4001.721833977766553318114128596643
TCGA-GBM	TCGA-06	TCGA-06-0166	t1Gd	M			AXIAL_T1_POST_GD	7	1.46824E+15	6/1/1999		BAY		memp		3	500	14	0			3	90		57			0.938\0.938	TCGA-06-0166/1.3.6.1.4.1.14519.5.2.1.4591.4001.312918919617224104082765445485/1.3.6.1.4.1.14519.5.2.1.4591.4001.337430353322187787108565325391
TCGA-GBM	TCGA-06	TCGA-06-0166	t1	M			AXIAL_T1_PRE_GD	5	1.46824E+15	6/1/1999		BAY		memp		3	500	14	0			3	90		57			0.938\0.938	TCGA-06-0166/1.3.6.1.4.1.14519.5.2.1.4591.4001.312918919617224104082765445485/1.3.6.1.4.1.14519.5.2.1.4591.4001.261991934956286396033599671586
TCGA-GBM	TCGA-06	TCGA-06-0166	t2	M			AXIAL__FSE	2	1.46824E+15	6/1/1999		BAY		fse		3	3500	22	0			3	90		114			0.938\0.938	TCGA-06-0166/1.3.6.1.4.1.14519.5.2.1.4591.4001.312918919617224104082765445485/1.3.6.1.4.1.14519.5.2.1.4591.4001.236385881105540723406863292408
TCGA-GBM	TCGA-06	TCGA-06-0168	flair	F			AXIAL_FLAIR	5	2.8195E+15	8/12/2000		BAY2BAY2	IR	2D	3	10004	155	2200	6.38E+08	1.5	3	90	0.031899	56	256	192	0.937500\0.937500	TCGA-06-0168/1.3.6.1.4.1.14519.5.2.1.4591.4001.183683152249300569710859088871/1.3.6.1.4.1.14519.5.2.1.4591.4001.247852152024838103066111644027	
TCGA-GBM	TCGA-06	TCGA-06-0168	t1Gd	F			AXIAL_T1_GD	10	2.8195E+15	8/12/2000		BAY2BAY2	SE	2D	3	500	14		6.38E+08	1.5	3	90	0.039514	56	256	192	0.937500\0.937500	TCGA-06-0168/1.3.6.1.4.1.14519.5.2.1.4591.4001.183683152249300569710859088871/1.3.6.1.4.1.14519.5.2.1.4591.4001.839741962266350805891879065337	
TCGA-GBM	TCGA-06	TCGA-06-0168	t1	F			AXIAL_T1	8	2.8195E+15	8/12/2000		BAY2BAY2	SE	2D	3	500	14		6.38E+08	1.5	3	90	0.039514	56	256	192	0.937500\0.937500	TCGA-06-0168/1.3.6.1.4.1.14519.5.2.1.4591.4001.183683152249300569710859088871/1.3.6.1.4.1.14519.5.2.1.4591.4001.231945653959668895422748933210	
TCGA-GBM	TCGA-06	TCGA-06-0168	t2	F			AXIAL_FSE	4	2.8195E+15	8/12/2000		BAY2BAY2	SE	2D	3	3500	20		6.38E+08	1.5	3	90	0.054732	112	256	192	0.937500\0.937500	TCGA-06-0168/1.3.6.1.4.1.14519.5.2.1.4591.4001.183683152249300569710859088871/1.3.6.1.4.1.14519.5.2.1.4591.4001.285985999678514345339762054607	
TCGA-GBM	TCGA-06	TCGA-06-0175	flair	M			AXIAL_FLAIR	5	2.68236E+15	11/20/2001		MR01MROW	IR	2D	3	10004	149.048	2200	6.39E+08	1.5	3	90	0.0326	62	256	192	0.9375\0.9375	TCGA-06-0175/1.3.6.1.4.1.14519.5.2.1.4591.4001.845891727594474018825776844085/1.3.6.1.4.1.14519.5.2.1.4591.4001.332198392917983887866042069159	
TCGA-GBM	TCGA-06	TCGA-06-0175	t1Gd	M			AXIAL_T1_GD	10	2.68236E+15	11/20/2001		MR01MROW	SE	2D	3	500	13		6.39E+08	1.5	3	90	0.046179	62	256	192	0.9375\0.9375	TCGA-06-0175/1.3.6.1.4.1.14519.5.2.1.4591.4001.845891727594474018825776844085/1.3.6.1.4.1.14519.5.2.1.4591.4001.457501165871046087756658548895	
TCGA-GBM	TCGA-06	TCGA-06-0175	t1	M			AXIAL_T1	8	2.68236E+15	11/20/2001		MR01MROW	SE	2D	3	500	13		6.39E+08	1.5	3	90	0.046179	62	256	192	0.9375\0.9375	TCGA-06-0175/1.3.6.1.4.1.14519.5.2.1.4591.4001.845891727594474018825776844085/1.3.6.1.4.1.14519.5.2.1.4591.4001.298011777083077277088460913040	
TCGA-GBM	TCGA-06	TCGA-06-0175	t2	M			AXIAL_FSE	4	2.68236E+15	11/20/2001		MR01MROW	SE	2D	3	3500	65.216		6.39E+08	1.5	3	90	0.1018	124	256	192	0.9375\0.9375	TCGA-06-0175/1.3.6.1.4.1.14519.5.2.1.4591.4001.845891727594474018825776844085/1.3.6.1.4.1.14519.5.2.1.4591.4001.248832015830985831479055831470	
TCGA-GBM	TCGA-06	TCGA-06-0176	flair	M			AXIAL_FLAIR	4	2.61862E+15	4/9/2002		BAY2OC0		IR	2D	3	10004	155	2200	6.38E+08	1.5	3	90	0.033175	60	256	192	0.9375\0.9375	TCGA-06-0176/1.3.6.1.4.1.14519.5.2.1.4591.4001.198999303901323755527019586055/1.3.6.1.4.1.14519.5.2.1.4591.4001.107610771943798529727458745540
TCGA-GBM	TCGA-06	TCGA-06-0176	t1Gd	M			AXIAL_T1_GD	9	2.61862E+15	4/9/2002		BAY2OC0		SE	2D	3	600	14		6.38E+08	1.5	3	90	0.048888	60	256	192	0.9375\0.9375	TCGA-06-0176/1.3.6.1.4.1.14519.5.2.1.4591.4001.198999303901323755527019586055/1.3.6.1.4.1.14519.5.2.1.4591.4001.304427305594571200001961934814
TCGA-GBM	TCGA-06	TCGA-06-0176	t1	M			AXIAL_T1	7	2.61862E+15	4/9/2002		BAY2OC0		SE	2D	3	600	14		6.38E+08	1.5	3	90	0.048888	60	256	192	0.9375\0.9375	TCGA-06-0176/1.3.6.1.4.1.14519.5.2.1.4591.4001.198999303901323755527019586055/1.3.6.1.4.1.14519.5.2.1.4591.4001.159192685899774190444844541113
TCGA-GBM	TCGA-06	TCGA-06-0176	t2	M			AXIAL_FSE	3	2.61862E+15	4/9/2002		BAY2OC0		SE	2D	3	3266.66	20		6.38E+08	1.5	3	90	0.065345	120	256	192	0.9375\0.9375	TCGA-06-0176/1.3.6.1.4.1.14519.5.2.1.4591.4001.198999303901323755527019586055/1.3.6.1.4.1.14519.5.2.1.4591.4001.137484989470516487260129921893
TCGA-GBM	TCGA-06	TCGA-06-0177	flair	M			Ax_FLAIR_2.5mm_for_surgery	4	1.44338E+15	5/25/2002		MR01MROW	IR	2D	2.5	10004	149.544	2200	63.88592	1.5	2.5	90	0.0331	68	256	192	0.9375\0.9375	TCGA-06-0177/1.3.6.1.4.1.14519.5.2.1.4591.4001.145527987060041086616654852708/1.3.6.1.4.1.14519.5.2.1.4591.4001.176834268304831977485617496866	
TCGA-GBM	TCGA-06	TCGA-06-0177	t1Gd	M			Ax_T1_2.5mm_for_surgery	8	1.44338E+15	5/25/2002		MR01MROW	SE	2D	2.5	500	13	0	63.88592	1.5	2.5	90	0.0499	68	256	192	0.9375\0.9375	TCGA-06-0177/1.3.6.1.4.1.14519.5.2.1.4591.4001.145527987060041086616654852708/1.3.6.1.4.1.14519.5.2.1.4591.4001.125672926847564208162176608941	
TCGA-GBM	TCGA-06	TCGA-06-0177	t1	M			AXIAL_T1	6	1.44338E+15	5/25/2002		MR01MROW	SE	2D	5	500	14	0	63.88592	1.5	5	90	0.0499	34	256	192	0.9375\0.9375	TCGA-06-0177/1.3.6.1.4.1.14519.5.2.1.4591.4001.145527987060041086616654852708/1.3.6.1.4.1.14519.5.2.1.4591.4001.884924697454363427147927460748	
TCGA-GBM	TCGA-06	TCGA-06-0177	t2	M			AXIAL_FSE	3	1.44338E+15	5/25/2002		MR01MROW	SE	2D	5	3500	63.168	0	63.88592	1.5	5	90	0.1135	68	256	192	0.9375\0.9375	TCGA-06-0177/1.3.6.1.4.1.14519.5.2.1.4591.4001.145527987060041086616654852708/1.3.6.1.4.1.14519.5.2.1.4591.4001.227594382691920181249054186980	
TCGA-GBM	TCGA-06	TCGA-06-0179	flair	M			AXIAL_T2_FLAIR	7	2.74487E+15	7/27/2002		MR3T		IR	2D	2.5	10002	124.9	2250	127.7324	3	2.5	90	0.6658	71	320	224	0.46875\0.46875	TCGA-06-0179/1.3.6.1.4.1.14519.5.2.1.4591.4001.803812054574937880227208773217/1.3.6.1.4.1.14519.5.2.1.4591.4001.229925037084238292303114803384
TCGA-GBM	TCGA-06	TCGA-06-0179	t1Gd	M			AX_T1_POST_GD_FLAIR	11	2.74487E+15	7/27/2002		MR3T		IR	2D	2.5	3047.38	6.956	1238	127.7324	3	2.5	90	1.484	71	320	224	0.46875\0.46875	TCGA-06-0179/1.3.6.1.4.1.14519.5.2.1.4591.4001.803812054574937880227208773217/1.3.6.1.4.1.14519.5.2.1.4591.4001.186621475598111141744713394004
TCGA-GBM	TCGA-06	TCGA-06-0179	t1	M			AX_T1_pre_gd	9	2.74487E+15	7/27/2002		MR3T		IR	2D	5	3047.46	6.964	1238	127.7324	3	5	90	1.484	36	320	224	0.46875\0.46875	TCGA-06-0179/1.3.6.1.4.1.14519.5.2.1.4591.4001.803812054574937880227208773217/1.3.6.1.4.1.14519.5.2.1.4591.4001.265101231179155598255544655629
TCGA-GBM	TCGA-06	TCGA-06-0179	t2	M			AX_T2_FR-FSE_RF2_150	5	2.74487E+15	7/27/2002		MR3T		SE	2D	5	3000	104.436	0	127.7324	3	5	90	1.1837	36	320	224	0.46875\0.46875	TCGA-06-0179/1.3.6.1.4.1.14519.5.2.1.4591.4001.803812054574937880227208773217/1.3.6.1.4.1.14519.5.2.1.4591.4001.162280835080528945073223289120
TCGA-GBM	TCGA-06	TCGA-06-0182	flair	M	76	83.915	AxFLAIR-thin_for_surgery	7	1.15217E+15	9/1/2002	GE MEDICAL SYSTEMS	BAY2OC0	GENESIS_SIGNA	IR	2D	2.5	10004	155	2200	6.38E+08	1.5	2.5	90	0.033029	79	256	192	0.9375\0.9375	TCGA-06-0182/1.3.6.1.4.1.14519.5.2.1.4591.4001.257293214662038104885045596990/1.3.6.1.4.1.14519.5.2.1.4591.4001.310335155097062460898875231783
TCGA-GBM	TCGA-06	TCGA-06-0182	t1Gd	M	76	83.915	=+AXS1-thin_for_surgery	13	1.15217E+15	9/1/2002	GE MEDICAL SYSTEMS	BAY2OC0	GENESIS_SIGNA	SE	2D	2.5	500	13		6.38E+08	1.5	2.5	90	0.046759	79	256	192	0.9375\0.9375	TCGA-06-0182/1.3.6.1.4.1.14519.5.2.1.4591.4001.257293214662038104885045596990/1.3.6.1.4.1.14519.5.2.1.4591.4001.328210509441925795754155912089
TCGA-GBM	TCGA-06	TCGA-06-0182	t1	M	76	83.915	AXIAL_T1	9	1.15217E+15	9/1/2002	GE MEDICAL SYSTEMS	BAY2OC0	GENESIS_SIGNA	SE	2D	5	500	14		6.38E+08	1.5	5	90	0.040914	40	256	192	0.9375\0.9375	TCGA-06-0182/1.3.6.1.4.1.14519.5.2.1.4591.4001.257293214662038104885045596990/1.3.6.1.4.1.14519.5.2.1.4591.4001.149790107241376368982697513082
TCGA-GBM	TCGA-06	TCGA-06-0182	t2	M	76	83.915	AXIAL_FSE	6	1.15217E+15	9/1/2002	GE MEDICAL SYSTEMS	BAY2OC0	GENESIS_SIGNA	SE	2D	5	3500	20		6.38E+08	1.5	5	90	0.04048	80	256	192	0.9375\0.9375	TCGA-06-0182/1.3.6.1.4.1.14519.5.2.1.4591.4001.257293214662038104885045596990/1.3.6.1.4.1.14519.5.2.1.4591.4001.285828690405234989005344319198
TCGA-GBM	TCGA-06	TCGA-06-0184	flair	M			Ax_FLAIR_2.5mm_for_surgery	6	1.89312E+15	7/13/2003		MR01MROW	IR	2D	2.5	10004	149.544	2200	63.88571	1.5	2.5	90	0.033	72	256	192	0.9375\0.9375	TCGA-06-0184/1.3.6.1.4.1.14519.5.2.1.4591.4001.305033536698386255017041052545/1.3.6.1.4.1.14519.5.2.1.4591.4001.298488025067598105207946452228	
TCGA-GBM	TCGA-06	TCGA-06-0184	t1Gd	M			Ax_T1_2.5mm_for_surgery	10	1.89312E+15	7/13/2003		MR01MROW	SE	2D	2.5	516.664	13	0	63.88574	1.5	2.5	90	0.050901	72	256	192	0.9375\0.9375	TCGA-06-0184/1.3.6.1.4.1.14519.5.2.1.4591.4001.305033536698386255017041052545/1.3.6.1.4.1.14519.5.2.1.4591.4001.902471635951021018846680182923	
TCGA-GBM	TCGA-06	TCGA-06-0184	t1	M			AXIAL_T1	8	1.89312E+15	7/13/2003		MR01MROW	SE	2D	5	550	14	0	63.88574	1.5	5	90	0.047805	36	256	192	0.9375\0.9375	TCGA-06-0184/1.3.6.1.4.1.14519.5.2.1.4591.4001.305033536698386255017041052545/1.3.6.1.4.1.14519.5.2.1.4591.4001.421295141066363874489270570138	
TCGA-GBM	TCGA-06	TCGA-06-0184	t2	M			AXIAL_FSE	5	1.89312E+15	7/13/2003		MR01MROW	SE	2D	5	3500	23.688	0	63.88571	1.5	5	90	0.1197	72	256	192	0.9375\0.9375	TCGA-06-0184/1.3.6.1.4.1.14519.5.2.1.4591.4001.305033536698386255017041052545/1.3.6.1.4.1.14519.5.2.1.4591.4001.784491244328130916312511268061	
TCGA-GBM	TCGA-06	TCGA-06-0185	flair	M			AxFLAIR-thin_for_surgery	4	2.61836E+15	11/7/2003		BAY2OC0		IR	2D	2.5	10004	155	2200	6.38E+08	1.5	2.5	90	0.03384	73	256	192	0.9375\0.9375	TCGA-06-0185/1.3.6.1.4.1.14519.5.2.1.4591.4001.232938998404177432213094426945/1.3.6.1.4.1.14519.5.2.1.4591.4001.131233116352308759897551017004
TCGA-GBM	TCGA-06	TCGA-06-0185	t1Gd	M			GD_AxT1-thin_for_surgery	11	2.61836E+15	11/7/2003		BAY2OC0		SE	2D	2.5	500	13		6.38E+08	1.5	2.5	90	0.044912	71	256	192	0.9375\0.9375	TCGA-06-0185/1.3.6.1.4.1.14519.5.2.1.4591.4001.232938998404177432213094426945/1.3.6.1.4.1.14519.5.2.1.4591.4001.266324093776988037240038212588
TCGA-GBM	TCGA-06	TCGA-06-0185	t1	M			AXIAL_T1	7	2.61836E+15	11/7/2003		BAY2OC0		SE	2D	5	500	14		6.38E+08	1.5	5	90	0.038924	37	256	192	0.9375\0.9375	TCGA-06-0185/1.3.6.1.4.1.14519.5.2.1.4591.4001.232938998404177432213094426945/1.3.6.1.4.1.14519.5.2.1.4591.4001.222960602322356165635258148379
TCGA-GBM	TCGA-06	TCGA-06-0185	t2	M			AXIAL_FSE	3	2.61836E+15	11/7/2003		BAY2OC0		SE	2D	5	3500	20		6.38E+08	1.5	5	90	0.039399	74	256	192	0.9375\0.9375	TCGA-06-0185/1.3.6.1.4.1.14519.5.2.1.4591.4001.232938998404177432213094426945/1.3.6.1.4.1.14519.5.2.1.4591.4001.513303216697905784166950817543
TCGA-GBM	TCGA-06	TCGA-06-0187	flair	M			AxFLAIR-thin_for_surgery	7	1.24186E+15	7/7/2004		BAY2OC0		IR	2D	2.5	10004	155	2200	6.38E+08	1.5	2.5	90	0.031943	71	256	192	0.9375\0.9375	TCGA-06-0187/1.3.6.1.4.1.14519.5.2.1.4591.4001.216767337030769268485462841002/1.3.6.1.4.1.14519.5.2.1.4591.4001.539190347422929234900620483857
TCGA-GBM	TCGA-06	TCGA-06-0187	t1Gd	M			=+AXS1-thin_for_surgery	12	1.24186E+15	7/7/2004		BAY2OC0		SE	2D	2.5	500	13		6.38E+08	1.5	2.5	90	0.048048	68	256	192	0.9375\0.9375	TCGA-06-0187/1.3.6.1.4.1.14519.5.2.1.4591.4001.216767337030769268485462841002/1.3.6.1.4.1.14519.5.2.1.4591.4001.222791162703643401365433542560
TCGA-GBM	TCGA-06	TCGA-06-0187	t1	M			AXIAL_T1	9	1.24186E+15	7/7/2004		BAY2OC0		SE	2D	5	500	14		6.38E+08	1.5	5	90	0.033916	36	256	192	0.9375\0.9375	TCGA-06-0187/1.3.6.1.4.1.14519.5.2.1.4591.4001.216767337030769268485462841002/1.3.6.1.4.1.14519.5.2.1.4591.4001.186471094046575208336353039993
TCGA-GBM	TCGA-06	TCGA-06-0187	t2	M			AXIAL_FSE	6	1.24186E+15	7/7/2004		BAY2OC0		SE	2D	5	3500	20		6.38E+08	1.5	5	90	0.035234	72	256	192	0.9375\0.9375	TCGA-06-0187/1.3.6.1.4.1.14519.5.2.1.4591.4001.216767337030769268485462841002/1.3.6.1.4.1.14519.5.2.1.4591.4001.318345880835704470736633622516
TCGA-GBM	TCGA-06	TCGA-06-0188	flair	M			AxFLAIR-thin_for_surgery	5	2.79037E+15	7/17/2004		BAY2OC0		IR	2D	2.5	10004	155	2200	6.38E+08	1.5	2.5	90	0.03364	68	256	192	0.9375\0.9375	TCGA-06-0188/1.3.6.1.4.1.14519.5.2.1.4591.4001.236824294948093279948709063675/1.3.6.1.4.1.14519.5.2.1.4591.4001.239261393324265132190998071373
TCGA-GBM	TCGA-06	TCGA-06-0188	t1Gd	M			=+AXS1-thin_for_surgery	9	2.79037E+15	7/17/2004		BAY2OC0		SE	2D	2.5	500	13		6.38E+08	1.5	2.5	90	0.050599	68	256	192	0.9375\0.9375	TCGA-06-0188/1.3.6.1.4.1.14519.5.2.1.4591.4001.236824294948093279948709063675/1.3.6.1.4.1.14519.5.2.1.4591.4001.115884482625152677354342118388
TCGA-GBM	TCGA-06	TCGA-06-0188	t1	M			AXIAL_T1	7	2.79037E+15	7/17/2004		BAY2OC0		SE	2D	5	516.664	14		6.38E+08	1.5	5	90	0.048961	34	256	192	0.9375\0.9375	TCGA-06-0188/1.3.6.1.4.1.14519.5.2.1.4591.4001.236824294948093279948709063675/1.3.6.1.4.1.14519.5.2.1.4591.4001.245392760904119688427626639535
TCGA-GBM	TCGA-06	TCGA-06-0188	t2	M			AXIAL_FSE	4	2.79037E+15	7/17/2004		BAY2OC0		SE	2D	5	3716.664	80		6.38E+08	1.5	5	90	0.065999	68	256	192	0.9375\0.9375	TCGA-06-0188/1.3.6.1.4.1.14519.5.2.1.4591.4001.236824294948093279948709063675/1.3.6.1.4.1.14519.5.2.1.4591.4001.285294068503087038157446001862
TCGA-GBM	TCGA-06	TCGA-06-0189	flair	M	55	97.522	AXIAL_FLAIR	5	2.17405E+15	10/2/2004	GE MEDICAL SYSTEMS	MR3T	SIGNA EXCITE	IR	2D	2.5	10002	125.4	2250	127.7322	3	2.5	90	0.7975	74	320	224	0.4688\0.4688	TCGA-06-0189/1.3.6.1.4.1.14519.5.2.1.4591.4001.271958506783844757321502711697/1.3.6.1.4.1.14519.5.2.1.4591.4001.204121947709766236097014303992
TCGA-GBM	TCGA-06	TCGA-06-0189	t1Gd	M	55	97.522	AX_T1_POST_GD_FLAIR	14	2.17405E+15	10/2/2004	GE MEDICAL SYSTEMS	MR3T	SIGNA EXCITE	IR	2D	2.5	2720.75	6.356	1154	127.7322	3	2.5	90	1.9775	76	320	224	0.4688\0.4688	TCGA-06-0189/1.3.6.1.4.1.14519.5.2.1.4591.4001.271958506783844757321502711697/1.3.6.1.4.1.14519.5.2.1.4591.4001.617019599027525799589736647295
TCGA-GBM	TCGA-06	TCGA-06-0189	t1	M	55	97.522	AX_T1_pre_gd	8	2.17405E+15	10/2/2004	GE MEDICAL SYSTEMS	MR3T	SIGNA EXCITE	IR	2D	5	3256.32	6.356	1238	127.7322	3	5	90	1.652	38	320	224	0.4688\0.4688	TCGA-06-0189/1.3.6.1.4.1.14519.5.2.1.4591.4001.271958506783844757321502711697/1.3.6.1.4.1.14519.5.2.1.4591.4001.322517941325558672246365758146
TCGA-GBM	TCGA-06	TCGA-06-0189	t2	M	55	97.522	AX_T2_FR-FSE_RF2_150	6	2.17405E+15	10/2/2004	GE MEDICAL SYSTEMS	MR3T	SIGNA EXCITE	SE	2D	5	3000	103.464	0	127.7322	3	5	90	1.4452	39	320	224	0.4688\0.4688	TCGA-06-0189/1.3.6.1.4.1.14519.5.2.1.4591.4001.271958506783844757321502711697/1.3.6.1.4.1.14519.5.2.1.4591.4001.287445780789038071301584012772
TCGA-GBM	TCGA-06	TCGA-06-0190	flair	M			AxFLAIR-thin_for_surgery	4	9.56561E+15	12/10/2004		BAY2OC0		IR	2D	2.5	10004	155	2200	6.39E+08	1.5	2.5	90	0.033029	71	256	192	0.9375\0.9375	TCGA-06-0190/1.3.6.1.4.1.14519.5.2.1.4591.4001.176500029848577499575043841856/1.3.6.1.4.1.14519.5.2.1.4591.4001.491441365887397929225136700818
TCGA-GBM	TCGA-06	TCGA-06-0190	t1Gd	M			=+AXS1-thin_for_surgery	8	9.56561E+15	12/10/2004		BAY2OC0		SE	2D	2.5	516.664	13		6.39E+08	1.5	2.5	90	0.050901	71	256	192	0.9375\0.9375	TCGA-06-0190/1.3.6.1.4.1.14519.5.2.1.4591.4001.176500029848577499575043841856/1.3.6.1.4.1.14519.5.2.1.4591.4001.671165767421867654226548857596
TCGA-GBM	TCGA-06	TCGA-06-0190	t1	M			AXIAL_T1	6	9.56561E+15	12/10/2004		BAY2OC0		SE	2D	5	550	14		6.39E+08	1.5	5	90	0.047805	36	256	192	0.9375\0.9375	TCGA-06-0190/1.3.6.1.4.1.14519.5.2.1.4591.4001.176500029848577499575043841856/1.3.6.1.4.1.14519.5.2.1.4591.4001.716572214843115361797157088313
TCGA-GBM	TCGA-06	TCGA-06-0190	t2	M			AXIAL_FSE	3	9.56561E+15	12/10/2004		BAY2OC0		SE	2D	5	3800	20		6.39E+08	1.5	5	90	0.067108	72	256	192	0.9375\0.9375	TCGA-06-0190/1.3.6.1.4.1.14519.5.2.1.4591.4001.176500029848577499575043841856/1.3.6.1.4.1.14519.5.2.1.4591.4001.119615138695506529759357598587
TCGA-GBM	TCGA-06	TCGA-06-0192	flair	M			AXIAL_FLAIR	6	2.9077E+15	4/8/2005		MR3T		IR	2D	2.5	10002	125.4	2250	127.7323	3	2.5	90	0.8357	71	320	224	0.4688\0.4688	TCGA-06-0192/1.3.6.1.4.1.14519.5.2.1.4591.4001.678770301318444628129910220947/1.3.6.1.4.1.14519.5.2.1.4591.4001.157734237416927367774289543725
TCGA-GBM	TCGA-06	TCGA-06-0192	t1Gd	M			AX_T1_POST_GD_FLAIR	10	2.9077E+15	4/8/2005		MR3T		IR	2D	2.5	3189.01	6.356	1238	127.7323	3	2.5	90	1.4927	71	320	224	0.4688\0.4688	TCGA-06-0192/1.3.6.1.4.1.14519.5.2.1.4591.4001.297257903497184376204809816721/1.3.6.1.4.1.14519.5.2.1.4591.4001.587950467567034197943180807635
TCGA-GBM	TCGA-06	TCGA-06-0192	t1	M			AX_T1_pre_gd	8	2.9077E+15	4/8/2005		MR3T		IR	2D	5	3000.01	6.356	1238	127.7323	3	5	90	1.1901	36	320	224	0.4688\0.4688	TCGA-06-0192/1.3.6.1.4.1.14519.5.2.1.4591.4001.678770301318444628129910220947/1.3.6.1.4.1.14519.5.2.1.4591.4001.242390157644773510605680848291
TCGA-GBM	TCGA-06	TCGA-06-0192	t2	M			AX_T2_FR-FSE_RF2_150	5	2.9077E+15	4/8/2005		MR3T		SE	2D	5	3000	103.464	0	127.7323	3	5	90	1.5901	36	320	224	0.4688\0.4688	TCGA-06-0192/1.3.6.1.4.1.14519.5.2.1.4591.4001.678770301318444628129910220947/1.3.6.1.4.1.14519.5.2.1.4591.4001.265674194081211616900213689317
TCGA-GBM	TCGA-06	TCGA-06-0213	flair	F			AXIAL_FLAIR	3	2.24926E+15	10/23/1996		BAY		flair		3	10002	155	2200			3	90		55			0.938\0.938	TCGA-06-0213/1.3.6.1.4.1.14519.5.2.1.4591.4001.126830789018616922158734856914/1.3.6.1.4.1.14519.5.2.1.4591.4001.868309828837037489455165428643
TCGA-GBM	TCGA-06	TCGA-06-0213	t1Gd	F			AXIAL_T1_GD	9	2.24926E+15	10/23/1996		BAY		memp		3	500	14	0			3	90		56			0.938\0.938	TCGA-06-0213/1.3.6.1.4.1.14519.5.2.1.4591.4001.126830789018616922158734856914/1.3.6.1.4.1.14519.5.2.1.4591.4001.712441540279598808730794710099
TCGA-GBM	TCGA-06	TCGA-06-0213	t1	F			AXIAL_T1_PRE	7	2.24926E+15	10/23/1996		BAY		memp		3	500	14	0			3	90		56			0.938\0.938	TCGA-06-0213/1.3.6.1.4.1.14519.5.2.1.4591.4001.126830789018616922158734856914/1.3.6.1.4.1.14519.5.2.1.4591.4001.132760894917845514939967527728
TCGA-GBM	TCGA-06	TCGA-06-0213	t2	F			AXIAL_FSE	2	2.24926E+15	10/23/1996		BAY		fse		3	3500	88	0			3	90		112			0.938\0.938	TCGA-06-0213/1.3.6.1.4.1.14519.5.2.1.4591.4001.126830789018616922158734856914/1.3.6.1.4.1.14519.5.2.1.4591.4001.201340664225508569277190185868
TCGA-GBM	TCGA-06	TCGA-06-0238	flair	M	46	68.039	AXIAL_FLAIR	5	1.73589E+15	4/12/2005	GE MEDICAL SYSTEMS	MR3T	SIGNA EXCITE	IR	2D	2.5	10002	125.4	2250	127.7323	3	2.5	90	0.5979	72	320	224	0.4688\0.4688	TCGA-06-0238/1.3.6.1.4.1.14519.5.2.1.4591.4001.234150403961428051973172824302/1.3.6.1.4.1.14519.5.2.1.4591.4001.157984242172762905506145896839
TCGA-GBM	TCGA-06	TCGA-06-0238	t1Gd	M	46	68.039	AX_T1_POST_GD_FLAIR	13	1.73589E+15	4/12/2005	GE MEDICAL SYSTEMS	MR3T	SIGNA EXCITE	IR	2D	2.5	3026.5	6.356	1238	127.7323	3	2.5	90	1.8694	84	320	224	0.4688\0.4688	TCGA-06-0238/1.3.6.1.4.1.14519.5.2.1.4591.4001.234150403961428051973172824302/1.3.6.1.4.1.14519.5.2.1.4591.4001.202504047648555593992532497928
TCGA-GBM	TCGA-06	TCGA-06-0238	t1	M	46	68.039	AX_T1_pre_gd	11	1.73589E+15	4/12/2005	GE MEDICAL SYSTEMS	MR3T	SIGNA EXCITE	IR	2D	5	3026.5	6.356	1238	127.7323	3	5	90	1.8694	42	320	224	0.4688\0.4688	TCGA-06-0238/1.3.6.1.4.1.14519.5.2.1.4591.4001.234150403961428051973172824302/1.3.6.1.4.1.14519.5.2.1.4591.4001.294172607033594086316981791804
TCGA-GBM	TCGA-06	TCGA-06-0238	t2	M	46	68.039	AX_T2_FR-FSE_RF2_150	9	1.73589E+15	4/12/2005	GE MEDICAL SYSTEMS	MR3T	SIGNA EXCITE	SE	2D	5	3000	103.464	0	127.7323	3	5	90	1.0799	36	320	224	0.4688\0.4688	TCGA-06-0238/1.3.6.1.4.1.14519.5.2.1.4591.4001.234150403961428051973172824302/1.3.6.1.4.1.14519.5.2.1.4591.4001.910762318489313615740449757293
TCGA-GBM	TCGA-06	TCGA-06-0240	flair	M			AxFLAIR-thin_for_surgery	4	2.74124E+15	7/2/2005		BAY2OC0		IR	2D	2.5	10004	155	2200	6.39E+08	1.5	2.5	90	0.03262	77	256	192	1.015625\1.015625	TCGA-06-0240/1.3.6.1.4.1.14519.5.2.1.4591.4001.104811886009138088457642404728/1.3.6.1.4.1.14519.5.2.1.4591.4001.329775737865220938751020847036
TCGA-GBM	TCGA-06	TCGA-06-0240	t1Gd	M			=+AXS1-thin_for_surgery	9	2.74124E+15	7/2/2005		BAY2OC0		SE	2D	2.5	500	13		6.39E+08	1.5	2.5	90	0.046179	77	256	192	1.015625\1.015625	TCGA-06-0240/1.3.6.1.4.1.14519.5.2.1.4591.4001.104811886009138088457642404728/1.3.6.1.4.1.14519.5.2.1.4591.4001.198392408592904340848985827598
TCGA-GBM	TCGA-06	TCGA-06-0240	t1	M			AXIAL_T1	6	2.74124E+15	7/2/2005		BAY2OC0		SE	2D	5	500	14		6.39E+08	1.5	5	90	0.037521	39	256	192	1.015625\1.015625	TCGA-06-0240/1.3.6.1.4.1.14519.5.2.1.4591.4001.104811886009138088457642404728/1.3.6.1.4.1.14519.5.2.1.4591.4001.222834836258287210760464320532
TCGA-GBM	TCGA-06	TCGA-06-0240	t2	M			AXIAL_FSE	3	2.74124E+15	7/2/2005		BAY2OC0		SE	2D	5	3500	80		6.39E+08	1.5	5	90	0.039978	78	256	192	1.015625\1.015625	TCGA-06-0240/1.3.6.1.4.1.14519.5.2.1.4591.4001.104811886009138088457642404728/1.3.6.1.4.1.14519.5.2.1.4591.4001.249974415144435604635369691012
TCGA-GBM	TCGA-06	TCGA-06-0241	flair	F			AXIAL_FLAIR	5	2.14612E+15	8/30/2005		MR3T		IR	2D	2.5	10002	125.4	2250	127.7322	3	2.5	90	0.6258	75	320	224	0.4688\0.4688	TCGA-06-0241/1.3.6.1.4.1.14519.5.2.1.4591.4001.278928118420898534392751993299/1.3.6.1.4.1.14519.5.2.1.4591.4001.129865339243072579534823799930
TCGA-GBM	TCGA-06	TCGA-06-0241	t1Gd	F			AX_T1_POST_GD_FLAIR	10	2.14612E+15	8/30/2005		MR3T		IR	2D	2.5	3000	6.356	1238	127.7322	3	2.5	90	1.3217	75	320	224	0.4688\0.4688	TCGA-06-0241/1.3.6.1.4.1.14519.5.2.1.4591.4001.278928118420898534392751993299/1.3.6.1.4.1.14519.5.2.1.4591.4001.170539642438067923558826595256
TCGA-GBM	TCGA-06	TCGA-06-0241	t1	F			AX_T1_pre_gd	8	2.14612E+15	8/30/2005		MR3T		IR	2D	5	3108.73	6.356	1238	127.7322	3	5	90	0.9693	38	320	224	0.4688\0.4688	TCGA-06-0241/1.3.6.1.4.1.14519.5.2.1.4591.4001.278928118420898534392751993299/1.3.6.1.4.1.14519.5.2.1.4591.4001.316434438257717295392918423241
TCGA-GBM	TCGA-06	TCGA-06-0241	t2	F			AX_T2_FR-FSE_RF2_150	6	2.14612E+15	8/30/2005		MR3T		SE	2D	5	3000	103.464	0	127.7322	3	5	90	0.9183	38	320	224	0.4688\0.4688	TCGA-06-0241/1.3.6.1.4.1.14519.5.2.1.4591.4001.278928118420898534392751993299/1.3.6.1.4.1.14519.5.2.1.4591.4001.196151036460577742361087055213
TCGA-GBM	TCGA-06	TCGA-06-0644	flair	M			AXIAL_FLAIR	5	3.92717E+15	11/28/2005		MR3T		IR	2D	2.5	10002	125.4	2250	127.7322	3	2.5	90	0.9012	75	320	224	0.4688\0.4688	TCGA-06-0644/1.3.6.1.4.1.14519.5.2.1.4591.4001.146995087154371962686852678030/1.3.6.1.4.1.14519.5.2.1.4591.4001.263636323693757141556368836667
TCGA-GBM	TCGA-06	TCGA-06-0644	t1Gd	M			AX_T1_POST_GD_FLAIR	10	3.92717E+15	11/28/2005		MR3T		IR	2D	2.5	3228.58	6.356	1238	127.7322	3	2.5	90	1.528	75	320	224	0.4688\0.4688	TCGA-06-0644/1.3.6.1.4.1.14519.5.2.1.4591.4001.146995087154371962686852678030/1.3.6.1.4.1.14519.5.2.1.4591.4001.922420967850060003702279065253
TCGA-GBM	TCGA-06	TCGA-06-0644	t1	M			AX_T1_pre_gd	8	3.92717E+15	11/28/2005		MR3T		IR	2D	5	3228.66	6.356	1238	127.7322	3	5	90	1.5279	38	320	224	0.4688\0.4688	TCGA-06-0644/1.3.6.1.4.1.14519.5.2.1.4591.4001.146995087154371962686852678030/1.3.6.1.4.1.14519.5.2.1.4591.4001.138727768548758840916384891966
TCGA-GBM	TCGA-06	TCGA-06-0644	t2	M			AX_T2_FR-FSE_RF2_150	6	3.92717E+15	11/28/2005		MR3T		SE	2D	5	3000	103.464	0	127.7322	3	5	90	1.5033	38	320	224	0.4688\0.4688	TCGA-06-0644/1.3.6.1.4.1.14519.5.2.1.4591.4001.146995087154371962686852678030/1.3.6.1.4.1.14519.5.2.1.4591.4001.621008573828456994425541022047
TCGA-GBM	TCGA-06	TCGA-06-0646	flair	M			AXIAL_FLAIR	5	2.5659E+15	12/9/2005		MR3T		IR	2D	2.5	10002	125.4	2250	127.7322	3	2.5	90	1.0022	72	320	224	0.4688\0.4688	TCGA-06-0646/1.3.6.1.4.1.14519.5.2.1.4591.4001.384328429938630524147521856216/1.3.6.1.4.1.14519.5.2.1.4591.4001.140001491379116667324415668587
TCGA-GBM	TCGA-06	TCGA-06-0646	t1Gd	M			AX_T1_POST_GD_FLAIR	10	2.5659E+15	12/9/2005		MR3T		IR	2D	2.5	3054.22	6.356	1238	127.7322	3	2.5	90	1.5065	72	320	224	0.4688\0.4688	TCGA-06-0646/1.3.6.1.4.1.14519.5.2.1.4591.4001.384328429938630524147521856216/1.3.6.1.4.1.14519.5.2.1.4591.4001.133122288144320695822686611824
TCGA-GBM	TCGA-06	TCGA-06-0646	t1	M			AX_T1_pre_gd	8	2.5659E+15	12/9/2005		MR3T		IR	2D	5	3054.3	6.356	1238	127.7322	3	5	90	1.5064	36	320	224	0.4688\0.4688	TCGA-06-0646/1.3.6.1.4.1.14519.5.2.1.4591.4001.384328429938630524147521856216/1.3.6.1.4.1.14519.5.2.1.4591.4001.176490286279929598870187285927
TCGA-GBM	TCGA-06	TCGA-06-0646	t2	M			AX_T2_FR-FSE_RF2_150	6	2.5659E+15	12/9/2005		MR3T		SE	2D	5	3000	103.464	0	127.7322	3	5	90	1.3661	36	320	224	0.4688\0.4688	TCGA-06-0646/1.3.6.1.4.1.14519.5.2.1.4591.4001.384328429938630524147521856216/1.3.6.1.4.1.14519.5.2.1.4591.4001.104763531222810995600952616748
TCGA-GBM	TCGA-06	TCGA-06-0648	flair	M			AXIAL_FLAIR	6	3.12165E+15	1/20/2006		MR3T		IR	2D	2.5	10002	125.4	2250	127.7322	3	2.5	90	0.7669	71	320	224	0.4688\0.4688	TCGA-06-0648/1.3.6.1.4.1.14519.5.2.1.4591.4001.177101472748785866117292551419/1.3.6.1.4.1.14519.5.2.1.4591.4001.956802103164319379282258280030
TCGA-GBM	TCGA-06	TCGA-06-0648	t1Gd	M			AX_T1_POST_GD_FLAIR	11	3.12165E+15	1/20/2006		MR3T		IR	2D	2.5	3161.55	6.356	1238	127.7322	3	2.5	90	1.3317	71	320	224	0.4688\0.4688	TCGA-06-0648/1.3.6.1.4.1.14519.5.2.1.4591.4001.177101472748785866117292551419/1.3.6.1.4.1.14519.5.2.1.4591.4001.461813783107058700607975602197
TCGA-GBM	TCGA-06	TCGA-06-0648	t1	M			AX_T1_pre_gd	9	3.12165E+15	1/20/2006		MR3T		IR	2D	5	3000.01	6.356	1238	127.7322	3	5	90	1.0526	36	320	224	0.4688\0.4688	TCGA-06-0648/1.3.6.1.4.1.14519.5.2.1.4591.4001.177101472748785866117292551419/1.3.6.1.4.1.14519.5.2.1.4591.4001.310583318436210971810347049929
TCGA-GBM	TCGA-06	TCGA-06-0648	t2	M			AX_T2_FR-FSE_RF2_150	7	3.12165E+15	1/20/2006		MR3T		SE	2D	5	3000	103.464	0	127.7322	3	5	90	1.4064	36	320	224	0.4688\0.4688	TCGA-06-0648/1.3.6.1.4.1.14519.5.2.1.4591.4001.177101472748785866117292551419/1.3.6.1.4.1.14519.5.2.1.4591.4001.560732524941534483904820998846
TCGA-GBM	TCGA-06	TCGA-06-0649	flair	F			AX._FLAIR	501	2.28347E+15	1/26/2006		PMSN-AA8Y7H70NX	IR		5	11000	140	2800	42.58868	1	6	90		25	256	147	0.8984375\0.8984375	TCGA-06-0649/1.3.6.1.4.1.14519.5.2.1.4591.4001.111226195519163301116460123619/1.3.6.1.4.1.14519.5.2.1.4591.4001.118521252167755472165852492837	
TCGA-GBM	TCGA-06	TCGA-06-0649	t1Gd	F			T1_AX_POST	801	2.28347E+15	1/26/2006		PMSN-AA8Y7H70NX	SE		5	696.6778	15		42.58868	1	6	62		26	256	205	0.8984375\0.8984375	TCGA-06-0649/1.3.6.1.4.1.14519.5.2.1.4591.4001.111226195519163301116460123619/1.3.6.1.4.1.14519.5.2.1.4591.4001.111395755305019629134143006170	
TCGA-GBM	TCGA-06	TCGA-06-0649	t1	F			T1_AX_PRE	701	2.28347E+15	1/26/2006		PMSN-AA8Y7H70NX	SE		5	696.6778	15		42.58868	1	6	62		26	256	205	0.8984375\0.8984375	TCGA-06-0649/1.3.6.1.4.1.14519.5.2.1.4591.4001.111226195519163301116460123619/1.3.6.1.4.1.14519.5.2.1.4591.4001.253942008984398191974160718322	
TCGA-GBM	TCGA-06	TCGA-06-0649	t2	F			T2W_TSE	401	2.28347E+15	1/26/2006		PMSN-AA8Y7H70NX	SE		5	5780.362	110		42.58868	1	6	90		25	288	227	0.71875\0.71875	TCGA-06-0649/1.3.6.1.4.1.14519.5.2.1.4591.4001.111226195519163301116460123619/1.3.6.1.4.1.14519.5.2.1.4591.4001.310081522664139753162843331456	
TCGA-GBM	TCGA-06	TCGA-06-1084	flair	M			AXIAL_FLAIR	4	3.62419E+15	1/15/2007		MR01MROW	IR	2D	5	8002	152.768	2000	63.88585	1.5	5	90	0.0378	34	256	192	0.9375\0.9375	TCGA-06-1084/1.3.6.1.4.1.14519.5.2.1.4591.4001.205933064051641141719264624760/1.3.6.1.4.1.14519.5.2.1.4591.4001.997008093475972500685701576370	
TCGA-GBM	TCGA-06	TCGA-06-1084	t1Gd	M			AXIAL_T1_GD	8	3.62419E+15	1/15/2007		MR01MROW	SE	2D	5	516.664	14	0	63.88585	1.5	5	90	0.0440378	34	256	192	0.9375\0.9375	TCGA-06-1084/1.3.6.1.4.1.14519.5.2.1.4591.4001.205933064051641141719264624760/1.3.6.1.4.1.14519.5.2.1.4591.4001.184518206794525227519819333834	
TCGA-GBM	TCGA-06	TCGA-06-1084	t1	M			AXIAL_T1	6	3.62419E+15	1/15/2007		MR01MROW	SE	2D	5	516.664	14	0	63.88585	1.5	5	90	0.0440378	34	256	192	0.9375\0.9375	TCGA-06-1084/1.3.6.1.4.1.14519.5.2.1.4591.4001.205933064051641141719264624760/1.3.6.1.4.1.14519.5.2.1.4591.4001.253975845398997713297147176708	
TCGA-GBM	TCGA-06	TCGA-06-1084	t2	M			AXIAL_FSE	3	3.62419E+15	1/15/2007		MR01MROW	SE	2D	5	3750	23.688	0	63.88585	1.5	5	90	0.0966	68	256	192	0.9375\0.9375	TCGA-06-1084/1.3.6.1.4.1.14519.5.2.1.4591.4001.205933064051641141719264624760/1.3.6.1.4.1.14519.5.2.1.4591.4001.225846560104323611908626019440	
TCGA-GBM	TCGA-06	TCGA-06-1802	flair	M			Ax_T2_FLAIR	6	1.85764E+15	4/3/2007		MR3T		SE\IR	2D	5	9002	121.284	2250	127.7321	3	6	90	0.8068	25	320	224	0.4297\0.4297	TCGA-06-1802/1.3.6.1.4.1.14519.5.2.1.4591.4001.319548581969656999665412328642/1.3.6.1.4.1.14519.5.2.1.4591.4001.303785431491112534531563759163
TCGA-GBM	TCGA-06	TCGA-06-1802	t1Gd	M			AX_T1_POST_GD_FLAIR	8	1.85764E+15	4/3/2007		MR3T		SE\IR	2D	4	3050.6	6.772	1238	127.7321	3	5	90	1.8105	30	320	224	0.4297\0.4297	TCGA-06-1802/1.3.6.1.4.1.14519.5.2.1.4591.4001.319548581969656999665412328642/1.3.6.1.4.1.14519.5.2.1.4591.4001.215549370013921736472676782979
TCGA-GBM	TCGA-06	TCGA-06-1802	t1	M			AX_T1_pre_GD_FLAIR	7	1.85764E+15	4/3/2007		MR3T		SE\IR	2D	4	3050.6	6.4	1238	127.7321	3	5	90	1.8105	30	320	224	0.4297\0.4297	TCGA-06-1802/1.3.6.1.4.1.14519.5.2.1.4591.4001.319548581969656999665412328642/1.3.6.1.4.1.14519.5.2.1.4591.4001.165267478442345010741661718562
TCGA-GBM	TCGA-06	TCGA-06-1802	t2	M			AX_T2_FR-FSE	5	1.85764E+15	4/3/2007		MR3T		SE	2D	5	3400	110.032	0	127.7321	3	7.5	90	1.2567	20	352	224	0.4297\0.4297	TCGA-06-1802/1.3.6.1.4.1.14519.5.2.1.4591.4001.319548581969656999665412328642/1.3.6.1.4.1.14519.5.2.1.4591.4001.431304703040316559486127609898
TCGA-GBM	TCGA-06	TCGA-06-2570	flair	F	21	68.492	AXIAL_FLAIR	7	1.4621E+15	7/26/2007	GE MEDICAL SYSTEMS		SIGNA EXCITE	SE\IR	2D	2.5	10002	125.4	2250	127.7322	3	2.5	90	0.6048	67	320	224	0.4688\0.4688	TCGA-06-2570/1.3.6.1.4.1.14519.5.2.1.4591.4001.763554173270318063812534542847/1.3.6.1.4.1.14519.5.2.1.4591.4001.875590044906522068491547240162
TCGA-GBM	TCGA-06	TCGA-06-2570	t1Gd	F	21	68.492	AX_T1_POST_GD_FLAIR	11	1.4621E+15	7/26/2007	GE MEDICAL SYSTEMS		SIGNA EXCITE	SE\IR	2D	2.5	2970.47	6.356	1238	127.7322	3	2.5	90	1.6819	67	320	224	0.4688\0.4688	TCGA-06-2570/1.3.6.1.4.1.14519.5.2.1.4591.4001.763554173270318063812534542847/1.3.6.1.4.1.14519.5.2.1.4591.4001.851364973348227212233680415818
TCGA-GBM	TCGA-06	TCGA-06-2570	t1	F	21	68.492	AX_T1_pre_gd	8	1.4621E+15	7/26/2007	GE MEDICAL SYSTEMS		SIGNA EXCITE	SE\IR	2D	5	2970.61	6.356	1238	127.7322	3	5	90	1.5595	34	320	224	0.4688\0.4688	TCGA-06-2570/1.3.6.1.4.1.14519.5.2.1.4591.4001.763554173270318063812534542847/1.3.6.1.4.1.14519.5.2.1.4591.4001.334156106497189532473121151584
TCGA-GBM	TCGA-06	TCGA-06-2570	t2	F	21	68.492	AX_T2_FR-FSE_RF2_150	6	1.4621E+15	7/26/2007	GE MEDICAL SYSTEMS		SIGNA EXCITE	SE	2D	5	3000	103.464	0	127.7322	3	5	90	1.0316	34	320	224	0.4688\0.4688	TCGA-06-2570/1.3.6.1.4.1.14519.5.2.1.4591.4001.763554173270318063812534542847/1.3.6.1.4.1.14519.5.2.1.4591.4001.181186298778100327141079566062
TCGA-GBM	TCGA-06	TCGA-06-5408	flair	F			Ax_FLAIR_2.5mm_for_surgery	4	1.18161E+15	1/11/2008		MR01MROW	IR	2D	2.5	10004	149.544	2200	63.88579	1.5	2.5	90	0.034	67	256	160	0.9375\0.9375	TCGA-06-5408/1.3.6.1.4.1.14519.5.2.1.4591.4001.694644982891228681410637134135/1.3.6.1.4.1.14519.5.2.1.4591.4001.178021249403048866403790750450	
TCGA-GBM	TCGA-06	TCGA-06-5408	t1Gd	F			Ax_T1_2.5mm_for_surgery	10	1.18161E+15	1/11/2008		MR01MROW	SE	2D	2.5	500	13	0	63.88584	1.5	2.5	90	0.0512679	68	256	160	0.9375\0.9375	TCGA-06-5408/1.3.6.1.4.1.14519.5.2.1.4591.4001.694644982891228681410637134135/1.3.6.1.4.1.14519.5.2.1.4591.4001.255056887939741615994527174538	
TCGA-GBM	TCGA-06	TCGA-06-5408	t1	F			AXIAL_T1	7	1.18161E+15	1/11/2008		MR01MROW	SE	2D	5	500	14	0	63.8858	1.5	5	90	0.036189	36	256	192	0.9375\0.9375	TCGA-06-5408/1.3.6.1.4.1.14519.5.2.1.4591.4001.694644982891228681410637134135/1.3.6.1.4.1.14519.5.2.1.4591.4001.366203051096691083339875526418	
TCGA-GBM	TCGA-06	TCGA-06-5408	t2	F			AXIAL_FSE	3	1.18161E+15	1/11/2008		MR01MROW	SE	2D	5	3500	23.688	0	63.88579	1.5	5	90	0.1235	72	256	192	0.9375\0.9375	TCGA-06-5408/1.3.6.1.4.1.14519.5.2.1.4591.4001.694644982891228681410637134135/1.3.6.1.4.1.14519.5.2.1.4591.4001.190151680889708453331941210343	
TCGA-GBM	TCGA-06	TCGA-06-5412	flair	F			AXIAL_FLAIR	10	2.70336E+15	6/3/2008		MR3T		SE\IR	2D	2.5	10002	125.4	2250	127.7323	3	2.5	90	0.4689	73	320	224	0.4688\0.4688	TCGA-06-5412/1.3.6.1.4.1.14519.5.2.1.4591.4001.221248717333016631625848271272/1.3.6.1.4.1.14519.5.2.1.4591.4001.232178263135282100751854200786
TCGA-GBM	TCGA-06	TCGA-06-5412	t1Gd	F			AX_T1_POST_GD_FLAIR	14	2.70336E+15	6/3/2008		MR3T		SE\IR	2D	2.5	2969.99	6.356	1238	127.7323	3	2.5	90	1.5969	73	320	224	0.4688\0.4688	TCGA-06-5412/1.3.6.1.4.1.14519.5.2.1.4591.4001.221248717333016631625848271272/1.3.6.1.4.1.14519.5.2.1.4591.4001.164214036339812685287628785362
TCGA-GBM	TCGA-06	TCGA-06-5412	t1	F			AX_T1_pre_gd	11	2.70336E+15	6/3/2008		MR3T		SE\IR	2D	5	2039.94	6.356	883	127.7323	3	5	90	1.1943	38	320	224	0.4688\0.4688	TCGA-06-5412/1.3.6.1.4.1.14519.5.2.1.4591.4001.221248717333016631625848271272/1.3.6.1.4.1.14519.5.2.1.4591.4001.120669219115152282285142798139
TCGA-GBM	TCGA-06	TCGA-06-5412	t2	F			AX_T2_FR-FSE_RF2_150	7	2.70336E+15	6/3/2008		MR3T		SE	2D	5	4000	103.464	0	127.7323	3	5	90	0.7542	38	320	224	0.4688\0.4688	TCGA-06-5412/1.3.6.1.4.1.14519.5.2.1.4591.4001.221248717333016631625848271272/1.3.6.1.4.1.14519.5.2.1.4591.4001.142440088173859427055176239317
TCGA-GBM	TCGA-06	TCGA-06-5413	flair	M			AXIAL_FLAIR	6	7.1913E+15	6/17/2008		MR3T		SE\IR	2D	2.5	8002	125.4	2250	127.7322	3	2.5	90	1.1589	75	320	224	0.4688\0.4688	TCGA-06-5413/1.3.6.1.4.1.14519.5.2.1.4591.4001.397375653956580386291577018002/1.3.6.1.4.1.14519.5.2.1.4591.4001.300669427817208545563445520070
TCGA-GBM	TCGA-06	TCGA-06-5413	t1Gd	M			AX_T1_POST_GD_FLAIR	13	7.1913E+15	6/17/2008		MR3T		SE\IR	2D	2.5	3042.02	6.484	1238	127.7322	3	2.5	90	1.6205	75	320	224	0.4688\0.4688	TCGA-06-5413/1.3.6.1.4.1.14519.5.2.1.4591.4001.397375653956580386291577018002/1.3.6.1.4.1.14519.5.2.1.4591.4001.234074548712879476847342581821
TCGA-GBM	TCGA-06	TCGA-06-5413	t1	M			AX_T1_pre_gd	9	7.1913E+15	6/17/2008		MR3T		SE\IR	2D	5	2551.56	6.484	1022	127.7322	3	5	90	1.6746	38	320	224	0.4688\0.4688	TCGA-06-5413/1.3.6.1.4.1.14519.5.2.1.4591.4001.397375653956580386291577018002/1.3.6.1.4.1.14519.5.2.1.4591.4001.593318939393691383906509606881
TCGA-GBM	TCGA-06	TCGA-06-5413	t2	M			AX_T2_FR-FSE_RF2_150	8	7.1913E+15	6/17/2008		MR3T		SE	2D	5	4000	105.192	0	127.7322	3	5	90	1.9607	38	320	224	0.4688\0.4688	TCGA-06-5413/1.3.6.1.4.1.14519.5.2.1.4591.4001.397375653956580386291577018002/1.3.6.1.4.1.14519.5.2.1.4591.4001.248855779352869249994890192373
TCGA-GBM	TCGA-06	TCGA-06-5417	flair	F	45	47.627	AXIAL_FLAIR	5	3.24633E+15	9/3/2008	GE MEDICAL SYSTEMS		SIGNA EXCITE	SE\IR	2D	2.5	10002	128.7	2250	127.7323	3	2.5	90	0.5087	72	320	224	0.4688\0.4688	TCGA-06-5417/1.3.6.1.4.1.14519.5.2.1.4591.4001.304604545029494418165835320551/1.3.6.1.4.1.14519.5.2.1.4591.4001.290772413693145619532853352893
TCGA-GBM	TCGA-06	TCGA-06-5417	t1Gd	F	45	47.627	AX_T1_POST_GD_FLAIR	13	3.24633E+15	9/3/2008	GE MEDICAL SYSTEMS		SIGNA EXCITE	SE\IR	2D	2.5	3066.62	6.356	1238	127.7323	3	2.5	90	1.5316	76	320	224	0.4688\0.4688	TCGA-06-5417/1.3.6.1.4.1.14519.5.2.1.4591.4001.304604545029494418165835320551/1.3.6.1.4.1.14519.5.2.1.4591.4001.309906575449439613084040317395
TCGA-GBM	TCGA-06	TCGA-06-5417	t1	F	45	47.627	AX_T1_pre_gd	7	3.24633E+15	9/3/2008	GE MEDICAL SYSTEMS		SIGNA EXCITE	SE\IR	2D	5	2040.55	6.36	883	127.7323	3	5	90	1.1512	38	320	224	0.4688\0.4688	TCGA-06-5417/1.3.6.1.4.1.14519.5.2.1.4591.4001.304604545029494418165835320551/1.3.6.1.4.1.14519.5.2.1.4591.4001.742441362139497895209446188614
TCGA-GBM	TCGA-06	TCGA-06-5417	t2	F	45	47.627	AX_T2_FR-FSE_RF2_150	6	3.24633E+15	9/3/2008	GE MEDICAL SYSTEMS		SIGNA EXCITE	SE	2D	5	4000	105.732	0	127.7323	3	5	90	0.7272	38	320	224	0.4688\0.4688	TCGA-06-5417/1.3.6.1.4.1.14519.5.2.1.4591.4001.304604545029494418165835320551/1.3.6.1.4.1.14519.5.2.1.4591.4001.165675604340541610850133926501
TCGA-GBM	TCGA-06	TCGA-06-6389	flair	F	49	68.039	AXIAL_FLAIR	6	2.44088E+15	4/4/2009	GE MEDICAL SYSTEMS		SIGNA EXCITE	SE\IR	2D	2.5	10002	125.4	2250	127.7323	3	2.5	90	0.5315	78	320	224	0.4688\0.4688	TCGA-06-6389/1.3.6.1.4.1.14519.5.2.1.4591.4001.296868957331552138094328434431/1.3.6.1.4.1.14519.5.2.1.4591.4001.161665405486670689151197029575
TCGA-GBM	TCGA-06	TCGA-06-6389	t1Gd	F	49	68.039	COR__T1_POST_GD_FLAIR	12	2.44088E+15	4/4/2009	GE MEDICAL SYSTEMS		SIGNA EXCITE	SE\IR	2D	5	2907.36	6.356	1205	127.7323	3	5	90	1.0657	45	320	224	0.4688\0.4688	TCGA-06-6389/1.3.6.1.4.1.14519.5.2.1.4591.4001.296868957331552138094328434431/1.3.6.1.4.1.14519.5.2.1.4591.4001.272603426520903457320862363374
TCGA-GBM	TCGA-06	TCGA-06-6389	t1	F	49	68.039	AX_T1_pre_gd	8	2.44088E+15	4/4/2009	GE MEDICAL SYSTEMS		SIGNA EXCITE	SE\IR	2D	5	2105.28	6.356	883	127.7323	3	5	90	1.3805	39	320	224	0.4688\0.4688	TCGA-06-6389/1.3.6.1.4.1.14519.5.2.1.4591.4001.296868957331552138094328434431/1.3.6.1.4.1.14519.5.2.1.4591.4001.154154311262940377270912110183
TCGA-GBM	TCGA-06	TCGA-06-6389	t2	F	49	68.039	AX_T2_FR-FSE_RF2_150	7	2.44088E+15	4/4/2009	GE MEDICAL SYSTEMS		SIGNA EXCITE	SE	2D	5	4000	103.464	0	127.7323	3	5	90	0.8998	39	320	224	0.4688\0.4688	TCGA-06-6389/1.3.6.1.4.1.14519.5.2.1.4591.4001.296868957331552138094328434431/1.3.6.1.4.1.14519.5.2.1.4591.4001.141696374723260511363949850190
TCGA-GBM	TCGA-08	TCGA-08-0350	flair	M	32	95.254	FLAIR_AXIAL	6	1.74615E+15	12/15/1998	GE MEDICAL SYSTEMS	LPOCOC0	GENESIS_SIGNA	RM	2D	5	10002	142.5		63.87656	1.5	5	90	0.027114	29	256	192	0.8593750000\0.8593750000	TCGA-08-0350/1.3.6.1.4.1.14519.5.2.1.7695.4001.569320506186381800578120391569/1.3.6.1.4.1.14519.5.2.1.7695.4001.223790420624969264518080364078
TCGA-GBM	TCGA-08	TCGA-08-0350	t1Gd	M	32	95.254	3DSPGR_AXIAL	14	1.74615E+15	12/15/1998	GE MEDICAL SYSTEMS	LPOCOC0	GENESIS_SIGNA	GR	3D	1.5	34	3		63.87656	1.5	1.5	35	0.018941	124	256	192	0.9375000000\0.9375000000	TCGA-08-0350/1.3.6.1.4.1.14519.5.2.1.7695.4001.569320506186381800578120391569/1.3.6.1.4.1.14519.5.2.1.7695.4001.149026159959841415765042402601
TCGA-GBM	TCGA-08	TCGA-08-0350	t1	M	32	95.254	TI_SAG_!MR_BRAIN!_WHOLE_HEAD	1	1.74615E+15	12/15/1998	GE MEDICAL SYSTEMS	LPOCOC0	GENESIS_SIGNA	SE	2D	4	600	16		63.87656	1.5	6	90	0.043908	24	256	192	1.1718750000\1.1718750000	TCGA-08-0350/1.3.6.1.4.1.14519.5.2.1.7695.4001.569320506186381800578120391569/1.3.6.1.4.1.14519.5.2.1.7695.4001.187092894138919886916310352088
TCGA-GBM	TCGA-08	TCGA-08-0350	t2	M	32	95.254	3DFSE_AXIAL	5	1.74615E+15	12/15/1998	GE MEDICAL SYSTEMS	LPOCOC0	GENESIS_SIGNA	SE		1.5	3000	104.576		63.87656	1.5	1.5	90	0.072917	114	256	192	1.0156250000\1.0156250000	TCGA-08-0350/1.3.6.1.4.1.14519.5.2.1.7695.4001.569320506186381800578120391569/1.3.6.1.4.1.14519.5.2.1.7695.4001.173215282973968589273253996333
TCGA-GBM	TCGA-08	TCGA-08-0352	flair	M	79	90.718	AX_FLAIR	2	1.74615E+15	12/25/2000	GE MEDICAL SYSTEMS	GEMSLPMR	GENESIS_SIGNA	IR	2D	5	10002	127.5	2200	6.39E+08	1.5	5	90	0.024711	29	256	192	0.859375\0.859375	TCGA-08-0352/1.3.6.1.4.1.14519.5.2.1.7695.4001.163028456110647174493384721797/1.3.6.1.4.1.14519.5.2.1.7695.4001.976433586898626163749090098621
TCGA-GBM	TCGA-08	TCGA-08-0352	t1Gd	M	79	90.718	3D_SPGR_AX	5	1.74615E+15	12/25/2000	GE MEDICAL SYSTEMS	GEMSLPMR	GENESIS_SIGNA	GR	3D	1.5	34	3		6.39E+08	1.5	1.5	35	0.019095	124	256	192	1.015625\1.015625	TCGA-08-0352/1.3.6.1.4.1.14519.5.2.1.7695.4001.163028456110647174493384721797/1.3.6.1.4.1.14519.5.2.1.7695.4001.114566536453601371704250309307
TCGA-GBM	TCGA-08	TCGA-08-0352	t1	M	79	90.718	FSPGR_SAG	1	1.74615E+15	12/25/2000	GE MEDICAL SYSTEMS	GEMSLPMR	GENESIS_SIGNA	GR	2D	4	100	2.8		6.39E+08	1.5	4	70	0.00068	44	256	192	0.937500\0.937500	TCGA-08-0352/1.3.6.1.4.1.14519.5.2.1.7695.4001.163028456110647174493384721797/1.3.6.1.4.1.14519.5.2.1.7695.4001.273255727733399439043422333634
TCGA-GBM	TCGA-08	TCGA-08-0352	t2	M	79	90.718	3D_FSE_AX	3	1.74615E+15	12/25/2000	GE MEDICAL SYSTEMS	GEMSLPMR	GENESIS_SIGNA	SE	3D	1.5	4000	104.576		6.39E+08	1.5	1.5	90	0.055123	120	256	192	1.015625\1.015625	TCGA-08-0352/1.3.6.1.4.1.14519.5.2.1.7695.4001.163028456110647174493384721797/1.3.6.1.4.1.14519.5.2.1.7695.4001.208292216616720295976805439852
TCGA-GBM	TCGA-08	TCGA-08-0353	flair	M	58	71.668	AX_FLAIR	2	1.74615E+15	3/14/2001	GE MEDICAL SYSTEMS	GEMROW	GENESIS_SIGNA	IR	2D	5	10002	133	2200	6.39E+08	1.5	5	90	0.024837	28	256	192	0.937500\0.937500	TCGA-08-0353/1.3.6.1.4.1.14519.5.2.1.7695.4001.337574128368739942371163019020/1.3.6.1.4.1.14519.5.2.1.7695.4001.140753700428222158006731649445
TCGA-GBM	TCGA-08	TCGA-08-0353	t1Gd	M	58	71.668	3D_SPGR_AX	5	1.74615E+15	3/14/2001	GE MEDICAL SYSTEMS	GEMROW	GENESIS_SIGNA	GR	3D	1.5	34	3		6.39E+08	1.5	1.5	35	0.019829	124	256	192	1.015625\1.015625	TCGA-08-0353/1.3.6.1.4.1.14519.5.2.1.7695.4001.337574128368739942371163019020/1.3.6.1.4.1.14519.5.2.1.7695.4001.328234903870025738854180702672
TCGA-GBM	TCGA-08	TCGA-08-0353	t1	M	58	71.668	FSPGR_SAG	1	1.74615E+15	3/14/2001	GE MEDICAL SYSTEMS	GEMROW	GENESIS_SIGNA	GR	2D	4	100	2.1		6.39E+08	1.5	4	70	0.002756	44	256	192	0.937500\0.937500	TCGA-08-0353/1.3.6.1.4.1.14519.5.2.1.7695.4001.337574128368739942371163019020/1.3.6.1.4.1.14519.5.2.1.7695.4001.307993577811064417415017105143
TCGA-GBM	TCGA-08	TCGA-08-0353	t2	M	58	71.668	3D_FSE_AX	4	1.74615E+15	3/14/2001	GE MEDICAL SYSTEMS	GEMROW	GENESIS_SIGNA	SE	3D	1.5	2500	104.64		6.39E+08	1.5	1.5	90	0.091613	114	256	192	1.015625\1.015625	TCGA-08-0353/1.3.6.1.4.1.14519.5.2.1.7695.4001.337574128368739942371163019020/1.3.6.1.4.1.14519.5.2.1.7695.4001.196310385969371473051006799315
TCGA-GBM	TCGA-08	TCGA-08-0354	flair	F	52	84.822	AX_FLAIR	3	1.74615E+15	5/8/2001	GE MEDICAL SYSTEMS	GEMSLPMR	GENESIS_SIGNA	IR	2D	5	10002	142.5	2200	6.39E+08	1.5	5	90	0.024176	28	256	192	0.859375\0.859375	TCGA-08-0354/1.3.6.1.4.1.14519.5.2.1.7695.4001.193272565505328856496292965242/1.3.6.1.4.1.14519.5.2.1.7695.4001.323527541860368517646892071129
TCGA-GBM	TCGA-08	TCGA-08-0354	t1	F	52	84.822	FSPGR_SAG	2	1.74615E+15	5/8/2001	GE MEDICAL SYSTEMS	GEMSLPMR	GENESIS_SIGNA	GR	2D	4	100	2.8		6.39E+08	1.5	4	70	0.000687	44	256	192	0.937500\0.937500	TCGA-08-0354/1.3.6.1.4.1.14519.5.2.1.7695.4001.193272565505328856496292965242/1.3.6.1.4.1.14519.5.2.1.7695.4001.118503307861787885716018452534
TCGA-GBM	TCGA-08	TCGA-08-0354	t2	F	52	84.822	3D_FSE_AX	7	1.74615E+15	5/8/2001	GE MEDICAL SYSTEMS	GEMSLPMR	GENESIS_SIGNA	SE	3D	1.5	4000	104.016		6.39E+08	1.5	1.5	90	0.06129	126	256	192	1.015625\1.015625	TCGA-08-0354/1.3.6.1.4.1.14519.5.2.1.7695.4001.193272565505328856496292965242/1.3.6.1.4.1.14519.5.2.1.7695.4001.230224561061739836820209141244
TCGA-GBM	TCGA-08	TCGA-08-0355	flair	F	30	61.235	AX_FLAIR	4	1.74615E+15	6/6/2001	GE MEDICAL SYSTEMS	GEMSLPMR	GENESIS_SIGNA	IR	2D	5	8802	133	2200	6.39E+08	1.5	5	90	0.033078	31	256	192	0.859375\0.859375	TCGA-08-0355/1.3.6.1.4.1.14519.5.2.1.7695.4001.218361089000532962354851713589/1.3.6.1.4.1.14519.5.2.1.7695.4001.134415699017245527869615131670
TCGA-GBM	TCGA-08	TCGA-08-0355	t1Gd	F	30	61.235	3D_SPGR_AX	6	1.74615E+15	6/6/2001	GE MEDICAL SYSTEMS	GEMSLPMR	GENESIS_SIGNA	GR	3D	1.5	34	3		6.39E+08	1.5	1.5	35	0.020334	108	256	192	1.015625\1.015625	TCGA-08-0355/1.3.6.1.4.1.14519.5.2.1.7695.4001.218361089000532962354851713589/1.3.6.1.4.1.14519.5.2.1.7695.4001.337847097697226473402589529720
TCGA-GBM	TCGA-08	TCGA-08-0355	t1	F	30	61.235	FSPGR_SAG	2	1.74615E+15	6/6/2001	GE MEDICAL SYSTEMS	GEMSLPMR	GENESIS_SIGNA	GR	2D	4	100	2.8		6.39E+08	1.5	4	70	0.000724	44	256	192	0.937500\0.937500	TCGA-08-0355/1.3.6.1.4.1.14519.5.2.1.7695.4001.218361089000532962354851713589/1.3.6.1.4.1.14519.5.2.1.7695.4001.332032456864712781947929643599
TCGA-GBM	TCGA-08	TCGA-08-0355	t2	F	30	61.235	3D_FSE_AX	7	1.74615E+15	6/6/2001	GE MEDICAL SYSTEMS	GEMSLPMR	GENESIS_SIGNA	SE	3D	1.5	2600	104.576		6.39E+08	1.5	1.5	90	0.081299	108	256	192	1.015625\1.015625	TCGA-08-0355/1.3.6.1.4.1.14519.5.2.1.7695.4001.218361089000532962354851713589/1.3.6.1.4.1.14519.5.2.1.7695.4001.339057153668167454576759289870
TCGA-GBM	TCGA-08	TCGA-08-0356	flair	F	59	104.326	AX_FLAIR	3	1.74615E+15	3/26/2002	GE MEDICAL SYSTEMS	GEMROW	GENESIS_SIGNA	IR	2D	5	10002	133	2200	6.39E+08	1.5	5	90	0.02673	32	256	160	0.859375\0.859375	TCGA-08-0356/1.3.6.1.4.1.14519.5.2.1.7695.4001.232438853782668841142871631923/1.3.6.1.4.1.14519.5.2.1.7695.4001.145267262126068158967843466003
TCGA-GBM	TCGA-08	TCGA-08-0356	t1Gd	F	59	104.326	AX_3D_SPGR_GAD	7	1.74615E+15	3/26/2002	GE MEDICAL SYSTEMS	GEMROW	GENESIS_SIGNA	GR	3D	1.5	34	3		6.39E+08	1.5	1.5	35	0.018673	124	256	192	1.015625\1.015625	TCGA-08-0356/1.3.6.1.4.1.14519.5.2.1.7695.4001.232438853782668841142871631923/1.3.6.1.4.1.14519.5.2.1.7695.4001.242840321773074493339785317095
TCGA-GBM	TCGA-08	TCGA-08-0356	t1	F	59	104.326	SAG_FSPGR	2	1.74615E+15	3/26/2002	GE MEDICAL SYSTEMS	GEMROW	GENESIS_SIGNA	GR	2D	4	100	2.1		6.39E+08	1.5	4	70	0.002596	44	256	192	0.937500\0.937500	TCGA-08-0356/1.3.6.1.4.1.14519.5.2.1.7695.4001.232438853782668841142871631923/1.3.6.1.4.1.14519.5.2.1.7695.4001.185236563426956780421070538793
TCGA-GBM	TCGA-08	TCGA-08-0356	t2	F	59	104.326	AX_3D_FSE	4	1.74615E+15	3/26/2002	GE MEDICAL SYSTEMS	GEMROW	GENESIS_SIGNA	SE	3D	1.5	3000	104.64		6.39E+08	1.5	1.5	90	0.071883	120	256	192	1.015625\1.015625	TCGA-08-0356/1.3.6.1.4.1.14519.5.2.1.7695.4001.232438853782668841142871631923/1.3.6.1.4.1.14519.5.2.1.7695.4001.338312619633234820114774625995
TCGA-GBM	TCGA-08	TCGA-08-0357	flair	M	49	81.647	AX_FLAIR	2	1.74615E+15	5/28/2002	GE MEDICAL SYSTEMS	GEMSLPMR	GENESIS_SIGNA	IR	2D	5	10002	127.5	2200	6.39E+08	1.5	5	90	0.025131	32	256	160	0.937500\0.937500	TCGA-08-0357/1.3.6.1.4.1.14519.5.2.1.7695.4001.119967927917425608159550386921/1.3.6.1.4.1.14519.5.2.1.7695.4001.209481123456866732766059421408
TCGA-GBM	TCGA-08	TCGA-08-0357	t1Gd	M	49	81.647	3D_SPGR_AX	6	1.74615E+15	5/28/2002	GE MEDICAL SYSTEMS	GEMSLPMR	GENESIS_SIGNA	GR	3D	1.5	34	3		6.39E+08	1.5	1.5	35	0.01942	124	256	192	1.015625\1.015625	TCGA-08-0357/1.3.6.1.4.1.14519.5.2.1.7695.4001.119967927917425608159550386921/1.3.6.1.4.1.14519.5.2.1.7695.4001.262710894696531450868984668624
TCGA-GBM	TCGA-08	TCGA-08-0357	t1	M	49	81.647	FSPGR_SAG	1	1.74615E+15	5/28/2002	GE MEDICAL SYSTEMS	GEMSLPMR	GENESIS_SIGNA	GR	2D	4	100	2.8		6.39E+08	1.5	4	70	0.000691	44	256	160	0.937500\0.937500	TCGA-08-0357/1.3.6.1.4.1.14519.5.2.1.7695.4001.119967927917425608159550386921/1.3.6.1.4.1.14519.5.2.1.7695.4001.659866133278407752982263311318
TCGA-GBM	TCGA-08	TCGA-08-0357	t2	M	49	81.647	3D_FSE_AX	3	1.74615E+15	5/28/2002	GE MEDICAL SYSTEMS	GEMSLPMR	GENESIS_SIGNA	SE	3D	1.5	3000	104.576		6.39E+08	1.5	1.5	90	0.074759	120	256	160	1.015625\1.015625	TCGA-08-0357/1.3.6.1.4.1.14519.5.2.1.7695.4001.119967927917425608159550386921/1.3.6.1.4.1.14519.5.2.1.7695.4001.205237217403372024720024068239
TCGA-GBM	TCGA-08	TCGA-08-0358	flair	M	50	83.915	COR_FLAIR	5	1.74615E+15	11/26/2002	GE MEDICAL SYSTEMS	GEMSLPMR	GENESIS_SIGNA	IR	2D	5	10002	127.5	2200	6.39E+08	1.5	5	90	0.025453	32	256	192	0.859375\0.859375	TCGA-08-0358/1.3.6.1.4.1.14519.5.2.1.7695.4001.178258647502369298089351214604/1.3.6.1.4.1.14519.5.2.1.7695.4001.182758059237808096357467156444
TCGA-GBM	TCGA-08	TCGA-08-0358	t1Gd	M	50	83.915	3D_SPGR_AX	7	1.74615E+15	11/26/2002	GE MEDICAL SYSTEMS	GEMSLPMR	GENESIS_SIGNA	GR	3D	3	23	8		6.39E+08	1.5	3	35	0.005102	60	256	160	0.937500\0.937500	TCGA-08-0358/1.3.6.1.4.1.14519.5.2.1.7695.4001.178258647502369298089351214604/1.3.6.1.4.1.14519.5.2.1.7695.4001.216730440392063319195987347730
TCGA-GBM	TCGA-08	TCGA-08-0358	t1	M	50	83.915	AX_T1	2	1.74615E+15	11/26/2002	GE MEDICAL SYSTEMS	GEMSLPMR	GENESIS_SIGNA	SE	2D	5	600	9		6.39E+08	1.5	6	90	0.058411	24	256	192	0.859384\0.859375	TCGA-08-0358/1.3.6.1.4.1.14519.5.2.1.7695.4001.178258647502369298089351214604/1.3.6.1.4.1.14519.5.2.1.7695.4001.179655607006515166822372048602
TCGA-GBM	TCGA-08	TCGA-08-0358	t2	M	50	83.915	3D_FSE_AX	3	1.74615E+15	11/26/2002	GE MEDICAL SYSTEMS	GEMSLPMR	GENESIS_SIGNA	SE	3D	1.5	4000	104.576		6.39E+08	1.5	1.5	90	0.055123	114	256	192	1.015625\1.015625	TCGA-08-0358/1.3.6.1.4.1.14519.5.2.1.7695.4001.211723469240412331121762942910/1.3.6.1.4.1.14519.5.2.1.7695.4001.302868369039586931692020679777
TCGA-GBM	TCGA-08	TCGA-08-0359	flair	F	59	83.915	AX_FLAIR_gad	7	1.74615E+15	1/19/2003	GE MEDICAL SYSTEMS	GEMSLPMR	GENESIS_SIGNA	IR	2D	5	10002	142.5	2200	6.39E+08	1.5	5	90	0.024218	27	256	192	0.859375\0.859375	TCGA-08-0359/1.3.6.1.4.1.14519.5.2.1.7695.4001.291982097074076414376657462951/1.3.6.1.4.1.14519.5.2.1.7695.4001.720373715113146700092274252984
TCGA-GBM	TCGA-08	TCGA-08-0359	t1Gd	F	59	83.915	3D_SPGR_AX	6	1.74615E+15	1/19/2003	GE MEDICAL SYSTEMS	GEMSLPMR	GENESIS_SIGNA	GR	3D	1.5	34	3		6.39E+08	1.5	1.5	35	0.011393	102	256	192	1.015625\1.015625	TCGA-08-0359/1.3.6.1.4.1.14519.5.2.1.7695.4001.291982097074076414376657462951/1.3.6.1.4.1.14519.5.2.1.7695.4001.441692279775841498698035053980
TCGA-GBM	TCGA-08	TCGA-08-0359	t1	F	59	83.915	FSPGR_SAG	1	1.74615E+15	1/19/2003	GE MEDICAL SYSTEMS	GEMSLPMR	GENESIS_SIGNA	GR	2D	4	100	2.8		6.39E+08	1.5	4	70	0.000688	44	256	192	0.937500\0.937500	TCGA-08-0359/1.3.6.1.4.1.14519.5.2.1.7695.4001.291982097074076414376657462951/1.3.6.1.4.1.14519.5.2.1.7695.4001.303658910963991106533940013361
TCGA-GBM	TCGA-08	TCGA-08-0359	t2	F	59	83.915	3D_FSE_AX	2	1.74615E+15	1/19/2003	GE MEDICAL SYSTEMS	GEMSLPMR	GENESIS_SIGNA	SE	3D	1.5	4000	104.576		6.39E+08	1.5	1.5	90	0.050233	102	256	192	1.015625\1.015625	TCGA-08-0359/1.3.6.1.4.1.14519.5.2.1.7695.4001.291982097074076414376657462951/1.3.6.1.4.1.14519.5.2.1.7695.4001.147430811790810817730832339214
TCGA-GBM	TCGA-08	TCGA-08-0360	flair	M	76	72.575	AX_FLAIR_gad	7	1.74615E+15	2/23/2003	GE MEDICAL SYSTEMS	GEMSLPMR	GENESIS_SIGNA	IR	2D	5	10002	127.5	2200	6.39E+08	1.5	5	90	0.025609	32	256	192	0.859375\0.859375	TCGA-08-0360/1.3.6.1.4.1.14519.5.2.1.7695.4001.775866763600199916309126131064/1.3.6.1.4.1.14519.5.2.1.7695.4001.116744760614299221617250951944
TCGA-GBM	TCGA-08	TCGA-08-0360	t1Gd	M	76	72.575	3D_SPGR_AX	6	1.74615E+15	2/23/2003	GE MEDICAL SYSTEMS	GEMSLPMR	GENESIS_SIGNA	GR	3D	1.5	34	3		6.39E+08	1.5	1.5	35	0.011661	124	256	192	1.015625\1.015625	TCGA-08-0360/1.3.6.1.4.1.14519.5.2.1.7695.4001.775866763600199916309126131064/1.3.6.1.4.1.14519.5.2.1.7695.4001.318817306516501633455833057389
TCGA-GBM	TCGA-08	TCGA-08-0360	t1	M	76	72.575	FSPGR_SAG	1	1.74615E+15	2/23/2003	GE MEDICAL SYSTEMS	GEMSLPMR	GENESIS_SIGNA	GR	2D	4	100	2.8		6.39E+08	1.5	4	70	0.000704	44	256	192	0.937500\0.937500	TCGA-08-0360/1.3.6.1.4.1.14519.5.2.1.7695.4001.775866763600199916309126131064/1.3.6.1.4.1.14519.5.2.1.7695.4001.103650870268111201936685511415
TCGA-GBM	TCGA-08	TCGA-08-0360	t2	M	76	72.575	3D_FSE_AX	2	1.74615E+15	2/23/2003	GE MEDICAL SYSTEMS	GEMSLPMR	GENESIS_SIGNA	SE	3D	1.5	4000	104.576		6.39E+08	1.5	1.5	90	0.062839	126	256	192	1.015625\1.015625	TCGA-08-0360/1.3.6.1.4.1.14519.5.2.1.7695.4001.775866763600199916309126131064/1.3.6.1.4.1.14519.5.2.1.7695.4001.245474303741731760955757807517
TCGA-GBM	TCGA-08	TCGA-08-0385	flair	M	71	70.307	AX_FLAIR	2	1.74615E+15	8/27/2001	GE MEDICAL SYSTEMS	GEMSLPMR	GENESIS_SIGNA	IR	2D	5	10002	142.5	2200	6.39E+08	1.5	5	90	0.024913	28	256	192	0.859375\0.859375	TCGA-08-0385/1.3.6.1.4.1.14519.5.2.1.7695.4001.222434989025722161720779872375/1.3.6.1.4.1.14519.5.2.1.7695.4001.470684108406413400813519745909
TCGA-GBM	TCGA-08	TCGA-08-0385	t1Gd	M	71	70.307	3D_SPGR_AX	7	1.74615E+15	8/27/2001	GE MEDICAL SYSTEMS	GEMSLPMR	GENESIS_SIGNA	GR	3D	1.5	34	6		6.39E+08	1.5	1.5	35	0.01989	124	256	192	1.015625\1.015625	TCGA-08-0385/1.3.6.1.4.1.14519.5.2.1.7695.4001.222434989025722161720779872375/1.3.6.1.4.1.14519.5.2.1.7695.4001.721759255675381060893415325949
TCGA-GBM	TCGA-08	TCGA-08-0385	t1	M	71	70.307	FSPGR_SAG	1	1.74615E+15	8/27/2001	GE MEDICAL SYSTEMS	GEMSLPMR	GENESIS_SIGNA	GR	2D	4	100	2.8		6.39E+08	1.5	4	70	0.000708	44	256	192	0.937500\0.937500	TCGA-08-0385/1.3.6.1.4.1.14519.5.2.1.7695.4001.222434989025722161720779872375/1.3.6.1.4.1.14519.5.2.1.7695.4001.204026741851778126255510768761
TCGA-GBM	TCGA-08	TCGA-08-0385	t2	M	71	70.307	3D_FSE_AX	3	1.74615E+15	8/27/2001	GE MEDICAL SYSTEMS	GEMSLPMR	GENESIS_SIGNA	SE	3D	1.5	2500	104.576		6.39E+08	1.5	1.5	90	0.091895	120	256	192	1.015625\1.015625	TCGA-08-0385/1.3.6.1.4.1.14519.5.2.1.7695.4001.222434989025722161720779872375/1.3.6.1.4.1.14519.5.2.1.7695.4001.280478740522485950254533656856
TCGA-GBM	TCGA-08	TCGA-08-0389	flair	M	59	70.307	Ax_T2_FLAIR	5	1.74615E+15	7/25/2003	GE MEDICAL SYSTEMS	CBMR1	SIGNA EXCITE	IR	2D	5	10002	120.3	2200	63.82921	1.5	5	90	0.4477	32	256	224	0.429688\0.429688	TCGA-08-0389/1.3.6.1.4.1.14519.5.2.1.7695.4001.155576204150333185525274253431/1.3.6.1.4.1.14519.5.2.1.7695.4001.231537080539550629623423633749
TCGA-GBM	TCGA-08	TCGA-08-0389	t1Gd	M	59	70.307	3d_T1_FSPGR__CONT	10	1.74615E+15	7/25/2003	GE MEDICAL SYSTEMS	CBMR1	SIGNA EXCITE	GR	3D	1.5	34	3	0	63.82923	1.5	1.5	70	0.350961	124	256	192	1.01562\1.01562	TCGA-08-0389/1.3.6.1.4.1.14519.5.2.1.7695.4001.155576204150333185525274253431/1.3.6.1.4.1.14519.5.2.1.7695.4001.332152476495056790815972732567
TCGA-GBM	TCGA-08	TCGA-08-0389	t1	M	59	70.307	FSPGR_SAG	2	1.74615E+15	7/25/2003	GE MEDICAL SYSTEMS	CBMR1	SIGNA EXCITE	GR	2D	4	100	2.36	0	63.8292	1.5	4	70	0.192506	44	256	192	1.01562\1.01562	TCGA-08-0389/1.3.6.1.4.1.14519.5.2.1.7695.4001.155576204150333185525274253431/1.3.6.1.4.1.14519.5.2.1.7695.4001.265467282136232588736256233486
TCGA-GBM	TCGA-08	TCGA-08-0389	t2	M	59	70.307	3D_FSE_AXIAL	8	1.74615E+15	7/25/2003	GE MEDICAL SYSTEMS	CBMR1	SIGNA EXCITE	SE	3D	1.5	3000	104.256	0	63.82923	1.5	1.5	90	1.012569	114	256	160	1.01562\1.01562	TCGA-08-0389/1.3.6.1.4.1.14519.5.2.1.7695.4001.155576204150333185525274253431/1.3.6.1.4.1.14519.5.2.1.7695.4001.898120371300749852808668825214
TCGA-GBM	TCGA-08	TCGA-08-0390	flair	M	69	74.843	AX_FLAIR	5	1.74615E+15	8/5/2003	GE MEDICAL SYSTEMS	mrc2	SIGNA EXCITE	IR	2D	5	9302	140.116	2100	127.7146	3	5	90	0.8505	29	320	192	0.429688\0.429685	TCGA-08-0390/1.3.6.1.4.1.14519.5.2.1.7695.4001.162486595336728686386525070636/1.3.6.1.4.1.14519.5.2.1.7695.4001.215135936895486917436141586502
TCGA-GBM	TCGA-08	TCGA-08-0390	t1Gd	M	69	74.843	AX_SPGR_3D	8	1.74615E+15	8/5/2003	GE MEDICAL SYSTEMS	mrc2	SIGNA EXCITE	GR	3D	1.5	26	7	0	127.7147	3	1.5	35	0.805193	128	256	192	1.01562\1.01562	TCGA-08-0390/1.3.6.1.4.1.14519.5.2.1.7695.4001.162486595336728686386525070636/1.3.6.1.4.1.14519.5.2.1.7695.4001.339923650761117556933165423898
TCGA-GBM	TCGA-08	TCGA-08-0390	t1	M	69	74.843	FSPGR_SAG	2	1.74615E+15	8/5/2003	GE MEDICAL SYSTEMS	mrc2	SIGNA EXCITE	GR	2D	4	425	3.292	0	127.7147	3	4	90	2.345111	44	256	192	0.9375\0.9375	TCGA-08-0390/1.3.6.1.4.1.14519.5.2.1.7695.4001.162486595336728686386525070636/1.3.6.1.4.1.14519.5.2.1.7695.4001.128426618960163084409336878674
TCGA-GBM	TCGA-08	TCGA-08-0390	t2	M	69	74.843	3D_FSE_AX_20_slabs	6	1.74615E+15	8/5/2003	GE MEDICAL SYSTEMS	mrc2	SIGNA EXCITE	SE	3D	1.5	4300	99.288	0	127.7147	3	1.5	90	2.917361	120	256	256	1.01562\1.01562	TCGA-08-0390/1.3.6.1.4.1.14519.5.2.1.7695.4001.162486595336728686386525070636/1.3.6.1.4.1.14519.5.2.1.7695.4001.581616982218423289196570600960
TCGA-GBM	TCGA-08	TCGA-08-0392	flair	M	60	81.647	AX_FLAIR	4	1.74615E+15	11/3/2003	GE MEDICAL SYSTEMS	EXTERNAL_MR	GENESIS_SIGNA	IR	2D	5	8802	127.5	2200	6.39E+08	1.5	5	90	0.028558	30	256	160	0.859387\0.859375	TCGA-08-0392/1.3.6.1.4.1.14519.5.2.1.7695.4001.227489248220590980899973679112/1.3.6.1.4.1.14519.5.2.1.7695.4001.305330809372782012100149087676
TCGA-GBM	TCGA-08	TCGA-08-0392	t1Gd	M	60	81.647	3D_SPGR_AX	7	1.74615E+15	11/3/2003	GE MEDICAL SYSTEMS	EXTERNAL_MR	GENESIS_SIGNA	GR	3D	1.5	34	3		6.39E+08	1.5	1.5	35	0.01942	124	256	192	1.015625\1.015625	TCGA-08-0392/1.3.6.1.4.1.14519.5.2.1.7695.4001.227489248220590980899973679112/1.3.6.1.4.1.14519.5.2.1.7695.4001.325303380020247035105126562668
TCGA-GBM	TCGA-08	TCGA-08-0392	t1	M	60	81.647	FSPGR_SAG	2	1.74615E+15	11/3/2003	GE MEDICAL SYSTEMS	EXTERNAL_MR	GENESIS_SIGNA	GR	2D	4	100	2.8		6.39E+08	1.5	4	70	0.000691	44	256	192	0.937500\0.937500	TCGA-08-0392/1.3.6.1.4.1.14519.5.2.1.7695.4001.227489248220590980899973679112/1.3.6.1.4.1.14519.5.2.1.7695.4001.159334401813324113568299278427
TCGA-GBM	TCGA-08	TCGA-08-0392	t2	M	60	81.647	3D_FSE_AX	5	1.74615E+15	11/3/2003	GE MEDICAL SYSTEMS	EXTERNAL_MR	GENESIS_SIGNA	SE	3D	1.5	4000	104.576		6.39E+08	1.5	1.5	90	0.05606	120	256	192	1.015625\1.015625	TCGA-08-0392/1.3.6.1.4.1.14519.5.2.1.7695.4001.227489248220590980899973679112/1.3.6.1.4.1.14519.5.2.1.7695.4001.285943743580803495108812000858
TCGA-GBM	TCGA-08	TCGA-08-0509	flair	M	63	77.111	FLAIR_AXIAL	4	1.74615E+15	11/13/1997	GE MEDICAL SYSTEMS	LPOCOC0	GENESIS_SIGNA	RM	2D	5	10002	152		63.87615	1.5	5	90	0.028047	29	256	192	0.8593851328\0.8593750000	TCGA-08-0509/1.3.6.1.4.1.14519.5.2.1.7695.4001.292568156105181015740272479103/1.3.6.1.4.1.14519.5.2.1.7695.4001.408596602542998666261259876805
TCGA-GBM	TCGA-08	TCGA-08-0509	t1Gd	M	63	77.111	3DSPGR_AXIAL	2	1.74615E+15	11/13/1997	GE MEDICAL SYSTEMS	LPOCOC0	GENESIS_SIGNA	GR	3D	1.5	34	6		63.87616	1.5	1.5	35	0.019593	124	256	192	1.0156250000\1.0156250000	TCGA-08-0509/1.3.6.1.4.1.14519.5.2.1.7695.4001.292568156105181015740272479103/1.3.6.1.4.1.14519.5.2.1.7695.4001.258664400961230551340675293298
TCGA-GBM	TCGA-08	TCGA-08-0509	t1	M	63	77.111	FMPSPGR_SAG	1	1.74615E+15	11/13/1997	GE MEDICAL SYSTEMS	LPOCOC0	GENESIS_SIGNA	GR	2D	4	100	2.1		63.87618	1.5	4	70	0.017739	46	256	192	0.9375000000\0.9375000000	TCGA-08-0509/1.3.6.1.4.1.14519.5.2.1.7695.4001.292568156105181015740272479103/1.3.6.1.4.1.14519.5.2.1.7695.4001.224301481773938865793646319386
TCGA-GBM	TCGA-08	TCGA-08-0509	t2	M	63	77.111	3DFSE_AXIAL	3	1.74615E+15	11/13/1997	GE MEDICAL SYSTEMS	LPOCOC0	GENESIS_SIGNA	SE		1.5	2500	104.576		63.87616	1.5	1.5	90	0.090521	120	256	192	1.0156250000\1.0156250000	TCGA-08-0509/1.3.6.1.4.1.14519.5.2.1.7695.4001.292568156105181015740272479103/1.3.6.1.4.1.14519.5.2.1.7695.4001.178602050598278251326371799672
TCGA-GBM	TCGA-08	TCGA-08-0510	flair	M	75	92.986	AX_FLAIR	4	1.74615E+15	1/26/1998	GE MEDICAL SYSTEMS	GEMROW	GENESIS_SIGNA	IR	2D	5	10002	142.5	2200	6.39E+08	1.5	5	90	0.023893	28	256	192	0.859375\0.859375	TCGA-08-0510/1.3.6.1.4.1.14519.5.2.1.7695.4001.908705038093871340852313648625/1.3.6.1.4.1.14519.5.2.1.7695.4001.680572427201908205055469939074
TCGA-GBM	TCGA-08	TCGA-08-0510	t1Gd	M	75	92.986	3D_SPGR_AX	2	1.74615E+15	1/26/1998	GE MEDICAL SYSTEMS	GEMROW	GENESIS_SIGNA	GR	3D	1.5	34	3		6.39E+08	1.5	1.5	35	0.01902	124	256	192	1.015625\1.015625	TCGA-08-0510/1.3.6.1.4.1.14519.5.2.1.7695.4001.908705038093871340852313648625/1.3.6.1.4.1.14519.5.2.1.7695.4001.180669314170322596932458983139
TCGA-GBM	TCGA-08	TCGA-08-0510	t1	M	75	92.986	FSPGR_SAG	1	1.74615E+15	1/26/1998	GE MEDICAL SYSTEMS	GEMROW	GENESIS_SIGNA	GR	2D	4	100	2.1		6.39E+08	1.5	4	70	0.001722	44	256	192	0.937500\0.937500	TCGA-08-0510/1.3.6.1.4.1.14519.5.2.1.7695.4001.908705038093871340852313648625/1.3.6.1.4.1.14519.5.2.1.7695.4001.289052473786821559464871427227
TCGA-GBM	TCGA-08	TCGA-08-0510	t2	M	75	92.986	3D_FSE_AX	3	1.74615E+15	1/26/1998	GE MEDICAL SYSTEMS	GEMROW	GENESIS_SIGNA	SE	3D	1.5	4000	104.016		6.39E+08	1.5	1.5	90	0.054905	120	256	192	1.015625\1.015625	TCGA-08-0510/1.3.6.1.4.1.14519.5.2.1.7695.4001.908705038093871340852313648625/1.3.6.1.4.1.14519.5.2.1.7695.4001.208630687586140812333680115393
TCGA-GBM	TCGA-08	TCGA-08-0512	flair	M	48	72.575	FLAIR_AXIAL	3	1.74615E+15	5/25/1998	GE MEDICAL SYSTEMS	LPOCOC0	GENESIS_SIGNA	RM	2D	5	10002	142.5		63.87613	1.5	5	90	0.02832	29	256	192	0.8593750000\0.8593750000	TCGA-08-0512/1.3.6.1.4.1.14519.5.2.1.7695.4001.280248392879135411898507571335/1.3.6.1.4.1.14519.5.2.1.7695.4001.740352126423395607820113197890
TCGA-GBM	TCGA-08	TCGA-08-0512	t1Gd	M	48	72.575	3DSPGR_AXIAL	5	1.74615E+15	5/25/1998	GE MEDICAL SYSTEMS	LPOCOC0	GENESIS_SIGNA	GR	3D	1.5	34	6		63.87612	1.5	1.5	35	0.019784	124	256	192	1.0156250000\1.0156250000	TCGA-08-0512/1.3.6.1.4.1.14519.5.2.1.7695.4001.280248392879135411898507571335/1.3.6.1.4.1.14519.5.2.1.7695.4001.188649667958862219325502314719
TCGA-GBM	TCGA-08	TCGA-08-0512	t1	M	48	72.575	FMPSPGR_SAG	1	1.74615E+15	5/25/1998	GE MEDICAL SYSTEMS	LPOCOC0	GENESIS_SIGNA	GR	2D	4	100	2.1		63.87613	1.5	4	70	0.017912	46	256	192	0.9375000000\0.9375000000	TCGA-08-0512/1.3.6.1.4.1.14519.5.2.1.7695.4001.280248392879135411898507571335/1.3.6.1.4.1.14519.5.2.1.7695.4001.312239011718497662985901571869
TCGA-GBM	TCGA-08	TCGA-08-0512	t2	M	48	72.575	3DFSE_AXIAL	2	1.74615E+15	5/25/1998	GE MEDICAL SYSTEMS	LPOCOC0	GENESIS_SIGNA	SE		1.5	2700	104.576		63.87612	1.5	1.5	90	0.093091	126	256	192	1.0156250000\1.0156250000	TCGA-08-0512/1.3.6.1.4.1.14519.5.2.1.7695.4001.280248392879135411898507571335/1.3.6.1.4.1.14519.5.2.1.7695.4001.351239022505812855531771936817
TCGA-GBM	TCGA-08	TCGA-08-0520	flair	M	70	70.307	AX_FLAIR	5	1.74615E+15	10/13/1999	GE MEDICAL SYSTEMS	GEMSLPMR	GENESIS_SIGNA	IR	2D	5	10002	142.5	2200	6.39E+08	1.5	5	90	0.024913	28	256	192	0.859386\0.859375	TCGA-08-0520/1.3.6.1.4.1.14519.5.2.1.7695.4001.273090318153644004169216810536/1.3.6.1.4.1.14519.5.2.1.7695.4001.213779719506834152892047845101
TCGA-GBM	TCGA-08	TCGA-08-0520	t1Gd	M	70	70.307	3D_SPGR_AX	7	1.74615E+15	10/13/1999	GE MEDICAL SYSTEMS	GEMSLPMR	GENESIS_SIGNA	GR	3D	1.5	34	3		6.39E+08	1.5	1.5	35	0.01172	124	256	192	1.015625\1.015625	TCGA-08-0520/1.3.6.1.4.1.14519.5.2.1.7695.4001.273090318153644004169216810536/1.3.6.1.4.1.14519.5.2.1.7695.4001.231723835730198665428126824467
TCGA-GBM	TCGA-08	TCGA-08-0520	t1	M	70	70.307	FSPGR_SAG	1	1.74615E+15	10/13/1999	GE MEDICAL SYSTEMS	GEMSLPMR	GENESIS_SIGNA	GR	2D	4	100	2.9		6.39E+08	1.5	4	70	0.000708	44	256	192	0.937500\0.937500	TCGA-08-0520/1.3.6.1.4.1.14519.5.2.1.7695.4001.273090318153644004169216810536/1.3.6.1.4.1.14519.5.2.1.7695.4001.327168483589011701407590503204
TCGA-GBM	TCGA-08	TCGA-08-0520	t2	M	70	70.307	3D_FSE_AX	4	1.74615E+15	10/13/1999	GE MEDICAL SYSTEMS	GEMSLPMR	GENESIS_SIGNA	SE	3D	1.5	4000	104.016		6.39E+08	1.5	1.5	90	0.063159	126	256	192	1.015625\1.015625	TCGA-08-0520/1.3.6.1.4.1.14519.5.2.1.7695.4001.273090318153644004169216810536/1.3.6.1.4.1.14519.5.2.1.7695.4001.268232372267011104866408504758
TCGA-GBM	TCGA-08	TCGA-08-0521	flair	M	17	68.039	AX_FLAIR	2	1.74615E+15	3/7/2000	GE MEDICAL SYSTEMS	GEMSLPMR	GENESIS_SIGNA	IR	2D	5	10002	135	2200	6.39E+08	1.5	5	90	0.034659	28	256	192	0.859375\0.859375	TCGA-08-0521/1.3.6.1.4.1.14519.5.2.1.7695.4001.238470932121913921057408160948/1.3.6.1.4.1.14519.5.2.1.7695.4001.845104538012589376178146557772
TCGA-GBM	TCGA-08	TCGA-08-0521	t1Gd	M	17	68.039	3D_SPGR_AX	6	1.74615E+15	3/7/2000	GE MEDICAL SYSTEMS	GEMSLPMR	GENESIS_SIGNA	GR	3D	1.5	34	3		6.39E+08	1.5	1.5	35	0.011782	124	256	192	1.015625\1.015625	TCGA-08-0521/1.3.6.1.4.1.14519.5.2.1.7695.4001.238470932121913921057408160948/1.3.6.1.4.1.14519.5.2.1.7695.4001.110099365603373672025357679240
TCGA-GBM	TCGA-08	TCGA-08-0521	t1	M	17	68.039	FSPGR_SAG	1	1.74615E+15	3/7/2000	GE MEDICAL SYSTEMS	GEMSLPMR	GENESIS_SIGNA	GR	2D	4	100	2.9		6.39E+08	1.5	4	70	0.000712	44	256	192	0.937500\0.937500	TCGA-08-0521/1.3.6.1.4.1.14519.5.2.1.7695.4001.238470932121913921057408160948/1.3.6.1.4.1.14519.5.2.1.7695.4001.596008525691300018297895420363
TCGA-GBM	TCGA-08	TCGA-08-0521	t2	M	17	68.039	3D_FSE_AX	3	1.74615E+15	3/7/2000	GE MEDICAL SYSTEMS	GEMSLPMR	GENESIS_SIGNA	SE	3D	1.5	4000	104.016		6.39E+08	1.5	1.5	90	0.057719	114	256	192	1.015625\1.015625	TCGA-08-0521/1.3.6.1.4.1.14519.5.2.1.7695.4001.238470932121913921057408160948/1.3.6.1.4.1.14519.5.2.1.7695.4001.217015902186698075264229258207
TCGA-GBM	TCGA-08	TCGA-08-0522	flair	M	61	72.575	AX_FLAIR	9	1.74615E+15	4/24/2000	GE MEDICAL SYSTEMS	GEMSLPMR	GENESIS_SIGNA	IR	2D	5	10002	127.5	2200	6.39E+08	1.5	5	90	0.025609	30	256	192	0.859375\0.859375	TCGA-08-0522/1.3.6.1.4.1.14519.5.2.1.7695.4001.247226492578736624112472654446/1.3.6.1.4.1.14519.5.2.1.7695.4001.158696227902295886606720382429
TCGA-GBM	TCGA-08	TCGA-08-0522	t1Gd	M	61	72.575	3D_SPGR_AX	7	1.74615E+15	4/24/2000	GE MEDICAL SYSTEMS	GEMSLPMR	GENESIS_SIGNA	GR	3D	1.5	34	3		6.39E+08	1.5	1.5	35	0.019789	124	256	192	1.015625\1.015625	TCGA-08-0522/1.3.6.1.4.1.14519.5.2.1.7695.4001.247226492578736624112472654446/1.3.6.1.4.1.14519.5.2.1.7695.4001.168762703209156995755737783606
TCGA-GBM	TCGA-08	TCGA-08-0522	t1	M	61	72.575	FSPGR_SAG	3	1.74615E+15	4/24/2000	GE MEDICAL SYSTEMS	GEMSLPMR	GENESIS_SIGNA	GR	2D	4	100	2.9		6.39E+08	1.5	4	70	0.000704	44	256	192	0.937500\0.937500	TCGA-08-0522/1.3.6.1.4.1.14519.5.2.1.7695.4001.247226492578736624112472654446/1.3.6.1.4.1.14519.5.2.1.7695.4001.243308281114053949145480905289
TCGA-GBM	TCGA-08	TCGA-08-0522	t2	M	61	72.575	3D_FSE_AX	6	1.74615E+15	4/24/2000	GE MEDICAL SYSTEMS	GEMSLPMR	GENESIS_SIGNA	SE	3D	1.5	3000	104.016		6.39E+08	1.5	1.5	90	0.083799	126	256	192	1.015625\1.015625	TCGA-08-0522/1.3.6.1.4.1.14519.5.2.1.7695.4001.247226492578736624112472654446/1.3.6.1.4.1.14519.5.2.1.7695.4001.160182616952637474240510659084
TCGA-GBM	TCGA-08	TCGA-08-0524	flair	F	17	52.163	AX_FLAIR	2	1.74615E+15	11/2/2000	GE MEDICAL SYSTEMS	GEMSLPMR	GENESIS_SIGNA	IR	2D	3	10002	133	2200	6.39E+08	1.5	3	90	0.029865	32	256	192	0.859375\0.859375	TCGA-08-0524/1.3.6.1.4.1.14519.5.2.1.7695.4001.195614997200019607573614338362/1.3.6.1.4.1.14519.5.2.1.7695.4001.232360725935275321651480467041
TCGA-GBM	TCGA-08	TCGA-08-0524	t1Gd	F	17	52.163	3D_SPGR_AX	4	1.74615E+15	11/2/2000	GE MEDICAL SYSTEMS	GEMSLPMR	GENESIS_SIGNA	GR	3D	1.5	34	3		6.39E+08	1.5	1.5	35	0.020863	124	256	192	1.015625\1.015625	TCGA-08-0524/1.3.6.1.4.1.14519.5.2.1.7695.4001.195614997200019607573614338362/1.3.6.1.4.1.14519.5.2.1.7695.4001.267971913863007998166877158233
TCGA-GBM	TCGA-08	TCGA-08-0524	t1	F	17	52.163	FSPGR_SAG	1	1.74615E+15	11/2/2000	GE MEDICAL SYSTEMS	GEMSLPMR	GENESIS_SIGNA	GR	2D	4	100	2.8		6.39E+08	1.5	4	70	0.000743	44	256	192	0.937500\0.937500	TCGA-08-0524/1.3.6.1.4.1.14519.5.2.1.7695.4001.195614997200019607573614338362/1.3.6.1.4.1.14519.5.2.1.7695.4001.863645671849260537371170935073
TCGA-GBM	TCGA-08	TCGA-08-0524	t2	F	17	52.163	3D_FSE_AX	5	1.74615E+15	11/2/2000	GE MEDICAL SYSTEMS	GEMSLPMR	GENESIS_SIGNA	SE	3D	1.5	2600	104.576		6.39E+08	1.5	1.5	90	0.083412	108	256	192	1.015625\1.015625	TCGA-08-0524/1.3.6.1.4.1.14519.5.2.1.7695.4001.195614997200019607573614338362/1.3.6.1.4.1.14519.5.2.1.7695.4001.269722421607288207652501119281
TCGA-GBM	TCGA-08	TCGA-08-0529	flair	F	56	72.575	AX_FLAIR	3	1.74615E+15	10/29/2002	GE MEDICAL SYSTEMS	GEMSLPMR	GENESIS_SIGNA	IR	2D	5	10002	127.5	2200	6.39E+08	1.5	5	90	0.025609	32	256	192	0.859375\0.859375	TCGA-08-0529/1.3.6.1.4.1.14519.5.2.1.7695.4001.912696403696942091184547452317/1.3.6.1.4.1.14519.5.2.1.7695.4001.320850822961595333060405852108
TCGA-GBM	TCGA-08	TCGA-08-0529	t1Gd	F	56	72.575	3D_SPGR_AX	7	1.74615E+15	10/29/2002	GE MEDICAL SYSTEMS	GEMSLPMR	GENESIS_SIGNA	GR	3D	1.5	34	3		6.39E+08	1.5	1.5	35	0.019789	124	256	192	1.015625\1.015625	TCGA-08-0529/1.3.6.1.4.1.14519.5.2.1.7695.4001.912696403696942091184547452317/1.3.6.1.4.1.14519.5.2.1.7695.4001.220439982391729657710258636833
TCGA-GBM	TCGA-08	TCGA-08-0529	t1	F	56	72.575	FSPGR_SAG	2	1.74615E+15	10/29/2002	GE MEDICAL SYSTEMS	GEMSLPMR	GENESIS_SIGNA	GR	2D	4	100	2.8		6.39E+08	1.5	4	70	0.000704	42	256	192	0.937500\0.937500	TCGA-08-0529/1.3.6.1.4.1.14519.5.2.1.7695.4001.912696403696942091184547452317/1.3.6.1.4.1.14519.5.2.1.7695.4001.324296635305794383120984682544
TCGA-GBM	TCGA-08	TCGA-08-0529	t2	F	56	72.575	3D_FSE_AX	5	1.74615E+15	10/29/2002	GE MEDICAL SYSTEMS	GEMSLPMR	GENESIS_SIGNA	SE	3D	1.5	3000	104.576		6.39E+08	1.5	1.5	90	0.076181	120	256	160	1.015625\1.015625	TCGA-08-0529/1.3.6.1.4.1.14519.5.2.1.7695.4001.912696403696942091184547452317/1.3.6.1.4.1.14519.5.2.1.7695.4001.229810524792550999481504742060
TCGA-GBM	TCGA-12	TCGA-12-0616	flair	F	36	68.1	ax_flair	5	3.29916E+15	4/12/1999	SIEMENS		Trio	IR\SE	2D	5	9000	121	2200	123.1898	2.893620014	7.5	150	0.301020682	22	320	208	0.6875\0.6875	TCGA-12-0616/1.3.6.1.4.1.14519.5.2.1.8862.4001.266192358731001325275209303138/1.3.6.1.4.1.14519.5.2.1.8862.4001.169997737294570942795903209401
TCGA-GBM	TCGA-12	TCGA-12-0616	t1Gd	F	36	68.1	cor_mprage_+c	8	3.29916E+15	4/12/1999	SIEMENS		Trio	IR\GR	3D	1.25	2100	5.26	1100	123.1898	2.893620014		8	0.046533085	176	256	192	0.859375\0.859375	TCGA-12-0616/1.3.6.1.4.1.14519.5.2.1.8862.4001.266192358731001325275209303138/1.3.6.1.4.1.14519.5.2.1.8862.4001.238427576098066516919422281607
TCGA-GBM	TCGA-12	TCGA-12-0616	t1	F	36	68.1	ax_t1	2	3.29916E+15	4/12/1999	SIEMENS		Trio	SE	2D	5	800	10		123.1899	2.893620014	7.5	90	0.308275163	22	384	240	0.5729166665625\0.5729166665625	TCGA-12-0616/1.3.6.1.4.1.14519.5.2.1.8862.4001.266192358731001325275209303138/1.3.6.1.4.1.14519.5.2.1.8862.4001.724789870643862849694451333256
TCGA-GBM	TCGA-12	TCGA-12-0616	t2	F	36	68.1	ax_t2	3	3.29916E+15	4/12/1999	SIEMENS		Trio	SE	2D	5	3500	116		123.1899	2.893620014	7.5	180	0.255155891	22	512	312	0.4296875\0.4296875	TCGA-12-0616/1.3.6.1.4.1.14519.5.2.1.8862.4001.266192358731001325275209303138/1.3.6.1.4.1.14519.5.2.1.8862.4001.133973750207915213340858829260
TCGA-GBM	TCGA-12	TCGA-12-0776	flair	M	52	96.162	Ax_Flair_irFSE_H	3	2.8195E+15	5/12/1998	GE MEDICAL SYSTEMS		GENESIS_SIGNA	IR	2D	5	9002	147	2200	6.39E+08	1.5	6.5	90	0.025085	23	256	160	0.4296875\0.4296875	TCGA-12-0776/1.3.6.1.4.1.14519.5.2.1.8862.4001.252675253473152183963522222391/1.3.6.1.4.1.14519.5.2.1.8862.4001.546462055894409848329223976518
TCGA-GBM	TCGA-12	TCGA-12-0776	t1Gd	M	52	96.162	AX_T1_FSE_H+C	5	2.8195E+15	5/12/1998	GE MEDICAL SYSTEMS		GENESIS_SIGNA	RM	2D	5	500	11.12		6.39E+08	1.5	6.5	90	0.0451	23	320	160	0.4296875\0.4296875	TCGA-12-0776/1.3.6.1.4.1.14519.5.2.1.8862.4001.252675253473152183963522222391/1.3.6.1.4.1.14519.5.2.1.8862.4001.242852138496700175683090714079
TCGA-GBM	TCGA-12	TCGA-12-0776	t1	M	52	96.162	Sag_T1_FSE_S	1	2.8195E+15	5/12/1998	GE MEDICAL SYSTEMS		GENESIS_SIGNA	SE	2D	4	450	14		6.39E+08	1.5	5	90	0.036675	23	256	160	0.46875\0.46875	TCGA-12-0776/1.3.6.1.4.1.14519.5.2.1.8862.4001.252675253473152183963522222391/1.3.6.1.4.1.14519.5.2.1.8862.4001.269331403973802247544189874015
TCGA-GBM	TCGA-12	TCGA-12-0776	t2	M	52	96.162	Ax_T2_FSE_H	2	2.8195E+15	5/12/1998	GE MEDICAL SYSTEMS		GENESIS_SIGNA	RM	2D	5	4666.664	81.032		6.39E+08	1.5	6.5	90	0.1077	23	320	192	0.4296875\0.4296875	TCGA-12-0776/1.3.6.1.4.1.14519.5.2.1.8862.4001.252675253473152183963522222391/1.3.6.1.4.1.14519.5.2.1.8862.4001.204050589111893183636316823335
TCGA-GBM	TCGA-12	TCGA-12-0829	flair	M	75	90	FLAIR	4	2.8195E+15	6/2/1999	SIEMENS		Symphony	IR\SE	2D	5	8000	108	2500	63.66644	1.494	6.5	180	0.30549785	23	192	133	0.59895833\0.59895833	TCGA-12-0829/1.3.6.1.4.1.14519.5.2.1.8862.4001.161067531590129181313302509460/1.3.6.1.4.1.14519.5.2.1.8862.4001.256774824254590199629447782367
TCGA-GBM	TCGA-12	TCGA-12-0829	t1Gd	M	75	90	T1_AX_POST	18	2.8195E+15	6/2/1999	SIEMENS		Symphony	SE	2D	5	635	17		63.66644	1.494	6.5	75	0.73542476	23	256	144	0.44921875\0.44921875	TCGA-12-0829/1.3.6.1.4.1.14519.5.2.1.8862.4001.161067531590129181313302509460/1.3.6.1.4.1.14519.5.2.1.8862.4001.848905768294232154546172773832
TCGA-GBM	TCGA-12	TCGA-12-0829	t1	M	75	90	T1_AX_PRE	5	2.8195E+15	6/2/1999	SIEMENS		Symphony	SE	2D	5	571	17		63.66644	1.494	6.5	75	0.12039723	23	256	154	0.44921875\0.44921875	TCGA-12-0829/1.3.6.1.4.1.14519.5.2.1.8862.4001.161067531590129181313302509460/1.3.6.1.4.1.14519.5.2.1.8862.4001.229514096688570743417897123096
TCGA-GBM	TCGA-12	TCGA-12-0829	t2	M	75	90	T2_TSE_AX	3	2.8195E+15	6/2/1999	SIEMENS		Symphony	SE	2D	5	4670	104		63.6664	1.494	6.5	180	0.006011039	23	512	204	0.44921875\0.44921875	TCGA-12-0829/1.3.6.1.4.1.14519.5.2.1.8862.4001.161067531590129181313302509460/1.3.6.1.4.1.14519.5.2.1.8862.4001.126416306775413055857394841475
TCGA-GBM	TCGA-12	TCGA-12-1093	flair	F	66	54.431	AX_T2_FLAIR	4	2.8195E+15	9/20/1999	GE MEDICAL SYSTEMS		SIGNA EXCITE	IR	2D	5	9002	146.816	2250	63.86599	1.5	5	90	0.4465	30	256	192	0.859371\0.859374	TCGA-12-1093/1.3.6.1.4.1.14519.5.2.1.8862.4001.101131717694922416453780234026/1.3.6.1.4.1.14519.5.2.1.8862.4001.114806709082901981977289923516
TCGA-GBM	TCGA-12	TCGA-12-1093	t1Gd	F	66	54.431	AX_T1+C	9	2.8195E+15	9/20/1999	GE MEDICAL SYSTEMS		SIGNA EXCITE	SE	2D	5	466.664	20	0	63.86599	1.5	5	90	0.860357	30	288	192	0.429685\0.429687	TCGA-12-1093/1.3.6.1.4.1.14519.5.2.1.8862.4001.101131717694922416453780234026/1.3.6.1.4.1.14519.5.2.1.8862.4001.208421097810800556291170968762
TCGA-GBM	TCGA-12	TCGA-12-1093	t1	F	66	54.431	AX_T1	8	2.8195E+15	9/20/1999	GE MEDICAL SYSTEMS		SIGNA EXCITE	SE	2D	5	366.664	15	0	63.86599	1.5	5	90	1.09631	30	288	192	0.429685\0.429687	TCGA-12-1093/1.3.6.1.4.1.14519.5.2.1.8862.4001.101131717694922416453780234026/1.3.6.1.4.1.14519.5.2.1.8862.4001.850115408111720965337317146830
TCGA-GBM	TCGA-12	TCGA-12-1093	t2	F	66	54.431	AX_T2	7	2.8195E+15	9/20/1999	GE MEDICAL SYSTEMS		SIGNA EXCITE	SE	2D	5	4100	85.92	0	63.86599	1.5	5	90	1.3717	30	256	256	0.429685\0.429687	TCGA-12-1093/1.3.6.1.4.1.14519.5.2.1.8862.4001.101131717694922416453780234026/1.3.6.1.4.1.14519.5.2.1.8862.4001.201607716070807113655311579763
TCGA-GBM	TCGA-12	TCGA-12-1094	flair	M	56	136.078	COR_FLAIR	3	2.8195E+15	11/15/1999	GE MEDICAL SYSTEMS		GENESIS_SIGNA	IR	2D	5	8002	127.5	2000	6.39E+08	1.5	6.5	90	0.025767	27	256	192	0.937500\0.937500	TCGA-12-1094/1.3.6.1.4.1.14519.5.2.1.8862.4001.172549962021947577169833248421/1.3.6.1.4.1.14519.5.2.1.8862.4001.135805150196719046218951097371
TCGA-GBM	TCGA-12	TCGA-12-1094	t1Gd	M	56	136.078	POST_AX_T1	7	2.8195E+15	11/15/1999	GE MEDICAL SYSTEMS		GENESIS_SIGNA	SE	2D	5	550	9		6.39E+08	1.5	6.5	90	0.054078	22	256	224	0.937500\0.937500	TCGA-12-1094/1.3.6.1.4.1.14519.5.2.1.8862.4001.172549962021947577169833248421/1.3.6.1.4.1.14519.5.2.1.8862.4001.195674900023171610747750919749
TCGA-GBM	TCGA-12	TCGA-12-1094	t1	M	56	136.078	AX_T1	6	2.8195E+15	11/15/1999	GE MEDICAL SYSTEMS		GENESIS_SIGNA	SE	2D	5	550	9		6.39E+08	1.5	6.5	90	0.054078	22	256	224	0.937500\0.937500	TCGA-12-1094/1.3.6.1.4.1.14519.5.2.1.8862.4001.172549962021947577169833248421/1.3.6.1.4.1.14519.5.2.1.8862.4001.190799192727661447752457405978
TCGA-GBM	TCGA-12	TCGA-12-1094	t2	M	56	136.078	AX_T2_FS	5	2.8195E+15	11/15/1999	GE MEDICAL SYSTEMS		GENESIS_SIGNA	RM	2D	5	6650	81.928		6.39E+08	1.5	6.5	90	0.0395	22	512	224	0.468750\0.468750	TCGA-12-1094/1.3.6.1.4.1.14519.5.2.1.8862.4001.172549962021947577169833248421/1.3.6.1.4.1.14519.5.2.1.8862.4001.204812843794603036437575477292
TCGA-GBM	TCGA-12	TCGA-12-1098	flair	F	75	77.18	FLAIR_AXIAL	8	2.8195E+15	7/23/2000	SIEMENS		Avanto	IR\SE	2D	5	8630	110	2500	63.67312	1.493999958	6.25	150	0.111189924	23	256	179	0.44921875\0.44921875	TCGA-12-1098/1.3.6.1.4.1.14519.5.2.1.8862.4001.119873216352344754496678099313/1.3.6.1.4.1.14519.5.2.1.8862.4001.444903703803452795088981514764
TCGA-GBM	TCGA-12	TCGA-12-1098	t1Gd	F	75	77.18	T1_AXIAL__POST	10	2.8195E+15	7/23/2000	SIEMENS		Avanto	IR\GR	3D	2	1830	4.38	1100	63.67312	1.493999958		15	0.020332757	72	256	157	0.48828125\0.48828125	TCGA-12-1098/1.3.6.1.4.1.14519.5.2.1.8862.4001.119873216352344754496678099313/1.3.6.1.4.1.14519.5.2.1.8862.4001.278563943372409161004573397398
TCGA-GBM	TCGA-12	TCGA-12-1098	t1	F	75	77.18	T1_AXIAL	9	2.8195E+15	7/23/2000	SIEMENS		Avanto	IR\GR	3D	2	1830	4.38	1100	63.67312	1.493999958		15	0.020332757	72	256	157	0.48828125\0.48828125	TCGA-12-1098/1.3.6.1.4.1.14519.5.2.1.8862.4001.119873216352344754496678099313/1.3.6.1.4.1.14519.5.2.1.8862.4001.222247651601980577893548825337
TCGA-GBM	TCGA-12	TCGA-12-1098	t2	F	75	77.18	T2_TSE_AXIAL	6	2.8195E+15	7/23/2000	SIEMENS		Avanto	SE	2D	5	4390	109		63.67309	1.493999958	6.25	150	0.265220344	23	512	304	0.21502535927022\0.21502535927022	TCGA-12-1098/1.3.6.1.4.1.14519.5.2.1.8862.4001.119873216352344754496678099313/1.3.6.1.4.1.14519.5.2.1.8862.4001.266821148594345502971893058552
TCGA-GBM	TCGA-12	TCGA-12-1598	flair	F	75	81.72	FLAIR_AX	4	2.8195E+15	8/23/1999	SIEMENS		Symphony	IR\SE	2D	5	10000	106	2300	63.68462	1.494	6	180	0.00539074	20	256	179	0.8984375\0.8984375	TCGA-12-1598/1.3.6.1.4.1.14519.5.2.1.8862.4001.146632443531148261788047561414/1.3.6.1.4.1.14519.5.2.1.8862.4001.336849870613672922999907742707
TCGA-GBM	TCGA-12	TCGA-12-1598	t1Gd	F	75	81.72	T1_AX_gado	9	2.8195E+15	8/23/1999	SIEMENS		Symphony	SE	2D	5	552	17		63.68464	1.494	6.5	90	0.11230121	20	256	192	0.8984375\0.8984375	TCGA-12-1598/1.3.6.1.4.1.14519.5.2.1.8862.4001.146632443531148261788047561414/1.3.6.1.4.1.14519.5.2.1.8862.4001.342060257312097189842582111091
TCGA-GBM	TCGA-12	TCGA-12-1598	t1	F	75	81.72	T1_AX	5	2.8195E+15	8/23/1999	SIEMENS		Symphony	SE	2D	5	506	17		63.68464	1.494	6.5	90	0.18678181	20	256	192	0.8984375\0.8984375	TCGA-12-1598/1.3.6.1.4.1.14519.5.2.1.8862.4001.146632443531148261788047561414/1.3.6.1.4.1.14519.5.2.1.8862.4001.102361236469082315415067903736
TCGA-GBM	TCGA-12	TCGA-12-1598	t2	F	75	81.72	T2_AX	3	2.8195E+15	8/23/1999	SIEMENS		Symphony	SE	2D	5	5760	110		63.68462	1.494	6.5	180	0.005438163	20	512	288	0.44921875\0.44921875	TCGA-12-1598/1.3.6.1.4.1.14519.5.2.1.8862.4001.146632443531148261788047561414/1.3.6.1.4.1.14519.5.2.1.8862.4001.735255270119748511424115051454
TCGA-GBM	TCGA-12	TCGA-12-1601	flair	M	71	90.71849	ax_flair	5	1.76067E+15	6/19/2000	SIEMENS		TrioTim	SE\IR	2D	5	9000	134	2500	123.2367	3	7.5	125	0.695210424	24	320	320	0.71875\0.71875	TCGA-12-1601/1.3.6.1.4.1.14519.5.2.1.8862.4001.201486216522847049056957283859/1.3.6.1.4.1.14519.5.2.1.8862.4001.248879959554046988740819326552
TCGA-GBM	TCGA-12	TCGA-12-1601	t1Gd	M	71	90.71849	ax_t1_+c	9	1.76067E+15	6/19/2000	SIEMENS		TrioTim	SE	2D	5	720	20		123.2367	3	7.5	75	0.674986112	24	384	240	0.59895837306976\0.59895837306976	TCGA-12-1601/1.3.6.1.4.1.14519.5.2.1.8862.4001.201486216522847049056957283859/1.3.6.1.4.1.14519.5.2.1.8862.4001.146592301314373210626155028600
TCGA-GBM	TCGA-12	TCGA-12-1601	t1	M	71	90.71849	ax_t1	3	1.76067E+15	6/19/2000	SIEMENS		TrioTim	SE	2D	5	796	10		123.2367	3	7.5	75	0.694832124	24	320	192	0.71875\0.71875	TCGA-12-1601/1.3.6.1.4.1.14519.5.2.1.8862.4001.201486216522847049056957283859/1.3.6.1.4.1.14519.5.2.1.8862.4001.609705420088117660227141140219
TCGA-GBM	TCGA-12	TCGA-12-1601	t2	M	71	90.71849	ax_t2	4	1.76067E+15	6/19/2000	SIEMENS		TrioTim	SE	2D	5	4830	113		123.2367	3	7.5	140	0.695210424	24	320	320	0.71875\0.71875	TCGA-12-1601/1.3.6.1.4.1.14519.5.2.1.8862.4001.201486216522847049056957283859/1.3.6.1.4.1.14519.5.2.1.8862.4001.111322175120195590616905223490
TCGA-GBM	TCGA-12	TCGA-12-1602	flair	M	58	0	AX_FLAIR	7	2.8195E+15	3/4/2001	SIEMENS		Trio	IR\SE	2D	5	10000	115	2500	123.2288	3	6	180	0.156334221	24	256	208	0.41015625\0.41015625	TCGA-12-1602/1.3.6.1.4.1.14519.5.2.1.8862.4001.322746683938440731450945038341/1.3.6.1.4.1.14519.5.2.1.8862.4001.239852680542995747210886629062
TCGA-GBM	TCGA-12	TCGA-12-1602	t1Gd	M	58	0	T1_POST_GD	19	2.8195E+15	3/4/2001	SIEMENS		Trio	SE	2D	5	720	20		123.2289	3	6	60	0.161792412	24	256	179	0.8203125\0.8203125	TCGA-12-1602/1.3.6.1.4.1.14519.5.2.1.8862.4001.322746683938440731450945038341/1.3.6.1.4.1.14519.5.2.1.8862.4001.325175595450208505943785543644
TCGA-GBM	TCGA-12	TCGA-12-1602	t1	M	58	0	AX_SE_T1	6	2.8195E+15	3/4/2001	SIEMENS		Trio	SE	2D	5	678	17		123.2289	3	6	60	0.159116566	24	256	224	0.8203125\0.8203125	TCGA-12-1602/1.3.6.1.4.1.14519.5.2.1.8862.4001.322746683938440731450945038341/1.3.6.1.4.1.14519.5.2.1.8862.4001.504967401532712438614608840804
TCGA-GBM	TCGA-12	TCGA-12-1602	t2	M	58	0	SAG_TSE3D_T2	10	2.8195E+15	3/4/2001	SIEMENS		Trio	SE	3D	1.2	3000	355		123.2289	3		180	0.127274916	144	256	192	1\1	TCGA-12-1602/1.3.6.1.4.1.14519.5.2.1.8862.4001.322746683938440731450945038341/1.3.6.1.4.1.14519.5.2.1.8862.4001.955499093208862961641223883317
TCGA-GBM	TCGA-12	TCGA-12-3650	flair	M	46	102.058	Ax_T2Flair	4	2.8195E+15	7/29/2001	GE MEDICAL SYSTEMS		SIGNA HDx	SE\\\\	2D	5	8000	135.648	2000	63.82825	1.5	6	90	0.846887	25	288	288	0.468750\0.468750	TCGA-12-3650/1.3.6.1.4.1.14519.5.2.1.8862.4001.853973730099261399255070895330/1.3.6.1.4.1.14519.5.2.1.8862.4001.239036983327341632160933559587
TCGA-GBM	TCGA-12	TCGA-12-3650	t1Gd	M	46	102.058	AX_T1_POST	9	2.8195E+15	7/29/2001	GE MEDICAL SYSTEMS		SIGNA HDx	SE\\\\	2D	5	750	10.344	0	63.82825	1.5	6	90	1.5028	25	320	224	0.4688\0.4688	TCGA-12-3650/1.3.6.1.4.1.14519.5.2.1.8862.4001.853973730099261399255070895330/1.3.6.1.4.1.14519.5.2.1.8862.4001.259092977773592001212080458593
TCGA-GBM	TCGA-12	TCGA-12-3650	t1	M	46	102.058	AX_T1	6	2.8195E+15	7/29/2001	GE MEDICAL SYSTEMS		SIGNA HDx	SE\\\\	2D	5	750	10.344	0	63.82825	1.5	6	90	1.5028	25	320	224	0.4688\0.4688	TCGA-12-3650/1.3.6.1.4.1.14519.5.2.1.8862.4001.853973730099261399255070895330/1.3.6.1.4.1.14519.5.2.1.8862.4001.298865676266232133850135994761
TCGA-GBM	TCGA-12	TCGA-12-3650	t2	M	46	102.058	Ax_T2	5	2.8195E+15	7/29/2001	GE MEDICAL SYSTEMS		SIGNA HDx	SE\\\\	2D	5	6000	115.36		63.82825	1.5	6	90	1.553567	25	416	416	0.468750\0.468750	TCGA-12-3650/1.3.6.1.4.1.14519.5.2.1.8862.4001.853973730099261399255070895330/1.3.6.1.4.1.14519.5.2.1.8862.4001.250106226507995703345955579876
TCGA-GBM	TCGA-14	TCGA-14-0789	flair	M		80	BRAIN______FLAIR	301	1.3568E+15	11/19/1997	Philips Medical Systems	Gyro01	Intera	IR	2D	5	11000	140	2600	63.895	1.5	6	90		25	256	151	004.492188e-01\004.492188e-01	TCGA-14-0789/1.3.6.1.4.1.14519.5.2.1.2783.4001.184309347042506581266884481594/1.3.6.1.4.1.14519.5.2.1.2783.4001.801520796772002669204007145386
TCGA-GBM	TCGA-14	TCGA-14-0789	t1Gd	M		80	BRAIN______T1_AX_POST	801	1.3568E+15	11/19/1997	Philips Medical Systems	Gyro01	Intera	SE	2D	5	565	12		63.895	1.5	6	90		25	256	205	004.492188e-01\004.492188e-01	TCGA-14-0789/1.3.6.1.4.1.14519.5.2.1.2783.4001.184309347042506581266884481594/1.3.6.1.4.1.14519.5.2.1.2783.4001.890592197338541936330714143214
TCGA-GBM	TCGA-14	TCGA-14-0789	t1	M		80	BRAIN______T1_AX_PRE	701	1.3568E+15	11/19/1997	Philips Medical Systems	Gyro01	Intera	SE	2D	5	565	12		63.895	1.5	6	90		25	256	205	004.492188e-01\004.492188e-01	TCGA-14-0789/1.3.6.1.4.1.14519.5.2.1.2783.4001.184309347042506581266884481594/1.3.6.1.4.1.14519.5.2.1.2783.4001.233041259508543870852056565003
TCGA-GBM	TCGA-14	TCGA-14-0789	t2	M		80	BRAIN______T2_GRASE	401	1.3568E+15	11/19/1997	Philips Medical Systems	Gyro01	Intera	SE	2D	5	5000	100		63.895	1.5	6	90		25	256	241	004.492188e-01\004.492188e-01	TCGA-14-0789/1.3.6.1.4.1.14519.5.2.1.2783.4001.184309347042506581266884481594/1.3.6.1.4.1.14519.5.2.1.2783.4001.141146456081128930177162025757
TCGA-GBM	TCGA-14	TCGA-14-1456	flair	M		80	STROKE_____FLAIR	401	1.34201E+15	4/22/1999	Philips Medical Systems	Gyro01	Intera	IR	2D	5	11000	140	2600	63.90691	1.5	5	90		25	256	159	009.375000e-01\009.375000e-01	TCGA-14-1456/1.3.6.1.4.1.14519.5.2.1.2783.4001.250594672846486970701098971076/1.3.6.1.4.1.14519.5.2.1.2783.4001.321134067484147578724672344840
TCGA-GBM	TCGA-14	TCGA-14-1456	t1Gd	M		80	BRAIN______T1_AX_POST	1901	1.34201E+15	4/22/1999	Philips Medical Systems	Gyro01	Intera	SE	2D	5	565	12		63.90692	1.5	6	90		25	256	205	004.492188e-01\004.492188e-01	TCGA-14-1456/1.3.6.1.4.1.14519.5.2.1.2783.4001.250594672846486970701098971076/1.3.6.1.4.1.14519.5.2.1.2783.4001.784458997482067360267090300516
TCGA-GBM	TCGA-14	TCGA-14-1456	t1	M		80	Brain______T1W_SE	201	1.34201E+15	4/22/1999	Philips Medical Systems	Gyro01	Intera	SE	2D	5	352.1115	15		63.90692	1.5	5	69		25	256	141	009.375000e-01\009.375000e-01	TCGA-14-1456/1.3.6.1.4.1.14519.5.2.1.2783.4001.250594672846486970701098971076/1.3.6.1.4.1.14519.5.2.1.2783.4001.925674708608577414119113282332
TCGA-GBM	TCGA-14	TCGA-14-1456	t2	M		80	SEIZURE____T2_GRASE	301	1.34201E+15	4/22/1999	Philips Medical Systems	Gyro01	Intera	SE	2D	5	5000	100		63.90692	1.5	5	90		25	256	216	009.375000e-01\009.375000e-01	TCGA-14-1456/1.3.6.1.4.1.14519.5.2.1.2783.4001.250594672846486970701098971076/1.3.6.1.4.1.14519.5.2.1.2783.4001.139583367552275817017048428824
TCGA-GBM	TCGA-14	TCGA-14-1794	flair	M		90	BRAIN______FLAIR	301	2.13909E+15	4/11/1998	Philips Medical Systems	Gyro01	Intera	IR	2D	5	11000	140	2600	63.89496	1.5	6	90		25	256	151	004.492188e-01\004.492188e-01	TCGA-14-1794/1.3.6.1.4.1.14519.5.2.1.2783.4001.867649433395377496837912142545/1.3.6.1.4.1.14519.5.2.1.2783.4001.655280789746543642623011826556
TCGA-GBM	TCGA-14	TCGA-14-1794	t1Gd	M		90	BRAIN______T1_AX_POST	1101	2.13909E+15	4/11/1998	Philips Medical Systems	Gyro01	Intera	SE	2D	5	565	12		63.89496	1.5	6	90		25	256	205	004.492188e-01\004.492188e-01	TCGA-14-1794/1.3.6.1.4.1.14519.5.2.1.2783.4001.867649433395377496837912142545/1.3.6.1.4.1.14519.5.2.1.2783.4001.121223650477077769385830996743
TCGA-GBM	TCGA-14	TCGA-14-1794	t1	M		90	BRAIN______T1_AX_PRE	701	2.13909E+15	4/11/1998	Philips Medical Systems	Gyro01	Intera	SE	2D	5	565	12		63.89496	1.5	6	90		25	256	205	004.492188e-01\004.492188e-01	TCGA-14-1794/1.3.6.1.4.1.14519.5.2.1.2783.4001.867649433395377496837912142545/1.3.6.1.4.1.14519.5.2.1.2783.4001.101882499143559305814061553019
TCGA-GBM	TCGA-14	TCGA-14-1794	t2	M		90	BRAIN______T2_GRASE	401	2.13909E+15	4/11/1998	Philips Medical Systems	Gyro01	Intera	SE	2D	5	5000	100		63.89496	1.5	6	90		25	256	241	004.492188e-01\004.492188e-01	TCGA-14-1794/1.3.6.1.4.1.14519.5.2.1.2783.4001.867649433395377496837912142545/1.3.6.1.4.1.14519.5.2.1.2783.4001.466914301691885355538840307808
TCGA-GBM	TCGA-14	TCGA-14-1825	flair	M	70	76.65712	t2_flair_ax	3	1.4078E+15	2/10/2000	SIEMENS	MEDPC	Avanto	SE\IR	2D	5	8000	74	2500	63.67925	1.5	6	180	0.278502221	27	256	256	0.44921875\0.44921875	TCGA-14-1825/1.3.6.1.4.1.14519.5.2.1.2783.4001.303234879376974091026102189271/1.3.6.1.4.1.14519.5.2.1.2783.4001.277699748513063132657345876103
TCGA-GBM	TCGA-14	TCGA-14-1825	t1Gd	M	70	76.65712	t1_tra_post	10	1.4078E+15	2/10/2000	SIEMENS	MEDPC	Avanto	SE	2D	5	430	17		63.67925	1.5	6	90	0.299730615	27	320	144	0.71875\0.71875	TCGA-14-1825/1.3.6.1.4.1.14519.5.2.1.2783.4001.303234879376974091026102189271/1.3.6.1.4.1.14519.5.2.1.2783.4001.258939865753180397841218680382
TCGA-GBM	TCGA-14	TCGA-14-1825	t1	M	70	76.65712	t1_tra_pre	7	1.4078E+15	2/10/2000	SIEMENS	MEDPC	Avanto	SE	2D	5	430	17		63.67925	1.5	6	90	0.299730615	27	320	144	0.71875\0.71875	TCGA-14-1825/1.3.6.1.4.1.14519.5.2.1.2783.4001.303234879376974091026102189271/1.3.6.1.4.1.14519.5.2.1.2783.4001.208384222600208194575599573994
TCGA-GBM	TCGA-14	TCGA-14-1825	t2	M	70	76.65712	T2_AX_FS_post	8	1.4078E+15	2/10/2000	SIEMENS	MEDPC	Avanto	SE	2D	5	6070	110		63.67922	1.5	6	180	0.255179287	27	512	318	0.44921875\0.44921875	TCGA-14-1825/1.3.6.1.4.1.14519.5.2.1.2783.4001.303234879376974091026102189271/1.3.6.1.4.1.14519.5.2.1.2783.4001.642315295692187367949394853394
TCGA-GBM	TCGA-14	TCGA-14-1829	flair	M	57	86.18256	t2_flair_ax	3	1.63624E+15	6/14/2001	SIEMENS	EUHMR2	Avanto	SE\IR	2D	5	10000	130	2700	63.67914	1.5	6	180	0.317840978	27	256	172	0.44921875\0.44921875	TCGA-14-1829/1.3.6.1.4.1.14519.5.2.1.2783.4001.103854061130261737009165000972/1.3.6.1.4.1.14519.5.2.1.2783.4001.501460447631638541537723941093
TCGA-GBM	TCGA-14	TCGA-14-1829	t1Gd	M	57	86.18256	T1_3D_FFE	9	1.63624E+15	6/14/2001	SIEMENS	EUHMR2	Avanto	GR	3D	1.5	25	9		63.67914	1.5		35	0.100076854	120	256	256	0.9375\0.9375	TCGA-14-1829/1.3.6.1.4.1.14519.5.2.1.2783.4001.103854061130261737009165000972/1.3.6.1.4.1.14519.5.2.1.2783.4001.216813657680753995205839488022
TCGA-GBM	TCGA-14	TCGA-14-1829	t1	M	57	86.18256	t1_tra_pre_FIL	7	1.63624E+15	6/14/2001	SIEMENS	EUHMR2	Avanto	SE	2D	5	454	17		63.67914	1.5	6	76	0.728576006	27	320	144	0.71875\0.71875	TCGA-14-1829/1.3.6.1.4.1.14519.5.2.1.2783.4001.103854061130261737009165000972/1.3.6.1.4.1.14519.5.2.1.2783.4001.281678003339795573732020134118
TCGA-GBM	TCGA-14	TCGA-14-1829	t2	M	57	86.18256	T2_AX_FS	8	1.63624E+15	6/14/2001	SIEMENS	EUHMR2	Avanto	SE	2D	5	4450	110		63.6791	1.5	6	180	0.608993381	27	512	318	0.44921875\0.44921875	TCGA-14-1829/1.3.6.1.4.1.14519.5.2.1.2783.4001.103854061130261737009165000972/1.3.6.1.4.1.14519.5.2.1.2783.4001.375367856236772946053988995526
TCGA-GBM	TCGA-14	TCGA-14-3477	flair	F	38	58.96702	t2_flair_ax	7	2.67067E+15	5/1/2002	SIEMENS	MEDPC	TrioTim	SE\IR	2D	5	8000	91	2371.5	123.2522	3	6	130	0.353191561	25	256	205	0.44921875\0.44921875	TCGA-14-3477/1.3.6.1.4.1.14519.5.2.1.2783.4001.123124691157725520037267500618/1.3.6.1.4.1.14519.5.2.1.2783.4001.477389024768558126793730754601
TCGA-GBM	TCGA-14	TCGA-14-3477	t1Gd	F	38	58.96702	t1_ax_post	15	2.67067E+15	5/1/2002	SIEMENS	MEDPC	TrioTim	SE	2D	5	500	8.5		123.2523	3	6	70	0.225981221	25	256	180	0.44921875\0.44921875	TCGA-14-3477/1.3.6.1.4.1.14519.5.2.1.2783.4001.123124691157725520037267500618/1.3.6.1.4.1.14519.5.2.1.2783.4001.323059567715228362108477826515
TCGA-GBM	TCGA-14	TCGA-14-3477	t1	F	38	58.96702	t1_ax_pre	9	2.67067E+15	5/1/2002	SIEMENS	MEDPC	TrioTim	SE	2D	5	600	8.5		123.2523	3	6	70	0.326445016	25	256	218	0.44921875\0.44921875	TCGA-14-3477/1.3.6.1.4.1.14519.5.2.1.2783.4001.123124691157725520037267500618/1.3.6.1.4.1.14519.5.2.1.2783.4001.260352956461789136764709992439
TCGA-GBM	TCGA-14	TCGA-14-3477	t2	F	38	58.96702	t2_fs_ax	10	2.67067E+15	5/1/2002	SIEMENS	MEDPC	TrioTim	SE	2D	5	6000	93		123.2522	3	6	120	0.479688506	25	320	320	0.359375\0.359375	TCGA-14-3477/1.3.6.1.4.1.14519.5.2.1.2783.4001.123124691157725520037267500618/1.3.6.1.4.1.14519.5.2.1.2783.4001.126087670895542739221764627538
TCGA-GBM	TCGA-19	TCGA-19-0963	flair	M	61	77.18	MRHG_FLAIR_AX	3	1.06342E+15	10/12/2001	SIEMENS		Symphony	IR\SE	2D	5	9000	129	2500	63.65864	1.5	7.5	150	0.062243182	20	256	224	0.8984375\0.8984375	TCGA-19-0963/1.3.6.1.4.1.14519.5.2.1.5826.4001.182689849979752622358485655607/1.3.6.1.4.1.14519.5.2.1.5826.4001.414254955526029720850488987168
TCGA-GBM	TCGA-19	TCGA-19-0963	t1Gd	M	61	77.18	MRHG_T1_AX_POST_GAD	7	1.06342E+15	10/12/2001	SIEMENS		Symphony	SE	2D	5	745	17		63.65864	1.5	7.5	90	0.293716699	20	256	224	0.8984375\0.8984375	TCGA-19-0963/1.3.6.1.4.1.14519.5.2.1.5826.4001.182689849979752622358485655607/1.3.6.1.4.1.14519.5.2.1.5826.4001.203638647034312656830437984768
TCGA-GBM	TCGA-19	TCGA-19-0963	t1	M	61	77.18	MRHG_T1_AX	6	1.06342E+15	10/12/2001	SIEMENS		Symphony	SE	2D	5	587	15		63.65864	1.5	7.5	90	0.132274136	20	256	224	0.8984375\0.8984375	TCGA-19-0963/1.3.6.1.4.1.14519.5.2.1.5826.4001.182689849979752622358485655607/1.3.6.1.4.1.14519.5.2.1.5826.4001.524833529463103213283998865851
TCGA-GBM	TCGA-19	TCGA-19-0963	t2	M	61	77.18	MRHG_T2_AXIALS	2	1.06342E+15	10/12/2001	SIEMENS		Symphony	SE	2D	5	4000	100		63.65863	1.5	7.5	150	0.185955927	20	256	224	0.8984375\0.8984375	TCGA-19-0963/1.3.6.1.4.1.14519.5.2.1.5826.4001.182689849979752622358485655607/1.3.6.1.4.1.14519.5.2.1.5826.4001.122111680760213786740031783338
TCGA-GBM	TCGA-19	TCGA-19-1390	flair	F	63	113.5	MRHG_FLAIR_AX	3	2.89638E+15	3/20/2001	SIEMENS		Symphony	IR\SE	2D	5	9000	129	2500	63.50445	1.5	7	150	0.110135838	20	256	224	0.8984375\0.8984375	TCGA-19-1390/1.3.6.1.4.1.14519.5.2.1.5826.4001.231567380210698672838678790326/1.3.6.1.4.1.14519.5.2.1.5826.4001.249851327270284089776000264301
TCGA-GBM	TCGA-19	TCGA-19-1390	t1Gd	F	63	113.5	MP_RAGE_AXIAL	8	2.89638E+15	3/20/2001	SIEMENS		Symphony	IR\GR	3D	1	2160	3.45	1100	63.50445	1.5		15	0.051605709	192	256	256	1\1	TCGA-19-1390/1.3.6.1.4.1.14519.5.2.1.5826.4001.231567380210698672838678790326/1.3.6.1.4.1.14519.5.2.1.5826.4001.303700331855058891941526823748
TCGA-GBM	TCGA-19	TCGA-19-1390	t1	F	63	113.5	MRHG_T1_AX	4	2.89638E+15	3/20/2001	SIEMENS		Symphony	SE	2D	5	587	15		63.50445	1.5	7	90	0.234051749	20	256	224	0.8984375\0.8984375	TCGA-19-1390/1.3.6.1.4.1.14519.5.2.1.5826.4001.231567380210698672838678790326/1.3.6.1.4.1.14519.5.2.1.5826.4001.330679245867626368751388377114
TCGA-GBM	TCGA-19	TCGA-19-1390	t2	F	63	113.5	MRHG_T2_AXIALS	2	2.89638E+15	3/20/2001	SIEMENS		Symphony	SE	2D	5	4000	100		63.5044	1.5	7	150	0.32903868	20	256	224	0.8984375\0.8984375	TCGA-19-1390/1.3.6.1.4.1.14519.5.2.1.5826.4001.231567380210698672838678790326/1.3.6.1.4.1.14519.5.2.1.5826.4001.250425106424807032072862969016
TCGA-GBM	TCGA-19	TCGA-19-1789	flair	F	69	79.83227	FLAIR_AX	8	3.12367E+15	8/10/2002	SIEMENS		Avanto	SE\IR	2D	3	9430	109	2500	63.68429	1.5	3	150	0.239955982	60	256	232	1\1	TCGA-19-1789/1.3.6.1.4.1.14519.5.2.1.5826.4001.231928631261949063719336921407/1.3.6.1.4.1.14519.5.2.1.5826.4001.235249827946511179930616208703
TCGA-GBM	TCGA-19	TCGA-19-1789	t1Gd	F	69	79.83227	VOLUMAXGAD	12	3.12367E+15	8/10/2002	SIEMENS		Avanto	GR\IR	3D	1	2160	2.81	1100	63.68429	1.5		15	0.059041861	192	256	256	1\1	TCGA-19-1789/1.3.6.1.4.1.14519.5.2.1.5826.4001.231928631261949063719336921407/1.3.6.1.4.1.14519.5.2.1.5826.4001.110732246637966706387432991466
TCGA-GBM	TCGA-19	TCGA-19-1789	t1	F	69	79.83227	MP_RAGE_AXIAL_VOLUMETRIC	2	3.12367E+15	8/10/2002	SIEMENS		Avanto	GR\IR	3D	1	2160	2.81	1100	63.68429	1.5		15	0.059041861	192	256	256	1\1	TCGA-19-1789/1.3.6.1.4.1.14519.5.2.1.5826.4001.231928631261949063719336921407/1.3.6.1.4.1.14519.5.2.1.5826.4001.248150557952959587380829005187
TCGA-GBM	TCGA-19	TCGA-19-1789	t2	F	69	79.83227	TSE_T2_ax	9	3.12367E+15	8/10/2002	SIEMENS		Avanto	SE	2D	3	3580	91		63.68434	1.5	3	180	0.703742258	60	256	232	0.5\0.5	TCGA-19-1789/1.3.6.1.4.1.14519.5.2.1.5826.4001.231928631261949063719336921407/1.3.6.1.4.1.14519.5.2.1.5826.4001.296790073644899137258609675950
TCGA-GBM	TCGA-19	TCGA-19-2624	flair	M	51	90.71849	MRHR_FLAIR_AX	3	1.73254E+15	12/10/2002	SIEMENS		Avanto	SE\IR	2D	5	9000	109	2500	63.68411	1.5	7	150	0.13693494	25	256	192	0.95703125\0.95703125	TCGA-19-2624/1.3.6.1.4.1.14519.5.2.1.5826.4001.209294630890894137037336921815/1.3.6.1.4.1.14519.5.2.1.5826.4001.237957332039616981825055417941
TCGA-GBM	TCGA-19	TCGA-19-2624	t1Gd	M	51	90.71849	MP_RAGE_AXIAL_VOLUMETRIC	12	1.73254E+15	12/10/2002	SIEMENS		Avanto	GR\IR	3D	1	2160	2.81	1100	63.68411	1.5		15	0.05179356	192	256	256	1\1	TCGA-19-2624/1.3.6.1.4.1.14519.5.2.1.5826.4001.209294630890894137037336921815/1.3.6.1.4.1.14519.5.2.1.5826.4001.315244591826595784983776021079
TCGA-GBM	TCGA-19	TCGA-19-2624	t1	M	51	90.71849	MRHR_T1_AX	11	1.73254E+15	12/10/2002	SIEMENS		Avanto	SE	2D	5	586	14		63.68411	1.5	7	70	0.419917791	25	256	192	0.95703125\0.95703125	TCGA-19-2624/1.3.6.1.4.1.14519.5.2.1.5826.4001.209294630890894137037336921815/1.3.6.1.4.1.14519.5.2.1.5826.4001.288482729261153427645680784384
TCGA-GBM	TCGA-19	TCGA-19-2624	t2	M	51	90.71849	MRHR_T2_AX	2	1.73254E+15	12/10/2002	SIEMENS		Avanto	SE	2D	5	4650	96		63.68408	1.5	7	150	0.236890556	25	256	192	0.95703125\0.95703125	TCGA-19-2624/1.3.6.1.4.1.14519.5.2.1.5826.4001.209294630890894137037336921815/1.3.6.1.4.1.14519.5.2.1.5826.4001.648135048726787046712784361279
TCGA-GBM	TCGA-19	TCGA-19-2631	flair	F	74	102.0583	MRHR_FLAIR_AX	4	2.58878E+15	3/2/2003	SIEMENS		Avanto	SE\IR	2D	5	9000	109	2500	63.68498	1.5	5.5	150	0.125045094	28	256	192	0.8984375\0.8984375	TCGA-19-2631/1.3.6.1.4.1.14519.5.2.1.5826.4001.113291808789528092767840707216/1.3.6.1.4.1.14519.5.2.1.5826.4001.152727517643491829458173260916
TCGA-GBM	TCGA-19	TCGA-19-2631	t1Gd	F	74	102.0583	MRHR_T1_AX_POST_GAD	8	2.58878E+15	3/2/2003	SIEMENS		Avanto	SE	2D	5	718	17		63.68498	1.5	5.5	90	0.170915377	28	256	192	0.8984375\0.8984375	TCGA-19-2631/1.3.6.1.4.1.14519.5.2.1.5826.4001.113291808789528092767840707216/1.3.6.1.4.1.14519.5.2.1.5826.4001.328461846490310318014149271099
TCGA-GBM	TCGA-19	TCGA-19-2631	t1	F	74	102.0583	MRHR_T1_AX	7	2.58878E+15	3/2/2003	SIEMENS		Avanto	SE	2D	5	532	10		63.68498	1.5	5.5	90	0.230387199	28	256	192	0.8984375\0.8984375	TCGA-19-2631/1.3.6.1.4.1.14519.5.2.1.5826.4001.113291808789528092767840707216/1.3.6.1.4.1.14519.5.2.1.5826.4001.171072817207494283871306430547
TCGA-GBM	TCGA-19	TCGA-19-2631	t2	F	74	102.0583	MRHR_T2_AX	3	2.58878E+15	3/2/2003	SIEMENS		Avanto	SE	2D	5	3800	95		63.68497	1.5	5.5	150	0.327169674	28	256	192	0.8984375\0.8984375	TCGA-19-2631/1.3.6.1.4.1.14519.5.2.1.5826.4001.113291808789528092767840707216/1.3.6.1.4.1.14519.5.2.1.5826.4001.114530788702118112150801713711
TCGA-GBM	TCGA-19	TCGA-19-5951	flair	M	76	93.89363	FLAIR_AX	3	3.39069E+15	9/14/2003	SIEMENS		Verio	SE\IR	2D	4	9000	94	2500	123.2487	3	4.52	150	0.132235472	31	256	169	0.8984375\0.8984375	TCGA-19-5951/1.3.6.1.4.1.14519.5.2.1.5826.4001.194554107490457210178645733278/1.3.6.1.4.1.14519.5.2.1.5826.4001.321497813789636909159148294851
TCGA-GBM	TCGA-19	TCGA-19-5951	t1Gd	M	76	93.89363	T1_AXIAL_Gd	7	3.39069E+15	9/14/2003	SIEMENS		Verio	GR	2D	4	250	2.48		123.2487	3	4.52	70	0.258163656	31	320	211	0.71875\0.71875	TCGA-19-5951/1.3.6.1.4.1.14519.5.2.1.5826.4001.194554107490457210178645733278/1.3.6.1.4.1.14519.5.2.1.5826.4001.330225254008219946706138731112
TCGA-GBM	TCGA-19	TCGA-19-5951	t1	M	76	93.89363	TIR_T1_AX	6	3.39069E+15	9/14/2003	SIEMENS		Verio	SE\IR	2D	4	2800	19	701.1	123.2487	3	4.52	150	0.309723401	31	256	169	0.8984375\0.8984375	TCGA-19-5951/1.3.6.1.4.1.14519.5.2.1.5826.4001.194554107490457210178645733278/1.3.6.1.4.1.14519.5.2.1.5826.4001.555701715534477096875939011679
TCGA-GBM	TCGA-19	TCGA-19-5951	t2	M	76	93.89363	T2_AX	2	3.39069E+15	9/14/2003	SIEMENS		Verio	SE	2D	4	5500	96		123.2487	3	4.52	150	0.265902937	31	320	211	0.71875\0.71875	TCGA-19-5951/1.3.6.1.4.1.14519.5.2.1.5826.4001.194554107490457210178645733278/1.3.6.1.4.1.14519.5.2.1.5826.4001.932737691207898989363089745632
TCGA-GBM	TCGA-19	TCGA-19-5954	flair	F	72	77.11071	FLAIR_AX	3	2.97506E+15	12/30/2003	SIEMENS		Verio	SE\IR	2D	3	9000	94	2500	123.2487	3	3	150	0.330161553	54	256	256	1\1	TCGA-19-5954/1.3.6.1.4.1.14519.5.2.1.5826.4001.321057407286032186200454220971/1.3.6.1.4.1.14519.5.2.1.5826.4001.253159030516730311576836577490
TCGA-GBM	TCGA-19	TCGA-19-5954	t1Gd	F	72	77.11071	VOLUMETRIC_AXIAL_Gd	11	2.97506E+15	12/30/2003	SIEMENS		Verio	GR\IR	3D	1	1900	2.75	900	123.2487	3		9	0.054662186	192	256	251	1\1	TCGA-19-5954/1.3.6.1.4.1.14519.5.2.1.5826.4001.321057407286032186200454220971/1.3.6.1.4.1.14519.5.2.1.5826.4001.560018405631653221233558769839
TCGA-GBM	TCGA-19	TCGA-19-5954	t1	F	72	77.11071	VOLUMETRIC_AXIAL	2	2.97506E+15	12/30/2003	SIEMENS		Verio	GR\IR	3D	1	1900	2.75	900	123.2487	3		9	0.054662186	192	256	251	1\1	TCGA-19-5954/1.3.6.1.4.1.14519.5.2.1.5826.4001.321057407286032186200454220971/1.3.6.1.4.1.14519.5.2.1.5826.4001.295910608118826040218967173851
TCGA-GBM	TCGA-19	TCGA-19-5954	t2	F	72	77.11071	T2_AX	4	2.97506E+15	12/30/2003	SIEMENS		Verio	SE	2D	3	5500	93		123.2487	3	3	150	0.334439301	54	256	256	1\1	TCGA-19-5954/1.3.6.1.4.1.14519.5.2.1.5826.4001.321057407286032186200454220971/1.3.6.1.4.1.14519.5.2.1.5826.4001.301348699602097269733909521821
TCGA-GBM	TCGA-19	TCGA-19-5958	flair	M	56	77.11071	FLAIR_AX	3	2.64703E+15	3/9/2004	SIEMENS		Verio	SE\IR	2D	3	9000	94	2500	123.2486	3	3	150	0.253902656	54	256	256	1\1	TCGA-19-5958/1.3.6.1.4.1.14519.5.2.1.5826.4001.192041630414624709736670800727/1.3.6.1.4.1.14519.5.2.1.5826.4001.151132655513156536888042112741
TCGA-GBM	TCGA-19	TCGA-19-5958	t1Gd	M	56	77.11071	VOLUMETRIC_AXIAL_Gd	11	2.64703E+15	3/9/2004	SIEMENS		Verio	GR\IR	3D	1	1900	2.75	900	123.2487	3		9	0.042036613	192	256	251	1\1	TCGA-19-5958/1.3.6.1.4.1.14519.5.2.1.5826.4001.192041630414624709736670800727/1.3.6.1.4.1.14519.5.2.1.5826.4001.157222381431362159457522834384
TCGA-GBM	TCGA-19	TCGA-19-5958	t1	M	56	77.11071	VOLUMETRIC_AXIAL	2	2.64703E+15	3/9/2004	SIEMENS		Verio	GR\IR	3D	1	1900	2.75	900	123.2487	3		9	0.042036613	192	256	251	1\1	TCGA-19-5958/1.3.6.1.4.1.14519.5.2.1.5826.4001.192041630414624709736670800727/1.3.6.1.4.1.14519.5.2.1.5826.4001.179990125364012900028568210197
TCGA-GBM	TCGA-19	TCGA-19-5958	t2	M	56	77.11071	T2_AX	4	2.64703E+15	3/9/2004	SIEMENS		Verio	SE	2D	3	5500	93		123.2487	3	3	150	0.257192359	54	256	256	1\1	TCGA-19-5958/1.3.6.1.4.1.14519.5.2.1.5826.4001.192041630414624709736670800727/1.3.6.1.4.1.14519.5.2.1.5826.4001.307938529330684497954612875613
TCGA-GBM	TCGA-19	TCGA-19-5960	flair	M	56	72.57479	FLAIR_AX	7	3.03248E+15	3/15/2004	SIEMENS		Verio	SE\IR	2D	4	9000	94	2500	123.2486	3	4.52	150	0.12577795	34	256	183	0.8984375\0.8984375	TCGA-19-5960/1.3.6.1.4.1.14519.5.2.1.5826.4001.135874542095979859687918030388/1.3.6.1.4.1.14519.5.2.1.5826.4001.186796014901942467105889390386
TCGA-GBM	TCGA-19	TCGA-19-5960	t1Gd	M	56	72.57479	T1_AXIAL_Gd	9	3.03248E+15	3/15/2004	SIEMENS		Verio	GR	2D	4	250	2.48		123.2487	3	4.52	70	0.246707612	34	256	183	0.8984375\0.8984375	TCGA-19-5960/1.3.6.1.4.1.14519.5.2.1.5826.4001.135874542095979859687918030388/1.3.6.1.4.1.14519.5.2.1.5826.4001.330951756415447864524009831768
TCGA-GBM	TCGA-19	TCGA-19-5960	t1	M	56	72.57479	TIR_T1_AX	8	3.03248E+15	3/15/2004	SIEMENS		Verio	SE\IR	2D	4	2990	19	715.6	123.2487	3	4.52	150	0.24781075	34	256	183	0.8984375\0.8984375	TCGA-19-5960/1.3.6.1.4.1.14519.5.2.1.5826.4001.135874542095979859687918030388/1.3.6.1.4.1.14519.5.2.1.5826.4001.566265127405363595842987702076
TCGA-GBM	TCGA-19	TCGA-19-5960	t2	M	56	72.57479	T2_AX	3	3.03248E+15	3/15/2004	SIEMENS		Verio	SE	2D	4	5500	93		123.2487	3	4.52	150	0.243332034	34	256	183	0.8984375\0.8984375	TCGA-19-5960/1.3.6.1.4.1.14519.5.2.1.5826.4001.135874542095979859687918030388/1.3.6.1.4.1.14519.5.2.1.5826.4001.139985413894688673535204262164
TCGA-GBM	TCGA-27	TCGA-27-1834	flair	M	57	65	T2_Flair_cor_5mm_5_9_07	4	3.05234E+15	11/3/1987	SIEMENS		Avanto	SE\IR	2D	5	8200	91	2500	63.63845	1.5	6.5	150	0.077359584	25	320	230	0.78125\0.78125	TCGA-27-1834/1.3.6.1.4.1.14519.5.2.1.3775.4001.257288270999874248292312942337/1.3.6.1.4.1.14519.5.2.1.3775.4001.220362068065489352287173639632
TCGA-GBM	TCGA-27	TCGA-27-1834	t1Gd	M	57	65	t1_se_tra_5_9_07	7	3.05234E+15	11/3/1987	SIEMENS		Avanto	SE	2D	6	477	10		63.63845	1.5	7.8	70	0.270370927	20	256	224	0.44921875\0.44921875	TCGA-27-1834/1.3.6.1.4.1.14519.5.2.1.3775.4001.257288270999874248292312942337/1.3.6.1.4.1.14519.5.2.1.3775.4001.578556393784694438698235299037
TCGA-GBM	TCGA-27	TCGA-27-1834	t1	M	57	65	t1_se_tra_5_9_07	5	3.05234E+15	11/3/1987	SIEMENS		Avanto	SE	2D	6	477	10		63.63845	1.5	7.8	70	0.270370927	20	256	224	0.44921875\0.44921875	TCGA-27-1834/1.3.6.1.4.1.14519.5.2.1.3775.4001.257288270999874248292312942337/1.3.6.1.4.1.14519.5.2.1.3775.4001.187906847315134178012322222812
TCGA-GBM	TCGA-27	TCGA-27-1834	t2	M	57	65	pd+t2_tse_tra_5_9_07	3	3.05234E+15	11/3/1987	SIEMENS		Avanto	SE	2D	5	3621.9	13		63.63845	1.5	6.5	150	0.30795125	50	320	198	0.71875\0.71875	TCGA-27-1834/1.3.6.1.4.1.14519.5.2.1.3775.4001.257288270999874248292312942337/1.3.6.1.4.1.14519.5.2.1.3775.4001.364247940891429788631023542309
TCGA-GBM	TCGA-27	TCGA-27-1838	flair	M		75	ET2W/FLAIR_	302	2.86841E+15	8/29/1988	Philips Medical Systems		Intera	IR		6	6000	120	1900	21.29607	0.5	7.2	90		20	256	176	0.8984375\0.8984375	TCGA-27-1838/1.3.6.1.4.1.14519.5.2.1.3775.4001.111712322912660120243025601400/1.3.6.1.4.1.14519.5.2.1.3775.4001.119287179819363124512383566810
TCGA-GBM	TCGA-27	TCGA-27-1838	t1Gd	M		75	Egad	502	2.86841E+15	8/29/1988	Philips Medical Systems		Intera	SE		6	542.022	15		21.29607	0.5	6.6	60		20	224	179	0.9375\0.9375	TCGA-27-1838/1.3.6.1.4.1.14519.5.2.1.3775.4001.111712322912660120243025601400/1.3.6.1.4.1.14519.5.2.1.3775.4001.994851682380835865056380711802
TCGA-GBM	TCGA-27	TCGA-27-1838	t1	M		75	Et1_se_tra	402	2.86841E+15	8/29/1988	Philips Medical Systems		Intera	SE		6	542.022	15		21.29607	0.5	6.6	60		20	224	179	0.9375\0.9375	TCGA-27-1838/1.3.6.1.4.1.14519.5.2.1.3775.4001.111712322912660120243025601400/1.3.6.1.4.1.14519.5.2.1.3775.4001.788024501395553679565117126823
TCGA-GBM	TCGA-27	TCGA-27-1838	t2	M		75	Edp+t2_se_t	202	2.86841E+15	8/29/1988	Philips Medical Systems		Intera	SE		6	2219.564	20		21.29607	0.5	6.6	90		40	256	192	0.9375\0.9375	TCGA-27-1838/1.3.6.1.4.1.14519.5.2.1.3775.4001.111712322912660120243025601400/1.3.6.1.4.1.14519.5.2.1.3775.4001.957862673647515802653015684121
TCGA-GBM	TCGA-27	TCGA-27-2526	flair	M		77	EFLAIR/long	402	2.29145E+15	3/7/1988	Philips Medical Systems		Intera	IR		6	11000	140	2800	21.29607	0.5	7	90		20	256	161	0.9375\0.9375	TCGA-27-2526/1.3.6.1.4.1.14519.5.2.1.3775.4001.338505699209290103023897865510/1.3.6.1.4.1.14519.5.2.1.3775.4001.242527138671194700564129592420
TCGA-GBM	TCGA-27	TCGA-27-2526	t1Gd	M		77	neuronavigaT1W/3D/GAD	501	2.29145E+15	3/7/1988	Philips Medical Systems		Intera	GR		2	30.3	13.81153		21.29607	0.5	2	30		80	256	256	1.0\1.0	TCGA-27-2526/1.3.6.1.4.1.14519.5.2.1.3775.4001.338505699209290103023897865510/1.3.6.1.4.1.14519.5.2.1.3775.4001.199545238646153862339050303771
TCGA-GBM	TCGA-27	TCGA-27-2526	t1	M		77	Et1_se_tra	302	2.29145E+15	3/7/1988	Philips Medical Systems		Intera	SE		6	541.926	15		21.29607	0.5	6.6	60		20	256	179	0.9375\0.9375	TCGA-27-2526/1.3.6.1.4.1.14519.5.2.1.3775.4001.338505699209290103023897865510/1.3.6.1.4.1.14519.5.2.1.3775.4001.290126392868809844278609708560
TCGA-GBM	TCGA-27	TCGA-27-2526	t2	M		77	Edp+t2_se_t	202	2.29145E+15	3/7/1988	Philips Medical Systems		Intera	SE		6	2219.564	20		21.29607	0.5	6.6	90		40	256	179	0.9375\0.9375	TCGA-27-2526/1.3.6.1.4.1.14519.5.2.1.3775.4001.338505699209290103023897865510/1.3.6.1.4.1.14519.5.2.1.3775.4001.337166470300809460748886608281
TCGA-GBM	TCGA-76	TCGA-76-4932	flair	F	50	77	FAST_BRAIN/FLAIR_AXIAL	6	2.08702E+15	3/16/1997	SIEMENS	mrscan	MAGNETOM VISION	IR	2D	5	7000	105	2250	63.61525	1.493806	2.5	180	0	178	512	140	008.984375E-01\08.984375E-01	TCGA-76-4932/1.3.6.1.4.1.14519.5.2.1.1188.4001.696081929184000436121119244803/1.3.6.1.4.1.14519.5.2.1.1188.4001.325184889034836529092840230248
TCGA-GBM	TCGA-76	TCGA-76-4932	t1Gd	F	50	77	BRAIN_STRYKER/t1_AX	8	2.08702E+15	3/16/1997	SIEMENS	mrscan	MAGNETOM VISION	RM	2D	5	880	12		63.61548	1.493806	2.5	70	0	258	512	144	008.593750E-01\08.593750E-01	TCGA-76-4932/1.3.6.1.4.1.14519.5.2.1.1188.4001.696081929184000436121119244803/1.3.6.1.4.1.14519.5.2.1.1188.4001.127105277778356529905172144511
TCGA-GBM	TCGA-76	TCGA-76-4932	t1	F	50	77	BRAIN_STRYKER/T1_AXIAL	3	2.08702E+15	3/16/1997	SIEMENS	mrscan	MAGNETOM VISION	RM	2D	5	540	12		63.61548	1.493806	2.5	70	0	39	512	144	008.593750E-01\08.593750E-01	TCGA-76-4932/1.3.6.1.4.1.14519.5.2.1.1188.4001.696081929184000436121119244803/1.3.6.1.4.1.14519.5.2.1.1188.4001.336336678889681314725089183401
TCGA-GBM	TCGA-76	TCGA-76-4932	t2	F	50	77	BRAIN_STRYKER/T2_AX_DOUBLE	4	2.08702E+15	3/16/1997	SIEMENS	mrscan	MAGNETOM VISION	RM	2D	5	2020	20		63.61548	1.493806	2.5	90	0	79	512	221	004.296875E-01\04.296875E-01	TCGA-76-4932/1.3.6.1.4.1.14519.5.2.1.1188.4001.696081929184000436121119244803/1.3.6.1.4.1.14519.5.2.1.1188.4001.210657790793239037253399861860
TCGA-GBM	TCGA-76	TCGA-76-4934	flair	F		100	T2_AX_FLAIR	601	7.26288E+15	10/8/2000	Philips Medical Systems	PHILIPS-13EEFF6	Achieva	IR	2D	5	11000	125	2850	127.796	3	6	90	0.90577203	25	336	255	0.68452382087707\0.68452382087707	TCGA-76-4934/1.3.6.1.4.1.14519.5.2.1.1188.4001.280345518346345066284488429203/1.3.6.1.4.1.14519.5.2.1.1188.4001.286635506916337591478062137823
TCGA-GBM	TCGA-76	TCGA-76-4934	t1Gd	F		100	T1_AX_SE_FS_POST	1401	7.26288E+15	10/8/2000	Philips Medical Systems	PHILIPS-13EEFF6	Achieva	SE	2D	4	699.84	10		127.7961	3	5	90	3.200000048	32	284	228	0.71875\0.71875	TCGA-76-4934/1.3.6.1.4.1.14519.5.2.1.1188.4001.280345518346345066284488429203/1.3.6.1.4.1.14519.5.2.1.1188.4001.301507599146459685277894885768
TCGA-GBM	TCGA-76	TCGA-76-4934	t1	F		100	T1_AX__SE	901	7.26288E+15	10/8/2000	Philips Medical Systems	PHILIPS-13EEFF6	Achieva	SE	2D	4	565.0768	10.3		127.7961	3	5	90	3.199997187	32	316	255	0.68452382087707\0.68452382087707	TCGA-76-4934/1.3.6.1.4.1.14519.5.2.1.1188.4001.280345518346345066284488429203/1.3.6.1.4.1.14519.5.2.1.1188.4001.338412102935503631330631798910
TCGA-GBM	TCGA-76	TCGA-76-4934	t2	F		100	AXIAL__T2	701	7.26288E+15	10/8/2000	Philips Medical Systems	PHILIPS-13EEFF6	Achieva	SE	2D	4	3361.032	100		127.796	3	5	90	3.160874844	32	404	340	0.44921875\0.44921875	TCGA-76-4934/1.3.6.1.4.1.14519.5.2.1.1188.4001.280345518346345066284488429203/1.3.6.1.4.1.14519.5.2.1.1188.4001.134660201518103802336514714033
TCGA-GBM	TCGA-76	TCGA-76-4935	flair	F		65	T2_AX_FLAIR	801	2.56097E+15	1/22/2001	Philips Medical Systems	PHILIPS-13EEFF6	Achieva	IR	2D	5	11000	125	2850	127.7953	3	6	90	1.033632755	28	348	272	0.68181818723678\0.68181818723678	TCGA-76-4935/1.3.6.1.4.1.14519.5.2.1.1188.4001.279290817710613414079827510861/1.3.6.1.4.1.14519.5.2.1.1188.4001.198703612394476952929652973732
TCGA-GBM	TCGA-76	TCGA-76-4935	t1Gd	F		65	T1_AX_SE_FS_POST	1301	2.56097E+15	1/22/2001	Philips Medical Systems	PHILIPS-13EEFF6	Achieva	SE	2D	4	533.63	10		127.7954	3	5	90	3.199999571	30	296	266	0.75\0.75	TCGA-76-4935/1.3.6.1.4.1.14519.5.2.1.1188.4001.279290817710613414079827510861/1.3.6.1.4.1.14519.5.2.1.1188.4001.120015554026401136166447740917
TCGA-GBM	TCGA-76	TCGA-76-4935	t1	F		65	T1_AX__SE	1101	2.56097E+15	1/22/2001	Philips Medical Systems	PHILIPS-13EEFF6	Achieva	SE	2D	4	719.0448	10.3		127.7954	3	5	90	3.199997187	32	332	266	0.68181818723678\0.68181818723678	TCGA-76-4935/1.3.6.1.4.1.14519.5.2.1.1188.4001.279290817710613414079827510861/1.3.6.1.4.1.14519.5.2.1.1188.4001.210457495932820162711563788216
TCGA-GBM	TCGA-76	TCGA-76-4935	t2	F		65	AXIAL__T2	901	2.56097E+15	1/22/2001	Philips Medical Systems	PHILIPS-13EEFF6	Achieva	SE	2D	4	3367.982	100		127.7953	3	5	90	3.15435195	32	420	337	0.42857143282890\0.42857143282890	TCGA-76-4935/1.3.6.1.4.1.14519.5.2.1.1188.4001.279290817710613414079827510861/1.3.6.1.4.1.14519.5.2.1.1188.4001.165274213290821040312747820547
TCGA-GBM	TCGA-76	TCGA-76-6191	flair	M	57	136	BRAIN_CONTRAST/FLAIR	3	9.71383E+15	10/20/2000	SIEMENS	mrscan	MAGNETOM VISION	IR	2D	5	9000	105	2340	63.62077	1.493806	3.25	180	0	43	512	112	009.765625E-01\09.765625E-01	TCGA-76-6191/1.3.6.1.4.1.14519.5.2.1.1188.4001.237134987835312583841357983114/1.3.6.1.4.1.14519.5.2.1.1188.4001.269462493891859236294268767034
TCGA-GBM	TCGA-76	TCGA-76-6191	t1Gd	M	57	136	BRAIN_CONTRAST/T1_AXIAL	4	9.71383E+15	10/20/2000	SIEMENS	mrscan	MAGNETOM VISION	RM	2D	5	600	14		63.62077	1.493806	3.25	70	0	63	512	115	009.765625E-01\09.765625E-01	TCGA-76-6191/1.3.6.1.4.1.14519.5.2.1.1188.4001.237134987835312583841357983114/1.3.6.1.4.1.14519.5.2.1.1188.4001.193088623684162190916524071714
TCGA-GBM	TCGA-76	TCGA-76-6191	t1	M	57	136	BRAIN_STRYKER/t1_AX	9	9.71383E+15	10/20/2000	SIEMENS	mrscan	MAGNETOM VISION	RM	2D	5	440	12		63.62068	1.493806	3.25	70	0	343	512	134	009.765625E-01\09.765625E-01	TCGA-76-6191/1.3.6.1.4.1.14519.5.2.1.1188.4001.237134987835312583841357983114/1.3.6.1.4.1.14519.5.2.1.1188.4001.139468407540359589585598023466
TCGA-GBM	TCGA-76	TCGA-76-6191	t2	M	57	136	BRAIN_CONTRAST/DOUBLE_ECHO	5	9.71383E+15	10/20/2000	SIEMENS	mrscan	MAGNETOM VISION	RM	2D	5	2020	20		63.62077	1.493806	3.25	90	0	103	512	115	004.882813E-01\04.882813E-01	TCGA-76-6191/1.3.6.1.4.1.14519.5.2.1.1188.4001.237134987835312583841357983114/1.3.6.1.4.1.14519.5.2.1.1188.4001.714380615105580420167556298073
TCGA-GBM	TCGA-76	TCGA-76-6193	flair	M	78	86	BRAIN_NON_CONTRAST/FLAIR_A	4	2.29039E+15	6/2/2001	SIEMENS	mrscan	MAGNETOM VISION	IR	2D	5	9000	105	2340	63.62074	1.493806	2.5	180	0	59	512	126	008.593750E-01\08.593750E-01	TCGA-76-6193/1.3.6.1.4.1.14519.5.2.1.1188.4001.532365074075118365845331160175/1.3.6.1.4.1.14519.5.2.1.1188.4001.331171186011270999664762183981
TCGA-GBM	TCGA-76	TCGA-76-6193	t1Gd	M	78	86	BRAIN_STRYKER/AX-RAGE-STRY	9	2.29039E+15	6/2/2001	SIEMENS	mrscan	MAGNETOM VISION	IR	3D	1.5	15	7	300	63.62069	1.493806		15	0	339	512	256	005.078125E-01\05.078125E-01	TCGA-76-6193/1.3.6.1.4.1.14519.5.2.1.1188.4001.532365074075118365845331160175/1.3.6.1.4.1.14519.5.2.1.1188.4001.140712239861920670139072389080
TCGA-GBM	TCGA-76	TCGA-76-6193	t1	M	78	86	BRAIN_STRYKER/t1_AX	10	2.29039E+15	6/2/2001	SIEMENS	mrscan	MAGNETOM VISION	RM	2D	5	540	12		63.62069	1.493806	2.500004	70	0	359	512	134	008.593750E-01\08.593750E-01	TCGA-76-6193/1.3.6.1.4.1.14519.5.2.1.1188.4001.532365074075118365845331160175/1.3.6.1.4.1.14519.5.2.1.1188.4001.164007274133639277695926891010
TCGA-GBM	TCGA-76	TCGA-76-6193	t2	M	78	86	BRAIN_CONTRAST/DOUBLE_ECHO	7	2.29039E+15	6/2/2001	SIEMENS	mrscan	MAGNETOM VISION	RM	2D	5	2020	20		63.62074	1.493806	2.499951	90	0	139	512	115	004.296875E-01\04.296875E-01	TCGA-76-6193/1.3.6.1.4.1.14519.5.2.1.1188.4001.532365074075118365845331160175/1.3.6.1.4.1.14519.5.2.1.1188.4001.520134226758299999468011999899
TCGA-GBM	TCGA-76	TCGA-76-6280	flair	M		79	T2_AX_FLAIR	401	1.39014E+15	7/21/1998	Philips Medical Systems	PMSN-1DL0VL9SN4	Achieva	IR		5	6000	120	2000	63.90027	1.5	6	90		28	256	161	0.859375\0.859375	TCGA-76-6280/1.3.6.1.4.1.14519.5.2.1.1188.4001.259303451742499057480927151400/1.3.6.1.4.1.14519.5.2.1.1188.4001.274502557202127285948259115378
TCGA-GBM	TCGA-76	TCGA-76-6280	t1Gd	M		79	T1_SE_POST	701	1.39014E+15	7/21/1998	Philips Medical Systems	PMSN-1DL0VL9SN4	Achieva	SE		5	589.224	12		63.90028	1.5	7	69		25	256	204	0.859375\0.859375	TCGA-76-6280/1.3.6.1.4.1.14519.5.2.1.1188.4001.259303451742499057480927151400/1.3.6.1.4.1.14519.5.2.1.1188.4001.259307244299991614762344622590
TCGA-GBM	TCGA-76	TCGA-76-6280	t1	M		79	T1_SE_PRE	601	1.39014E+15	7/21/1998	Philips Medical Systems	PMSN-1DL0VL9SN4	Achieva	SE		5	413.72	12		63.90027	1.5	7	69		25	256	204	0.859375\0.859375	TCGA-76-6280/1.3.6.1.4.1.14519.5.2.1.1188.4001.259303451742499057480927151400/1.3.6.1.4.1.14519.5.2.1.1188.4001.261189460146477809142077908700
TCGA-GBM	TCGA-76	TCGA-76-6280	t2	M		79	DUAL_AX_T2	501	1.39014E+15	7/21/1998	Philips Medical Systems	PMSN-1DL0VL9SN4	Achieva	SE		5	2991.893	30		63.90027	1.5	7.5	90		48	256	153	0.859375\0.859375	TCGA-76-6280/1.3.6.1.4.1.14519.5.2.1.1188.4001.259303451742499057480927151400/1.3.6.1.4.1.14519.5.2.1.1188.4001.113253988688109445728345358337
TCGA-GBM	TCGA-76	TCGA-76-6282	flair	M	63	91	BRAIN_STRYKER/FLAIR	6	5.47453E+15	8/24/1998	SIEMENS	mrscan	MAGNETOM VISION	IR	2D	5	9000	105	2340	63.61581	1.493806	2.5	180	0	104	512	140	008.593750E-01\08.593750E-01	TCGA-76-6282/1.3.6.1.4.1.14519.5.2.1.1188.4001.623989292006918600441736922866/1.3.6.1.4.1.14519.5.2.1.1188.4001.337742353862340790005797114133
TCGA-GBM	TCGA-76	TCGA-76-6282	t1Gd	M	63	91	BRAIN_STRYKER/AX-RAGE-STRY	8	5.47453E+15	8/24/1998	SIEMENS	mrscan	MAGNETOM VISION	IR	3D	1.5	15	7	300	63.61575	1.493806		15	0	304	512	179	004.687500E-01\04.687500E-01	TCGA-76-6282/1.3.6.1.4.1.14519.5.2.1.1188.4001.623989292006918600441736922866/1.3.6.1.4.1.14519.5.2.1.1188.4001.120631863329002959798073074121
TCGA-GBM	TCGA-76	TCGA-76-6282	t1	M	63	91	BRAIN_STRYKER/T1_AXIAL	4	5.47453E+15	8/24/1998	SIEMENS	mrscan	MAGNETOM VISION	RM	2D	5	540	12		63.61574	1.493806	2.5	70	0	44	512	134	008.593750E-01\08.593750E-01	TCGA-76-6282/1.3.6.1.4.1.14519.5.2.1.1188.4001.623989292006918600441736922866/1.3.6.1.4.1.14519.5.2.1.1188.4001.288195363293492828132191428304
TCGA-GBM	TCGA-76	TCGA-76-6282	t2	M	63	91	BRAIN_STRYKER/T2_AX_DOUBLE	5	5.47453E+15	8/24/1998	SIEMENS	mrscan	MAGNETOM VISION	RM	2D	5	2020	20		63.61574	1.493806	2.5	90	0	84	512	134	004.296875E-01\04.296875E-01	TCGA-76-6282/1.3.6.1.4.1.14519.5.2.1.1188.4001.623989292006918600441736922866/1.3.6.1.4.1.14519.5.2.1.1188.4001.404945593685724778366507370745
TCGA-GBM	TCGA-76	TCGA-76-6285	flair	F	64	136	BRAIN_STRYKER/FLAIR	4	8.29785E+15	12/19/1998	SIEMENS	mrscan	MAGNETOM VISION	IR	2D	5	9000	105	2340	63.6157	1.493806	2	180	0	61	512	98	009.375000E-01\09.375000E-01	TCGA-76-6285/1.3.6.1.4.1.14519.5.2.1.1188.4001.440169796153998480809455332999/1.3.6.1.4.1.14519.5.2.1.1188.4001.284225597658034426109919290461
TCGA-GBM	TCGA-76	TCGA-76-6285	t1Gd	F	64	136	BRAIN_STRYKER/t1_AX	7	8.29785E+15	12/19/1998	SIEMENS	mrscan	MAGNETOM VISION	RM	2D	5	440	12		63.61561	1.493806	2	70	0	181	512	134	009.375000E-01\09.375000E-01	TCGA-76-6285/1.3.6.1.4.1.14519.5.2.1.1188.4001.440169796153998480809455332999/1.3.6.1.4.1.14519.5.2.1.1188.4001.239825616284727719466680894711
TCGA-GBM	TCGA-76	TCGA-76-6285	t1	F	64	136	BRAIN_STRYKER/T1_AXIAL	3	8.29785E+15	12/19/1998	SIEMENS	mrscan	MAGNETOM VISION	RM	2D	5	440	12		63.61568	1.493806	2	70	0	41	512	134	009.375000E-01\09.375000E-01	TCGA-76-6285/1.3.6.1.4.1.14519.5.2.1.1188.4001.440169796153998480809455332999/1.3.6.1.4.1.14519.5.2.1.1188.4001.175007799198843363270738924370
TCGA-GBM	TCGA-76	TCGA-76-6285	t2	F	64	136	RETRO_ORBIT/T2_LATE_ECHO_B	5	8.29785E+15	12/19/1998	SIEMENS	mrscan	MAGNETOM VISION	RM	2D	5	4700	120		63.6157	1.493806	2	180	0	81	512	150	009.375000E-01\09.375000E-01	TCGA-76-6285/1.3.6.1.4.1.14519.5.2.1.1188.4001.440169796153998480809455332999/1.3.6.1.4.1.14519.5.2.1.1188.4001.140445044250167916734736842790
TCGA-GBM	TCGA-76	TCGA-76-6656	flair	M		68	T2_AX_FLAIR	601	2.06192E+15	6/2/2001	Philips Medical Systems	PHILIPS-13EEFF6	Achieva	IR	2D	5	11000	125	2850	127.7946	3	6	90	0.984625876	28	336	255	0.68452382087707\0.68452382087707	TCGA-76-6656/1.3.6.1.4.1.14519.5.2.1.1188.4001.313245752188924211777085602901/1.3.6.1.4.1.14519.5.2.1.1188.4001.313956114391186647662214330900
TCGA-GBM	TCGA-76	TCGA-76-6656	t1Gd	M		68	T1_AX_SE_FS_POST	1101	2.06192E+15	6/2/2001	Philips Medical Systems	PHILIPS-13EEFF6	Achieva	SE	2D	4	656.1	10		127.7947	3	5	90	3.200000048	30	284	228	0.71875\0.71875	TCGA-76-6656/1.3.6.1.4.1.14519.5.2.1.1188.4001.313245752188924211777085602901/1.3.6.1.4.1.14519.5.2.1.1188.4001.307641110251975013714713829108
TCGA-GBM	TCGA-76	TCGA-76-6656	t1	M		68	T1_AX__SE	901	2.06192E+15	6/2/2001	Philips Medical Systems	PHILIPS-13EEFF6	Achieva	SE	2D	4	529.7595	10.3		127.7946	3	5	90	3.199996948	30	316	255	0.68452382087707\0.68452382087707	TCGA-76-6656/1.3.6.1.4.1.14519.5.2.1.1188.4001.313245752188924211777085602901/1.3.6.1.4.1.14519.5.2.1.1188.4001.234057418002688075397382125444
TCGA-GBM	TCGA-76	TCGA-76-6656	t2	M		68	AXIAL__T2	701	2.06192E+15	6/2/2001	Philips Medical Systems	PHILIPS-13EEFF6	Achieva	SE	2D	4	3143.824	100		127.7946	3	5	90	3.168056727	30	404	340	0.44921875\0.44921875	TCGA-76-6656/1.3.6.1.4.1.14519.5.2.1.1188.4001.313245752188924211777085602901/1.3.6.1.4.1.14519.5.2.1.1188.4001.175702182191848905428876073118
TCGA-GBM	TCGA-76	TCGA-76-6657	flair	M		90	T2_AX_FLAIR	501	6.29841E+15	6/11/2001	Philips Medical Systems	MR1	Achieva	IR	2D	5	11000	125	2850	127.7817	3	6	90	0.985684812	28	336	249	0.68452382087707\0.68452382087707	TCGA-76-6657/1.3.6.1.4.1.14519.5.2.1.1188.4001.114621594146207945121756272697/1.3.6.1.4.1.14519.5.2.1.1188.4001.281208541851434233735133229295
TCGA-GBM	TCGA-76	TCGA-76-6657	t1Gd	M		90	T1_AX_SE	1301	6.29841E+15	6/11/2001	Philips Medical Systems	MR1	Achieva	SE	2D	5	481.14	10		127.7817	3	7.5	90	3.200000048	22	284	227	0.71875\0.71875	TCGA-76-6657/1.3.6.1.4.1.14519.5.2.1.1188.4001.114621594146207945121756272697/1.3.6.1.4.1.14519.5.2.1.1188.4001.139759446384249479928559633803
TCGA-GBM	TCGA-76	TCGA-76-6657	t1	M		90	T1_AX__SE	901	6.29841E+15	6/11/2001	Philips Medical Systems	MR1	Achieva	SE	2D	5	423.8076	10		127.7817	3	7.5	90	3.199997187	24	316	254	0.68452382087707\0.68452382087707	TCGA-76-6657/1.3.6.1.4.1.14519.5.2.1.1188.4001.114621594146207945121756272697/1.3.6.1.4.1.14519.5.2.1.1188.4001.133273809379709831292592700914
TCGA-GBM	TCGA-76	TCGA-76-6657	t2	M		90	T2W_TSE	701	6.29841E+15	6/11/2001	Philips Medical Systems	MR1	Achieva	SE	2D	5	3000	105		127.7817	3	6	90	2.275380373	26	420	380	0.41071429848670\0.41071429848670	TCGA-76-6657/1.3.6.1.4.1.14519.5.2.1.1188.4001.114621594146207945121756272697/1.3.6.1.4.1.14519.5.2.1.1188.4001.231168282285866175377263571095
TCGA-GBM	TCGA-76	TCGA-76-6661	flair	M		70	T2_AX_FLAIR	701	9.0359E+15	12/8/2001	Philips Medical Systems	MR1	Achieva	IR	2D	5	11000	125	2850	127.7744	3	6	90	0.984946549	28	320	218	0.6875\0.6875	TCGA-76-6661/1.3.6.1.4.1.14519.5.2.1.1188.4001.102058737511198476066014834840/1.3.6.1.4.1.14519.5.2.1.1188.4001.281066854198433689922490047868
TCGA-GBM	TCGA-76	TCGA-76-6661	t1Gd	M		70	T1_SAG_SE	1501	9.0359E+15	12/8/2001	Philips Medical Systems	MR1	Achieva	SE	2D	5	400	10		127.7744	3	7.5	90	1.644433618	16	256	219	0.9375\0.9375	TCGA-76-6661/1.3.6.1.4.1.14519.5.2.1.1188.4001.102058737511198476066014834840/1.3.6.1.4.1.14519.5.2.1.1188.4001.474873019581159388054539531313
TCGA-GBM	TCGA-76	TCGA-76-6661	t1	M		70	T1_AX__SE	1101	9.0359E+15	12/8/2001	Philips Medical Systems	MR1	Achieva	SE	2D	5	423.8076	10.2		127.7744	3	7.5	90	3.199997187	24	304	242	0.6875\0.6875	TCGA-76-6661/1.3.6.1.4.1.14519.5.2.1.1188.4001.102058737511198476066014834840/1.3.6.1.4.1.14519.5.2.1.1188.4001.279952068981139943403600145423
TCGA-GBM	TCGA-76	TCGA-76-6661	t2	M		70	T2W_TSE	901	9.0359E+15	12/8/2001	Philips Medical Systems	MR1	Achieva	SE	2D	5	3000	105		127.7744	3	6	90	3.033840179	24	400	355	0.4296875\0.4296875	TCGA-76-6661/1.3.6.1.4.1.14519.5.2.1.1188.4001.102058737511198476066014834840/1.3.6.1.4.1.14519.5.2.1.1188.4001.270839976577326958300328675313
TCGA-GBM	TCGA-76	TCGA-76-6662	flair	M		95	T2_AX_FLAIR	501	1.52941E+15	12/13/2001	Philips Medical Systems	MR1	Achieva	IR	2D	5	11000	125	2850	127.7742	3	6	90	0.906080961	27	348	262	0.68181818723678\0.68181818723678	TCGA-76-6662/1.3.6.1.4.1.14519.5.2.1.1188.4001.313762558732585076631143086043/1.3.6.1.4.1.14519.5.2.1.1188.4001.248075422254562638743658647594
TCGA-GBM	TCGA-76	TCGA-76-6662	t1Gd	M		95	T1_AX_SE	1001	1.52941E+15	12/13/2001	Philips Medical Systems	MR1	Achieva	SE	2D	5	409.404	10		127.7742	3	7	90	3.200001001	24	296	239	0.75\0.75	TCGA-76-6662/1.3.6.1.4.1.14519.5.2.1.1188.4001.313762558732585076631143086043/1.3.6.1.4.1.14519.5.2.1.1188.4001.187826247221510809421836351871
TCGA-GBM	TCGA-76	TCGA-76-6662	t1	M		95	T1_AX__SE	801	1.52941E+15	12/13/2001	Philips Medical Systems	MR1	Achieva	SE	2D	5	375	10		127.7742	3	7	90	2.631093741	24	332	266	0.68181818723678\0.68181818723678	TCGA-76-6662/1.3.6.1.4.1.14519.5.2.1.1188.4001.313762558732585076631143086043/1.3.6.1.4.1.14519.5.2.1.1188.4001.998300758126981185243547729186
TCGA-GBM	TCGA-76	TCGA-76-6662	t2	M		95	T2W_TSE	701	1.52941E+15	12/13/2001	Philips Medical Systems	MR1	Achieva	SE	2D	5	3318.263	105		127.7742	3	6	90	3.199999571	27	436	401	0.42857143282890\0.42857143282890	TCGA-76-6662/1.3.6.1.4.1.14519.5.2.1.1188.4001.313762558732585076631143086043/1.3.6.1.4.1.14519.5.2.1.1188.4001.152280069325527575285353872757
TCGA-GBM	TCGA-76	TCGA-76-6663	flair	F		74	T2_AX_FLAIR	501	2.30791E+15	12/27/2001	Philips Medical Systems	MR1	Achieva	IR	2D	5	11000	125	2850	127.7735	3	6	90	0.984931111	28	320	218	0.6875\0.6875	TCGA-76-6663/1.3.6.1.4.1.14519.5.2.1.1188.4001.461523921338830081291431565499/1.3.6.1.4.1.14519.5.2.1.1188.4001.178083867370599982819220236278
TCGA-GBM	TCGA-76	TCGA-76-6663	t1Gd	F		74	T1_AX_SE	1001	2.30791E+15	12/27/2001	Philips Medical Systems	MR1	Achieva	SE	2D	5	586.993	10		127.7735	3	7.5	90	3.199999809	22	272	217	0.76388889551162\0.76388889551162	TCGA-76-6663/1.3.6.1.4.1.14519.5.2.1.1188.4001.461523921338830081291431565499/1.3.6.1.4.1.14519.5.2.1.1188.4001.166315135512753068373556679698
TCGA-GBM	TCGA-76	TCGA-76-6663	t1	F		74	T1_AX__SE	801	2.30791E+15	12/27/2001	Philips Medical Systems	MR1	Achieva	SE	2D	5	388.4903	10		127.7735	3	7.5	90	3.199997187	22	304	242	0.6875\0.6875	TCGA-76-6663/1.3.6.1.4.1.14519.5.2.1.1188.4001.461523921338830081291431565499/1.3.6.1.4.1.14519.5.2.1.1188.4001.106189376158731728515656074602
TCGA-GBM	TCGA-76	TCGA-76-6663	t2	F		74	T2W_TSE	701	2.30791E+15	12/27/2001	Philips Medical Systems	MR1	Achieva	SE	2D	5	2555.412	105		127.7735	3	6	90	3.200000048	24	400	374	0.4296875\0.4296875	TCGA-76-6663/1.3.6.1.4.1.14519.5.2.1.1188.4001.461523921338830081291431565499/1.3.6.1.4.1.14519.5.2.1.1188.4001.185350532654453205286429576409
TCGA-GBM	TCGA-76	TCGA-76-6664	t1	F		47	T1W_3D_stryker	301	2.68045E+15	1/10/2002	Philips Medical Systems	PHILIPS-DE02D8D	Achieva	GR	3D	1	8.0406	3.746		127.793	3	1	8	0.016735241	167	300	300	0.75\0.75	TCGA-76-6664/1.3.6.1.4.1.14519.5.2.1.1188.4001.280508857811965887839758381790/1.3.6.1.4.1.14519.5.2.1.1188.4001.182497160692414636856926373398
TCGA-GBM	TCGA-76	TCGA-76-6664	flair	F		47	T2_AX_FLAIR	601	2.68045E+15	1/10/2002	Philips Medical Systems	PHILIPS-DC8D0AA	Achieva	IR	2D	5	6000	120	2000	63.90021	1.5	6	90	1.003544927	28	256	161	0.859375\0.859375	TCGA-76-6664/1.3.6.1.4.1.14519.5.2.1.1188.4001.280508857811965887839758381790/1.3.6.1.4.1.14519.5.2.1.1188.4001.172605629671945162895985475280
TCGA-GBM	TCGA-76	TCGA-76-6664	t1Gd	F		47	T1_SE_POST	1201	2.68045E+15	1/10/2002	Philips Medical Systems	PHILIPS-DC8D0AA	Achieva	SE	2D	5	589.512	12		63.90021	1.5	7	69	1.466690779	22	256	204	0.859375\0.859375	TCGA-76-6664/1.3.6.1.4.1.14519.5.2.1.1188.4001.280508857811965887839758381790/1.3.6.1.4.1.14519.5.2.1.1188.4001.302763847255950328481343215267
TCGA-GBM	TCGA-76	TCGA-76-6664	t2	F		47	T2-WHOLE_BRAIN	801	2.68045E+15	1/10/2002	Philips Medical Systems	PHILIPS-DC8D0AA	Achieva	SE	2D	5	4530.271	100		63.90021	1.5	7.5	90	1.586941838	22	256	225	0.41015625\0.41015625	TCGA-76-6664/1.3.6.1.4.1.14519.5.2.1.1188.4001.280508857811965887839758381790/1.3.6.1.4.1.14519.5.2.1.1188.4001.101392134704233686294499449068
TCGA-LGG	TCGA-CS	TCGA-CS-4941	flair	M	67	86.183	AXIAL_FLAIR	5	2.6496E+15	9/9/1996	GE MEDICAL SYSTEMS	mr1OW	GENESIS_SIGNA	IR	2D	5	10002	157.5	2500	6.39E+08	1.5	6	90	0.027182	28	256	192	0.8593798876\0.859375	TCGA-CS-4941/1.3.6.1.4.1.14519.5.2.1.1188.4001.128551799994885838421577683863/1.3.6.1.4.1.14519.5.2.1.1188.4001.311513693456544543531535133857
TCGA-LGG	TCGA-CS	TCGA-CS-4941	t1Gd	M	67	86.183	*AX_3D_FOR_STRYKER_PROTOCOL	9	2.6496E+15	9/9/1996	GE MEDICAL SYSTEMS	mr1OW	GENESIS_SIGNA	GR	3D	1.5	7.68	1.692		6.39E+08	1.5	1.5	30	0.032676	124	256	192	0.9375\0.9375	TCGA-CS-4941/1.3.6.1.4.1.14519.5.2.1.1188.4001.128551799994885838421577683863/1.3.6.1.4.1.14519.5.2.1.1188.4001.211133122498306244529495926656
TCGA-LGG	TCGA-CS	TCGA-CS-4941	t1	M	67	86.183	AXIAL_T1	2	2.6496E+15	9/9/1996	GE MEDICAL SYSTEMS	mr1OW	GENESIS_SIGNA	SE	2D	5	600	9		6.39E+08	1.5	7.5	90	0.066078	23	256	192	0.7812440991\0.78125	TCGA-CS-4941/1.3.6.1.4.1.14519.5.2.1.1188.4001.128551799994885838421577683863/1.3.6.1.4.1.14519.5.2.1.1188.4001.132691411436475486878420509309
TCGA-LGG	TCGA-CS	TCGA-CS-4941	t2	M	67	86.183	AXIAL_T2	3	2.6496E+15	9/9/1996	GE MEDICAL SYSTEMS	mr1OW	GENESIS_SIGNA	SE	2D	5	3000	30		6.39E+08	1.5	7.5	90	0.022943	50	256	192	0.8593685627\0.859375	TCGA-CS-4941/1.3.6.1.4.1.14519.5.2.1.1188.4001.128551799994885838421577683863/1.3.6.1.4.1.14519.5.2.1.1188.4001.724227449850990988403354297278
TCGA-LGG	TCGA-CS	TCGA-CS-4942	flair	F		15	T2_AX_FLAIR	501	3.05011E+15	2/22/1997	Philips Medical Systems	MR3T	Intera Achieva	IR		5	11000	125	2800	127.7969	3	6	90		24	272	189	0.69444441795349\0.69444441795349	TCGA-CS-4942/1.3.6.1.4.1.14519.5.2.1.1188.4001.205805171270283233290247304689/1.3.6.1.4.1.14519.5.2.1.1188.4001.203315309793102340695695217917
TCGA-LGG	TCGA-CS	TCGA-CS-4942	t1Gd	F		15	TI_AX_SE	801	3.05011E+15	2/22/1997	Philips Medical Systems	MR3T	Intera Achieva	SE		5	500.0002	10		127.7968	3	7.5	90		20	256	179	0.8984375\0.8984375	TCGA-CS-4942/1.3.6.1.4.1.14519.5.2.1.1188.4001.205805171270283233290247304689/1.3.6.1.4.1.14519.5.2.1.1188.4001.147319190030218663096857357676
TCGA-LGG	TCGA-CS	TCGA-CS-4942	t1	F		15	T1_AX__SE	301	3.05011E+15	2/22/1997	Philips Medical Systems	MR3T	Intera Achieva	SE		5	500.0002	10		127.7969	3	7.5	90		20	256	205	0.8984375\0.8984375	TCGA-CS-4942/1.3.6.1.4.1.14519.5.2.1.1188.4001.205805171270283233290247304689/1.3.6.1.4.1.14519.5.2.1.1188.4001.122619603792498033279228114936
TCGA-LGG	TCGA-CS	TCGA-CS-4942	t2	F		15	DUAL_AX_SE	401	3.05011E+15	2/22/1997	Philips Medical Systems	MR3T	Intera Achieva	SE		5	3187.174	20		127.7969	3	7.5	90		48	256	192	0.50925928354263\0.50925928354263	TCGA-CS-4942/1.3.6.1.4.1.14519.5.2.1.1188.4001.205805171270283233290247304689/1.3.6.1.4.1.14519.5.2.1.1188.4001.309224177898458617251995007653
TCGA-LGG	TCGA-CS	TCGA-CS-4943	flair	M	37	91	BRAIN_STRYKER/FLAIR	5	7.24147E+15	9/2/2000	SIEMENS	mrscan	MAGNETOM VISION	IR	2D	5	9000	105	2340	63.62082	1.493806	2.499996	180	0	101	512	140	008.593750E-01\08.593750E-01	TCGA-CS-4943/1.3.6.1.4.1.14519.5.2.1.1188.4001.340234692744555909933537052921/1.3.6.1.4.1.14519.5.2.1.1188.4001.147804566525382153225080630011
TCGA-LGG	TCGA-CS	TCGA-CS-4943	t1Gd	M	37	91	BRAIN_STRYKER/AX-RAGE-STRY	7	7.24147E+15	9/2/2000	SIEMENS	mrscan	MAGNETOM VISION	IR	3D	1.5	15	7	300	63.62078	1.493806		15	0	301	512	134	005.078125E-01\05.078125E-01	TCGA-CS-4943/1.3.6.1.4.1.14519.5.2.1.1188.4001.340234692744555909933537052921/1.3.6.1.4.1.14519.5.2.1.1188.4001.123016478892109436983134203477
TCGA-LGG	TCGA-CS	TCGA-CS-4943	t1	M	37	91	BRAIN_STRYKER/T1_SAG	2	7.24147E+15	9/2/2000	SIEMENS	mrscan	MAGNETOM VISION	RM	2D	5	400	12		63.62078	1.493806	2	70	0	21	512	179	009.375000E-01\09.375000E-01	TCGA-CS-4943/1.3.6.1.4.1.14519.5.2.1.1188.4001.340234692744555909933537052921/1.3.6.1.4.1.14519.5.2.1.1188.4001.290529229678888668035398419158
TCGA-LGG	TCGA-CS	TCGA-CS-4943	t2	M	37	91	BRAIN_STRYKER/T2_AX_DOUBLE	4	7.24147E+15	9/2/2000	SIEMENS	mrscan	MAGNETOM VISION	RM	2D	5	2020	20		63.62078	1.493806	2.499996	90	0	81	512	115	004.296875E-01\04.296875E-01	TCGA-CS-4943/1.3.6.1.4.1.14519.5.2.1.1188.4001.340234692744555909933537052921/1.3.6.1.4.1.14519.5.2.1.1188.4001.287263919814542538289235456512
TCGA-LGG	TCGA-CS	TCGA-CS-4944	flair	M	50	77	BRAIN_STRYKER/FLAIR	5	2.19794E+15	2/8/2001	SIEMENS	mrscan	MAGNETOM VISION	IR	2D	5	9000	105	2340	63.62075	1.493806	2.5	180	0	103	512	140	008.593750E-01\08.593750E-01	TCGA-CS-4944/1.3.6.1.4.1.14519.5.2.1.1188.4001.129145242695024660859453065472/1.3.6.1.4.1.14519.5.2.1.1188.4001.203804375049412380660273335819
TCGA-LGG	TCGA-CS	TCGA-CS-4944	t1Gd	M	50	77	BRAIN_STRYKER/AX-RAGE-STRY	8	2.19794E+15	2/8/2001	SIEMENS	mrscan	MAGNETOM VISION	IR	3D	1.5	15	7	300	63.62066	1.493806		15	0	323	512	256	005.078125E-01\05.078125E-01	TCGA-CS-4944/1.3.6.1.4.1.14519.5.2.1.1188.4001.129145242695024660859453065472/1.3.6.1.4.1.14519.5.2.1.1188.4001.339210546101357716851441307311
TCGA-LGG	TCGA-CS	TCGA-CS-4944	t1	M	50	77	BRAIN_STRYKER/T1_AXIAL	3	2.19794E+15	2/8/2001	SIEMENS	mrscan	MAGNETOM VISION	RM	2D	5	540	12		63.62067	1.493806	2.5	70	0	43	512	134	008.593750E-01\08.593750E-01	TCGA-CS-4944/1.3.6.1.4.1.14519.5.2.1.1188.4001.129145242695024660859453065472/1.3.6.1.4.1.14519.5.2.1.1188.4001.207297865468602642550067713550
TCGA-LGG	TCGA-CS	TCGA-CS-4944	t2	M	50	77	BRAIN_NON_CONTRAST/FL_2D_A	6	2.19794E+15	2/8/2001	SIEMENS	mrscan	MAGNETOM VISION	RM	2D	5	800	26		63.62075	1.493806	2.499996	15	0	123	512	96	008.593750E-01\08.593750E-01	TCGA-CS-4944/1.3.6.1.4.1.14519.5.2.1.1188.4001.129145242695024660859453065472/1.3.6.1.4.1.14519.5.2.1.1188.4001.162789104395443584625385186701
TCGA-LGG	TCGA-CS	TCGA-CS-5393	flair	M	39	100	BRAIN_CONTRAST/FLAIR	4	8.62427E+15	6/6/1999	SIEMENS	mrscan	MAGNETOM VISION	IR	2D	5	9000	105	2340	63.61585	1.493806	2.5	180	0	61	512	112	008.593750E-01\08.593750E-01	TCGA-CS-5393/1.3.6.1.4.1.14519.5.2.1.1188.4001.270866039761941345997853128540/1.3.6.1.4.1.14519.5.2.1.1188.4001.132954609680080850667151992756
TCGA-LGG	TCGA-CS	TCGA-CS-5393	t1Gd	M	39	100	BRAIN_STRYKER/AX-RAGE-STRY	8	8.62427E+15	6/6/1999	SIEMENS	mrscan	MAGNETOM VISION	IR	3D	1.5	15	7	300	63.61583	1.493806		15	0	321	512	134	005.078125E-01\05.078125E-01	TCGA-CS-5393/1.3.6.1.4.1.14519.5.2.1.1188.4001.270866039761941345997853128540/1.3.6.1.4.1.14519.5.2.1.1188.4001.229538992522004896365823528826
TCGA-LGG	TCGA-CS	TCGA-CS-5393	t1	M	39	100	BRAIN_CONTRAST/T1_AXIAL	3	8.62427E+15	6/6/1999	SIEMENS	mrscan	MAGNETOM VISION	RM	2D	5	600	14		63.61582	1.493806	2.500004	70	0	41	512	115	008.593750E-01\08.593750E-01	TCGA-CS-5393/1.3.6.1.4.1.14519.5.2.1.1188.4001.270866039761941345997853128540/1.3.6.1.4.1.14519.5.2.1.1188.4001.290034506973629211391468552664
TCGA-LGG	TCGA-CS	TCGA-CS-5393	t2	M	39	100	BRAIN_CONTRAST/hemosiderin	6	8.62427E+15	6/6/1999	SIEMENS	mrscan	MAGNETOM VISION	RM	2D	5	800	26		63.61585	1.493806	2.5	15	0	121	512	134	008.593750E-01\08.593750E-01	TCGA-CS-5393/1.3.6.1.4.1.14519.5.2.1.1188.4001.270866039761941345997853128540/1.3.6.1.4.1.14519.5.2.1.1188.4001.335851554045791851017805707229
TCGA-LGG	TCGA-CS	TCGA-CS-5395	flair	M	43	91	BRAIN_CONTRAST/FLAIR	3	1.65611E+15	10/4/1998	SIEMENS	mrscan	MAGNETOM VISION	IR	2D	5	9000	105	2340	63.61564	1.493806	2.5	180	0	41	512	140	008.593750E-01\08.593750E-01	TCGA-CS-5395/1.3.6.1.4.1.14519.5.2.1.1188.4001.202878570115416620116241845955/1.3.6.1.4.1.14519.5.2.1.1188.4001.187917860252441163272978739749
TCGA-LGG	TCGA-CS	TCGA-CS-5395	t1Gd	M	43	91	BRAIN_STRYKER/AX-RAGE-STRY	13	1.65611E+15	10/4/1998	SIEMENS	mrscan	MAGNETOM VISION	IR	3D	1.5	15	7	300	63.6156	1.493806		15	0	332	512	179	004.687500E-01\04.687500E-01	TCGA-CS-5395/1.3.6.1.4.1.14519.5.2.1.1188.4001.202878570115416620116241845955/1.3.6.1.4.1.14519.5.2.1.1188.4001.174104235571112005602820353561
TCGA-LGG	TCGA-CS	TCGA-CS-5395	t1	M	43	91	BRAIN_STRYKER/T1_AXIAL	10	1.65611E+15	10/4/1998	SIEMENS	mrscan	MAGNETOM VISION	RM	2D	5	540	12		63.61564	1.493806	2.5	70	0	92	512	134	008.593750E-01\08.593750E-01	TCGA-CS-5395/1.3.6.1.4.1.14519.5.2.1.1188.4001.202878570115416620116241845955/1.3.6.1.4.1.14519.5.2.1.1188.4001.977609850409468267249989944139
TCGA-LGG	TCGA-CS	TCGA-CS-5395	t2	M	43	91	BRAIN_PITUITARY/PD_T2_AXIA	11	1.65611E+15	10/4/1998	SIEMENS	mrscan	MAGNETOM VISION	RM	2D	5	3200	15		63.61564	1.493806	2.5	180	0	132	1024	196	004.296875E-01\04.296875E-01	TCGA-CS-5395/1.3.6.1.4.1.14519.5.2.1.1188.4001.202878570115416620116241845955/1.3.6.1.4.1.14519.5.2.1.1188.4001.154523005230294777772339327074
TCGA-LGG	TCGA-CS	TCGA-CS-5396	flair	F		81	T2_AX_FLAIR	601	7.91565E+15	3/2/2001	Philips Medical Systems	MR1	Achieva	IR	2D	5	11000	125	2850	127.7858	3	6	90	0.984979868	28	320	250	0.6875\0.6875	TCGA-CS-5396/1.3.6.1.4.1.14519.5.2.1.1188.4001.866856253970500879015300047605/1.3.6.1.4.1.14519.5.2.1.1188.4001.170311900627152897231718498890
TCGA-LGG	TCGA-CS	TCGA-CS-5396	t1Gd	F		81	T1_AX_SE	1101	7.91565E+15	3/2/2001	Philips Medical Systems	MR1	Achieva	SE	2D	5	640.356	10		127.7858	3	7.5	90	3.199999571	24	272	217	0.76388889551162\0.76388889551162	TCGA-CS-5396/1.3.6.1.4.1.14519.5.2.1.1188.4001.866856253970500879015300047605/1.3.6.1.4.1.14519.5.2.1.1188.4001.250829490408448799557825354608
TCGA-LGG	TCGA-CS	TCGA-CS-5396	t1	F		81	T1_AX__SE	1001	7.91565E+15	3/2/2001	Philips Medical Systems	MR1	Achieva	SE	2D	5	423.8076	10		127.7858	3	7.5	90	3.199997187	24	304	243	0.6875\0.6875	TCGA-CS-5396/1.3.6.1.4.1.14519.5.2.1.1188.4001.866856253970500879015300047605/1.3.6.1.4.1.14519.5.2.1.1188.4001.121538176450529977959006375237
TCGA-LGG	TCGA-CS	TCGA-CS-5396	t2	F		81	T2W_TSE	701	7.91565E+15	3/2/2001	Philips Medical Systems	MR1	Achieva	SE	2D	5	3000	105		127.7858	3	6	90	3.033840179	24	400	380	0.4296875\0.4296875	TCGA-CS-5396/1.3.6.1.4.1.14519.5.2.1.1188.4001.866856253970500879015300047605/1.3.6.1.4.1.14519.5.2.1.1188.4001.186406456919519776851284170754
TCGA-LGG	TCGA-CS	TCGA-CS-5397	flair	F		49	T2_AX_FLAIR	501	8.75582E+15	3/15/2001	Philips Medical Systems	MR1	Achieva	IR	2D	5	11000	125	2850	127.7853	3	6	90	0.985324383	28	336	255	0.68452382087707\0.68452382087707	TCGA-CS-5397/1.3.6.1.4.1.14519.5.2.1.1188.4001.157283642653722420546464271991/1.3.6.1.4.1.14519.5.2.1.1188.4001.186076363067152633547598779834
TCGA-LGG	TCGA-CS	TCGA-CS-5397	t1Gd	F		49	T1W_3D_stryker	1101	8.75582E+15	3/15/2001	Philips Medical Systems	MR1	Achieva	GR	3D	3	8.3691	4.1335		127.7853	3	1.5	8	0.276564628	120	272	240	0.83333331346511\0.83333331346511	TCGA-CS-5397/1.3.6.1.4.1.14519.5.2.1.1188.4001.157283642653722420546464271991/1.3.6.1.4.1.14519.5.2.1.1188.4001.309416896241519723753279836949
TCGA-LGG	TCGA-CS	TCGA-CS-5397	t1	F		49	T1_AX__SE	901	8.75582E+15	3/15/2001	Philips Medical Systems	MR1	Achieva	SE	2D	5	399.9996	10		127.7853	3	7.5	90	3.107922792	22	316	255	0.68452382087707\0.68452382087707	TCGA-CS-5397/1.3.6.1.4.1.14519.5.2.1.1188.4001.157283642653722420546464271991/1.3.6.1.4.1.14519.5.2.1.1188.4001.148929021964876442187111599680
TCGA-LGG	TCGA-CS	TCGA-CS-5397	t2	F		49	T2W_TSE	601	8.75582E+15	3/15/2001	Philips Medical Systems	MR1	Achieva	SE	2D	5	3318.263	105		127.7853	3	6	90	3.199999571	28	420	380	0.41071429848670\0.41071429848670	TCGA-CS-5397/1.3.6.1.4.1.14519.5.2.1.1188.4001.157283642653722420546464271991/1.3.6.1.4.1.14519.5.2.1.1188.4001.993332431135153833493944928693
TCGA-LGG	TCGA-CS	TCGA-CS-6186	flair	M		85	T2_AX_FLAIR	401	4.696E+15	6/1/2000	Philips Medical Systems	PHILIPS-8D9B7F3	Achieva	IR	2D	5	6000	120	2000	63.90019	1.5	6	90	1.003544927	30	256	161	0.859375\0.859375	TCGA-CS-6186/1.3.6.1.4.1.14519.5.2.1.1188.4001.242366823329087276501028197608/1.3.6.1.4.1.14519.5.2.1.1188.4001.206296150819865714212366072445
TCGA-LGG	TCGA-CS	TCGA-CS-6186	t1Gd	M		85	STRYKER	1101	4.696E+15	6/1/2000	Philips Medical Systems	PHILIPS-8D9B7F3	Achieva	GR	3D	3	25	4.5994		63.90019	1.5	1.5	30	0.319924802	118	256	244	0.9375\0.9375	TCGA-CS-6186/1.3.6.1.4.1.14519.5.2.1.1188.4001.242366823329087276501028197608/1.3.6.1.4.1.14519.5.2.1.1188.4001.171955373280735242099311227064
TCGA-LGG	TCGA-CS	TCGA-CS-6186	t1	M		85	T1_SE_PRE	901	4.696E+15	6/1/2000	Philips Medical Systems	PHILIPS-8D9B7F3	Achieva	SE	2D	5	413.944	12		63.90019	1.5	7	69	1.721659899	25	256	204	0.859375\0.859375	TCGA-CS-6186/1.3.6.1.4.1.14519.5.2.1.1188.4001.242366823329087276501028197608/1.3.6.1.4.1.14519.5.2.1.1188.4001.269684106183325571291783916483
TCGA-LGG	TCGA-CS	TCGA-CS-6186	t2	M		85	T2-WHOLE_BRAIN	501	4.696E+15	6/1/2000	Philips Medical Systems	PHILIPS-8D9B7F3	Achieva	SE	2D	5	4938.149	100		63.90019	1.5	7.5	90	1.588215947	24	256	225	0.41015625\0.41015625	TCGA-CS-6186/1.3.6.1.4.1.14519.5.2.1.1188.4001.242366823329087276501028197608/1.3.6.1.4.1.14519.5.2.1.1188.4001.664999071910196574827570132557
TCGA-LGG	TCGA-CS	TCGA-CS-6188	flair	M		113	T2_AX_FLAIR	501	2.21267E+15	8/12/2001	Philips Medical Systems	MR1	Achieva	IR	2D	5	11000	125	2850	127.7793	3	6	90	0.984957218	28	320	250	0.6875\0.6875	TCGA-CS-6188/1.3.6.1.4.1.14519.5.2.1.1188.4001.203713524649110849540001495530/1.3.6.1.4.1.14519.5.2.1.1188.4001.102732308226606493016714027780
TCGA-LGG	TCGA-CS	TCGA-CS-6188	t1Gd	M		113	COR_MPR	1002	2.21267E+15	8/12/2001	Philips Medical Systems		Achieva	GR	3D	1	7.6244	3.5515		127.7793	3	1	8	0.246233359	200	300	300	0.75\0.75	TCGA-CS-6188/1.3.6.1.4.1.14519.5.2.1.1188.4001.203713524649110849540001495530/1.3.6.1.4.1.14519.5.2.1.1188.4001.255282194301702091567144865881
TCGA-LGG	TCGA-CS	TCGA-CS-6188	t1	M		113	T1W___SE	401	2.21267E+15	8/12/2001	Philips Medical Systems	MR1	Achieva	SE	2D	5	400	10		127.7792	3	7.5	90	1.644433618	16	256	205	0.9375\0.9375	TCGA-CS-6188/1.3.6.1.4.1.14519.5.2.1.1188.4001.203713524649110849540001495530/1.3.6.1.4.1.14519.5.2.1.1188.4001.663981468162513582180008232641
TCGA-LGG	TCGA-CS	TCGA-CS-6188	t2	M		113	T2W_TSE	701	2.21267E+15	8/12/2001	Philips Medical Systems	MR1	Achieva	SE	2D	5	3000	105		127.7793	3	6	90	3.033840179	24	400	380	0.4296875\0.4296875	TCGA-CS-6188/1.3.6.1.4.1.14519.5.2.1.1188.4001.203713524649110849540001495530/1.3.6.1.4.1.14519.5.2.1.1188.4001.237410497056644642573784487223
TCGA-LGG	TCGA-CS	TCGA-CS-6290	flair	M	31	113	BRAIN_CONTRAST/FLAIR	11	3.38024E+15	9/17/2000	SIEMENS	mrscan	MAGNETOM VISION	IR	2D	5	9000	105	2340	63.62079	1.493806	2.5	180	0	351	512	112	008.593750E-01\08.593750E-01	TCGA-CS-6290/1.3.6.1.4.1.14519.5.2.1.1188.4001.103822155980636301332134926774/1.3.6.1.4.1.14519.5.2.1.1188.4001.752531645793998918722907278370
TCGA-LGG	TCGA-CS	TCGA-CS-6290	t1Gd	M	31	113	BRAIN_CONTRAST/T1_CORONAL_	15	3.38024E+15	9/17/2000	SIEMENS	mrscan	MAGNETOM VISION	RM	2D	5	440	12		63.62079	1.493806	3	70	0	451	512	134	008.593750E-01\08.593750E-01	TCGA-CS-6290/1.3.6.1.4.1.14519.5.2.1.1188.4001.103822155980636301332134926774/1.3.6.1.4.1.14519.5.2.1.1188.4001.331156113378797656070442234457
TCGA-LGG	TCGA-CS	TCGA-CS-6290	t1	M	31	113	BRAIN_CONTRAST/T1_AXIAL	7	3.38024E+15	9/17/2000	SIEMENS	mrscan	MAGNETOM VISION	RM	2D	5	600	14		63.62079	1.493806	2.500004	70	0	309	512	115	008.593750E-01\08.593750E-01	TCGA-CS-6290/1.3.6.1.4.1.14519.5.2.1.1188.4001.103822155980636301332134926774/1.3.6.1.4.1.14519.5.2.1.1188.4001.184518783244690052738829109319
TCGA-LGG	TCGA-CS	TCGA-CS-6290	t2	M	31	113	BRAIN_CONTRAST/hemosiderin	13	3.38024E+15	9/17/2000	SIEMENS	mrscan	MAGNETOM VISION	RM	2D	5	800	26		63.62079	1.493806	2.5	15	0	411	512	134	008.593750E-01\08.593750E-01	TCGA-CS-6290/1.3.6.1.4.1.14519.5.2.1.1188.4001.103822155980636301332134926774/1.3.6.1.4.1.14519.5.2.1.1188.4001.296212542270973986107566466837
TCGA-LGG	TCGA-CS	TCGA-CS-6665	flair	F		68	T2_AX_FLAIR	501	1.29453E+15	8/17/2001	Philips Medical Systems	MR1	Achieva	IR	2D	5	11000	125	2850	127.7791	3	6	90	0.827241838	24	320	247	0.65476191043853\0.65476191043853	TCGA-CS-6665/1.3.6.1.4.1.14519.5.2.1.1188.4001.159768644950365298898163950114/1.3.6.1.4.1.14519.5.2.1.1188.4001.173192188712863545223866126615
TCGA-LGG	TCGA-CS	TCGA-CS-6665	t1Gd	F		68	T1_AX_SE	1101	1.29453E+15	8/17/2001	Philips Medical Systems	MR1	Achieva	SE	2D	5	600	10		127.7791	3	6	90	2.799360037	24	272	217	0.76388889551162\0.76388889551162	TCGA-CS-6665/1.3.6.1.4.1.14519.5.2.1.1188.4001.159768644950365298898163950114/1.3.6.1.4.1.14519.5.2.1.1188.4001.304734519877038894320972032214
TCGA-LGG	TCGA-CS	TCGA-CS-6665	t1	F		68	T1_AX__SE	901	1.29453E+15	8/17/2001	Philips Medical Systems	MR1	Achieva	SE	2D	5	423.8076	10		127.7791	3	6	90	3.199997187	24	304	243	0.6875\0.6875	TCGA-CS-6665/1.3.6.1.4.1.14519.5.2.1.1188.4001.159768644950365298898163950114/1.3.6.1.4.1.14519.5.2.1.1188.4001.291878956429584404970815668083
TCGA-LGG	TCGA-CS	TCGA-CS-6665	t2	F		68	T2W_TSE	701	1.29453E+15	8/17/2001	Philips Medical Systems	MR1	Achieva	SE	2D	5	3000	105		127.7791	3	6	90	3.033840179	24	400	365	0.4296875\0.4296875	TCGA-CS-6665/1.3.6.1.4.1.14519.5.2.1.1188.4001.159768644950365298898163950114/1.3.6.1.4.1.14519.5.2.1.1188.4001.120098837774084096221182816485
TCGA-LGG	TCGA-CS	TCGA-CS-6666	flair	M		83	T2_AX_FLAIR	701	5.29894E+15	11/9/2001	Philips Medical Systems	PHILIPS-DE02D8D	Achieva	IR	2D	5	11000	125	2850	127.7934	3	6	90	0.067167066	28	320	247	0.6875\0.6875	TCGA-CS-6666/1.3.6.1.4.1.14519.5.2.1.1188.4001.562796873094346571498215071602/1.3.6.1.4.1.14519.5.2.1.1188.4001.128846142536225521942208108756
TCGA-LGG	TCGA-CS	TCGA-CS-6666	t1Gd	M		83	T1W_3D_STRYKER	1001	5.29894E+15	11/9/2001	Philips Medical Systems	PHILIPS-DE02D8D	Achieva	GR	3D	1	7.6774	3.583		127.7934	3	1	8	0.017320534	200	300	300	0.75\0.75	TCGA-CS-6666/1.3.6.1.4.1.14519.5.2.1.1188.4001.562796873094346571498215071602/1.3.6.1.4.1.14519.5.2.1.1188.4001.160531909116163750684978784137
TCGA-LGG	TCGA-CS	TCGA-CS-6666	t1	M		83	T1_AX__SE	901	5.29894E+15	11/9/2001	Philips Medical Systems	PHILIPS-DE02D8D	Achieva	SE	2D	5	461.4519	10.3		127.7934	3	6	90	0.217081264	26	304	242	0.6875\0.6875	TCGA-CS-6666/1.3.6.1.4.1.14519.5.2.1.1188.4001.562796873094346571498215071602/1.3.6.1.4.1.14519.5.2.1.1188.4001.143563584178390497626882678879
TCGA-LGG	TCGA-CS	TCGA-CS-6666	t2	M		83	AXIAL__T2	801	5.29894E+15	11/9/2001	Philips Medical Systems	PHILIPS-DE02D8D	Achieva	SE	2D	5	3000	100		127.7934	3	6	90	0.196178049	26	384	324	0.4296875\0.4296875	TCGA-CS-6666/1.3.6.1.4.1.14519.5.2.1.1188.4001.562796873094346571498215071602/1.3.6.1.4.1.14519.5.2.1.1188.4001.309761309865874206034533043433
TCGA-LGG	TCGA-CS	TCGA-CS-6667	flair	F	39	58.967	AX_FLAIR	7	4.45891E+15	11/5/2001	GE MEDICAL SYSTEMS	GEMSOW	Signa HDxt	SE\IR	2D	5	10002	144.884	2500	63.84815	1.5	6	90	0.5505	26	288	192	0.4297\0.4297	TCGA-CS-6667/1.3.6.1.4.1.14519.5.2.1.1188.4001.190019357332393685119456096919/1.3.6.1.4.1.14519.5.2.1.1188.4001.144509347774443554281904614614
TCGA-LGG	TCGA-CS	TCGA-CS-6667	t1Gd	F	39	58.967	Ax_T1_FS_BRAIN_POST	9	4.45891E+15	11/5/2001	GE MEDICAL SYSTEMS	GEMSOW	Signa HDxt	SE	2D	5	483.336	12	0	63.84816	1.5	7.5	90	0.916982	20	256	160	0.4297\0.4297	TCGA-CS-6667/1.3.6.1.4.1.14519.5.2.1.1188.4001.190019357332393685119456096919/1.3.6.1.4.1.14519.5.2.1.1188.4001.270532183341952426047363273660
TCGA-LGG	TCGA-CS	TCGA-CS-6667	t1	F	39	58.967	Ax_T1	5	4.45891E+15	11/5/2001	GE MEDICAL SYSTEMS	GEMSOW	Signa HDxt	SE	2D	5	533.336	12	0	63.84815	1.5	7.5	90	1.51747	20	256	192	0.4297\0.4297	TCGA-CS-6667/1.3.6.1.4.1.14519.5.2.1.1188.4001.190019357332393685119456096919/1.3.6.1.4.1.14519.5.2.1.1188.4001.342053548701630640583211031183
TCGA-LGG	TCGA-CS	TCGA-CS-6667	t2	F	39	58.967	AX_FSE_T2	6	4.45891E+15	11/5/2001	GE MEDICAL SYSTEMS	GEMSOW	Signa HDxt	SE	2D	5	3000	91.52	0	63.84815	1.5	7.5	90	0.7956	20	320	224	0.4297\0.4297	TCGA-CS-6667/1.3.6.1.4.1.14519.5.2.1.1188.4001.190019357332393685119456096919/1.3.6.1.4.1.14519.5.2.1.1188.4001.118303589976037481805625826661
TCGA-LGG	TCGA-CS	TCGA-CS-6668	flair	F		83	T2_AX_FLAIR	501	2.03626E+15	10/25/2001	Philips Medical Systems	PHILIPS-DE02D8D	Achieva	IR	2D	5	11000	125	2850	127.7935	3	6	90	0.067193471	28	320	247	0.6875\0.6875	TCGA-CS-6668/1.3.6.1.4.1.14519.5.2.1.1188.4001.980162626547355442928625529896/1.3.6.1.4.1.14519.5.2.1.1188.4001.169889219500152307085456851345
TCGA-LGG	TCGA-CS	TCGA-CS-6668	t1Gd	F		83	T1W_3D_STRYKER	1101	2.03626E+15	10/25/2001	Philips Medical Systems	PHILIPS-DE02D8D	Achieva	GR	3D	1	8.1329	3.824		127.7934	3	1	8	0.017320534	170	300	300	0.75\0.75	TCGA-CS-6668/1.3.6.1.4.1.14519.5.2.1.1188.4001.980162626547355442928625529896/1.3.6.1.4.1.14519.5.2.1.1188.4001.114078538471828186024243211217
TCGA-LGG	TCGA-CS	TCGA-CS-6668	t1	F		83	T1W_3D_STRYKER	701	2.03626E+15	10/25/2001	Philips Medical Systems	PHILIPS-DE02D8D	Achieva	GR	3D	1	8.0072	3.736		127.7935	3	1	8	0.017320534	170	300	300	0.75\0.75	TCGA-CS-6668/1.3.6.1.4.1.14519.5.2.1.1188.4001.980162626547355442928625529896/1.3.6.1.4.1.14519.5.2.1.1188.4001.142459179818453226386988487183
TCGA-LGG	TCGA-CS	TCGA-CS-6668	t2	F		83	AXIAL__T2	601	2.03626E+15	10/25/2001	Philips Medical Systems	PHILIPS-DE02D8D	Achieva	SE	2D	4	3584.453	100		127.7936	3	5	90	0.214710966	33	384	324	0.4296875\0.4296875	TCGA-CS-6668/1.3.6.1.4.1.14519.5.2.1.1188.4001.980162626547355442928625529896/1.3.6.1.4.1.14519.5.2.1.1188.4001.323550053999956700006878511084
TCGA-LGG	TCGA-CS	TCGA-CS-6669	flair	F		68	T2_AX_FLAIR	501	3.05642E+15	1/2/2002	Philips Medical Systems	PHILIPS-DE02D8D	Achieva	IR	2D	5	11000	125	2850	127.7932	3	6	90	0.067156747	28	320	250	0.6875\0.6875	TCGA-CS-6669/1.3.6.1.4.1.14519.5.2.1.1188.4001.158950330763169507420209045633/1.3.6.1.4.1.14519.5.2.1.1188.4001.331668859120900125509759340193
TCGA-LGG	TCGA-CS	TCGA-CS-6669	t1Gd	F		68	T1_AX_SE	901	3.05642E+15	1/2/2002	Philips Medical Systems	PHILIPS-DE02D8D	Achieva	SE	2D	5	600.0005	10		127.7932	3	7.5	90	0.174959749	22	272	217	0.76388889551162\0.76388889551162	TCGA-CS-6669/1.3.6.1.4.1.14519.5.2.1.1188.4001.158950330763169507420209045633/1.3.6.1.4.1.14519.5.2.1.1188.4001.975564333883987761901816698053
TCGA-LGG	TCGA-CS	TCGA-CS-6669	t1	F		68	T1_AX__SE	801	3.05642E+15	1/2/2002	Philips Medical Systems	PHILIPS-DE02D8D	Achieva	SE	2D	5	388.4903	10.344		127.7932	3	7.5	90	0.218181506	22	304	243	0.6875\0.6875	TCGA-CS-6669/1.3.6.1.4.1.14519.5.2.1.1188.4001.158950330763169507420209045633/1.3.6.1.4.1.14519.5.2.1.1188.4001.270701452492080803456322167850
TCGA-LGG	TCGA-CS	TCGA-CS-6669	t2	F		68	T2W_TSE	701	3.05642E+15	1/2/2002	Philips Medical Systems	PHILIPS-DE02D8D	Achieva	SE	2D	5	3081.244	105		127.7932	3	6	90	0.218181595	26	400	380	0.4296875\0.4296875	TCGA-CS-6669/1.3.6.1.4.1.14519.5.2.1.1188.4001.158950330763169507420209045633/1.3.6.1.4.1.14519.5.2.1.1188.4001.478644020758691119117361200831
TCGA-LGG	TCGA-DU	TCGA-DU-5849	flair	U	48	90.718	AXIAL_FLAIR	8	1.61956E+15	4/5/1995	GE MEDICAL SYSTEMS	MR3T	SIGNA EXCITE	SE\IR	2D	2.5	10002	125.4	2250	127.7323	3	2.5	90	0.9008	72	320	224	0.4688\0.4688	TCGA-DU-5849/1.3.6.1.4.1.14519.5.2.1.4591.4003.109364397081642293014271520866/1.3.6.1.4.1.14519.5.2.1.4591.4003.216135639247172328000547905561
TCGA-LGG	TCGA-DU	TCGA-DU-5849	t1Gd	U	48	90.718	AX_T1_POST_GD_FLAIR	12	1.61956E+15	4/5/1995	GE MEDICAL SYSTEMS	MR3T	SIGNA EXCITE	SE\IR	2D	2.5	3236.34	6.356	1238	127.7323	3	2.5	90	1.7636	72	320	224	0.4688\0.4688	TCGA-DU-5849/1.3.6.1.4.1.14519.5.2.1.4591.4003.109364397081642293014271520866/1.3.6.1.4.1.14519.5.2.1.4591.4003.141732357113095863404589053586
TCGA-LGG	TCGA-DU	TCGA-DU-5849	t1	U	48	90.718	AX_T1_pre_gd	9	1.61956E+15	4/5/1995	GE MEDICAL SYSTEMS	MR3T	SIGNA EXCITE	SE\IR	2D	5	2152.82	6.356	883	127.7323	3	5	90	1.4365	38	320	224	0.4688\0.4688	TCGA-DU-5849/1.3.6.1.4.1.14519.5.2.1.4591.4003.109364397081642293014271520866/1.3.6.1.4.1.14519.5.2.1.4591.4003.326882926621335107573626721019
TCGA-LGG	TCGA-DU	TCGA-DU-5849	t2	U	48	90.718	AX_T2_FR-FSE_RF2_150	7	1.61956E+15	4/5/1995	GE MEDICAL SYSTEMS	MR3T	SIGNA EXCITE	SE	2D	5	4000	103.464	0	127.7323	3	5	90	1.3993	38	320	224	0.4688\0.4688	TCGA-DU-5849/1.3.6.1.4.1.14519.5.2.1.4591.4003.109364397081642293014271520866/1.3.6.1.4.1.14519.5.2.1.4591.4003.280912757798474012433274591281
TCGA-LGG	TCGA-DU	TCGA-DU-5851	flair	F	40	72.575	Ax_FLAIR_2.5mm_for_surgery	4	1.32058E+15	4/28/1995	GE MEDICAL SYSTEMS	MR01MROW	Signa HDxt	IR	2D	2.5	10002	149.544	2200	63.88583	1.5	2.5	90	0.039	79	256	192	0.9375\0.9375	TCGA-DU-5851/1.3.6.1.4.1.14519.5.2.1.4591.4003.129680596450908220610617702930/1.3.6.1.4.1.14519.5.2.1.4591.4003.121163298773025279216831005877
TCGA-LGG	TCGA-DU	TCGA-DU-5851	t1Gd	F	40	72.575	COR_T1_POST_GD	9	1.32058E+15	4/28/1995	GE MEDICAL SYSTEMS	MR01MROW	Signa HDxt	SE	2D	5	483.336	14	0	63.88583	1.5	5	90	0.0495152	48	256	192	0.9375\0.9375	TCGA-DU-5851/1.3.6.1.4.1.14519.5.2.1.4591.4003.129680596450908220610617702930/1.3.6.1.4.1.14519.5.2.1.4591.4003.304925718859314734932464300032
TCGA-LGG	TCGA-DU	TCGA-DU-5851	t1	F	40	72.575	Ax_T1_2.5mm_for_surgery	8	1.32058E+15	4/28/1995	GE MEDICAL SYSTEMS	MR01MROW	Signa HDxt	SE	2D	2.5	600	13	0	63.88583	1.5	2.5	90	0.054777	79	256	192	0.9375\0.9375	TCGA-DU-5851/1.3.6.1.4.1.14519.5.2.1.4591.4003.129680596450908220610617702930/1.3.6.1.4.1.14519.5.2.1.4591.4003.269931978354736840234304467989
TCGA-LGG	TCGA-DU	TCGA-DU-5851	t2	F	40	72.575	AXIAL_FSE	3	1.32058E+15	4/28/1995	GE MEDICAL SYSTEMS	MR01MROW	Signa HDxt	SE	2D	5	3516.67	23.688	0	63.88583	1.5	5	90	0.1355	80	256	192	0.9375\0.9375	TCGA-DU-5851/1.3.6.1.4.1.14519.5.2.1.4591.4003.129680596450908220610617702930/1.3.6.1.4.1.14519.5.2.1.4591.4003.198926562240285905428979227040
TCGA-LGG	TCGA-DU	TCGA-DU-5852	flair	F	61	85.275	AXIAL_FLAIR	7	8.68519E+15	7/9/1995	GE MEDICAL SYSTEMS	MR3T	SIGNA EXCITE	SE\IR	2D	2.5	10002	125.4	2250	127.7322	3	2.5	90	0.8357	70	320	224	0.4688\0.4688	TCGA-DU-5852/1.3.6.1.4.1.14519.5.2.1.4591.4003.149176124210739275471526262702/1.3.6.1.4.1.14519.5.2.1.4591.4003.750066108904337108875868387535
TCGA-LGG	TCGA-DU	TCGA-DU-5852	t1Gd	F	61	85.275	AX_T1_POST_GD_FLAIR	11	8.68519E+15	7/9/1995	GE MEDICAL SYSTEMS	MR3T	SIGNA EXCITE	SE\IR	2D	2.5	3207.73	6.356	1238	127.7322	3	2.5	90	1.6004	70	320	224	0.4688\0.4688	TCGA-DU-5852/1.3.6.1.4.1.14519.5.2.1.4591.4003.149176124210739275471526262702/1.3.6.1.4.1.14519.5.2.1.4591.4003.369433803261020392440363841253
TCGA-LGG	TCGA-DU	TCGA-DU-5852	t1	F	61	85.275	AX_T1_pre_gd	8	8.68519E+15	7/9/1995	GE MEDICAL SYSTEMS	MR3T	SIGNA EXCITE	SE\IR	2D	5	2208.66	6.356	883	127.7322	3	5	90	1.7437	36	320	224	0.4688\0.4688	TCGA-DU-5852/1.3.6.1.4.1.14519.5.2.1.4591.4003.149176124210739275471526262702/1.3.6.1.4.1.14519.5.2.1.4591.4003.421016340465864461716578061202
TCGA-LGG	TCGA-DU	TCGA-DU-5852	t2	F	61	85.275	AX_T2_FR-FSE_RF2_150	5	8.68519E+15	7/9/1995	GE MEDICAL SYSTEMS	MR3T	SIGNA EXCITE	SE	2D	5	4000	103.464	0	127.7322	3	5	90	1.1923	36	320	224	0.4688\0.4688	TCGA-DU-5852/1.3.6.1.4.1.14519.5.2.1.4591.4003.149176124210739275471526262702/1.3.6.1.4.1.14519.5.2.1.4591.4003.258127417527584037720287408573
TCGA-LGG	TCGA-DU	TCGA-DU-5853	flair	M	29	90.265	AXIAL_FLAIR	6	1.49065E+15	8/23/1995	GE MEDICAL SYSTEMS	MR3T	SIGNA EXCITE	SE\IR	2D	2.5	10002	125.4	2250	127.7323	3	2.5	90	0.8954	72	320	224	0.4688\0.4688	TCGA-DU-5853/1.3.6.1.4.1.14519.5.2.1.4591.4003.488910637254672352689793378436/1.3.6.1.4.1.14519.5.2.1.4591.4003.179001448691349455487193384159
TCGA-LGG	TCGA-DU	TCGA-DU-5853	t1Gd	M	29	90.265	AX_T1_POST_GD_FLAIR	11	1.49065E+15	8/23/1995	GE MEDICAL SYSTEMS	MR3T	SIGNA EXCITE	SE\IR	2D	2.5	3233.94	6.356	1238	127.7323	3	2.5	90	1.7498	72	320	224	0.4688\0.4688	TCGA-DU-5853/1.3.6.1.4.1.14519.5.2.1.4591.4003.488910637254672352689793378436/1.3.6.1.4.1.14519.5.2.1.4591.4003.864077196769485883942711381300
TCGA-LGG	TCGA-DU	TCGA-DU-5853	t1	M	29	90.265	AX_T1_pre_gd	8	1.49065E+15	8/23/1995	GE MEDICAL SYSTEMS	MR3T	SIGNA EXCITE	SE\IR	2D	5	2040.27	6.356	883	127.7323	3	5	90	1.3873	36	320	224	0.4688\0.4688	TCGA-DU-5853/1.3.6.1.4.1.14519.5.2.1.4591.4003.488910637254672352689793378436/1.3.6.1.4.1.14519.5.2.1.4591.4003.185137173531988376064097714019
TCGA-LGG	TCGA-DU	TCGA-DU-5853	t2	M	29	90.265	AX_T2_FR-FSE_RF2_150	7	1.49065E+15	8/23/1995	GE MEDICAL SYSTEMS	MR3T	SIGNA EXCITE	SE	2D	5	4000	103.464	0	127.7323	3	5	90	1.3144	36	320	224	0.4688\0.4688	TCGA-DU-5853/1.3.6.1.4.1.14519.5.2.1.4591.4003.488910637254672352689793378436/1.3.6.1.4.1.14519.5.2.1.4591.4003.118506229786576611944108655295
TCGA-LGG	TCGA-DU	TCGA-DU-5854	flair	F	57	86	Ax_FLAIR_2.5mm_for_surgery	5	2.27863E+15	11/4/1995	GE MEDICAL SYSTEMS	MR01MROW	Signa HDxt	IR	2D	2.5	10004	149.544	2200	63.8858	1.5	2.5	90	0.0328	71	256	192	0.9375\0.9375	TCGA-DU-5854/1.3.6.1.4.1.14519.5.2.1.4591.4003.765125757509176996485268928000/1.3.6.1.4.1.14519.5.2.1.4591.4003.295508881770666613087332596147
TCGA-LGG	TCGA-DU	TCGA-DU-5854	t1Gd	F	57	86	COR_T1_POST_GD	9	2.27863E+15	11/4/1995	GE MEDICAL SYSTEMS	MR01MROW	Signa HDxt	SE	2D	5	500	14	0	63.88579	1.5	5	90	0.0436644	43	256	192	0.9375\0.9375	TCGA-DU-5854/1.3.6.1.4.1.14519.5.2.1.4591.4003.765125757509176996485268928000/1.3.6.1.4.1.14519.5.2.1.4591.4003.496112103419126320360113889954
TCGA-LGG	TCGA-DU	TCGA-DU-5854	t1	F	57	86	AXIAL_T1	6	2.27863E+15	11/4/1995	GE MEDICAL SYSTEMS	MR01MROW	Signa HDxt	SE	2D	5	500	14	0	63.88579	1.5	5	90	0.0349315	36	256	192	0.9375\0.9375	TCGA-DU-5854/1.3.6.1.4.1.14519.5.2.1.4591.4003.765125757509176996485268928000/1.3.6.1.4.1.14519.5.2.1.4591.4003.106838409056090834066289704722
TCGA-LGG	TCGA-DU	TCGA-DU-5854	t2	F	57	86	AXIAL_FSE	3	2.27863E+15	11/4/1995	GE MEDICAL SYSTEMS	MR01MROW	Signa HDxt	SE	2D	5	3500	23.16	0	63.88577	1.5	5	90	0.1192	72	256	192	0.9375\0.9375	TCGA-DU-5854/1.3.6.1.4.1.14519.5.2.1.4591.4003.765125757509176996485268928000/1.3.6.1.4.1.14519.5.2.1.4591.4003.249770102183222124726802201732
TCGA-LGG	TCGA-DU	TCGA-DU-5855	flair	F		80	RA_ROUTINE_FLAIR_AX	501	2.90902E+15	12/17/1995	Philips Medical Systems	PMSN-9KQRJRSGE8	Intera	IR		5	11000	140	2800	63.89301	1.5	6	90		26	256	176	0.9765625\0.9765625	TCGA-DU-5855/1.3.6.1.4.1.14519.5.2.1.4591.4003.852581990127740690043374426777/1.3.6.1.4.1.14519.5.2.1.4591.4003.290438072272990333107581688659
TCGA-LGG	TCGA-DU	TCGA-DU-5855	t1Gd	F		80	BrainLab_onBL_3DT1axC+	1201	2.90902E+15	12/17/1995	Philips Medical Systems	PMSN-9KQRJRSGE8	Intera	GR		2	25	4.5874		63.89301	1.5	1	30		180	256	256	0.48828125\0.48828125	TCGA-DU-5855/1.3.6.1.4.1.14519.5.2.1.4591.4003.852581990127740690043374426777/1.3.6.1.4.1.14519.5.2.1.4591.4003.112592252232666023998953315710
TCGA-LGG	TCGA-DU	TCGA-DU-5855	t1	F		80	RA_ROUTINE_T1_AX	701	2.90902E+15	12/17/1995	Philips Medical Systems	PMSN-9KQRJRSGE8	Intera	SE		5	450.0002	14		63.89301	1.5	6.074518	90		26	256	203	0.9375\0.9375	TCGA-DU-5855/1.3.6.1.4.1.14519.5.2.1.4591.4003.852581990127740690043374426777/1.3.6.1.4.1.14519.5.2.1.4591.4003.304501649602313541252309917008
TCGA-LGG	TCGA-DU	TCGA-DU-5855	t2	F		80	RA_ROUTINE_T2_AX	601	2.90902E+15	12/17/1995	Philips Medical Systems	PMSN-9KQRJRSGE8	Intera	SE		5	5729.641	110		63.89301	1.5	6	90		26	352	264	0.46875\0.46875	TCGA-DU-5855/1.3.6.1.4.1.14519.5.2.1.4591.4003.852581990127740690043374426777/1.3.6.1.4.1.14519.5.2.1.4591.4003.478972965056203795981794302830
TCGA-LGG	TCGA-DU	TCGA-DU-5871	flair	F	37	86.183	AXIAL_FLAIR	6	1.41281E+15	12/6/1994	GE MEDICAL SYSTEMS	MR3T	SIGNA EXCITE	SE\IR	2D	2.5	10002	125.4	2250	127.7322	3	2.5	90	0.8466	72	320	224	0.4688\0.4688	TCGA-DU-5871/1.3.6.1.4.1.14519.5.2.1.4591.4003.247530214976989618914834177531/1.3.6.1.4.1.14519.5.2.1.4591.4003.275038399338610640375595922910
TCGA-LGG	TCGA-DU	TCGA-DU-5871	t1Gd	F	37	86.183	AX_T1_POST_GD_FLAIR	11	1.41281E+15	12/6/1994	GE MEDICAL SYSTEMS	MR3T	SIGNA EXCITE	SE\IR	2D	2.5	3212.43	6.356	1238	127.7322	3	2.5	90	1.6273	72	320	224	0.4688\0.4688	TCGA-DU-5871/1.3.6.1.4.1.14519.5.2.1.4591.4003.247530214976989618914834177531/1.3.6.1.4.1.14519.5.2.1.4591.4003.109993263227573949563103763849
TCGA-LGG	TCGA-DU	TCGA-DU-5871	t1	F	37	86.183	AX_T1_pre_gd	8	1.41281E+15	12/6/1994	GE MEDICAL SYSTEMS	MR3T	SIGNA EXCITE	SE\IR	2D	5	2040.27	6.356	883	127.7322	3	5	90	1.2815	36	320	224	0.4688\0.4688	TCGA-DU-5871/1.3.6.1.4.1.14519.5.2.1.4591.4003.247530214976989618914834177531/1.3.6.1.4.1.14519.5.2.1.4591.4003.264195765064795376195430575838
TCGA-LGG	TCGA-DU	TCGA-DU-5871	t2	F	37	86.183	AX_T2_FR-FSE_RF2_150	7	1.41281E+15	12/6/1994	GE MEDICAL SYSTEMS	MR3T	SIGNA EXCITE	SE	2D	5	4000	103.464	0	127.7322	3	5	90	1.2142	36	320	224	0.4688\0.4688	TCGA-DU-5871/1.3.6.1.4.1.14519.5.2.1.4591.4003.247530214976989618914834177531/1.3.6.1.4.1.14519.5.2.1.4591.4003.313460819031107627612831965483
TCGA-LGG	TCGA-DU	TCGA-DU-5872	flair	F	43	102.965	Ax_FLAIR_2.5mm_for_surgery	5	6.32252E+15	2/23/1995	GE MEDICAL SYSTEMS		Signa HDxt	IR	2D	2.5	10004	149.544	2200	63.8858	1.5	2.5	90	0.0316	71	256	192	0.9375\0.9375	TCGA-DU-5872/1.3.6.1.4.1.14519.5.2.1.4591.4003.120185163731564589832419288714/1.3.6.1.4.1.14519.5.2.1.4591.4003.261961539641753291673667731127
TCGA-LGG	TCGA-DU	TCGA-DU-5872	t1Gd	F	43	102.965	Ax_T1_2.5mm_for_surgery	13	6.32252E+15	2/23/1995	GE MEDICAL SYSTEMS		Signa HDxt	SE	2D	2.5	433.336	13	0	63.88584	1.5	2.5	90	0.0489826	71	256	192	0.9375\0.9375	TCGA-DU-5872/1.3.6.1.4.1.14519.5.2.1.4591.4003.120185163731564589832419288714/1.3.6.1.4.1.14519.5.2.1.4591.4003.312067442126966343281611085579
TCGA-LGG	TCGA-DU	TCGA-DU-5872	t1	F	43	102.965	AXIAL_T1	11	6.32252E+15	2/23/1995	GE MEDICAL SYSTEMS		Signa HDxt	SE	2D	2.5	433.336	13	0	63.88584	1.5	2.5	90	0.0489826	71	256	192	0.9375\0.9375	TCGA-DU-5872/1.3.6.1.4.1.14519.5.2.1.4591.4003.120185163731564589832419288714/1.3.6.1.4.1.14519.5.2.1.4591.4003.292214029866915931071160355226
TCGA-LGG	TCGA-DU	TCGA-DU-5872	t2	F	43	102.965	AXIAL_FSE	8	6.32252E+15	2/23/1995	GE MEDICAL SYSTEMS		Signa HDxt	SE	2D	5	3500	23.688	0	63.88584	1.5	5	90	0.1158	72	256	192	0.9375\0.9375	TCGA-DU-5872/1.3.6.1.4.1.14519.5.2.1.4591.4003.120185163731564589832419288714/1.3.6.1.4.1.14519.5.2.1.4591.4003.560328669711494922031263596257
TCGA-LGG	TCGA-DU	TCGA-DU-5874	flair	F	62	72.575	AXIAL_FLAIR	8	1.72824E+15	5/10/1995	GE MEDICAL SYSTEMS	MR3T	SIGNA EXCITE	SE\IR	2D	2.5	10002	125.4	2250	127.7323	3	2.5	90	0.5936	75	320	224	0.4688\0.4688	TCGA-DU-5874/1.3.6.1.4.1.14519.5.2.1.4591.4003.146738498094443311044095973527/1.3.6.1.4.1.14519.5.2.1.4591.4003.313704071781391494489476699641
TCGA-LGG	TCGA-DU	TCGA-DU-5874	t1Gd	F	62	72.575	AX_T1_POST_GD_FLAIR	12	1.72824E+15	5/10/1995	GE MEDICAL SYSTEMS	MR3T	SIGNA EXCITE	SE\IR	2D	2.5	3042	6.356	1238	127.7323	3	2.5	90	1.3335	75	320	224	0.4688\0.4688	TCGA-DU-5874/1.3.6.1.4.1.14519.5.2.1.4591.4003.146738498094443311044095973527/1.3.6.1.4.1.14519.5.2.1.4591.4003.911828461315012354254276528074
TCGA-LGG	TCGA-DU	TCGA-DU-5874	t1	F	62	72.575	AX_T1_pre_gd	9	1.72824E+15	5/10/1995	GE MEDICAL SYSTEMS	MR3T	SIGNA EXCITE	SE\IR	2D	5	2039.94	6.356	883	127.7323	3	5	90	1.5118	38	320	224	0.4688\0.4688	TCGA-DU-5874/1.3.6.1.4.1.14519.5.2.1.4591.4003.146738498094443311044095973527/1.3.6.1.4.1.14519.5.2.1.4591.4003.244192045261781363315969668886
TCGA-LGG	TCGA-DU	TCGA-DU-5874	t2	F	62	72.575	AX_T2_FR-FSE_RF2_150	6	1.72824E+15	5/10/1995	GE MEDICAL SYSTEMS	MR3T	SIGNA EXCITE	SE	2D	5	4000	103.464	0	127.7323	3	5	90	0.9547	38	320	224	0.4688\0.4688	TCGA-DU-5874/1.3.6.1.4.1.14519.5.2.1.4591.4003.146738498094443311044095973527/1.3.6.1.4.1.14519.5.2.1.4591.4003.282335375849209407307272273321
TCGA-LGG	TCGA-DU	TCGA-DU-6397	flair	M		92	AXIAL_FLAIR	3	3.39228E+15	1/30/1985	General Electric		GENESIS	flair		3	10004	155	2200			3	90		60			0.938\0.938	TCGA-DU-6397/1.3.6.1.4.1.14519.5.2.1.4591.4003.258077058743333147571656830833/1.3.6.1.4.1.14519.5.2.1.4591.4003.188438482998491912137083743459
TCGA-LGG	TCGA-DU	TCGA-DU-6397	t1Gd	M		92	AXIAL_T1_POST_GD	9	3.39228E+15	1/30/1985	General Electric		GENESIS	memp		3	500	14	0			3	90		60			0.938\0.938	TCGA-DU-6397/1.3.6.1.4.1.14519.5.2.1.4591.4003.284484161156328443723397198607/1.3.6.1.4.1.14519.5.2.1.4591.4003.270565032379072141870325726078
TCGA-LGG	TCGA-DU	TCGA-DU-6397	t1	M		92	AXIAL_T1_PRE_GD	7	3.39228E+15	1/30/1985	General Electric		GENESIS	memp		3	500	14	0			3	90		60			0.938\0.938	TCGA-DU-6397/1.3.6.1.4.1.14519.5.2.1.4591.4003.258077058743333147571656830833/1.3.6.1.4.1.14519.5.2.1.4591.4003.274264134427812998608655897757
TCGA-LGG	TCGA-DU	TCGA-DU-6397	t2	M		92	AXIAL__FSE	2	3.39228E+15	1/30/1985	General Electric		GENESIS	fse		3	3500	22	0			3	90		120			0.938\0.938	TCGA-DU-6397/1.3.6.1.4.1.14519.5.2.1.4591.4003.284484161156328443723397198607/1.3.6.1.4.1.14519.5.2.1.4591.4003.899617347532250132137213482681
TCGA-LGG	TCGA-DU	TCGA-DU-6399	flair	M		81	AXIAL_FLAIR	3	1.11121E+15	4/16/1983	General Electric	BAY	GENESIS	flair		3	10004	155	2200			3	90		53			0.938\0.938	TCGA-DU-6399/1.3.6.1.4.1.14519.5.2.1.4591.4003.299248386745518212707766902195/1.3.6.1.4.1.14519.5.2.1.4591.4003.130842300032756201956702941632
TCGA-LGG	TCGA-DU	TCGA-DU-6399	t1Gd	M		81	AXIAL_T1_PRE/POST_GD	7	1.11121E+15	4/16/1983	General Electric	BAY	GENESIS	memp		3	500	8	0			3	90		53			0.938\0.938	TCGA-DU-6399/1.3.6.1.4.1.14519.5.2.1.4591.4003.299248386745518212707766902195/1.3.6.1.4.1.14519.5.2.1.4591.4003.278779321592049493927377920886
TCGA-LGG	TCGA-DU	TCGA-DU-6399	t1	M		81	AXIAL_T1_PRE/POST_GD	9	1.11121E+15	4/16/1983	General Electric	BAY	GENESIS	memp		3	500	8	0			3	90		53			0.938\0.938	TCGA-DU-6399/1.3.6.1.4.1.14519.5.2.1.4591.4003.299248386745518212707766902195/1.3.6.1.4.1.14519.5.2.1.4591.4003.272344052386358898114067225953
TCGA-LGG	TCGA-DU	TCGA-DU-6399	t2	M		81	AXIAL_FSE	2	1.11121E+15	4/16/1983	General Electric	BAY	GENESIS	fse		3	3500	22	0			3	90		106			0.938\0.938	TCGA-DU-6399/1.3.6.1.4.1.14519.5.2.1.4591.4003.299248386745518212707766902195/1.3.6.1.4.1.14519.5.2.1.4591.4003.217995210743248085634186942839
TCGA-LGG	TCGA-DU	TCGA-DU-6400	flair	F		90	AXIAL_FLAIR	3	2.10335E+15	5/18/1983	General Electric	BAY	GENESIS	flair		3	10004	155	2200			3	90		57			0.938\0.938	TCGA-DU-6400/1.3.6.1.4.1.14519.5.2.1.4591.4003.908985028885715482708348438359/1.3.6.1.4.1.14519.5.2.1.4591.4003.566925305517233881271079424180
TCGA-LGG	TCGA-DU	TCGA-DU-6400	t1Gd	F		90	axial_t1/gd	9	2.10335E+15	5/18/1983	General Electric	BAY	GENESIS	memp		3	500	8	0			3	90		57			0.938\0.938	TCGA-DU-6400/1.3.6.1.4.1.14519.5.2.1.4591.4003.908985028885715482708348438359/1.3.6.1.4.1.14519.5.2.1.4591.4003.192875840084482262754628153598
TCGA-LGG	TCGA-DU	TCGA-DU-6400	t1	F		90	axial_t1_pre_gd	7	2.10335E+15	5/18/1983	General Electric	BAY	GENESIS	memp		3	500	8	0			3	90		57			0.938\0.938	TCGA-DU-6400/1.3.6.1.4.1.14519.5.2.1.4591.4003.908985028885715482708348438359/1.3.6.1.4.1.14519.5.2.1.4591.4003.189029122549488052602811406393
TCGA-LGG	TCGA-DU	TCGA-DU-6400	t2	F		90	AXIAL_FSE	2	2.10335E+15	5/18/1983	General Electric	BAY	GENESIS	fse		3	3500	22	0			3	90		114			0.938\0.938	TCGA-DU-6400/1.3.6.1.4.1.14519.5.2.1.4591.4003.908985028885715482708348438359/1.3.6.1.4.1.14519.5.2.1.4591.4003.706430347214749666182639541093
TCGA-LGG	TCGA-DU	TCGA-DU-6401	flair	F		105	AXIAL_FLAIR	3	1.81358E+15	10/1/1983	General Electric		GENESIS	flair		3	10004	155	2200			3	90		51			0.938\0.938	TCGA-DU-6401/1.3.6.1.4.1.14519.5.2.1.4591.4003.313654124851567890245405685812/1.3.6.1.4.1.14519.5.2.1.4591.4003.277524703900606944630060015579
TCGA-LGG	TCGA-DU	TCGA-DU-6401	t1Gd	F		105	AXIAL_T1_POST_GD	7	1.81358E+15	10/1/1983	General Electric		GENESIS	memp		3	500	14	0			3	90		58			0.938\0.938	TCGA-DU-6401/1.3.6.1.4.1.14519.5.2.1.4591.4003.313654124851567890245405685812/1.3.6.1.4.1.14519.5.2.1.4591.4003.119828237865293463375263609301
TCGA-LGG	TCGA-DU	TCGA-DU-6401	t1	F		105	AXIAL_T1_PRE_GD	5	1.81358E+15	10/1/1983	General Electric		GENESIS	memp		3	500	14	0			3	90		58			0.938\0.938	TCGA-DU-6401/1.3.6.1.4.1.14519.5.2.1.4591.4003.313654124851567890245405685812/1.3.6.1.4.1.14519.5.2.1.4591.4003.193992828285897381024209543565
TCGA-LGG	TCGA-DU	TCGA-DU-6401	t2	F		105	AXIAL__FSE	2	1.81358E+15	10/1/1983	General Electric		GENESIS	fse		3	3500	22	0			3	90		102			0.938\0.938	TCGA-DU-6401/1.3.6.1.4.1.14519.5.2.1.4591.4003.313654124851567890245405685812/1.3.6.1.4.1.14519.5.2.1.4591.4003.747042361635308948495529305328
TCGA-LGG	TCGA-DU	TCGA-DU-6404	flair	F		72	AXIAL_FLAIR	3	3.3067E+15	6/29/1985	General Electric	BAY	GENESIS	flair		3	10004	155	2200			3	90		53			0.938\0.938	TCGA-DU-6404/1.3.6.1.4.1.14519.5.2.1.4591.4003.261147184642760980049477062392/1.3.6.1.4.1.14519.5.2.1.4591.4003.123957563256151384189601599356
TCGA-LGG	TCGA-DU	TCGA-DU-6404	t1Gd	F		72	AXIAL_T1_POST_GD	10	3.3067E+15	6/29/1985	General Electric	BAY	GENESIS	memp		3	500	14	0			3	90		55			0.938\0.938	TCGA-DU-6404/1.3.6.1.4.1.14519.5.2.1.4591.4003.261147184642760980049477062392/1.3.6.1.4.1.14519.5.2.1.4591.4003.416743692444202760044121743862
TCGA-LGG	TCGA-DU	TCGA-DU-6404	t1	F		72	AXIAL_T1_PRE_GD	7	3.3067E+15	6/29/1985	General Electric	BAY	GENESIS	memp		3	500	14	0			3	90		55			0.938\0.938	TCGA-DU-6404/1.3.6.1.4.1.14519.5.2.1.4591.4003.261147184642760980049477062392/1.3.6.1.4.1.14519.5.2.1.4591.4003.318797057168031444167840441025
TCGA-LGG	TCGA-DU	TCGA-DU-6404	t2	F		72	AXIAL__FSE	2	3.3067E+15	6/29/1985	General Electric	BAY	GENESIS	fse		3	3500	22	0			3	90		110			0.938\0.938	TCGA-DU-6404/1.3.6.1.4.1.14519.5.2.1.4591.4003.261147184642760980049477062392/1.3.6.1.4.1.14519.5.2.1.4591.4003.187814519293639539595282245500
TCGA-LGG	TCGA-DU	TCGA-DU-6405	flair	F		74	AXIAL_FLAIR	3	1.72784E+15	10/5/1985	General Electric	BAY	GENESIS	flair		3	10004	155	2200			3	90		60			0.938\0.938	TCGA-DU-6405/1.3.6.1.4.1.14519.5.2.1.4591.4003.185886426431608624150226113206/1.3.6.1.4.1.14519.5.2.1.4591.4003.165112875200401239584250174943
TCGA-LGG	TCGA-DU	TCGA-DU-6405	t1Gd	F		74	AXIAL_T1_POST_GD	10	1.72784E+15	10/5/1985	General Electric	BAY	GENESIS	memp		3	500	14	0			3	90		60			0.938\0.938	TCGA-DU-6405/1.3.6.1.4.1.14519.5.2.1.4591.4003.185886426431608624150226113206/1.3.6.1.4.1.14519.5.2.1.4591.4003.652945086744147125437089852534
TCGA-LGG	TCGA-DU	TCGA-DU-6405	t1	F		74	AXIAL_T1_PRE_GD	7	1.72784E+15	10/5/1985	General Electric	BAY	GENESIS	memp		3	500	14	0			3	90		60			0.938\0.938	TCGA-DU-6405/1.3.6.1.4.1.14519.5.2.1.4591.4003.185886426431608624150226113206/1.3.6.1.4.1.14519.5.2.1.4591.4003.322175775658634614307924880128
TCGA-LGG	TCGA-DU	TCGA-DU-6405	t2	F		74	AXIAL__FSE	2	1.72784E+15	10/5/1985	General Electric	BAY	GENESIS	fse		3	3500	22	0			3	90		120			0.938\0.938	TCGA-DU-6405/1.3.6.1.4.1.14519.5.2.1.4591.4003.185886426431608624150226113206/1.3.6.1.4.1.14519.5.2.1.4591.4003.233437082494886273190709309681
TCGA-LGG	TCGA-DU	TCGA-DU-6407	flair	F	35	75.296	AXIAL_FLAIR	3	4.68473E+15	5/14/1986	GE MEDICAL SYSTEMS		GENESIS_SIGNA	RM	2D	3	10004	155		63.85846	1.5	3	90	0.033598	58	256	192	0.9375000000\0.9375000000	TCGA-DU-6407/1.3.6.1.4.1.14519.5.2.1.4591.4003.115454172245963376362112828234/1.3.6.1.4.1.14519.5.2.1.4591.4003.118594670002293851625094179535
TCGA-LGG	TCGA-DU	TCGA-DU-6407	t1Gd	F	35	75.296	AXIAL_T1_POST_GD	1	2.58298E+15	5/14/1986	GE MEDICAL SYSTEMS		GENESIS_SIGNA	SE	2D	3	500	14		63.85847	1.5	3	90	0.044591	58	256	192	0.9375000000\0.9375000000	TCGA-DU-6407/1.3.6.1.4.1.14519.5.2.1.4591.4003.189340939734965094898503523740/1.3.6.1.4.1.14519.5.2.1.4591.4003.279601843629640300146176277810
TCGA-LGG	TCGA-DU	TCGA-DU-6407	t1	F	35	75.296	AXIAL_T1_PRE-GAD/SAME_LOCS	7	4.68473E+15	5/14/1986	GE MEDICAL SYSTEMS		GENESIS_SIGNA	SE	2D	3	500	14		63.85846	1.5	3	90	0.044591	58	256	192	0.9375000000\0.9375000000	TCGA-DU-6407/1.3.6.1.4.1.14519.5.2.1.4591.4003.115454172245963376362112828234/1.3.6.1.4.1.14519.5.2.1.4591.4003.231046420302507784804996585380
TCGA-LGG	TCGA-DU	TCGA-DU-6407	t2	F	35	75.296	AXIAL__FSE	2	4.68473E+15	5/14/1986	GE MEDICAL SYSTEMS		GENESIS_SIGNA	SE	2D	3	3500	22		63.85846	1.5	3	90	0.059279	116	256	192	0.9375000000\0.9375000000	TCGA-DU-6407/1.3.6.1.4.1.14519.5.2.1.4591.4003.115454172245963376362112828234/1.3.6.1.4.1.14519.5.2.1.4591.4003.651809519364398826987125576597
TCGA-LGG	TCGA-DU	TCGA-DU-6408	flair	F		68	AXIAL_FLAIR	3	2.17053E+15	5/21/1986	General Electric	BAY	GENESIS	flair		3	10004	155	2200			3	90		56			0.938\0.938	TCGA-DU-6408/1.3.6.1.4.1.14519.5.2.1.4591.4003.112304111943954735219267840424/1.3.6.1.4.1.14519.5.2.1.4591.4003.206509980716702405576239322793
TCGA-LGG	TCGA-DU	TCGA-DU-6408	t1Gd	F		68	AXIAL_T1_POST_GD	8	2.17053E+15	5/21/1986	General Electric	BAY	GENESIS	memp		3	500	14	0			3	90		56			0.938\0.938	TCGA-DU-6408/1.3.6.1.4.1.14519.5.2.1.4591.4003.112304111943954735219267840424/1.3.6.1.4.1.14519.5.2.1.4591.4003.192752058381051614023466272787
TCGA-LGG	TCGA-DU	TCGA-DU-6408	t1	F		68	AXIAL_T1_PRE-GAD.	7	2.17053E+15	5/21/1986	General Electric	BAY	GENESIS	memp		3	500	14	0			3	90		56			0.938\0.938	TCGA-DU-6408/1.3.6.1.4.1.14519.5.2.1.4591.4003.112304111943954735219267840424/1.3.6.1.4.1.14519.5.2.1.4591.4003.232902849705284049546633334954
TCGA-LGG	TCGA-DU	TCGA-DU-6408	t2	F		68	AXIAL__FSE	2	2.17053E+15	5/21/1986	General Electric	BAY	GENESIS	fse		3	3500	22	0			3	90		112			0.938\0.938	TCGA-DU-6408/1.3.6.1.4.1.14519.5.2.1.4591.4003.112304111943954735219267840424/1.3.6.1.4.1.14519.5.2.1.4591.4003.244913018716376399862060897029
TCGA-LGG	TCGA-DU	TCGA-DU-6410	flair	M	56	81.647	Ax_FLAIR_2.5mm_for_surgery	4	3.17478E+15	12/28/1995	GE MEDICAL SYSTEMS	MR01MROW	Signa HDxt	IR	2D	2.5	10004	151.8	2200	63.88581	1.5	2.5	90	0.0351	74	256	192	0.9375\0.9375	TCGA-DU-6410/1.3.6.1.4.1.14519.5.2.1.4591.4003.265163022076336806171223776637/1.3.6.1.4.1.14519.5.2.1.4591.4003.617078350735972730657422815388
TCGA-LGG	TCGA-DU	TCGA-DU-6410	t1Gd	M	56	81.647	Ax_T1_2.5mm_for_surgery	11	3.17478E+15	12/28/1995	GE MEDICAL SYSTEMS	MR01MROW	Signa HDxt	SE	2D	2.5	500	13	0	63.88583	1.5	2.5	90	0.0440288	74	256	192	0.9375\0.9375	TCGA-DU-6410/1.3.6.1.4.1.14519.5.2.1.4591.4003.265163022076336806171223776637/1.3.6.1.4.1.14519.5.2.1.4591.4003.704707103419055538089427941121
TCGA-LGG	TCGA-DU	TCGA-DU-6410	t1	M	56	81.647	AXIAL_T1	8	3.17478E+15	12/28/1995	GE MEDICAL SYSTEMS	MR01MROW	Signa HDxt	SE	2D	5	500	14	0	63.88581	1.5	5	90	0.038159	38	256	192	0.9375\0.9375	TCGA-DU-6410/1.3.6.1.4.1.14519.5.2.1.4591.4003.265163022076336806171223776637/1.3.6.1.4.1.14519.5.2.1.4591.4003.506942221024780688931689457526
TCGA-LGG	TCGA-DU	TCGA-DU-6410	t2	M	56	81.647	AXIAL_FSE	3	3.17478E+15	12/28/1995	GE MEDICAL SYSTEMS	MR01MROW	Signa HDxt	SE	2D	5	3500	23.16	0	63.88581	1.5	5	90	0.1269	76	256	192	0.9375\0.9375	TCGA-DU-6410/1.3.6.1.4.1.14519.5.2.1.4591.4003.265163022076336806171223776637/1.3.6.1.4.1.14519.5.2.1.4591.4003.334542090086353921649284997370
TCGA-LGG	TCGA-DU	TCGA-DU-6542	flair	U	25	72.575	AXIAL_FLAIR	4	1.33377E+15	5/8/1996	GE MEDICAL SYSTEMS	MR3T	SIGNA EXCITE	SE\IR	2D	2.5	10002	125.4	2250	127.7323	3	2.5	90	0.6678	72	320	224	0.4688\0.4688	TCGA-DU-6542/1.3.6.1.4.1.14519.5.2.1.4591.4003.179855774303237007643875027308/1.3.6.1.4.1.14519.5.2.1.4591.4003.341644629709955365244089935548
TCGA-LGG	TCGA-DU	TCGA-DU-6542	t1Gd	U	25	72.575	AX_T1_POST_GD_FLAIR	11	1.33377E+15	5/8/1996	GE MEDICAL SYSTEMS	MR3T	SIGNA EXCITE	SE\IR	2D	2.5	3145.81	6.356	1238	127.7323	3	2.5	90	1.2379	72	320	224	0.4688\0.4688	TCGA-DU-6542/1.3.6.1.4.1.14519.5.2.1.4591.4003.179855774303237007643875027308/1.3.6.1.4.1.14519.5.2.1.4591.4003.666216402911624214152103699692
TCGA-LGG	TCGA-DU	TCGA-DU-6542	t1	U	25	72.575	AX_T1_pre_gd	8	1.33377E+15	5/8/1996	GE MEDICAL SYSTEMS	MR3T	SIGNA EXCITE	SE\IR	2D	5	2039.94	6.356	883	127.7323	3	5	90	1.5118	37	320	224	0.4688\0.4688	TCGA-DU-6542/1.3.6.1.4.1.14519.5.2.1.4591.4003.179855774303237007643875027308/1.3.6.1.4.1.14519.5.2.1.4591.4003.767410396026066548941611317760
TCGA-LGG	TCGA-DU	TCGA-DU-6542	t2	U	25	72.575	AX_T2_FR-FSE_RF2_150	7	1.33377E+15	5/8/1996	GE MEDICAL SYSTEMS	MR3T	SIGNA EXCITE	SE	2D	5	4000	103.464	0	127.7323	3	5	90	0.9547	37	320	224	0.4688\0.4688	TCGA-DU-6542/1.3.6.1.4.1.14519.5.2.1.4591.4003.179855774303237007643875027308/1.3.6.1.4.1.14519.5.2.1.4591.4003.827513524462400327174945110201
TCGA-LGG	TCGA-DU	TCGA-DU-7008	flair	F		67	AXIAL_FLAIR	3	2.46393E+15	7/23/1983	General Electric	BAY	GENESIS	flair		3	10004	155	2200			3	90		52			0.938\0.938	TCGA-DU-7008/1.3.6.1.4.1.14519.5.2.1.4591.4003.122287415106636818029092960413/1.3.6.1.4.1.14519.5.2.1.4591.4003.238576995972747484602053820193
TCGA-LGG	TCGA-DU	TCGA-DU-7008	t1Gd	F		67	AXIAL_T1_POST_GD	9	2.46393E+15	7/23/1983	General Electric	BAY	GENESIS	memp		3	500	14	0			3	90		52			0.938\0.938	TCGA-DU-7008/1.3.6.1.4.1.14519.5.2.1.4591.4003.122287415106636818029092960413/1.3.6.1.4.1.14519.5.2.1.4591.4003.326331027531602715966185898176
TCGA-LGG	TCGA-DU	TCGA-DU-7008	t1	F		67	AXIAL_T1_PRE_GD	7	2.46393E+15	7/23/1983	General Electric	BAY	GENESIS	memp		3	500	14	0			3	90		52			0.938\0.938	TCGA-DU-7008/1.3.6.1.4.1.14519.5.2.1.4591.4003.122287415106636818029092960413/1.3.6.1.4.1.14519.5.2.1.4591.4003.512451012844469919337165237750
TCGA-LGG	TCGA-DU	TCGA-DU-7008	t2	F		67	AXIAL_FSE	2	2.46393E+15	7/23/1983	General Electric	BAY	GENESIS	fse		3	3500	22	0			3	90		104			0.781\0.781	TCGA-DU-7008/1.3.6.1.4.1.14519.5.2.1.4591.4003.122287415106636818029092960413/1.3.6.1.4.1.14519.5.2.1.4591.4003.287636355705793503766039754021
TCGA-LGG	TCGA-DU	TCGA-DU-7010	flair	F	58	131.542	AXIAL_FLAIR	3	1.02385E+15	3/7/1986	GE MEDICAL SYSTEMS		GENESIS_SIGNA	RM	2D	3	10004	155		63.78261	1.5	3	90	0.030729	58	256	192	0.9375000000\0.9375000000	TCGA-DU-7010/1.3.6.1.4.1.14519.5.2.1.4591.4003.143081416024357624170479022330/1.3.6.1.4.1.14519.5.2.1.4591.4003.264069163339825265971003353649
TCGA-LGG	TCGA-DU	TCGA-DU-7010	t1Gd	F	58	131.542	AXIAL_T1_POST_GD	10	1.02385E+15	3/7/1986	GE MEDICAL SYSTEMS		GENESIS_SIGNA	SE	2D	3	600	14		63.7826	1.5	3	90	0.045284	58	256	192	0.9375000000\0.9375000000	TCGA-DU-7010/1.3.6.1.4.1.14519.5.2.1.4591.4003.143081416024357624170479022330/1.3.6.1.4.1.14519.5.2.1.4591.4003.586339173501030344454818020544
TCGA-LGG	TCGA-DU	TCGA-DU-7010	t1	F	58	131.542	AXIAL_T1_PRE-GAD.	6	1.02385E+15	3/7/1986	GE MEDICAL SYSTEMS		GENESIS_SIGNA	SE	2D	3	500	14		63.7826	1.5	3	90	0.040783	58	256	192	0.9375000000\0.9375000000	TCGA-DU-7010/1.3.6.1.4.1.14519.5.2.1.4591.4003.143081416024357624170479022330/1.3.6.1.4.1.14519.5.2.1.4591.4003.323984999799359680736512212053
TCGA-LGG	TCGA-DU	TCGA-DU-7010	t2	F	58	131.542	AXIAL__FSE	2	1.02385E+15	3/7/1986	GE MEDICAL SYSTEMS		GENESIS_SIGNA	SE	2D	3	3500	22		63.78261	1.5	3	90	0.054217	116	256	192	0.9375000000\0.9375000000	TCGA-DU-7010/1.3.6.1.4.1.14519.5.2.1.4591.4003.143081416024357624170479022330/1.3.6.1.4.1.14519.5.2.1.4591.4003.530247852057132627671867267641
TCGA-LGG	TCGA-DU	TCGA-DU-7014	flair	M		135	AXIAL_FLAIR	5	4.3765E+15	6/18/1986	General Electric	BAY	GENESIS	flair		3	10004	155	2200			3	90		60			0.938\0.938	TCGA-DU-7014/1.3.6.1.4.1.14519.5.2.1.4591.4003.241850919885310339695393380617/1.3.6.1.4.1.14519.5.2.1.4591.4003.153756522270512057920568006644
TCGA-LGG	TCGA-DU	TCGA-DU-7014	t1Gd	M		135	AXIAL_T1_POST_GD	11	4.3765E+15	6/18/1986	General Electric	BAY	GENESIS	memp		3	500	14	0			3	90		60			0.938\0.938	TCGA-DU-7014/1.3.6.1.4.1.14519.5.2.1.4591.4003.241850919885310339695393380617/1.3.6.1.4.1.14519.5.2.1.4591.4003.190391970345142170156174344067
TCGA-LGG	TCGA-DU	TCGA-DU-7014	t1	M		135	AXIAL_T1_PRE-GAD.	9	4.3765E+15	6/18/1986	General Electric	BAY	GENESIS	memp		3	500	14	0			3	90		60			0.938\0.938	TCGA-DU-7014/1.3.6.1.4.1.14519.5.2.1.4591.4003.241850919885310339695393380617/1.3.6.1.4.1.14519.5.2.1.4591.4003.182726989500629952477447903736
TCGA-LGG	TCGA-DU	TCGA-DU-7014	t2	M		135	AXIAL__FSE	3	4.3765E+15	6/18/1986	General Electric	BAY	GENESIS	fse		3	3500	22	0			3	90		118			0.938\0.938	TCGA-DU-7014/1.3.6.1.4.1.14519.5.2.1.4591.4003.241850919885310339695393380617/1.3.6.1.4.1.14519.5.2.1.4591.4003.196810229656728396616579184712
TCGA-LGG	TCGA-DU	TCGA-DU-7015	flair	F	41	90.718	AXIAL_FLAIR	8	2.22697E+15	6/18/1989	GE MEDICAL SYSTEMS	MR3T	SIGNA EXCITE	IR	2D	2.5	10002	124.9	2250	127.7324	3	2.5	90	0.8978	72	320	224	0.46875\0.46875	TCGA-DU-7015/1.3.6.1.4.1.14519.5.2.1.4591.4003.213617385466211233370402716160/1.3.6.1.4.1.14519.5.2.1.4591.4003.933475500875157813970798042112
TCGA-LGG	TCGA-DU	TCGA-DU-7015	t1Gd	F	41	90.718	AX_T1_POST_GD_FLAIR	10	2.22697E+15	6/18/1989	GE MEDICAL SYSTEMS	MR3T	SIGNA EXCITE	IR	2D	2.5	2965.64	6.956	1238	127.7324	3	2.5	90	1.8622	85	320	224	0.46875\0.46875	TCGA-DU-7015/1.3.6.1.4.1.14519.5.2.1.4591.4003.213617385466211233370402716160/1.3.6.1.4.1.14519.5.2.1.4591.4003.286591706137405018110350719430
TCGA-LGG	TCGA-DU	TCGA-DU-7015	t1	F	41	90.718	AX_T1_pre_gd	7	2.22697E+15	6/18/1989	GE MEDICAL SYSTEMS	MR3T	SIGNA EXCITE	IR	2D	5	2965.71	6.964	1238	127.7325	3	5	90	1.8622	51	320	224	0.46875\0.46875	TCGA-DU-7015/1.3.6.1.4.1.14519.5.2.1.4591.4003.213617385466211233370402716160/1.3.6.1.4.1.14519.5.2.1.4591.4003.108609639777979340339125393841
TCGA-LGG	TCGA-DU	TCGA-DU-7015	t2	F	41	90.718	AX_T2_FR-FSE_RF2_150	5	2.22697E+15	6/18/1989	GE MEDICAL SYSTEMS	MR3T	SIGNA EXCITE	SE	2D	5	3000	104.436	0	127.7324	3	5	90	1.3494	42	320	224	0.46875\0.46875	TCGA-DU-7015/1.3.6.1.4.1.14519.5.2.1.4591.4003.213617385466211233370402716160/1.3.6.1.4.1.14519.5.2.1.4591.4003.716639613302745992208497298258
TCGA-LGG	TCGA-DU	TCGA-DU-7018	flair	F	57	59.874	AXIAL_FLAIR	8	1.77649E+15	12/20/1991	GE MEDICAL SYSTEMS	MR3T	SIGNA EXCITE	IR	2D	2.5	10002	125.4	2250	127.7323	3	2.5	90	0.4305	72	320	224	0.4688\0.4688	TCGA-DU-7018/1.3.6.1.4.1.14519.5.2.1.4591.4003.244030423007851267821957098852/1.3.6.1.4.1.14519.5.2.1.4591.4003.330490545332144580881833428777
TCGA-LGG	TCGA-DU	TCGA-DU-7018	t1Gd	F	57	59.874	AX_T1_POST_GD_FLAIR	13	1.77649E+15	12/20/1991	GE MEDICAL SYSTEMS	MR3T	SIGNA EXCITE	IR	2D	2.5	3115.28	6.356	1238	127.7323	3	2.5	90	1.5811	72	320	224	0.4688\0.4688	TCGA-DU-7018/1.3.6.1.4.1.14519.5.2.1.4591.4003.244030423007851267821957098852/1.3.6.1.4.1.14519.5.2.1.4591.4003.185659203907953115031317947091
TCGA-LGG	TCGA-DU	TCGA-DU-7018	t1	F	57	59.874	AX_T1_pre_gd	10	1.77649E+15	12/20/1991	GE MEDICAL SYSTEMS	MR3T	SIGNA EXCITE	IR	2D	5	3115.42	6.356	1238	127.7323	3	5	90	1.581	36	320	224	0.4688\0.4688	TCGA-DU-7018/1.3.6.1.4.1.14519.5.2.1.4591.4003.244030423007851267821957098852/1.3.6.1.4.1.14519.5.2.1.4591.4003.171723091698922856226026800693
TCGA-LGG	TCGA-DU	TCGA-DU-7018	t2	F	57	59.874	AX_T2_FR-FSE_RF2_150	5	1.77649E+15	12/20/1991	GE MEDICAL SYSTEMS	MR3T	SIGNA EXCITE	SE	2D	5	3000	103.464	0	127.7323	3	5	90	0.9517	36	320	224	0.4688\0.4688	TCGA-DU-7018/1.3.6.1.4.1.14519.5.2.1.4591.4003.244030423007851267821957098852/1.3.6.1.4.1.14519.5.2.1.4591.4003.292747299771299775245149391572
TCGA-LGG	TCGA-DU	TCGA-DU-7019	flair	M	39	83.915	AXIAL_FLAIR	6	3.3163E+15	9/8/1994	GE MEDICAL SYSTEMS	MR3T	SIGNA EXCITE	SE\IR	2D	2.5	10002	125.4	2250	127.7322	3	2.5	90	0.7285	76	320	224	0.4688\0.4688	TCGA-DU-7019/1.3.6.1.4.1.14519.5.2.1.4591.4003.307537448187255340523133336813/1.3.6.1.4.1.14519.5.2.1.4591.4003.151973570528562002920929536919
TCGA-LGG	TCGA-DU	TCGA-DU-7019	t1Gd	M	39	83.915	AX_T1_POST_GD_FLAIR	11	3.3163E+15	9/8/1994	GE MEDICAL SYSTEMS	MR3T	SIGNA EXCITE	SE\IR	2D	2.5	3152.26	6.356	1238	127.7322	3	2.5	90	1.7163	76	320	224	0.4688\0.4688	TCGA-DU-7019/1.3.6.1.4.1.14519.5.2.1.4591.4003.307537448187255340523133336813/1.3.6.1.4.1.14519.5.2.1.4591.4003.261334161791196607349981234098
TCGA-LGG	TCGA-DU	TCGA-DU-7019	t1	M	39	83.915	AX_T1_pre_gd	8	3.3163E+15	9/8/1994	GE MEDICAL SYSTEMS	MR3T	SIGNA EXCITE	SE\IR	2D	5	3149.54	6.356	1238	127.7322	3	5	90	1.164	38	320	224	0.4688\0.4688	TCGA-DU-7019/1.3.6.1.4.1.14519.5.2.1.4591.4003.307537448187255340523133336813/1.3.6.1.4.1.14519.5.2.1.4591.4003.240955732907901933945193246981
TCGA-LGG	TCGA-DU	TCGA-DU-7019	t2	M	39	83.915	AX_T2_FR-FSE_RF2_150	5	3.3163E+15	9/8/1994	GE MEDICAL SYSTEMS	MR3T	SIGNA EXCITE	SE	2D	5	3000	103.464	0	127.7322	3	5	90	1.1172	38	320	224	0.4688\0.4688	TCGA-DU-7019/1.3.6.1.4.1.14519.5.2.1.4591.4003.307537448187255340523133336813/1.3.6.1.4.1.14519.5.2.1.4591.4003.288258067481390732955397456476
TCGA-LGG	TCGA-DU	TCGA-DU-7294	flair	F	53	77.111	AXIAL_FLAIR_RF2__150	7	3.10478E+15	1/4/1989	GE MEDICAL SYSTEMS	MR3T	GENESIS_SIGNA	IR	2D	2.5	10002	123.5	2250	1.28E+09	3	2.5	90	0.085563	68	256	192	0.937500\0.937500	TCGA-DU-7294/1.3.6.1.4.1.14519.5.2.1.4591.4003.559420023378630100798761419917/1.3.6.1.4.1.14519.5.2.1.4591.4003.184784670334461777798195407121
TCGA-LGG	TCGA-DU	TCGA-DU-7294	t1Gd	F	53	77.111	AX_T1_POST_GD_FLAIR	13	3.10478E+15	1/4/1989	GE MEDICAL SYSTEMS	MR3T	GENESIS_SIGNA	RM	2D	2.5	2382.272	8.104	860	1.28E+09	3	2.5	90	0.120246	68	288	192	0.468750\0.468750	TCGA-DU-7294/1.3.6.1.4.1.14519.5.2.1.4591.4003.559420023378630100798761419917/1.3.6.1.4.1.14519.5.2.1.4591.4003.359311293105706905697963367377
TCGA-LGG	TCGA-DU	TCGA-DU-7294	t1	F	53	77.111	AX_T1_pre_gd	8	3.10478E+15	1/4/1989	GE MEDICAL SYSTEMS	MR3T	GENESIS_SIGNA	RM	2D	5	2382.476	8.104	860	1.28E+09	3	5	90	0.125032	34	288	192	0.468750\0.468750	TCGA-DU-7294/1.3.6.1.4.1.14519.5.2.1.4591.4003.559420023378630100798761419917/1.3.6.1.4.1.14519.5.2.1.4591.4003.365826625949084795369137973491
TCGA-LGG	TCGA-DU	TCGA-DU-7294	t2	F	53	77.111	AX_T2_FR-FSE_RF2_150	4	3.10478E+15	1/4/1989	GE MEDICAL SYSTEMS	MR3T	GENESIS_SIGNA	RM	2D	5	3500	104.972		1.28E+09	3	5	90	0.09	34	352	192	0.468750\0.468750	TCGA-DU-7294/1.3.6.1.4.1.14519.5.2.1.4591.4003.559420023378630100798761419917/1.3.6.1.4.1.14519.5.2.1.4591.4003.152901242239851224383447108103
TCGA-LGG	TCGA-DU	TCGA-DU-7298	flair	F	38	95.254	Ax_FLAIR_2.5mm_for_surgery	4	3.94591E+15	3/24/1991	GE MEDICAL SYSTEMS	MR01MROW	SIGNA EXCITE	IR	2D	2.5	10004	149.544	2200	63.88599	1.5	2.5	90	0.0323	65	256	192	0.9375\0.9375	TCGA-DU-7298/1.3.6.1.4.1.14519.5.2.1.4591.4003.850439737313047023965195610889/1.3.6.1.4.1.14519.5.2.1.4591.4003.617708870507397603144839116562
TCGA-LGG	TCGA-DU	TCGA-DU-7298	t1Gd	F	38	95.254	Ax_T1_2.5mm_for_surgery	8	3.94591E+15	3/24/1991	GE MEDICAL SYSTEMS	MR01MROW	SIGNA EXCITE	SE	2D	2.5	500	13	0	63.88599	1.5	2.5	90	0.0513142	65	256	192	0.9375\0.9375	TCGA-DU-7298/1.3.6.1.4.1.14519.5.2.1.4591.4003.850439737313047023965195610889/1.3.6.1.4.1.14519.5.2.1.4591.4003.522835700137350932664864037553
TCGA-LGG	TCGA-DU	TCGA-DU-7298	t1	F	38	95.254	AXIAL_T1	6	3.94591E+15	3/24/1991	GE MEDICAL SYSTEMS	MR01MROW	SIGNA EXCITE	SE	2D	5	500	14	0	63.886	1.5	5	90	0.04582	32	256	192	0.9375\0.9375	TCGA-DU-7298/1.3.6.1.4.1.14519.5.2.1.4591.4003.850439737313047023965195610889/1.3.6.1.4.1.14519.5.2.1.4591.4003.227924142993589728677636698859
TCGA-LGG	TCGA-DU	TCGA-DU-7298	t2	F	38	95.254	AXIAL_FSE	3	3.94591E+15	3/24/1991	GE MEDICAL SYSTEMS	MR01MROW	SIGNA EXCITE	SE	2D	5	3500	23.688	0	63.88599	1.5	5	90	0.1075	66	256	192	0.9375\0.9375	TCGA-DU-7298/1.3.6.1.4.1.14519.5.2.1.4591.4003.850439737313047023965195610889/1.3.6.1.4.1.14519.5.2.1.4591.4003.328014632509126852764708350605
TCGA-LGG	TCGA-DU	TCGA-DU-7299	flair	M	33	95.708	AXIAL_FLAIR	5	6.19481E+15	4/17/1991	GE MEDICAL SYSTEMS	MR3T	SIGNA EXCITE	IR	2D	2.5	10002	125.4	2250	127.7323	3	2.5	90	0.8754	72	320	224	0.46875\0.46875	TCGA-DU-7299/1.3.6.1.4.1.14519.5.2.1.4591.4003.100550055416544983972223484114/1.3.6.1.4.1.14519.5.2.1.4591.4003.280133920197978134668503903289
TCGA-LGG	TCGA-DU	TCGA-DU-7299	t1Gd	M	33	95.708	AX_T1_POST_GD_FLAIR	10	6.19481E+15	4/17/1991	GE MEDICAL SYSTEMS	MR3T	SIGNA EXCITE	IR	2D	2.5	3079.21	6.356	1238	127.7323	3	2.5	90	1.6242	72	320	224	0.46875\0.46875	TCGA-DU-7299/1.3.6.1.4.1.14519.5.2.1.4591.4003.100550055416544983972223484114/1.3.6.1.4.1.14519.5.2.1.4591.4003.208743704368662518972303449564
TCGA-LGG	TCGA-DU	TCGA-DU-7299	t1	M	33	95.708	AX_T1_pre_gd	8	6.19481E+15	4/17/1991	GE MEDICAL SYSTEMS	MR3T	SIGNA EXCITE	IR	2D	5	3079.28	6.356	1238	127.7323	3	5	90	1.6242	36	320	224	0.46875\0.46875	TCGA-DU-7299/1.3.6.1.4.1.14519.5.2.1.4591.4003.100550055416544983972223484114/1.3.6.1.4.1.14519.5.2.1.4591.4003.213644167210595395557913686144
TCGA-LGG	TCGA-DU	TCGA-DU-7299	t2	M	33	95.708	AX_T2_FR-FSE_RF2_150	6	6.19481E+15	4/17/1991	GE MEDICAL SYSTEMS	MR3T	SIGNA EXCITE	SE	2D	5	3000	103.464	0	127.7323	3	5	90	1.9378	36	320	224	0.46875\0.46875	TCGA-DU-7299/1.3.6.1.4.1.14519.5.2.1.4591.4003.100550055416544983972223484114/1.3.6.1.4.1.14519.5.2.1.4591.4003.243528672587003301299349211418
TCGA-LGG	TCGA-DU	TCGA-DU-7300	flair	F	53	61.235	AXIAL_FLAIR	5	1.32855E+15	8/14/1991	GE MEDICAL SYSTEMS	MR3T	SIGNA EXCITE	IR	2D	2.5	10002	125.4	2250	127.7323	3	2.5	90	0.432	71	320	224	0.4688\0.4688	TCGA-DU-7300/1.3.6.1.4.1.14519.5.2.1.4591.4003.182337016313469829681151533968/1.3.6.1.4.1.14519.5.2.1.4591.4003.610344381534068539694672916329
TCGA-LGG	TCGA-DU	TCGA-DU-7300	t1Gd	F	53	61.235	AX_T1_POST_GD_FLAIR	10	1.32855E+15	8/14/1991	GE MEDICAL SYSTEMS	MR3T	SIGNA EXCITE	IR	2D	2.5	3126.94	6.356	1238	127.7323	3	2.5	90	1.6882	71	320	224	0.4688\0.4688	TCGA-DU-7300/1.3.6.1.4.1.14519.5.2.1.4591.4003.182337016313469829681151533968/1.3.6.1.4.1.14519.5.2.1.4591.4003.107063738082189385510017517011
TCGA-LGG	TCGA-DU	TCGA-DU-7300	t1	F	53	61.235	AX_T1_pre_gd	8	1.32855E+15	8/14/1991	GE MEDICAL SYSTEMS	MR3T	SIGNA EXCITE	IR	2D	5	3116	6.356	1238	127.7323	3	5	90	1.5862	36	320	224	0.4688\0.4688	TCGA-DU-7300/1.3.6.1.4.1.14519.5.2.1.4591.4003.182337016313469829681151533968/1.3.6.1.4.1.14519.5.2.1.4591.4003.145651249822981603023440375477
TCGA-LGG	TCGA-DU	TCGA-DU-7300	t2	F	53	61.235	AX_T2_FR-FSE_RF2_150	6	1.32855E+15	8/14/1991	GE MEDICAL SYSTEMS	MR3T	SIGNA EXCITE	SE	2D	5	3000	103.464	0	127.7323	3	5	90	0.955	36	320	224	0.4688\0.4688	TCGA-DU-7300/1.3.6.1.4.1.14519.5.2.1.4591.4003.182337016313469829681151533968/1.3.6.1.4.1.14519.5.2.1.4591.4003.240459259364124440138958818382
TCGA-LGG	TCGA-DU	TCGA-DU-7301	flair	M	53	102.058	AXIAL_FLAIR	6	2.67272E+15	11/12/1991	GE MEDICAL SYSTEMS	MR3T	SIGNA EXCITE	IR	2D	2.5	10002	125.4	2250	127.7323	3	2.5	90	0.9521	69	320	224	0.4688\0.4688	TCGA-DU-7301/1.3.6.1.4.1.14519.5.2.1.4591.4003.186750792022738934663242822050/1.3.6.1.4.1.14519.5.2.1.4591.4003.161378909706211331451432266613
TCGA-LGG	TCGA-DU	TCGA-DU-7301	t1Gd	M	53	102.058	AX_T1_POST_GD_FLAIR	10	2.67272E+15	11/12/1991	GE MEDICAL SYSTEMS	MR3T	SIGNA EXCITE	IR	2D	2.5	3099.22	6.356	1238	127.7323	3	2.5	90	1.7204	69	320	224	0.4688\0.4688	TCGA-DU-7301/1.3.6.1.4.1.14519.5.2.1.4591.4003.186750792022738934663242822050/1.3.6.1.4.1.14519.5.2.1.4591.4003.159697020502195515104591556964
TCGA-LGG	TCGA-DU	TCGA-DU-7301	t1	M	53	102.058	AX_T1_pre_gd	8	2.67272E+15	11/12/1991	GE MEDICAL SYSTEMS	MR3T	SIGNA EXCITE	IR	2D	5	3099.3	6.356	1238	127.7323	3	5	90	1.7204	35	320	224	0.4688\0.4688	TCGA-DU-7301/1.3.6.1.4.1.14519.5.2.1.4591.4003.186750792022738934663242822050/1.3.6.1.4.1.14519.5.2.1.4591.4003.168130876918360040497499340072
TCGA-LGG	TCGA-DU	TCGA-DU-7301	t2	M	53	102.058	AX_T2_FR-FSE_RF2_150	5	2.67272E+15	11/12/1991	GE MEDICAL SYSTEMS	MR3T	SIGNA EXCITE	SE	2D	5	3000	103.464	0	127.7323	3	5	90	1.4421	35	320	224	0.4688\0.4688	TCGA-DU-7301/1.3.6.1.4.1.14519.5.2.1.4591.4003.186750792022738934663242822050/1.3.6.1.4.1.14519.5.2.1.4591.4003.242378501402238333467282668026
TCGA-LGG	TCGA-DU	TCGA-DU-7302	flair	F	48	86.183	AXIAL_FLAIR	5	2.53184E+15	12/3/1991	GE MEDICAL SYSTEMS	MR3T	SIGNA EXCITE	IR	2D	2.5	10002	125.4	2250	127.7323	3	2.5	90	0.7325	72	320	224	0.4688\0.4688	TCGA-DU-7302/1.3.6.1.4.1.14519.5.2.1.4591.4003.103908736272347635807818341440/1.3.6.1.4.1.14519.5.2.1.4591.4003.339742571964695966242385646378
TCGA-LGG	TCGA-DU	TCGA-DU-7302	t1Gd	F	48	86.183	AX_T1_POST_GD_FLAIR	11	2.53184E+15	12/3/1991	GE MEDICAL SYSTEMS	MR3T	SIGNA EXCITE	IR	2D	2.5	3230.38	6.356	1238	127.7323	3	2.5	90	1.7293	72	320	224	0.4688\0.4688	TCGA-DU-7302/1.3.6.1.4.1.14519.5.2.1.4591.4003.103908736272347635807818341440/1.3.6.1.4.1.14519.5.2.1.4591.4003.102325590172330913084588545183
TCGA-LGG	TCGA-DU	TCGA-DU-7302	t1	F	48	86.183	AX_T1_pre_gd	8	2.53184E+15	12/3/1991	GE MEDICAL SYSTEMS	MR3T	SIGNA EXCITE	IR	2D	5	3028.59	6.356	1238	127.7323	3	5	90	1.3835	36	320	224	0.4688\0.4688	TCGA-DU-7302/1.3.6.1.4.1.14519.5.2.1.4591.4003.103908736272347635807818341440/1.3.6.1.4.1.14519.5.2.1.4591.4003.319430134100597162181769253768
TCGA-LGG	TCGA-DU	TCGA-DU-7302	t2	F	48	86.183	AX_T2_FR-FSE_RF2_150	6	2.53184E+15	12/3/1991	GE MEDICAL SYSTEMS	MR3T	SIGNA EXCITE	SE	2D	5	3000	103.464	0	127.7323	3	5	90	1.6192	36	320	224	0.4688\0.4688	TCGA-DU-7302/1.3.6.1.4.1.14519.5.2.1.4591.4003.103908736272347635807818341440/1.3.6.1.4.1.14519.5.2.1.4591.4003.214930116826612964540175085470
TCGA-LGG	TCGA-DU	TCGA-DU-7304	flair	M	43	77.111	AXIAL_FLAIR	5	9.06509E+15	3/25/1993	GE MEDICAL SYSTEMS	MR3T	SIGNA EXCITE	IR	2D	2.5	10002	125.4	2250	127.7322	3	2.5	90	0.7409	71	320	224	0.4688\0.4688	TCGA-DU-7304/1.3.6.1.4.1.14519.5.2.1.4591.4003.233329049923110225958892646100/1.3.6.1.4.1.14519.5.2.1.4591.4003.963112459262733039117409779801
TCGA-LGG	TCGA-DU	TCGA-DU-7304	t1Gd	M	43	77.111	AX_T1_POST_GD_FLAIR	10	9.06509E+15	3/25/1993	GE MEDICAL SYSTEMS	MR3T	SIGNA EXCITE	IR	2D	2.5	3151.38	6.356	1238	127.7322	3	2.5	90	1.2713	71	320	224	0.4688\0.4688	TCGA-DU-7304/1.3.6.1.4.1.14519.5.2.1.4591.4003.233329049923110225958892646100/1.3.6.1.4.1.14519.5.2.1.4591.4003.278231939792427678289216579006
TCGA-LGG	TCGA-DU	TCGA-DU-7304	t1	M	43	77.111	AX_T1_pre_gd	8	9.06509E+15	3/25/1993	GE MEDICAL SYSTEMS	MR3T	SIGNA EXCITE	IR	2D	5	3000.01	6.356	1238	127.7322	3	5	90	1.0016	36	320	224	0.4688\0.4688	TCGA-DU-7304/1.3.6.1.4.1.14519.5.2.1.4591.4003.233329049923110225958892646100/1.3.6.1.4.1.14519.5.2.1.4591.4003.338852786223329525878990954697
TCGA-LGG	TCGA-DU	TCGA-DU-7304	t2	M	43	77.111	AX_T2_FR-FSE_RF2_150	6	9.06509E+15	3/25/1993	GE MEDICAL SYSTEMS	MR3T	SIGNA EXCITE	SE	2D	5	3000	103.464	0	127.7322	3	5	90	1.3382	36	320	224	0.4688\0.4688	TCGA-DU-7304/1.3.6.1.4.1.14519.5.2.1.4591.4003.233329049923110225958892646100/1.3.6.1.4.1.14519.5.2.1.4591.4003.157034137886992535107544502554
TCGA-LGG	TCGA-DU	TCGA-DU-7306	flair	U	67	88.451	AXIAL_FLAIR	6	1.75684E+15	5/12/1993	GE MEDICAL SYSTEMS	MR3T	SIGNA EXCITE	IR	2D	2.5	10002	125.4	2250	127.7322	3	2.5	90	0.8735	84	320	224	0.4688\0.4688	TCGA-DU-7306/1.3.6.1.4.1.14519.5.2.1.4591.4003.127855909272597883231465711323/1.3.6.1.4.1.14519.5.2.1.4591.4003.130974444495303333550284072811
TCGA-LGG	TCGA-DU	TCGA-DU-7306	t1Gd	U	67	88.451	AX_T1_POST_GD_FLAIR	11	1.75684E+15	5/12/1993	GE MEDICAL SYSTEMS	MR3T	SIGNA EXCITE	IR	2D	2.5	3115.46	6.356	1238	127.7322	3	2.5	90	1.4234	84	320	224	0.4688\0.4688	TCGA-DU-7306/1.3.6.1.4.1.14519.5.2.1.4591.4003.127855909272597883231465711323/1.3.6.1.4.1.14519.5.2.1.4591.4003.259382038351091849536381817891
TCGA-LGG	TCGA-DU	TCGA-DU-7306	t1	U	67	88.451	AX_T1_pre_gd	9	1.75684E+15	5/12/1993	GE MEDICAL SYSTEMS	MR3T	SIGNA EXCITE	IR	2D	5	3115.54	6.356	1238	127.7322	3	5	90	1.4233	42	320	224	0.4688\0.4688	TCGA-DU-7306/1.3.6.1.4.1.14519.5.2.1.4591.4003.127855909272597883231465711323/1.3.6.1.4.1.14519.5.2.1.4591.4003.155963458783583162763789728619
TCGA-LGG	TCGA-DU	TCGA-DU-7306	t2	U	67	88.451	AX_T2_FR-FSE_RF2_150	7	1.75684E+15	5/12/1993	GE MEDICAL SYSTEMS	MR3T	SIGNA EXCITE	SE	2D	5	3000	103.464	0	127.7322	3	5	90	1.3167	42	320	224	0.4688\0.4688	TCGA-DU-7306/1.3.6.1.4.1.14519.5.2.1.4591.4003.127855909272597883231465711323/1.3.6.1.4.1.14519.5.2.1.4591.4003.103388284169265511747126541748
TCGA-LGG	TCGA-DU	TCGA-DU-7309	flair	F	41	76.657	AXIAL_FLAIR	6	2.57831E+15	8/31/1996	GE MEDICAL SYSTEMS	MR3T	SIGNA EXCITE	SE\IR	2D	2.5	10002	125.4	2250	127.7323	3	2.5	90	0.652	80	320	224	0.4688\0.4688	TCGA-DU-7309/1.3.6.1.4.1.14519.5.2.1.4591.4003.168648868285787837108458883234/1.3.6.1.4.1.14519.5.2.1.4591.4003.492234721143934975701574344187
TCGA-LGG	TCGA-DU	TCGA-DU-7309	t1Gd	F	41	76.657	AX_T1_POST_GD_FLAIR	12	2.57831E+15	8/31/1996	GE MEDICAL SYSTEMS	MR3T	SIGNA EXCITE	SE\IR	2D	2.5	2970.27	6.356	1238	127.7323	3	2.5	90	1.62	80	320	224	0.4688\0.4688	TCGA-DU-7309/1.3.6.1.4.1.14519.5.2.1.4591.4003.168648868285787837108458883234/1.3.6.1.4.1.14519.5.2.1.4591.4003.179698976018344713629324575778
TCGA-LGG	TCGA-DU	TCGA-DU-7309	t1	F	41	76.657	AX_T1_pre_gd	10	2.57831E+15	8/31/1996	GE MEDICAL SYSTEMS	MR3T	SIGNA EXCITE	SE\IR	2D	5	2141.76	6.356	883	127.7323	3	5	90	1.6646	40	320	224	0.4688\0.4688	TCGA-DU-7309/1.3.6.1.4.1.14519.5.2.1.4591.4003.168648868285787837108458883234/1.3.6.1.4.1.14519.5.2.1.4591.4003.992012705357304347023368682760
TCGA-LGG	TCGA-DU	TCGA-DU-7309	t2	F	41	76.657	AX_T2_FR-FSE_RF2_150	4	2.57831E+15	8/31/1996	GE MEDICAL SYSTEMS	MR3T	SIGNA EXCITE	SE	2D	5	4000	103.464	0	127.7322	3	5	90	1.1038	40	320	224	0.4688\0.4688	TCGA-DU-7309/1.3.6.1.4.1.14519.5.2.1.4591.4003.168648868285787837108458883234/1.3.6.1.4.1.14519.5.2.1.4591.4003.156894594838434855648349079703
TCGA-LGG	TCGA-DU	TCGA-DU-8162	flair	F	61	56.699	AXIAL_FLAIR	4	2.43944E+15	10/29/1996	GE MEDICAL SYSTEMS	MR3T	SIGNA EXCITE	SE\IR	2D	2.5	10002	125.4	2250	127.7323	3	2.5	90	0.5225	72	320	224	0.4688\0.4688	TCGA-DU-8162/1.3.6.1.4.1.14519.5.2.1.4591.4003.239858237185580206387331844912/1.3.6.1.4.1.14519.5.2.1.4591.4003.232666278628494820601580634659
TCGA-LGG	TCGA-DU	TCGA-DU-8162	t1Gd	F	61	56.699	AX_T1_POST_GD_FLAIR	11	2.43944E+15	10/29/1996	GE MEDICAL SYSTEMS	MR3T	SIGNA EXCITE	SE\IR	2D	2.5	3103.47	6.356	1238	127.7322	3	2.5	90	1.4727	72	320	224	0.4688\0.4688	TCGA-DU-8162/1.3.6.1.4.1.14519.5.2.1.4591.4003.239858237185580206387331844912/1.3.6.1.4.1.14519.5.2.1.4591.4003.120802060567647850144606478295
TCGA-LGG	TCGA-DU	TCGA-DU-8162	t1	F	61	56.699	AX_T1_pre_gd	8	2.43944E+15	10/29/1996	GE MEDICAL SYSTEMS	MR3T	SIGNA EXCITE	SE\IR	2D	5	2039.94	6.356	883	127.7323	3	5	90	1.1829	37	320	224	0.4688\0.4688	TCGA-DU-8162/1.3.6.1.4.1.14519.5.2.1.4591.4003.239858237185580206387331844912/1.3.6.1.4.1.14519.5.2.1.4591.4003.199135420531148912087697063463
TCGA-LGG	TCGA-DU	TCGA-DU-8162	t2	F	61	56.699	AX_T2_FR-FSE_RF2_150	7	2.43944E+15	10/29/1996	GE MEDICAL SYSTEMS	MR3T	SIGNA EXCITE	SE	2D	5	4000	103.464	0	127.7323	3	5	90	0.747	37	320	224	0.4688\0.4688	TCGA-DU-8162/1.3.6.1.4.1.14519.5.2.1.4591.4003.239858237185580206387331844912/1.3.6.1.4.1.14519.5.2.1.4591.4003.233930895945610254694242682140
TCGA-LGG	TCGA-DU	TCGA-DU-8164	flair	M	51	99.79	AXIAL_FLAIR	4	2.13629E+15	1/11/1997	GE MEDICAL SYSTEMS	MR3T	SIGNA EXCITE	SE\IR	2D	2.5	10002	125.4	2250	127.7322	3	2.5	90	1.0139	72	320	224	0.4688\0.4688	TCGA-DU-8164/1.3.6.1.4.1.14519.5.2.1.4591.4003.286153250156424635720891098366/1.3.6.1.4.1.14519.5.2.1.4591.4003.263357156153587255427662103012
TCGA-LGG	TCGA-DU	TCGA-DU-8164	t1Gd	M	51	99.79	AX_T1_POST_GD_FLAIR	10	2.13629E+15	1/11/1997	GE MEDICAL SYSTEMS	MR3T	SIGNA EXCITE	SE\IR	2D	2.5	3081.66	6.356	1238	127.7322	3	2.5	90	1.6356	72	320	224	0.4688\0.4688	TCGA-DU-8164/1.3.6.1.4.1.14519.5.2.1.4591.4003.286153250156424635720891098366/1.3.6.1.4.1.14519.5.2.1.4591.4003.505191286789266005167052602819
TCGA-LGG	TCGA-DU	TCGA-DU-8164	t1	M	51	99.79	AX_T1_pre_gd	8	2.13629E+15	1/11/1997	GE MEDICAL SYSTEMS	MR3T	SIGNA EXCITE	SE\IR	2D	5	2198.48	6.356	883	127.7322	3	5	90	1.6562	37	320	224	0.4688\0.4688	TCGA-DU-8164/1.3.6.1.4.1.14519.5.2.1.4591.4003.286153250156424635720891098366/1.3.6.1.4.1.14519.5.2.1.4591.4003.304093308417890058498224439637
TCGA-LGG	TCGA-DU	TCGA-DU-8164	t2	M	51	99.79	AX_T2_FR-FSE_RF2_150	7	2.13629E+15	1/11/1997	GE MEDICAL SYSTEMS	MR3T	SIGNA EXCITE	SE	2D	5	4000	103.464	0	127.7322	3	5	90	1.6476	37	320	224	0.4688\0.4688	TCGA-DU-8164/1.3.6.1.4.1.14519.5.2.1.4591.4003.286153250156424635720891098366/1.3.6.1.4.1.14519.5.2.1.4591.4003.227082676099967810370197194767
TCGA-LGG	TCGA-DU	TCGA-DU-8165	flair	F	60	58.967	AXIAL_FLAIR	6	8.26478E+15	2/5/1997	GE MEDICAL SYSTEMS	MR3T	SIGNA EXCITE	SE\IR	2D	2.5	10002	125.4	2250	127.7322	3	2.5	90	0.5424	69	320	224	0.4688\0.4688	TCGA-DU-8165/1.3.6.1.4.1.14519.5.2.1.4591.4003.140730945904429213927161894305/1.3.6.1.4.1.14519.5.2.1.4591.4003.958512138684118440272310116811
TCGA-LGG	TCGA-DU	TCGA-DU-8165	t1Gd	F	60	58.967	AX_T1_POST_GD_FLAIR	12	8.26478E+15	2/5/1997	GE MEDICAL SYSTEMS	MR3T	SIGNA EXCITE	SE\IR	2D	2.5	3104.34	6.356	1238	127.7322	3	2.5	90	1.4813	72	320	224	0.4688\0.4688	TCGA-DU-8165/1.3.6.1.4.1.14519.5.2.1.4591.4003.140730945904429213927161894305/1.3.6.1.4.1.14519.5.2.1.4591.4003.317099727468822952520944196133
TCGA-LGG	TCGA-DU	TCGA-DU-8165	t1	F	60	58.967	AX_T1_pre_gd	9	8.26478E+15	2/5/1997	GE MEDICAL SYSTEMS	MR3T	SIGNA EXCITE	SE\IR	2D	5	2101.38	6.356	883	127.7322	3	5	90	1.0148	36	320	224	0.4688\0.4688	TCGA-DU-8165/1.3.6.1.4.1.14519.5.2.1.4591.4003.140730945904429213927161894305/1.3.6.1.4.1.14519.5.2.1.4591.4003.305131916722305493965002769017
TCGA-LGG	TCGA-DU	TCGA-DU-8165	t2	F	60	58.967	AX_T2_FR-FSE_RF2_150	8	8.26478E+15	2/5/1997	GE MEDICAL SYSTEMS	MR3T	SIGNA EXCITE	SE	2D	5	4000	103.464	0	127.7322	3	5	90	0.712	36	320	224	0.4688\0.4688	TCGA-DU-8165/1.3.6.1.4.1.14519.5.2.1.4591.4003.140730945904429213927161894305/1.3.6.1.4.1.14519.5.2.1.4591.4003.486167804750109627510369135709
TCGA-LGG	TCGA-DU	TCGA-DU-8166	flair	U	29	61.235	AXIAL_FLAIR	7	2.32218E+15	3/22/1997	GE MEDICAL SYSTEMS	MR3T	SIGNA EXCITE	SE\IR	2D	2.5	10002	125.4	2250	127.7323	3	2.5	90	0.5456	72	320	224	0.4688\0.4688	TCGA-DU-8166/1.3.6.1.4.1.14519.5.2.1.4591.4003.336568303900495873094738894974/1.3.6.1.4.1.14519.5.2.1.4591.4003.172004762305844447401871420431
TCGA-LGG	TCGA-DU	TCGA-DU-8166	t1Gd	U	29	61.235	AX_T1_POST_GD_FLAIR	12	2.32218E+15	3/22/1997	GE MEDICAL SYSTEMS	MR3T	SIGNA EXCITE	SE\IR	2D	2.5	2556.99	6.356	1076	127.7323	3	2.5	90	1.8091	72	320	224	0.4688\0.4688	TCGA-DU-8166/1.3.6.1.4.1.14519.5.2.1.4591.4003.336568303900495873094738894974/1.3.6.1.4.1.14519.5.2.1.4591.4003.141004776239267801712876247718
TCGA-LGG	TCGA-DU	TCGA-DU-8166	t1	U	29	61.235	AX_T1_pre_gd	9	2.32218E+15	3/22/1997	GE MEDICAL SYSTEMS	MR3T	SIGNA EXCITE	SE\IR	2D	5	2540.29	6.356	1067	127.7323	3	5	90	1.821	36	320	224	0.4688\0.4688	TCGA-DU-8166/1.3.6.1.4.1.14519.5.2.1.4591.4003.336568303900495873094738894974/1.3.6.1.4.1.14519.5.2.1.4591.4003.270588047395699670537024032082
TCGA-LGG	TCGA-DU	TCGA-DU-8166	t2	U	29	61.235	AX_T2_FR-FSE_RF2_150	8	2.32218E+15	3/22/1997	GE MEDICAL SYSTEMS	MR3T	SIGNA EXCITE	SE	2D	5	4000	103.464	0	127.7323	3	5	90	0.7582	36	320	224	0.4688\0.4688	TCGA-DU-8166/1.3.6.1.4.1.14519.5.2.1.4591.4003.336568303900495873094738894974/1.3.6.1.4.1.14519.5.2.1.4591.4003.180440215937530195248658513948
TCGA-LGG	TCGA-DU	TCGA-DU-8167	flair	F	69	74.843	AXIAL_FLAIR	6	3.35479E+15	4/2/1997	GE MEDICAL SYSTEMS	MR3T	SIGNA EXCITE	SE\IR	2D	2.5	10002	125.4	2250	127.7322	3	2.5	90	0.6258	73	320	224	0.4688\0.4688	TCGA-DU-8167/1.3.6.1.4.1.14519.5.2.1.4591.4003.168817713276868679404712029125/1.3.6.1.4.1.14519.5.2.1.4591.4003.973607962673153304494674925902
TCGA-LGG	TCGA-DU	TCGA-DU-8167	t1Gd	F	69	74.843	AX_T1_POST_GD_FLAIR	10	3.35479E+15	4/2/1997	GE MEDICAL SYSTEMS	MR3T	SIGNA EXCITE	SE\IR	2D	2.5	3042	6.356	1238	127.7322	3	2.5	90	1.4057	73	320	224	0.4688\0.4688	TCGA-DU-8167/1.3.6.1.4.1.14519.5.2.1.4591.4003.168817713276868679404712029125/1.3.6.1.4.1.14519.5.2.1.4591.4003.158150700999497317857027374207
TCGA-LGG	TCGA-DU	TCGA-DU-8167	t1	F	69	74.843	AX_T1_pre_gd	8	3.35479E+15	4/2/1997	GE MEDICAL SYSTEMS	MR3T	SIGNA EXCITE	SE\IR	2D	5	2039.94	6.356	883	127.7322	3	5	90	1.5937	37	320	224	0.4688\0.4688	TCGA-DU-8167/1.3.6.1.4.1.14519.5.2.1.4591.4003.168817713276868679404712029125/1.3.6.1.4.1.14519.5.2.1.4591.4003.200746342658024033787157836450
TCGA-LGG	TCGA-DU	TCGA-DU-8167	t2	F	69	74.843	AX_T2_FR-FSE_RF2_150	7	3.35479E+15	4/2/1997	GE MEDICAL SYSTEMS	MR3T	SIGNA EXCITE	SE	2D	5	4000	103.464	0	127.7322	3	5	90	0.9534	36	320	224	0.4688\0.4688	TCGA-DU-8167/1.3.6.1.4.1.14519.5.2.1.4591.4003.168817713276868679404712029125/1.3.6.1.4.1.14519.5.2.1.4591.4003.130537543563011500866320702988
TCGA-LGG	TCGA-DU	TCGA-DU-8168	flair	F	55	47.627	AXIAL_FLAIR	7	4.84646E+15	5/3/1997	GE MEDICAL SYSTEMS	MR3T	SIGNA EXCITE	SE\IR	2D	2.5	10002	125.4	2250	127.7323	3	2.5	90	0.5249	71	320	224	0.4688\0.4688	TCGA-DU-8168/1.3.6.1.4.1.14519.5.2.1.4591.4003.246416124390990436653999855138/1.3.6.1.4.1.14519.5.2.1.4591.4003.211295928669757772419648940636
TCGA-LGG	TCGA-DU	TCGA-DU-8168	t1Gd	F	55	47.627	AX_T1_POST_GD_FLAIR	12	4.84646E+15	5/3/1997	GE MEDICAL SYSTEMS	MR3T	SIGNA EXCITE	SE\IR	2D	2.5	3099.44	6.356	1238	127.7323	3	2.5	90	1.4356	71	320	224	0.4688\0.4688	TCGA-DU-8168/1.3.6.1.4.1.14519.5.2.1.4591.4003.246416124390990436653999855138/1.3.6.1.4.1.14519.5.2.1.4591.4003.207704217831644522002629479320
TCGA-LGG	TCGA-DU	TCGA-DU-8168	t1	F	55	47.627	AX_T1_pre_gd	8	4.84646E+15	5/3/1997	GE MEDICAL SYSTEMS	MR3T	SIGNA EXCITE	SE\IR	2D	5	2107.14	6.356	883	127.7323	3	5	90	1.0561	36	320	224	0.4688\0.4688	TCGA-DU-8168/1.3.6.1.4.1.14519.5.2.1.4591.4003.246416124390990436653999855138/1.3.6.1.4.1.14519.5.2.1.4591.4003.227113158557470572673750614297
TCGA-LGG	TCGA-DU	TCGA-DU-8168	t2	F	55	47.627	AX_T2_FR-FSE_RF2_150	4	4.84646E+15	5/3/1997	GE MEDICAL SYSTEMS	MR3T	SIGNA EXCITE	SE	2D	5	4000	103.464	0	127.7323	3	5	90	0.6889	36	320	224	0.4688\0.4688	TCGA-DU-8168/1.3.6.1.4.1.14519.5.2.1.4591.4003.246416124390990436653999855138/1.3.6.1.4.1.14519.5.2.1.4591.4003.197335199152369341066172598020
TCGA-LGG	TCGA-DU	TCGA-DU-A5TP	flair	M	33	106.594	AXIAL_FLAIR	6	8.32022E+15	6/14/1997	GE MEDICAL SYSTEMS		SIGNA EXCITE	SE\IR	2D	2.5	10002	125.4	2250	127.7322	3	2.5	90	1.1015	72	320	224	0.4688\0.4688	TCGA-DU-A5TP/1.3.6.1.4.1.14519.5.2.1.4591.4003.291142487825047263671923414542/1.3.6.1.4.1.14519.5.2.1.4591.4003.974831159092121399697175807910
TCGA-LGG	TCGA-DU	TCGA-DU-A5TP	t1Gd	M	33	106.594	AX_T1_POST_GD_FLAIR	10	8.32022E+15	6/14/1997	GE MEDICAL SYSTEMS		SIGNA EXCITE	SE\IR	2D	2.5	3042.02	6.356	1238	127.7322	3	2.5	90	1.5357	72	320	224	0.4688\0.4688	TCGA-DU-A5TP/1.3.6.1.4.1.14519.5.2.1.4591.4003.291142487825047263671923414542/1.3.6.1.4.1.14519.5.2.1.4591.4003.240056898333026509898056790264
TCGA-LGG	TCGA-DU	TCGA-DU-A5TP	t1	M	33	106.594	AX_T1_pre_gd	7	8.32022E+15	6/14/1997	GE MEDICAL SYSTEMS		SIGNA EXCITE	SE\IR	2D	5	2060.84	6.356	883	127.7322	3	5	90	1.5117	38	320	224	0.4688\0.4688	TCGA-DU-A5TP/1.3.6.1.4.1.14519.5.2.1.4591.4003.291142487825047263671923414542/1.3.6.1.4.1.14519.5.2.1.4591.4003.414793790467374440698643449092
TCGA-LGG	TCGA-DU	TCGA-DU-A5TP	t2	M	33	106.594	AX_T2_FR-FSE_RF2_150	5	8.32022E+15	6/14/1997	GE MEDICAL SYSTEMS		SIGNA EXCITE	SE	2D	5	4000	103.464	0	127.7322	3	5	90	1.8447	38	320	224	0.4688\0.4688	TCGA-DU-A5TP/1.3.6.1.4.1.14519.5.2.1.4591.4003.291142487825047263671923414542/1.3.6.1.4.1.14519.5.2.1.4591.4003.125375269087118703971208415488
TCGA-LGG	TCGA-DU	TCGA-DU-A5TR	flair	M	51	90.718	AXIAL_FLAIR	5	1.80456E+15	7/26/1997	GE MEDICAL SYSTEMS		SIGNA EXCITE	SE\IR	2D	2.5	10002	125.4	2250	127.7322	3	2.5	90	0.9008	72	320	224	0.4688\0.4688	TCGA-DU-A5TR/1.3.6.1.4.1.14519.5.2.1.4591.4003.425990241084610539458158617402/1.3.6.1.4.1.14519.5.2.1.4591.4003.279028600034741547478221979506
TCGA-LGG	TCGA-DU	TCGA-DU-A5TR	t1Gd	M	51	90.718	AX_T1_POST_GD_FLAIR	10	1.80456E+15	7/26/1997	GE MEDICAL SYSTEMS		SIGNA EXCITE	SE\IR	2D	2.5	3236.34	6.356	1238	127.7322	3	2.5	90	1.7636	72	320	224	0.4688\0.4688	TCGA-DU-A5TR/1.3.6.1.4.1.14519.5.2.1.4591.4003.425990241084610539458158617402/1.3.6.1.4.1.14519.5.2.1.4591.4003.149558942269897008551830323755
TCGA-LGG	TCGA-DU	TCGA-DU-A5TR	t1	M	51	90.718	AX_T1_pre_gd	7	1.80456E+15	7/26/1997	GE MEDICAL SYSTEMS		SIGNA EXCITE	SE\IR	2D	5	2152.82	6.356	883	127.7323	3	5	90	1.4365	38	320	224	0.4688\0.4688	TCGA-DU-A5TR/1.3.6.1.4.1.14519.5.2.1.4591.4003.425990241084610539458158617402/1.3.6.1.4.1.14519.5.2.1.4591.4003.421746639107968090165101173601
TCGA-LGG	TCGA-DU	TCGA-DU-A5TR	t2	M	51	90.718	AX_T2_FR-FSE_RF2_150	4	1.80456E+15	7/26/1997	GE MEDICAL SYSTEMS		SIGNA EXCITE	SE	2D	5	4000	103.464	0	127.7322	3	5	90	1.3993	38	320	224	0.4688\0.4688	TCGA-DU-A5TR/1.3.6.1.4.1.14519.5.2.1.4591.4003.425990241084610539458158617402/1.3.6.1.4.1.14519.5.2.1.4591.4003.796723886140993479035572234223
TCGA-LGG	TCGA-DU	TCGA-DU-A5TS	flair	M	42	90.718	Ax_FLAIR_2.5mm_for_surgery	4	2.8282E+15	7/26/1997	GE MEDICAL SYSTEMS		Signa HDxt	IR	2D	2.5	10004	149.544	2200	63.88574	1.5	2.5	90	0.9489	69	256	192	0.9375\0.9375	TCGA-DU-A5TS/1.3.6.1.4.1.14519.5.2.1.4591.4003.506469428889289487430866900379/1.3.6.1.4.1.14519.5.2.1.4591.4003.133906818467918965336047375341
TCGA-LGG	TCGA-DU	TCGA-DU-A5TS	t1Gd	M	42	90.718	Ax_T1_2.5mm_for_surgery	11	2.8282E+15	7/26/1997	GE MEDICAL SYSTEMS		Signa HDxt	SE	2D	2.5	516.668	13	0	63.88574	1.5	2.5	90	1.46247	69	256	192	0.9375\0.9375	TCGA-DU-A5TS/1.3.6.1.4.1.14519.5.2.1.4591.4003.506469428889289487430866900379/1.3.6.1.4.1.14519.5.2.1.4591.4003.108198143538126559661143849338
TCGA-LGG	TCGA-DU	TCGA-DU-A5TS	t1	M	42	90.718	AXIAL_T1	5	2.8282E+15	7/26/1997	GE MEDICAL SYSTEMS		Signa HDxt	SE	2D	5	550	14	0	63.88574	1.5	5	90	1.37351	35	256	192	0.9375\0.9375	TCGA-DU-A5TS/1.3.6.1.4.1.14519.5.2.1.4591.4003.506469428889289487430866900379/1.3.6.1.4.1.14519.5.2.1.4591.4003.318078055811576334314843090781
TCGA-LGG	TCGA-DU	TCGA-DU-A5TS	t2	M	42	90.718	Ax_T2_FSE	7	2.8282E+15	7/26/1997	GE MEDICAL SYSTEMS		Signa HDxt	SE	2D	5	3500	59.456	0	63.88575	1.5	5	90	1.2952	35	256	192	0.9375\0.9375	TCGA-DU-A5TS/1.3.6.1.4.1.14519.5.2.1.4591.4003.506469428889289487430866900379/1.3.6.1.4.1.14519.5.2.1.4591.4003.221097235994186959722655915953
TCGA-LGG	TCGA-DU	TCGA-DU-A5TT	flair	M		85	FLAIR_LongTR	601	1.13728E+15	3/18/1998	Philips Healthcare		Ingenia	IR	2D	2.5	11000	125	2800	127.7473	3	2.5	90	0.234652877	79	320	232	0.625\0.625	TCGA-DU-A5TT/1.3.6.1.4.1.14519.5.2.1.4591.4003.166583820652373876958420163650/1.3.6.1.4.1.14519.5.2.1.4591.4003.644099169794791264778319370500
TCGA-LGG	TCGA-DU	TCGA-DU-A5TT	t1Gd	M		85	=+ax_t1_1mm	1205	1.13728E+15	3/18/1998	Philips Healthcare		Ingenia	GR	3D	1	8.0445	3.685		127.7472	3	1	8	0.024283046	215	240	222	0.9375\0.9375	TCGA-DU-A5TT/1.3.6.1.4.1.14519.5.2.1.4591.4003.166583820652373876958420163650/1.3.6.1.4.1.14519.5.2.1.4591.4003.293175010350662286369185988684
TCGA-LGG	TCGA-DU	TCGA-DU-A5TT	t1	M		85	Pre_AX_T1W_IR_TSE	801	1.13728E+15	3/18/1998	Philips Healthcare		Ingenia	IR	2D	2.5	2000	20	900	127.7473	3	2.5	90	0.178819239	65	308	233	0.46875\0.46875	TCGA-DU-A5TT/1.3.6.1.4.1.14519.5.2.1.4591.4003.166583820652373876958420163650/1.3.6.1.4.1.14519.5.2.1.4591.4003.591742088771199762537683503915
TCGA-LGG	TCGA-DU	TCGA-DU-A5TT	t2	M		85	COR_T2_1MM	1303	1.13728E+15	3/18/1998	Philips Healthcare		Ingenia	SE	3D	0.977	2500	252.046		127.7472	3	0.977	90	0.032736853	235	252	252	0.9765625\0.9765625	TCGA-DU-A5TT/1.3.6.1.4.1.14519.5.2.1.4591.4003.166583820652373876958420163650/1.3.6.1.4.1.14519.5.2.1.4591.4003.319562880877655611034521122625
TCGA-LGG	TCGA-DU	TCGA-DU-A5TU	flair	F	62	158.76	Axial_FLAIR	10	1.46469E+15	3/12/1998	Hitachi Medical Corporation	OASIS	IR	2D	5	6600	100	2000	49.40144	1.16	6	90	0.96	24	256	230	0.390625\0.390625	TCGA-DU-A5TU/1.3.6.1.4.1.14519.5.2.1.4591.4003.141072676039453911508495577342/1.3.6.1.4.1.14519.5.2.1.4591.4003.312699074942345945201654113367	
TCGA-LGG	TCGA-DU	TCGA-DU-A5TU	t1Gd	F	62	158.76	Axial_T1_FSE_Post_Gad	15	1.46469E+15	3/12/1998	Hitachi Medical Corporation	OASIS	SE	2D	5	479	12	0	49.40143	1.16	7	90	1.48	23	256	230	0.390625\0.390625	TCGA-DU-A5TU/1.3.6.1.4.1.14519.5.2.1.4591.4003.141072676039453911508495577342/1.3.6.1.4.1.14519.5.2.1.4591.4003.171470113945364268606527917816	
TCGA-LGG	TCGA-DU	TCGA-DU-A5TU	t1	F	62	158.76	Axial_T1_FSE	9	1.46469E+15	3/12/1998	Hitachi Medical Corporation	OASIS	SE	2D	5	479	12	0	49.40144	1.16	7	90	1.48	23	256	230	0.390625\0.390625	TCGA-DU-A5TU/1.3.6.1.4.1.14519.5.2.1.4591.4003.141072676039453911508495577342/1.3.6.1.4.1.14519.5.2.1.4591.4003.203628204006459721121981606321	
TCGA-LGG	TCGA-DU	TCGA-DU-A5TU	t2	F	62	158.76	Axial_T2_FSE_Hi-Res	7	1.46469E+15	3/12/1998	Hitachi Medical Corporation	OASIS	SE	2D	5	4829	96	0	49.40144	1.16	7	90	1.52	23	512	288	0.390625\0.390625	TCGA-DU-A5TU/1.3.6.1.4.1.14519.5.2.1.4591.4003.141072676039453911508495577342/1.3.6.1.4.1.14519.5.2.1.4591.4003.254982119512511066350514016668	
TCGA-LGG	TCGA-DU	TCGA-DU-A5TW	flair	F		73	AX_T2_FLAIR	501	2.42768E+15	2/28/1998	Philips Healthcare		Ingenia	IR	2D	5	11000	140	2800	63.8746	1.5	6	90	0.056122024	31	256	182	0.65340906381607\0.65340906381607	TCGA-DU-A5TW/1.3.6.1.4.1.14519.5.2.1.4591.4003.151177506773916107803967580988/1.3.6.1.4.1.14519.5.2.1.4591.4003.999479300108182485740869637956
TCGA-LGG	TCGA-DU	TCGA-DU-A5TW	t1Gd	F		73	POST_COR_T1	1001	2.42768E+15	2/28/1998	Philips Healthcare		Ingenia	SE	2D	5	475.1262	15		63.8746	1.5	6	69	0.191281557	35	308	232	0.53240740299224\0.53240740299224	TCGA-DU-A5TW/1.3.6.1.4.1.14519.5.2.1.4591.4003.151177506773916107803967580988/1.3.6.1.4.1.14519.5.2.1.4591.4003.232216335634337768854876330351
TCGA-LGG	TCGA-DU	TCGA-DU-A5TW	t1	F		73	AX_T1	601	2.42768E+15	2/28/1998	Philips Healthcare		Ingenia	SE	2D	5	422.3344	15		63.8746	1.5	6	69	0.191281542	31	308	232	0.51339286565780\0.51339286565780	TCGA-DU-A5TW/1.3.6.1.4.1.14519.5.2.1.4591.4003.151177506773916107803967580988/1.3.6.1.4.1.14519.5.2.1.4591.4003.774128579215354606663851701946
TCGA-LGG	TCGA-DU	TCGA-DU-A5TW	t2	F		73	AX_T2	401	2.42768E+15	2/28/1998	Philips Healthcare		Ingenia	SE	2D	5	6251.354	100		63.8746	1.5	6	90	0.142390534	31	384	289	0.41071429848670\0.41071429848670	TCGA-DU-A5TW/1.3.6.1.4.1.14519.5.2.1.4591.4003.151177506773916107803967580988/1.3.6.1.4.1.14519.5.2.1.4591.4003.184593776366081692952346590558
TCGA-LGG	TCGA-DU	TCGA-DU-A5TY	flair	F	46	58.967	AXIAL_FLAIR	5	1.23443E+15	7/9/1997	GE MEDICAL SYSTEMS		SIGNA EXCITE	SE\IR	2D	2.5	10002	125.4	2250	127.7322	3	2.5	90	0.5257	72	320	224	0.4688\0.4688	TCGA-DU-A5TY/1.3.6.1.4.1.14519.5.2.1.4591.4003.100575669289135379476050880103/1.3.6.1.4.1.14519.5.2.1.4591.4003.337101806553456931837410645628
TCGA-LGG	TCGA-DU	TCGA-DU-A5TY	t1Gd	F	46	58.967	AX_T1_POST_GD_FLAIR	10	1.23443E+15	7/9/1997	GE MEDICAL SYSTEMS		SIGNA EXCITE	SE\IR	2D	2.5	3104.34	6.356	1238	127.7323	3	2.5	90	1.4813	72	320	224	0.4688\0.4688	TCGA-DU-A5TY/1.3.6.1.4.1.14519.5.2.1.4591.4003.100575669289135379476050880103/1.3.6.1.4.1.14519.5.2.1.4591.4003.245172832153486701331950115937
TCGA-LGG	TCGA-DU	TCGA-DU-A5TY	t1	F	46	58.967	AX_T1_pre_gd	7	1.23443E+15	7/9/1997	GE MEDICAL SYSTEMS		SIGNA EXCITE	SE\IR	2D	5	2111.74	6.356	883	127.7323	3	5	90	1.0891	36	320	224	0.4688\0.4688	TCGA-DU-A5TY/1.3.6.1.4.1.14519.5.2.1.4591.4003.100575669289135379476050880103/1.3.6.1.4.1.14519.5.2.1.4591.4003.240559113302056176406494179303
TCGA-LGG	TCGA-DU	TCGA-DU-A5TY	t2	F	46	58.967	AX_T2_FR-FSE_RF2_150	4	1.23443E+15	7/9/1997	GE MEDICAL SYSTEMS		SIGNA EXCITE	SE	2D	5	4000	103.464	0	127.7322	3	5	90	0.7515	37	320	224	0.4688\0.4688	TCGA-DU-A5TY/1.3.6.1.4.1.14519.5.2.1.4591.4003.100575669289135379476050880103/1.3.6.1.4.1.14519.5.2.1.4591.4003.275135018804563456372957805186
TCGA-LGG	TCGA-DU	TCGA-DU-A6S2	flair	F		136	FLAIR_LongTR	501	1.09867E+15	4/4/1998	Philips Healthcare		Ingenia	IR	2D	2.5	11000	125	2800	127.7472	3	2.5	90	0.221153647	75	320	232	0.625\0.625	TCGA-DU-A6S2/1.3.6.1.4.1.14519.5.2.1.4591.4003.141199374739420890948176927768/1.3.6.1.4.1.14519.5.2.1.4591.4003.293109290224113648309168335981
TCGA-LGG	TCGA-DU	TCGA-DU-A6S2	t1Gd	F		136	POST_AX_T1_BRAIN_LAB_1MM	1102	1.09867E+15	4/4/1998	Philips Medical Systems		Ingenia	GR	3D	1	8.0456	3.685		127.7473	3	1	8	0.024283046	194	240	222	0.9375\0.9375	TCGA-DU-A6S2/1.3.6.1.4.1.14519.5.2.1.4591.4003.141199374739420890948176927768/1.3.6.1.4.1.14519.5.2.1.4591.4003.135589548347858256981695184436
TCGA-LGG	TCGA-DU	TCGA-DU-A6S2	t1	F		136	AX_T1W_IR_TSE	601	1.09867E+15	4/4/1998	Philips Healthcare		Ingenia	IR	2D	5	2000	20	800	127.7472	3	5	90	0.211403593	38	352	262	0.42857143282890\0.42857143282890	TCGA-DU-A6S2/1.3.6.1.4.1.14519.5.2.1.4591.4003.141199374739420890948176927768/1.3.6.1.4.1.14519.5.2.1.4591.4003.179292058159580586521876864087
TCGA-LGG	TCGA-DU	TCGA-DU-A6S2	t2	F		136	AX_T2W_DRIVE	401	1.09867E+15	4/4/1998	Philips Healthcare		Ingenia	SE	2D	2.5	3000	80		127.7472	3	2.5	90	0.325780809	75	420	333	0.42857143282890\0.42857143282890	TCGA-DU-A6S2/1.3.6.1.4.1.14519.5.2.1.4591.4003.141199374739420890948176927768/1.3.6.1.4.1.14519.5.2.1.4591.4003.616725239162147941590320402703
TCGA-LGG	TCGA-DU	TCGA-DU-A6S3	flair	M		170	FLAIR_LongTR	701	1.80811E+15	7/11/1998	Philips Healthcare		Ingenia	IR	2D	2.5	11000	125	2800	127.7472	3	2.5	90	0.207654387	70	332	234	0.625\0.625	TCGA-DU-A6S3/1.3.6.1.4.1.14519.5.2.1.4591.4003.248355137484740846341982351715/1.3.6.1.4.1.14519.5.2.1.4591.4003.292224188967208352325450551998
TCGA-LGG	TCGA-DU	TCGA-DU-A6S3	t1Gd	M		170	POST_AX_T1__3MM	1303	1.80811E+15	7/11/1998	Philips Medical Systems		Ingenia	GR	3D	3	8.0451	3.685		127.7473	3	3	8	0.025058357	64	248	222	0.85714286565780\0.85714286565780	TCGA-DU-A6S3/1.3.6.1.4.1.14519.5.2.1.4591.4003.248355137484740846341982351715/1.3.6.1.4.1.14519.5.2.1.4591.4003.118759847392627168669666819967
TCGA-LGG	TCGA-DU	TCGA-DU-A6S3	t1	M		170	AX_T1W_IR_TSE	801	1.80811E+15	7/11/1998	Philips Healthcare		Ingenia	IR	2D	5	2000	20	800	127.7472	3	5	90	0.211403593	36	368	293	0.390625\0.390625	TCGA-DU-A6S3/1.3.6.1.4.1.14519.5.2.1.4591.4003.248355137484740846341982351715/1.3.6.1.4.1.14519.5.2.1.4591.4003.295602931149011312134948745170
TCGA-LGG	TCGA-DU	TCGA-DU-A6S3	t2	M		170	AX_T2W_DRIVE	601	1.80811E+15	7/11/1998	Philips Healthcare		Ingenia	SE	2D	2.5	3000	80		127.7472	3	2.5	90	0.350840807	70	440	337	0.390625\0.390625	TCGA-DU-A6S3/1.3.6.1.4.1.14519.5.2.1.4591.4003.248355137484740846341982351715/1.3.6.1.4.1.14519.5.2.1.4591.4003.176031418834958083041704052058
TCGA-LGG	TCGA-DU	TCGA-DU-A6S6	flair	F	35	56.699	Ax_FLAIR_2.5mm_for_surgery	4	2.44522E+15	5/21/1992	GE MEDICAL SYSTEMS		SIGNA HDx	IR	2D	2.5	10004	149.544	2200	63.88597	1.5	2.5	90	0.0351	70	256	192	0.9375\0.9375	TCGA-DU-A6S6/1.3.6.1.4.1.14519.5.2.1.4591.4003.157755470529359814735610642529/1.3.6.1.4.1.14519.5.2.1.4591.4003.247515038873804783086202408555
TCGA-LGG	TCGA-DU	TCGA-DU-A6S6	t1Gd	F	35	56.699	Ax_T1_2.5mm_for_surgery	8	2.44522E+15	5/21/1992	GE MEDICAL SYSTEMS		SIGNA HDx	SE	2D	2.5	516.664	13	0	63.886	1.5	2.5	90	0.0541962	70	256	192	0.9375\0.9375	TCGA-DU-A6S6/1.3.6.1.4.1.14519.5.2.1.4591.4003.157755470529359814735610642529/1.3.6.1.4.1.14519.5.2.1.4591.4003.147732384686361520756078194258
TCGA-LGG	TCGA-DU	TCGA-DU-A6S6	t1	F	35	56.699	AXIAL_T1	6	2.44522E+15	5/21/1992	GE MEDICAL SYSTEMS		SIGNA HDx	SE	2D	5	550	14	0	63.88598	1.5	5	90	0.0508993	35	256	192	0.9375\0.9375	TCGA-DU-A6S6/1.3.6.1.4.1.14519.5.2.1.4591.4003.157755470529359814735610642529/1.3.6.1.4.1.14519.5.2.1.4591.4003.238129359139198844765626935458
TCGA-LGG	TCGA-DU	TCGA-DU-A6S6	t2	F	35	56.699	AXIAL_FSE	3	2.44522E+15	5/21/1992	GE MEDICAL SYSTEMS		SIGNA HDx	SE	2D	5	3500	23.688	0	63.88597	1.5	5	90	0.1239	70	256	192	0.9375\0.9375	TCGA-DU-A6S6/1.3.6.1.4.1.14519.5.2.1.4591.4003.157755470529359814735610642529/1.3.6.1.4.1.14519.5.2.1.4591.4003.315196201662757798532330052767
TCGA-LGG	TCGA-DU	TCGA-DU-A6S7	flair	F		57	FLAIR_LongTR	601	3.08723E+15	5/13/1998	Philips Healthcare		Ingenia	IR	2D	2.5	11000	125	2800	127.7472	3	2.5	90	0.234652787	76	320	232	0.625\0.625	TCGA-DU-A6S7/1.3.6.1.4.1.14519.5.2.1.4591.4003.222463746204839267807160781139/1.3.6.1.4.1.14519.5.2.1.4591.4003.153209951824448019360838381161
TCGA-LGG	TCGA-DU	TCGA-DU-A6S7	t1Gd	F		57	POST_AX_T1_BRAIN_LAB_1MM	1102	3.08723E+15	5/13/1998	Philips Medical Systems		Ingenia	GR	3D	1	8.0445	3.685		127.7472	3	1	8	0.024283046	194	240	222	0.9375\0.9375	TCGA-DU-A6S7/1.3.6.1.4.1.14519.5.2.1.4591.4003.222463746204839267807160781139/1.3.6.1.4.1.14519.5.2.1.4591.4003.174567062740096698672017092596
TCGA-LGG	TCGA-DU	TCGA-DU-A6S7	t1	F		57	AX_T1W_IR_TSE	701	3.08723E+15	5/13/1998	Philips Healthcare		Ingenia	IR	2D	5	2000	20	800	127.7472	3	5	90	0.211403564	41	352	262	0.42857143282890\0.42857143282890	TCGA-DU-A6S7/1.3.6.1.4.1.14519.5.2.1.4591.4003.222463746204839267807160781139/1.3.6.1.4.1.14519.5.2.1.4591.4003.318036549395257841823120919980
TCGA-LGG	TCGA-DU	TCGA-DU-A6S7	t2	F		57	AX_T2W_DRIVE	501	3.08723E+15	5/13/1998	Philips Healthcare		Ingenia	SE	2D	2.5	3000	80		127.7472	3	2.5	90	0.325780809	76	420	333	0.42857143282890\0.42857143282890	TCGA-DU-A6S7/1.3.6.1.4.1.14519.5.2.1.4591.4003.222463746204839267807160781139/1.3.6.1.4.1.14519.5.2.1.4591.4003.240158011787928914076090296018
TCGA-LGG	TCGA-DU	TCGA-DU-A6S8	flair	F		82	FLAIR_LongTR	701	3.23035E+15	6/20/1998	Philips Healthcare		Ingenia	IR	2D	2.5	11000	125	2800	127.7473	3	2.5	90	0.221153691	73	320	232	0.625\0.625	TCGA-DU-A6S8/1.3.6.1.4.1.14519.5.2.1.4591.4003.217867204129018050748395707083/1.3.6.1.4.1.14519.5.2.1.4591.4003.204516936842937081704572758933
TCGA-LGG	TCGA-DU	TCGA-DU-A6S8	t1Gd	F		82	POST_AX_T1_BRAIN_LAB_1MM	1302	3.23035E+15	6/20/1998	Philips Medical Systems		Ingenia	GR	3D	1	8.0445	3.685		127.7473	3	1	8	0.024283046	194	240	222	0.9375\0.9375	TCGA-DU-A6S8/1.3.6.1.4.1.14519.5.2.1.4591.4003.217867204129018050748395707083/1.3.6.1.4.1.14519.5.2.1.4591.4003.132452867851880683397728197269
TCGA-LGG	TCGA-DU	TCGA-DU-A6S8	t1	F		82	AX_T1W_IR_TSE	801	3.23035E+15	6/20/1998	Philips Healthcare		Ingenia	IR	2D	5	2000	20	800	127.7473	3	5	90	0.211403742	37	352	262	0.42857143282890\0.42857143282890	TCGA-DU-A6S8/1.3.6.1.4.1.14519.5.2.1.4591.4003.217867204129018050748395707083/1.3.6.1.4.1.14519.5.2.1.4591.4003.889250232340214087576015361713
TCGA-LGG	TCGA-DU	TCGA-DU-A6S8	t2	F		82	AX_T2W_DRIVE	601	3.23035E+15	6/20/1998	Philips Healthcare		Ingenia	SE	2D	2.5	3000	80		127.7473	3	2.5	90	0.325780809	73	420	333	0.42857143282890\0.42857143282890	TCGA-DU-A6S8/1.3.6.1.4.1.14519.5.2.1.4591.4003.217867204129018050748395707083/1.3.6.1.4.1.14519.5.2.1.4591.4003.261214969156460346846015628974
TCGA-LGG	TCGA-EZ	TCGA-EZ-7265A	flair	M	26	97.06878	FLAIR_AXIAL	5	2.5083E+15	9/15/2001	SIEMENS		TrioTim	SE\IR	2D	4	8500	112	2500	123.2411	3	4.8	160	0.594111835	34	256	208	0.859375\0.859375	TCGA-EZ-7265A/1.3.6.1.4.1.14519.5.2.1.8421.4003.118065813881052850602362195650/1.3.6.1.4.1.14519.5.2.1.8421.4003.306492368085941976344452042305
TCGA-LGG	TCGA-EZ	TCGA-EZ-7265A	t1Gd	M	26	97.06878	T1_MPRAGE_AX	7	2.5083E+15	9/15/2001	SIEMENS		TrioTim	GR\IR	3D	1	1900	2.63	900	123.2411	3		9	0.094795003	176	256	256	1\1	TCGA-EZ-7265A/1.3.6.1.4.1.14519.5.2.1.8421.4003.118065813881052850602362195650/1.3.6.1.4.1.14519.5.2.1.8421.4003.471672703542665098957555277296
TCGA-LGG	TCGA-EZ	TCGA-EZ-7265A	t1	M	26	97.06878	T1_FL_COR	14	2.5083E+15	9/15/2001	SIEMENS		TrioTim	GR	2D	5	267	2.46		123.2412	3	6	80	0.707966423	32	320	224	0.71875\0.71875	TCGA-EZ-7265A/1.3.6.1.4.1.14519.5.2.1.8421.4003.118065813881052850602362195650/1.3.6.1.4.1.14519.5.2.1.8421.4003.556090517143317008209723958322
TCGA-LGG	TCGA-EZ	TCGA-EZ-7265A	t2	M	26	97.06878	T2_AX_TSE	6	2.5083E+15	9/15/2001	SIEMENS		TrioTim	SE	2D	3	4780	87		123.2411	3	3	150	0.455488092	55	512	256	0.4296875\0.4296875	TCGA-EZ-7265A/1.3.6.1.4.1.14519.5.2.1.8421.4003.118065813881052850602362195650/1.3.6.1.4.1.14519.5.2.1.8421.4003.105472801741628196191958426767
TCGA-LGG	TCGA-FG	TCGA-FG-5964	flair	M	62	90.71849	MRHG_FLAIR_AX	5	6.20494E+15	5/11/2001	Siemens		Symphony	IR\SE	2D	5	9590	129	2500	63.65037	1.5	6.5	150	0.104002565	26	256	224	0.8984375\0.8984375	TCGA-FG-5964/1.3.6.1.4.1.14519.5.2.1.5826.4003.238471712497372270512892955981/1.3.6.1.4.1.14519.5.2.1.5826.4003.404178152704844737198656421158
TCGA-LGG	TCGA-FG	TCGA-FG-5964	t1Gd	M	62	90.71849	MP_RAGE_AXIAL	7	6.20494E+15	5/11/2001	Siemens		Symphony	IR\GR	3D	1	2160	3.45	1100	63.65038	1.5		15	0.040618185	192	256	256	1\1	TCGA-FG-5964/1.3.6.1.4.1.14519.5.2.1.5826.4003.238471712497372270512892955981/1.3.6.1.4.1.14519.5.2.1.5826.4003.213387747040169487656654950911
TCGA-LGG	TCGA-FG	TCGA-FG-5964	t1	M	62	90.71849	MP_RAGE_AXIAL	2	6.20494E+15	5/11/2001	Siemens		Symphony	IR\GR	3D	1	2160	3.45	1100	63.65037	1.5		15	0.039926529	192	256	256	1\1	TCGA-FG-5964/1.3.6.1.4.1.14519.5.2.1.5826.4003.238471712497372270512892955981/1.3.6.1.4.1.14519.5.2.1.5826.4003.247020117690444362792912756464
TCGA-LGG	TCGA-FG	TCGA-FG-5964	t2	M	62	90.71849	TSE_T2_AXIALS	3	6.20494E+15	5/11/2001	Siemens		Symphony	SE	2D	2	5700	93		63.65036	1.5	2	150	0.185237393	92	256	256	1\1	TCGA-FG-5964/1.3.6.1.4.1.14519.5.2.1.5826.4003.238471712497372270512892955981/1.3.6.1.4.1.14519.5.2.1.5826.4003.272421027569753270309595372912
TCGA-LGG	TCGA-FG	TCGA-FG-6688	flair	F	59	99.79033	FLAIR_AXIALS	3	2.23104E+15	2/15/2002	Siemens		Symphony	IR\SE	2D	4	8790	114	2500	63.49749	1.5	4	150	0.153146952	36	256	208	1\1	TCGA-FG-6688/1.3.6.1.4.1.14519.5.2.1.5826.4003.323004807957544310211707018551/1.3.6.1.4.1.14519.5.2.1.5826.4003.224031693818178176078411166389
TCGA-LGG	TCGA-FG	TCGA-FG-6688	t1Gd	F	59	99.79033	MP_RAGE_AXIAL_PG	7	2.23104E+15	2/15/2002	Siemens		Symphony	IR\GR	3D	1	2160	3.45	1100	63.49749	1.5		15	0.058381364	192	256	256	1\1	TCGA-FG-6688/1.3.6.1.4.1.14519.5.2.1.5826.4003.323004807957544310211707018551/1.3.6.1.4.1.14519.5.2.1.5826.4003.305496105341737447254851785503
TCGA-LGG	TCGA-FG	TCGA-FG-6688	t1	F	59	99.79033	MP_RAGE_AXIAL	2	2.23104E+15	2/15/2002	Siemens		Symphony	IR\GR	3D	1	2160	3.45	1100	63.49749	1.5		15	0.058381364	192	256	256	1\1	TCGA-FG-6688/1.3.6.1.4.1.14519.5.2.1.5826.4003.323004807957544310211707018551/1.3.6.1.4.1.14519.5.2.1.5826.4003.315562198227737027776970803095
TCGA-LGG	TCGA-FG	TCGA-FG-6688	t2	F	59	99.79033	MRHG_T2_AXIALS	5	2.23104E+15	2/15/2002	Siemens		Symphony	SE	2D	5	4190	94		63.49745	1.5	5.5	150	0.462320268	26	256	224	0.8984375\0.8984375	TCGA-FG-6688/1.3.6.1.4.1.14519.5.2.1.5826.4003.323004807957544310211707018551/1.3.6.1.4.1.14519.5.2.1.5826.4003.222396944059496713742105306085
TCGA-LGG	TCGA-FG	TCGA-FG-6689	flair	M	30	83.9146	FLAIR_AXIALS	3	7.53987E+15	3/26/2002	Siemens		Symphony	IR\SE	2D	4	9980	114	2500	63.49709	1.5	4	150	0.127931744	48	256	256	1\1	TCGA-FG-6689/1.3.6.1.4.1.14519.5.2.1.5826.4003.844510743144337710585638347983/1.3.6.1.4.1.14519.5.2.1.5826.4003.194766601320226262498310199471
TCGA-LGG	TCGA-FG	TCGA-FG-6689	t1Gd	M	30	83.9146	MP_RAGE_AXIAL	6	7.53987E+15	3/26/2002	Siemens		Symphony	IR\GR	3D	1	2160	3.45	1100	63.49709	1.5		15	0.041316584	192	256	256	1\1	TCGA-FG-6689/1.3.6.1.4.1.14519.5.2.1.5826.4003.844510743144337710585638347983/1.3.6.1.4.1.14519.5.2.1.5826.4003.791850473131565744847058876558
TCGA-LGG	TCGA-FG	TCGA-FG-6689	t1	M	30	83.9146	MP_RAGE_AXIAL	5	7.53987E+15	3/26/2002	Siemens		Symphony	IR\GR	3D	1	2160	3.45	1100	63.49709	1.5		15	0.041316584	192	256	256	1\1	TCGA-FG-6689/1.3.6.1.4.1.14519.5.2.1.5826.4003.844510743144337710585638347983/1.3.6.1.4.1.14519.5.2.1.5826.4003.321356076384546982705007068721
TCGA-LGG	TCGA-FG	TCGA-FG-6689	t2	M	30	83.9146	T2_AXIALS	2	7.53987E+15	3/26/2002	Siemens		Symphony	SE	2D	4	4300	91		63.49709	1.5	4	150	0.297007412	48	256	256	1\1	TCGA-FG-6689/1.3.6.1.4.1.14519.5.2.1.5826.4003.844510743144337710585638347983/1.3.6.1.4.1.14519.5.2.1.5826.4003.502089644490762861281022782793
TCGA-LGG	TCGA-FG	TCGA-FG-6691	flair	F	23	81.64664	FLAIR_AXIALS	4	7.02986E+15	4/5/2002	Siemens		Symphony	IR\SE	2D	4	9980	114	2500	63.49715	1.5	4	150	0.146310553	48	256	256	1\1	TCGA-FG-6691/1.3.6.1.4.1.14519.5.2.1.5826.4003.329102386198066869568471530457/1.3.6.1.4.1.14519.5.2.1.5826.4003.256505097264461536420482208985
TCGA-LGG	TCGA-FG	TCGA-FG-6691	t1Gd	F	23	81.64664	MP_RAGE_AXIAL	6	7.02986E+15	4/5/2002	Siemens		Symphony	IR\GR	3D	1	2160	3.45	1100	63.49715	1.5		15	0.047252156	192	256	256	1\1	TCGA-FG-6691/1.3.6.1.4.1.14519.5.2.1.5826.4003.329102386198066869568471530457/1.3.6.1.4.1.14519.5.2.1.5826.4003.656879804613220409889901206605
TCGA-LGG	TCGA-FG	TCGA-FG-6691	t1	F	23	81.64664	MP_RAGE_AXIAL	2	7.02986E+15	4/5/2002	Siemens		Symphony	IR\GR	3D	1	2160	3.45	1100	63.49715	1.5		15	0.047252156	192	256	256	1\1	TCGA-FG-6691/1.3.6.1.4.1.14519.5.2.1.5826.4003.329102386198066869568471530457/1.3.6.1.4.1.14519.5.2.1.5826.4003.529788387304099813337988254846
TCGA-LGG	TCGA-FG	TCGA-FG-6691	t2	F	23	81.64664	T2_AXIALS	3	7.02986E+15	4/5/2002	Siemens		Symphony	SE	2D	4	4300	91		63.49715	1.5	4	150	0.339675784	48	256	256	1\1	TCGA-FG-6691/1.3.6.1.4.1.14519.5.2.1.5826.4003.329102386198066869568471530457/1.3.6.1.4.1.14519.5.2.1.5826.4003.943440743719674101532157437216
TCGA-LGG	TCGA-FG	TCGA-FG-6692	flair	M	63	65.31731	MRHR_FLAIR_AX	5	7.0351E+15	6/6/2002	Siemens		Avanto	SE\IR	2D	5	9000	109	2500	63.62652	1.5	5.5	150	0.124829227	26	256	208	0.8984375\0.8984375	TCGA-FG-6692/1.3.6.1.4.1.14519.5.2.1.5826.4003.319380682030030732733259154931/1.3.6.1.4.1.14519.5.2.1.5826.4003.192830341660611753009089717349
TCGA-LGG	TCGA-FG	TCGA-FG-6692	t1Gd	M	63	65.31731	MRHR_T1_SAG_POST_GAD	14	7.0351E+15	6/6/2002	Siemens		Avanto	SE	2D	5	690	17		63.62568	1.5	6.15	90	0.430428356	25	256	256	0.8984375\0.8984375	TCGA-FG-6692/1.3.6.1.4.1.14519.5.2.1.5826.4003.319380682030030732733259154931/1.3.6.1.4.1.14519.5.2.1.5826.4003.186672506079531283321680579916
TCGA-LGG	TCGA-FG	TCGA-FG-6692	t1	M	63	65.31731	T1_AX	10	7.0351E+15	6/6/2002	Siemens		Avanto	SE	2D	5	529	12		63.62568	1.5	5.5	70	0.531351037	26	256	224	0.8984375\0.8984375	TCGA-FG-6692/1.3.6.1.4.1.14519.5.2.1.5826.4003.319380682030030732733259154931/1.3.6.1.4.1.14519.5.2.1.5826.4003.186281903647307431209277892227
TCGA-LGG	TCGA-FG	TCGA-FG-6692	t2	M	63	65.31731	MRHR_T2_AX	2	7.0351E+15	6/6/2002	Siemens		Avanto	SE	2D	5	5000	91		63.6265	1.5	5.5	150	0.301251205	26	256	224	0.8984375\0.8984375	TCGA-FG-6692/1.3.6.1.4.1.14519.5.2.1.5826.4003.319380682030030732733259154931/1.3.6.1.4.1.14519.5.2.1.5826.4003.234091914086236121186030369038
TCGA-LGG	TCGA-FG	TCGA-FG-7634	flair	M	28	83.9146	FLAIR_AX	3	2.63434E+15	1/28/2000	Siemens		Verio	SE\IR	2D	4	9000	94	2500	123.2494	3	5.2	150	0.144740377	27	256	169	0.8984375\0.8984375	TCGA-FG-7634/1.3.6.1.4.1.14519.5.2.1.5826.4003.112089609616478176328783523271/1.3.6.1.4.1.14519.5.2.1.5826.4003.181265280864825404459789253505
TCGA-LGG	TCGA-FG	TCGA-FG-7634	t1Gd	M	28	83.9146	VOLUMETRIC_AXIAL	9	2.63434E+15	1/28/2000	Siemens		Verio	GR\IR	3D	1	5500	2.75	900	123.2494	3		9	0.016694271	192	256	251	1\1	TCGA-FG-7634/1.3.6.1.4.1.14519.5.2.1.5826.4003.112089609616478176328783523271/1.3.6.1.4.1.14519.5.2.1.5826.4003.120522077294140993470551701861
TCGA-LGG	TCGA-FG	TCGA-FG-7634	t1	M	28	83.9146	VOLUMETRIC_AXIAL	8	2.63434E+15	1/28/2000	Siemens		Verio	GR\IR	3D	1	1900	2.75	900	123.2494	3		9	0.048193246	192	256	251	1\1	TCGA-FG-7634/1.3.6.1.4.1.14519.5.2.1.5826.4003.112089609616478176328783523271/1.3.6.1.4.1.14519.5.2.1.5826.4003.162682992466409363718204747302
TCGA-LGG	TCGA-FG	TCGA-FG-7634	t2	M	28	83.9146	T2_AX	2	2.63434E+15	1/28/2000	Siemens		Verio	SE	2D	4	5000	96		123.2494	3	5.2	150	0.399312478	27	320	211	0.71875\0.71875	TCGA-FG-7634/1.3.6.1.4.1.14519.5.2.1.5826.4003.112089609616478176328783523271/1.3.6.1.4.1.14519.5.2.1.5826.4003.290432067045012638044885643345
TCGA-LGG	TCGA-FG	TCGA-FG-7643	flair							//																			TCGA-FG-7643/1.3.6.1.4.1.14519.5.2.1.5826.4003.543441953908033691486687933312/1.3.6.1.4.1.14519.5.2.1.5826.4003.566940196246926075314106414146
TCGA-LGG	TCGA-FG	TCGA-FG-7643	t1							//																			TCGA-FG-7643/1.3.6.1.4.1.14519.5.2.1.5826.4003.543441953908033691486687933312/1.3.6.1.4.1.14519.5.2.1.5826.4003.229152749348243416870891129832
TCGA-LGG	TCGA-FG	TCGA-FG-7643	t1Gd							//																			TCGA-FG-7643/1.3.6.1.4.1.14519.5.2.1.5826.4003.543441953908033691486687933312/1.3.6.1.4.1.14519.5.2.1.5826.4003.216434171023938431138472406051
TCGA-LGG	TCGA-FG	TCGA-FG-7643	t2							//																			TCGA-FG-7643/1.3.6.1.4.1.14519.5.2.1.5826.4003.543441953908033691486687933312/1.3.6.1.4.1.14519.5.2.1.5826.4003.179485783112349332939448183328
TCGA-LGG	TCGA-FG	TCGA-FG-A4MT	flair	F	27	61.68857	FLAIR_AXIALS	3	2.45097E+15	2/12/2002	Siemens		Symphony	IR\SE	2D	3	9050	114	2500	63.64688	1.5	3	150	0.10171707	50	256	192	1\1	TCGA-FG-A4MT/1.3.6.1.4.1.14519.5.2.1.5826.4003.329714707736603564770655892800/1.3.6.1.4.1.14519.5.2.1.5826.4003.108791662326014870116459702285
TCGA-LGG	TCGA-FG	TCGA-FG-A4MT	t1Gd	F	27	61.68857	MP_RAGE_AXIAL	6	2.45097E+15	2/12/2002	Siemens		Symphony	IR\GR	3D	1	2160	3.45	1100	63.64688	1.5		15	0.03827221	192	256	256	1\1	TCGA-FG-A4MT/1.3.6.1.4.1.14519.5.2.1.5826.4003.329714707736603564770655892800/1.3.6.1.4.1.14519.5.2.1.5826.4003.810239147090844490746341825104
TCGA-LGG	TCGA-FG	TCGA-FG-A4MT	t1	F	27	61.68857	MP_RAGE_AXIAL	2	2.45097E+15	2/12/2002	Siemens		Symphony	IR\GR	3D	1	2160	3.45	1100	63.64688	1.5		15	0.03827221	192	256	256	1\1	TCGA-FG-A4MT/1.3.6.1.4.1.14519.5.2.1.5826.4003.329714707736603564770655892800/1.3.6.1.4.1.14519.5.2.1.5826.4003.208492381534690436622701341883
TCGA-LGG	TCGA-FG	TCGA-FG-A4MT	t2	F	27	61.68857	TSE_T2_AXIALS	4	2.45097E+15	2/12/2002	Siemens		Symphony	SE	2D	3	4230	90		63.64687	1.5	3	150	0.12996757	50	256	192	1\1	TCGA-FG-A4MT/1.3.6.1.4.1.14519.5.2.1.5826.4003.329714707736603564770655892800/1.3.6.1.4.1.14519.5.2.1.5826.4003.323518390920997145878509984446
TCGA-LGG	TCGA-FG	TCGA-FG-A6IZ	flair	M	60	79.37867	FLAIR_AX	11	6.76664E+15	2/20/2004	SIEMENS		Avanto	SE\IR	2D	3	9430	109	2500	63.62654	1.5	3	150	0.195461899	60	256	232	1\1	TCGA-FG-A6IZ/1.3.6.1.4.1.14519.5.2.1.5826.4003.294186107439947951642908363316/1.3.6.1.4.1.14519.5.2.1.5826.4003.702011097062990231893856395088
TCGA-LGG	TCGA-FG	TCGA-FG-A6IZ	t1Gd	M	60	79.37867	MP_RAGE_AXIAL	13	6.76664E+15	2/20/2004	SIEMENS		Avanto	GR\IR	3D	1	2160	2.81	1100	63.62654	1.5		15	0.048093966	192	256	256	1\1	TCGA-FG-A6IZ/1.3.6.1.4.1.14519.5.2.1.5826.4003.294186107439947951642908363316/1.3.6.1.4.1.14519.5.2.1.5826.4003.331410843391598749669849855225
TCGA-LGG	TCGA-FG	TCGA-FG-A6IZ	t1	M	60	79.37867	MP_RAGE_AXIAL_VOLUMETRIC	12	6.76664E+15	2/20/2004	SIEMENS		Avanto	GR\IR	3D	1	2160	2.81	1100	63.62654	1.5		15	0.048093966	192	256	256	1\1	TCGA-FG-A6IZ/1.3.6.1.4.1.14519.5.2.1.5826.4003.294186107439947951642908363316/1.3.6.1.4.1.14519.5.2.1.5826.4003.277684855659716713037896125841
TCGA-LGG	TCGA-FG	TCGA-FG-A6IZ	t2	M	60	79.37867	MRHR_T2_AX	10	6.76664E+15	2/20/2004	SIEMENS		Avanto	SE	2D	3	5000	91		63.62649	1.5	3	150	0.374650202	60	256	232	1\1	TCGA-FG-A6IZ/1.3.6.1.4.1.14519.5.2.1.5826.4003.294186107439947951642908363316/1.3.6.1.4.1.14519.5.2.1.5826.4003.178027193899941193791707458901
TCGA-LGG	TCGA-FG	TCGA-FG-A713	flair	F	74	79.37867	FLAIR_axial	4	8.51776E+15	7/9/2004	SIEMENS		Skyra	SE\IR	2D	3	9000	94	2498.5	123.2567	3	3	150	0.657520147	64	256	208	1\1	TCGA-FG-A713/1.3.6.1.4.1.14519.5.2.1.5826.4003.239451224442205099709649006810/1.3.6.1.4.1.14519.5.2.1.5826.4003.103848826726158954491300808517
TCGA-LGG	TCGA-FG	TCGA-FG-A713	t1Gd	F	74	79.37867	t1_mprage_tra_Gd	11	8.51776E+15	7/9/2004	SIEMENS		Skyra	GR\IR	3D	1	2200	2.45	900	123.2567	3		8	0.070773934	192	256	256	1\1	TCGA-FG-A713/1.3.6.1.4.1.14519.5.2.1.5826.4003.239451224442205099709649006810/1.3.6.1.4.1.14519.5.2.1.5826.4003.780276319244830396853648645030
TCGA-LGG	TCGA-FG	TCGA-FG-A713	t1	F	74	79.37867	t1_mprage_tra	2	8.51776E+15	7/9/2004	SIEMENS		Skyra	GR\IR	3D	1	2200	2.45	900	123.2567	3		8	0.070773934	192	256	256	1\1	TCGA-FG-A713/1.3.6.1.4.1.14519.5.2.1.5826.4003.239451224442205099709649006810/1.3.6.1.4.1.14519.5.2.1.5826.4003.291884242340469108197706129148
TCGA-LGG	TCGA-FG	TCGA-FG-A713	t2	F	74	79.37867	T2_axial	3	8.51776E+15	7/9/2004	SIEMENS		Skyra	SE	2D	3	6000	104		123.2567	3	3	150	0.734946399	64	256	208	1\1	TCGA-FG-A713/1.3.6.1.4.1.14519.5.2.1.5826.4003.239451224442205099709649006810/1.3.6.1.4.1.14519.5.2.1.5826.4003.127828297999920935064228260226
TCGA-LGG	TCGA-HT	TCGA-HT-7473	flair	M	28	68.039	Ax_Flair_irFSE_H	8	6.82232E+15	8/26/1997	GE MEDICAL SYSTEMS		SIGNA EXCITE	SE\IR	2D	5	9002	121.752	2200	63.86294	1.5	5	90	0.9491	32	256	224	0.9375\0.9375	TCGA-HT-7473/1.3.6.1.4.1.14519.5.2.1.2531.4003.339148669016426179130214847712/1.3.6.1.4.1.14519.5.2.1.2531.4003.235597736552717483361225257005
TCGA-LGG	TCGA-HT	TCGA-HT-7473	t1Gd	M	28	68.039	AX_3D_SPGR+C	12	6.82232E+15	8/26/1997	GE MEDICAL SYSTEMS		SIGNA EXCITE	GR	3D	2	9.428	4.54	450	63.86294	1.5	1	20	0.293884	212	320	192	0.4688\0.4688	TCGA-HT-7473/1.3.6.1.4.1.14519.5.2.1.2531.4003.339148669016426179130214847712/1.3.6.1.4.1.14519.5.2.1.2531.4003.333980853070789124033221676794
TCGA-LGG	TCGA-HT	TCGA-HT-7473	t1	M	28	68.039	AX_3D_SPGR	5	6.82232E+15	8/26/1997	GE MEDICAL SYSTEMS		SIGNA EXCITE	GR	3D	2	9.428	4.54	450	63.86294	1.5	1	20	0.293884	212	320	192	0.4688\0.4688	TCGA-HT-7473/1.3.6.1.4.1.14519.5.2.1.2531.4003.339148669016426179130214847712/1.3.6.1.4.1.14519.5.2.1.2531.4003.233544485840464225800386044386
TCGA-LGG	TCGA-HT	TCGA-HT-7473	t2	M	28	68.039	3-pl_T2*_FGRE_S	1	6.82232E+15	8/26/1997	GE MEDICAL SYSTEMS		SIGNA EXCITE	RM	2D	5	5.104	1.46	0	63.86291	1.5	10	30	0.242037	9	256	128	0.9375\0.9375	TCGA-HT-7473/1.3.6.1.4.1.14519.5.2.1.2531.4003.339148669016426179130214847712/1.3.6.1.4.1.14519.5.2.1.2531.4003.479342730015683457990678269518
TCGA-LGG	TCGA-HT	TCGA-HT-7475	flair	M	67	106	Ax_Flair_irFSE_H	6	2.30316E+15	9/18/1997	GE MEDICAL SYSTEMS		SIGNA EXCITE	SE\IR	2D	5	9002	121.524	2200	63.86295	1.5	5	90	1.1742	32	256	224	0.9375\0.9375	TCGA-HT-7475/1.3.6.1.4.1.14519.5.2.1.2531.4003.306463775653742362436970431460/1.3.6.1.4.1.14519.5.2.1.2531.4003.174114720577685561285251606527
TCGA-LGG	TCGA-HT	TCGA-HT-7475	t1Gd	M	67	106	AX_3D_SPGR+C	11	2.30316E+15	9/18/1997	GE MEDICAL SYSTEMS		SIGNA EXCITE	GR	3D	2	9.388	4.488	450	63.86295	1.5	1	20	0.325404	212	320	192	0.5078\0.5078	TCGA-HT-7475/1.3.6.1.4.1.14519.5.2.1.2531.4003.306463775653742362436970431460/1.3.6.1.4.1.14519.5.2.1.2531.4003.241979134763350176634365733028
TCGA-LGG	TCGA-HT	TCGA-HT-7475	t1	M	67	106	AX_3D_SPGR	5	2.30316E+15	9/18/1997	GE MEDICAL SYSTEMS		SIGNA EXCITE	GR	3D	2	9.388	4.488	450	63.86295	1.5	1	20	0.325404	212	320	192	0.5078\0.5078	TCGA-HT-7475/1.3.6.1.4.1.14519.5.2.1.2531.4003.306463775653742362436970431460/1.3.6.1.4.1.14519.5.2.1.2531.4003.221279757470705906693181410807
TCGA-LGG	TCGA-HT	TCGA-HT-7475	t2	M	67	106	3-pl_T2*_FGRE_S	1	2.30316E+15	9/18/1997	GE MEDICAL SYSTEMS		SIGNA EXCITE	RM	2D	5	5.104	1.46	0	63.86294	1.5	10	30	0.299437	9	256	128	0.9375\0.9375	TCGA-HT-7475/1.3.6.1.4.1.14519.5.2.1.2531.4003.306463775653742362436970431460/1.3.6.1.4.1.14519.5.2.1.2531.4003.187157052835080951633840760211
TCGA-LGG	TCGA-HT	TCGA-HT-7602	flair	M	21	68.039	Ax_Flair_irFSE_H	4	5.214E+15	11/3/1995	GE MEDICAL SYSTEMS		SIGNA EXCITE	SE\IR	2D	5	9002	121.98	2200	63.86308	1.5	5	90	0.8304	28	256	224	0.9375\0.9375	TCGA-HT-7602/1.3.6.1.4.1.14519.5.2.1.2531.4003.204317324583381684838188896833/1.3.6.1.4.1.14519.5.2.1.2531.4003.935616829107968413051604663620
TCGA-LGG	TCGA-HT	TCGA-HT-7602	t1Gd	M	21	68.039	AX_3D_SPGR	10	5.214E+15	11/3/1995	GE MEDICAL SYSTEMS		SIGNA EXCITE	GR	3D	2	9.344	4.476	450	63.86308	1.5	2	20	0.295292	106	320	192	0.5078\0.5078	TCGA-HT-7602/1.3.6.1.4.1.14519.5.2.1.2531.4003.204317324583381684838188896833/1.3.6.1.4.1.14519.5.2.1.2531.4003.171677757382127546017310356128
TCGA-LGG	TCGA-HT	TCGA-HT-7602	t1	M	21	68.039	AX_T1	7	5.214E+15	11/3/1995	GE MEDICAL SYSTEMS		SIGNA EXCITE	SE	2D	5	666.664	17	0	63.86308	1.5	7.5	90	1.09596	20	256	192	0.9375\0.9375	TCGA-HT-7602/1.3.6.1.4.1.14519.5.2.1.2531.4003.204317324583381684838188896833/1.3.6.1.4.1.14519.5.2.1.2531.4003.242761211097040357467172205806
TCGA-LGG	TCGA-HT	TCGA-HT-7602	t2	M	21	68.039	3-pl_T2*_FGRE_S	1	5.214E+15	11/3/1995	GE MEDICAL SYSTEMS		SIGNA EXCITE	RM	2D	5	5.104	1.46	0	63.86306	1.5	10	30	0.242037	9	256	128	0.9375\0.9375	TCGA-HT-7602/1.3.6.1.4.1.14519.5.2.1.2531.4003.204317324583381684838188896833/1.3.6.1.4.1.14519.5.2.1.2531.4003.235986455034628242616079902294
TCGA-LGG	TCGA-HT	TCGA-HT-7616	flair	M	75	106.594	Ax_Flair_irFSE_H	8	3.14339E+15	8/13/1994	GE MEDICAL SYSTEMS		SIGNA EXCITE	IR	2D	5	9002	121.676	2200	63.86317	1.5	5	90	1.1773	30	256	224	0.9375\0.9375	TCGA-HT-7616/1.3.6.1.4.1.14519.5.2.1.2531.4003.282021490454584883892357674533/1.3.6.1.4.1.14519.5.2.1.2531.4003.246158931363136936871049530437
TCGA-LGG	TCGA-HT	TCGA-HT-7616	t1Gd	M	75	106.594	AX_3D_SPGR+C	10	3.14339E+15	8/13/1994	GE MEDICAL SYSTEMS		SIGNA EXCITE	GR	3D	2	23	4.6	0	63.86318	1.5	2	26	0.135368	92	256	160	1.0156\1.0156	TCGA-HT-7616/1.3.6.1.4.1.14519.5.2.1.2531.4003.282021490454584883892357674533/1.3.6.1.4.1.14519.5.2.1.2531.4003.187307241768156692958489188568
TCGA-LGG	TCGA-HT	TCGA-HT-7616	t1	M	75	106.594	AX_3D_SPGR	4	3.14339E+15	8/13/1994	GE MEDICAL SYSTEMS		SIGNA EXCITE	GR	3D	2	23	4.6	0	63.86317	1.5	2	26	0.499826	92	256	160	1.0156\1.0156	TCGA-HT-7616/1.3.6.1.4.1.14519.5.2.1.2531.4003.282021490454584883892357674533/1.3.6.1.4.1.14519.5.2.1.2531.4003.283487006692286623042316208228
TCGA-LGG	TCGA-HT	TCGA-HT-7616	t2	M	75	106.594	3-pl_T2*_FGRE_S	1	3.14339E+15	8/13/1994	GE MEDICAL SYSTEMS		SIGNA EXCITE	RM	2D	5	5.104	1.46	0	63.86315	1.5	10	30	0.300241	9	256	128	0.9375\0.9375	TCGA-HT-7616/1.3.6.1.4.1.14519.5.2.1.2531.4003.282021490454584883892357674533/1.3.6.1.4.1.14519.5.2.1.2531.4003.318187228786416199345869204369
TCGA-LGG	TCGA-HT	TCGA-HT-7680	flair	F	32	61.235	Ax_Flair_irFSE_H	6	2.71385E+15	2/2/1997	GE MEDICAL SYSTEMS		SIGNA EXCITE	SE\IR	2D	5	9002	121.524	2200	63.86295	1.5	5	90	0.9023	32	256	224	0.9375\0.9375	TCGA-HT-7680/1.3.6.1.4.1.14519.5.2.1.2531.4003.231158914302938549165293612810/1.3.6.1.4.1.14519.5.2.1.2531.4003.135075318828198110810035695027
TCGA-LGG	TCGA-HT	TCGA-HT-7680	t1Gd	F	32	61.235	C+AX_3D_SPGR	12	2.71385E+15	2/2/1997	GE MEDICAL SYSTEMS		SIGNA EXCITE	GR	3D	2	9.336	4.472	450	63.86295	1.5	2	20	0.286776	106	320	192	0.5078\0.5078	TCGA-HT-7680/1.3.6.1.4.1.14519.5.2.1.2531.4003.231158914302938549165293612810/1.3.6.1.4.1.14519.5.2.1.2531.4003.234480128631300125982061092286
TCGA-LGG	TCGA-HT	TCGA-HT-7680	t1	F	32	61.235	AX_T1	5	2.71385E+15	2/2/1997	GE MEDICAL SYSTEMS		SIGNA EXCITE	SE	2D	5	683.332	17	0	63.86295	1.5	7.5	90	1.06725	21	256	192	0.9375\0.9375	TCGA-HT-7680/1.3.6.1.4.1.14519.5.2.1.2531.4003.231158914302938549165293612810/1.3.6.1.4.1.14519.5.2.1.2531.4003.201090454229010635061782438562
TCGA-LGG	TCGA-HT	TCGA-HT-7680	t2	F	32	61.235	COR_GRE_T2	7	2.71385E+15	2/2/1997	GE MEDICAL SYSTEMS		SIGNA EXCITE	GR	2D	5	550	35	0	63.86295	1.5	7.5	20	0.00509736	48	256	192	0.9375\0.9375	TCGA-HT-7680/1.3.6.1.4.1.14519.5.2.1.2531.4003.231158914302938549165293612810/1.3.6.1.4.1.14519.5.2.1.2531.4003.109629960664151193660949097585
TCGA-LGG	TCGA-HT	TCGA-HT-7684	flair	M	58	90.718	Ax_Flair_irFSE_H	7	7.00231E+15	8/16/1995	GE MEDICAL SYSTEMS		SIGNA EXCITE	SE\IR	2D	5	9002	121.828	2200	63.86312	1.5	5	90	1.0896	32	256	224	0.9375\0.9375	TCGA-HT-7684/1.3.6.1.4.1.14519.5.2.1.2531.4003.186054156253219473364551929908/1.3.6.1.4.1.14519.5.2.1.2531.4003.326875482336787381981274326694
TCGA-LGG	TCGA-HT	TCGA-HT-7684	t1Gd	M	58	90.718	POST_AX_3D_SPGR	12	7.00231E+15	8/16/1995	GE MEDICAL SYSTEMS		SIGNA EXCITE	GR	3D	2	9.364	4.48	450	63.86311	1.5	2	20	0.319206	106	320	192	0.5078\0.5078	TCGA-HT-7684/1.3.6.1.4.1.14519.5.2.1.2531.4003.186054156253219473364551929908/1.3.6.1.4.1.14519.5.2.1.2531.4003.324057765300350182002585590682
TCGA-LGG	TCGA-HT	TCGA-HT-7684	t1	M	58	90.718	AX_T1	4	7.00231E+15	8/16/1995	GE MEDICAL SYSTEMS		SIGNA EXCITE	SE	2D	5	450	17	0	63.86312	1.5	7.5	90	1.2134	25	256	192	0.9375\0.9375	TCGA-HT-7684/1.3.6.1.4.1.14519.5.2.1.2531.4003.186054156253219473364551929908/1.3.6.1.4.1.14519.5.2.1.2531.4003.316825701703669893555766169473
TCGA-LGG	TCGA-HT	TCGA-HT-7684	t2	M	58	90.718	3-pl_T2*_FGRE_S	1	7.00231E+15	8/16/1995	GE MEDICAL SYSTEMS		SIGNA EXCITE	RM	2D	5	5.104	1.46	0	63.86308	1.5	10	30	0.277876	9	256	128	0.9375\0.9375	TCGA-HT-7684/1.3.6.1.4.1.14519.5.2.1.2531.4003.186054156253219473364551929908/1.3.6.1.4.1.14519.5.2.1.2531.4003.248152573378052966975211701886
TCGA-LGG	TCGA-HT	TCGA-HT-7686	flair	F	29	64.41	Ax_Flair_irFSE_H	7	2.71269E+15	6/29/1995	GE MEDICAL SYSTEMS		SIGNA EXCITE	IR	2D	5	9002	121.6	2200	63.86313	1.5	5	90	0.8089	28	256	224	0.9375\0.9375	TCGA-HT-7686/1.3.6.1.4.1.14519.5.2.1.2531.4003.244405970705727872326343639199/1.3.6.1.4.1.14519.5.2.1.2531.4003.164288781276096644376995835931
TCGA-LGG	TCGA-HT	TCGA-HT-7686	t1Gd	F	29	64.41	AX_3D_SPGR	10	2.71269E+15	6/29/1995	GE MEDICAL SYSTEMS		SIGNA EXCITE	GR	3D	2	9.336	4.472	450	63.86313	1.5	2	20	0.29382	106	320	192	0.5078\0.5078	TCGA-HT-7686/1.3.6.1.4.1.14519.5.2.1.2531.4003.244405970705727872326343639199/1.3.6.1.4.1.14519.5.2.1.2531.4003.617892831080967474932515439020
TCGA-LGG	TCGA-HT	TCGA-HT-7686	t1	F	29	64.41	AX_T1	5	2.71269E+15	6/29/1995	GE MEDICAL SYSTEMS		SIGNA EXCITE	SE	2D	5	666.664	17	0	63.86313	1.5	7.5	90	1.0675	20	256	192	0.9375\0.9375	TCGA-HT-7686/1.3.6.1.4.1.14519.5.2.1.2531.4003.244405970705727872326343639199/1.3.6.1.4.1.14519.5.2.1.2531.4003.244921667827712799297721626042
TCGA-LGG	TCGA-HT	TCGA-HT-7686	t2	F	29	64.41	COR_GRE_T2	9	2.71269E+15	6/29/1995	GE MEDICAL SYSTEMS		SIGNA EXCITE	GR	2D	5	467	35	0	63.86313	1.5	7.5	20	0.0051273	40	256	192	0.9375\0.9375	TCGA-HT-7686/1.3.6.1.4.1.14519.5.2.1.2531.4003.244405970705727872326343639199/1.3.6.1.4.1.14519.5.2.1.2531.4003.198314247552871385304859040626
TCGA-LGG	TCGA-HT	TCGA-HT-7690	flair	M	29	113.398	Ax_Flair_irFSE_H	7	9.81991E+15	3/12/1996	GE MEDICAL SYSTEMS		SIGNA EXCITE	SE\IR	2D	5	9002	121.524	2200	63.86304	1.5	5	90	1.2128	32	256	224	0.9375\0.9375	TCGA-HT-7690/1.3.6.1.4.1.14519.5.2.1.2531.4003.244290172471315468690716464118/1.3.6.1.4.1.14519.5.2.1.2531.4003.813549114384769615209313988757
TCGA-LGG	TCGA-HT	TCGA-HT-7690	t1Gd	M	29	113.398	C+AX_3D_SPGR	12	9.81991E+15	3/12/1996	GE MEDICAL SYSTEMS		SIGNA EXCITE	GR	3D	2	9.396	4.488	450	63.86304	1.5	2	20	0.330267	106	320	192	0.5078\0.5078	TCGA-HT-7690/1.3.6.1.4.1.14519.5.2.1.2531.4003.244290172471315468690716464118/1.3.6.1.4.1.14519.5.2.1.2531.4003.166257326692653730359194508511
TCGA-LGG	TCGA-HT	TCGA-HT-7690	t1	M	29	113.398	AX_T1	5	9.81991E+15	3/12/1996	GE MEDICAL SYSTEMS		SIGNA EXCITE	SE	2D	5	783.332	17	0	63.86304	1.5	7.5	90	1.22579	24	256	192	0.9375\0.9375	TCGA-HT-7690/1.3.6.1.4.1.14519.5.2.1.2531.4003.244290172471315468690716464118/1.3.6.1.4.1.14519.5.2.1.2531.4003.250335993934930482214933635923
TCGA-LGG	TCGA-HT	TCGA-HT-7690	t2	M	29	113.398	COR_GRE_T2	8	9.81991E+15	3/12/1996	GE MEDICAL SYSTEMS		SIGNA EXCITE	GR	2D	5	567	35	0	63.86304	1.5	7.5	20	0.00719977	52	256	192	0.9375\0.9375	TCGA-HT-7690/1.3.6.1.4.1.14519.5.2.1.2531.4003.244290172471315468690716464118/1.3.6.1.4.1.14519.5.2.1.2531.4003.114844545936671776285659054250
TCGA-LGG	TCGA-HT	TCGA-HT-7692	flair	M	43	70.307	Ax_Flair_irFSE_H	6	1.49904E+15	7/24/1996	GE MEDICAL SYSTEMS		SIGNA EXCITE	SE\IR	2D	5	9002	121.828	2200	63.86302	1.5	5	90	0.9641	30	256	224	0.9375\0.9375	TCGA-HT-7692/1.3.6.1.4.1.14519.5.2.1.2531.4003.211398814274191438581429613406/1.3.6.1.4.1.14519.5.2.1.2531.4003.222350740835087785989639436055
TCGA-LGG	TCGA-HT	TCGA-HT-7692	t1	M	43	70.307	AX_T1	5	1.49904E+15	7/24/1996	GE MEDICAL SYSTEMS		SIGNA EXCITE	SE	2D	5	666.664	17	0	63.86302	1.5	7.5	90	1.11334	20	256	192	0.9375\0.9375	TCGA-HT-7692/1.3.6.1.4.1.14519.5.2.1.2531.4003.211398814274191438581429613406/1.3.6.1.4.1.14519.5.2.1.2531.4003.957195987351897640611427265940
TCGA-LGG	TCGA-HT	TCGA-HT-7692	t2	M	43	70.307	AX_FSE_T2_inter	8	1.49904E+15	7/24/1996	GE MEDICAL SYSTEMS		SIGNA EXCITE	SE	2D	2	4766.66	93.248	0	63.86302	1.5	2	90	1.8934	84	256	160	1.0156\1.0156	TCGA-HT-7692/1.3.6.1.4.1.14519.5.2.1.2531.4003.211398814274191438581429613406/1.3.6.1.4.1.14519.5.2.1.2531.4003.326142621012177190108169467120
TCGA-LGG	TCGA-HT	TCGA-HT-7693	flair	F	51	83.915	Ax_Flair_irFSE_H	7	2.56082E+15	5/20/1995	GE MEDICAL SYSTEMS		SIGNA EXCITE	IR	2D	5	9002	121.6	2200	63.86308	1.5	5	90	1.0496	32	256	224	0.9375\0.9375	TCGA-HT-7693/1.3.6.1.4.1.14519.5.2.1.2531.4003.628801887079472592322253871036/1.3.6.1.4.1.14519.5.2.1.2531.4003.216268246131326829920251118767
TCGA-LGG	TCGA-HT	TCGA-HT-7693	t1Gd	F	51	83.915	C+AX_3D_SPGR	11	2.56082E+15	5/20/1995	GE MEDICAL SYSTEMS		SIGNA EXCITE	GR	3D	2	9.336	4.472	450	63.86308	1.5	2	20	0.3336	106	320	192	0.5078\0.5078	TCGA-HT-7693/1.3.6.1.4.1.14519.5.2.1.2531.4003.628801887079472592322253871036/1.3.6.1.4.1.14519.5.2.1.2531.4003.748980782452317231270307024306
TCGA-LGG	TCGA-HT	TCGA-HT-7693	t1	F	51	83.915	AX_T1	5	2.56082E+15	5/20/1995	GE MEDICAL SYSTEMS		SIGNA EXCITE	SE	2D	5	683.332	17	0	63.86308	1.5	7.5	90	1.2415	21	256	192	0.9375\0.9375	TCGA-HT-7693/1.3.6.1.4.1.14519.5.2.1.2531.4003.628801887079472592322253871036/1.3.6.1.4.1.14519.5.2.1.2531.4003.322960220935957352201340032031
TCGA-LGG	TCGA-HT	TCGA-HT-7693	t2	F	51	83.915	3-pl_T2*_FGRE_S	1	2.56082E+15	5/20/1995	GE MEDICAL SYSTEMS		SIGNA EXCITE	RM	2D	5	5.104	1.46	0	63.8631	1.5	10	30	0.267671	9	256	128	0.9375\0.9375	TCGA-HT-7693/1.3.6.1.4.1.14519.5.2.1.2531.4003.628801887079472592322253871036/1.3.6.1.4.1.14519.5.2.1.2531.4003.289315312435476678143013547067
TCGA-LGG	TCGA-HT	TCGA-HT-7694	flair	M	60	107	Ax_Flair_irFSE_H	7	2.73244E+15	4/4/1995	GE MEDICAL SYSTEMS		SIGNA EXCITE	IR	2D	5	9002	121.6	2200	63.86312	1.5	5	90	1.1795	32	256	224	0.9375\0.9375	TCGA-HT-7694/1.3.6.1.4.1.14519.5.2.1.2531.4003.287964934328207007202059293153/1.3.6.1.4.1.14519.5.2.1.2531.4003.131371499353948533109942982301
TCGA-LGG	TCGA-HT	TCGA-HT-7694	t1Gd	M	60	107	C+AX_3D_SPGR	13	2.73244E+15	4/4/1995	GE MEDICAL SYSTEMS		SIGNA EXCITE	GR	3D	2	9.128	4.468	450	63.86312	1.5	2	20	0.356997	106	320	192	0.5078\0.5078	TCGA-HT-7694/1.3.6.1.4.1.14519.5.2.1.2531.4003.287964934328207007202059293153/1.3.6.1.4.1.14519.5.2.1.2531.4003.125678417374893795437733747898
TCGA-LGG	TCGA-HT	TCGA-HT-7694	t1	M	60	107	AX_T1	5	2.73244E+15	4/4/1995	GE MEDICAL SYSTEMS		SIGNA EXCITE	SE	2D	5	683.332	17	0	63.86312	1.5	7.5	90	1.19617	21	256	192	1.0156\1.0156	TCGA-HT-7694/1.3.6.1.4.1.14519.5.2.1.2531.4003.287964934328207007202059293153/1.3.6.1.4.1.14519.5.2.1.2531.4003.326296038155567068613921506545
TCGA-LGG	TCGA-HT	TCGA-HT-7694	t2	M	60	107	COR_GRE_T2	8	2.73244E+15	4/4/1995	GE MEDICAL SYSTEMS		SIGNA EXCITE	GR	2D	5	550	35	0	63.86312	1.5	7.5	20	0.00666331	48	256	192	1.0156\1.0156	TCGA-HT-7694/1.3.6.1.4.1.14519.5.2.1.2531.4003.287964934328207007202059293153/1.3.6.1.4.1.14519.5.2.1.2531.4003.866906654890810756461307550000
TCGA-LGG	TCGA-HT	TCGA-HT-7855	flair	M	39	94.347	Ax_T2_Flair	5	2.90984E+15	10/20/1995	GE MEDICAL SYSTEMS		SIGNA EXCITE	SE\IR	2D	5	9002	152.964	2250	127.7268	3	5.5	90	0.6902	28	512	192	0.4688\0.4688	TCGA-HT-7855/1.3.6.1.4.1.14519.5.2.1.2531.4003.198324244153534584390623424581/1.3.6.1.4.1.14519.5.2.1.2531.4003.326558553266212493966722036356
TCGA-LGG	TCGA-HT	TCGA-HT-7855	t1Gd	M	39	94.347	Ax_T1_MP_SPGR+C	10	2.90984E+15	10/20/1995	GE MEDICAL SYSTEMS		SIGNA EXCITE	GR	3D	2	7.76	3.08	300	127.7268	3	2	13	0.643089	106	320	224	0.5078\0.5078	TCGA-HT-7855/1.3.6.1.4.1.14519.5.2.1.2531.4003.198324244153534584390623424581/1.3.6.1.4.1.14519.5.2.1.2531.4003.181614742987205173574912986643
TCGA-LGG	TCGA-HT	TCGA-HT-7855	t1	M	39	94.347	Ax_T1_SE	7	2.90984E+15	10/20/1995	GE MEDICAL SYSTEMS		SIGNA EXCITE	SE	2D	5	550	17	0	127.7268	3	7.5	90	2.02031	21	512	256	0.4688\0.4688	TCGA-HT-7855/1.3.6.1.4.1.14519.5.2.1.2531.4003.198324244153534584390623424581/1.3.6.1.4.1.14519.5.2.1.2531.4003.199095925660425709421413760410
TCGA-LGG	TCGA-HT	TCGA-HT-7855	t2	M	39	94.347	Cor_2D_T2*GRE	9	2.90984E+15	10/20/1995	GE MEDICAL SYSTEMS		SIGNA EXCITE	GR	2D	5	600	10	0	127.7268	3	7.5	25	0.0315047	44	512	192	0.4688\0.4688	TCGA-HT-7855/1.3.6.1.4.1.14519.5.2.1.2531.4003.198324244153534584390623424581/1.3.6.1.4.1.14519.5.2.1.2531.4003.350021473582796468994279160988
TCGA-LGG	TCGA-HT	TCGA-HT-7856	flair	M	35	92.079	Ax_Flair_irFSE_H	6	2.8409E+15	8/31/1995	GE MEDICAL SYSTEMS		SIGNA EXCITE	SE\IR	2D	5	9002	121.752	2200	63.86313	1.5	5	90	1.0974	32	256	224	0.9375\0.9375	TCGA-HT-7856/1.3.6.1.4.1.14519.5.2.1.2531.4003.100877690152591433926158971037/1.3.6.1.4.1.14519.5.2.1.2531.4003.335140292213978326526348108103
TCGA-LGG	TCGA-HT	TCGA-HT-7856	t1Gd	M	35	92.079	COR_T1+C	11	2.8409E+15	8/31/1995	GE MEDICAL SYSTEMS		SIGNA EXCITE	SE	2D	5	416.664	17	0	63.86313	1.5	7.5	90	1.21878	24	256	192	0.9375\0.9375	TCGA-HT-7856/1.3.6.1.4.1.14519.5.2.1.2531.4003.100877690152591433926158971037/1.3.6.1.4.1.14519.5.2.1.2531.4003.881801191410038591436680403595
TCGA-LGG	TCGA-HT	TCGA-HT-7856	t1	M	35	92.079	SAG_T1	4	2.8409E+15	8/31/1995	GE MEDICAL SYSTEMS		SIGNA EXCITE	SE	2D	7	566.664	17	0	63.86313	1.5	9	90	1.26795	17	256	192	0.9375\0.9375	TCGA-HT-7856/1.3.6.1.4.1.14519.5.2.1.2531.4003.100877690152591433926158971037/1.3.6.1.4.1.14519.5.2.1.2531.4003.882113785187560242328167389560
TCGA-LGG	TCGA-HT	TCGA-HT-7856	t2	M	35	92.079	COR_GRE_T2	7	2.8409E+15	8/31/1995	GE MEDICAL SYSTEMS		SIGNA EXCITE	GR	2D	5	500	35	0	63.86313	1.5	7.5	20	0.0045474	48	256	192	0.9375\0.9375	TCGA-HT-7856/1.3.6.1.4.1.14519.5.2.1.2531.4003.100877690152591433926158971037/1.3.6.1.4.1.14519.5.2.1.2531.4003.298451687529064117671386672478
TCGA-LGG	TCGA-HT	TCGA-HT-7860	flair	F	60	68	Ax_Flair_irFSE_H	7	1.97569E+15	5/13/1996	GE MEDICAL SYSTEMS		SIGNA EXCITE	SE\IR	2D	5	9002	121.524	2200	63.86304	1.5	5	90	0.9488	32	256	224	0.9375\0.9375	TCGA-HT-7860/1.3.6.1.4.1.14519.5.2.1.2531.4003.290722495921577895242204914721/1.3.6.1.4.1.14519.5.2.1.2531.4003.301059638139812997612342918747
TCGA-LGG	TCGA-HT	TCGA-HT-7860	t1Gd	F	60	68	C+AX_3D_SPGR	12	1.97569E+15	5/13/1996	GE MEDICAL SYSTEMS		SIGNA EXCITE	GR	3D	2	9.344	4.476	450	63.86304	1.5	2	20	0.29521	106	320	192	0.5078\0.5078	TCGA-HT-7860/1.3.6.1.4.1.14519.5.2.1.2531.4003.290722495921577895242204914721/1.3.6.1.4.1.14519.5.2.1.2531.4003.100799255779837914036141600878
TCGA-LGG	TCGA-HT	TCGA-HT-7860	t1	F	60	68	AX_T1	5	1.97569E+15	5/13/1996	GE MEDICAL SYSTEMS		SIGNA EXCITE	SE	2D	5	716.664	17	0	63.86304	1.5	7.5	90	1.12092	22	256	192	0.9375\0.9375	TCGA-HT-7860/1.3.6.1.4.1.14519.5.2.1.2531.4003.290722495921577895242204914721/1.3.6.1.4.1.14519.5.2.1.2531.4003.255671469725080757792140904566
TCGA-LGG	TCGA-HT	TCGA-HT-7860	t2	F	60	68	Prop_T2_TRF	6	1.97569E+15	5/13/1996	GE MEDICAL SYSTEMS		SIGNA EXCITE	SE	2D	5	5200	113.256		63.86303	1.5	7.5	90	1.2053772	22	384	384	0.468750\0.468750	TCGA-HT-7860/1.3.6.1.4.1.14519.5.2.1.2531.4003.290722495921577895242204914721/1.3.6.1.4.1.14519.5.2.1.2531.4003.228588646024368852629824081803
TCGA-LGG	TCGA-HT	TCGA-HT-7874	flair	F	47	62	Ax_Flair_irFSE_H	6	2.64153E+15	9/2/1995	GE MEDICAL SYSTEMS		SIGNA EXCITE	SE\IR	2D	5	9002	121.6	2200	63.86313	1.5	5	90	0.9077	30	256	224	0.9375\0.9375	TCGA-HT-7874/1.3.6.1.4.1.14519.5.2.1.2531.4003.253968930679883438764511461816/1.3.6.1.4.1.14519.5.2.1.2531.4003.114059255086495939980670300373
TCGA-LGG	TCGA-HT	TCGA-HT-7874	t1Gd	F	47	62	AX_3D_SPGR	11	2.64153E+15	9/2/1995	GE MEDICAL SYSTEMS		SIGNA EXCITE	GR	3D	2	9.42	4.536	450	63.86313	1.5	2	20	0.287115	106	320	192	0.4688\0.4688	TCGA-HT-7874/1.3.6.1.4.1.14519.5.2.1.2531.4003.253968930679883438764511461816/1.3.6.1.4.1.14519.5.2.1.2531.4003.271081801017486100580794738668
TCGA-LGG	TCGA-HT	TCGA-HT-7874	t1	F	47	62	AX_T1	5	2.64153E+15	9/2/1995	GE MEDICAL SYSTEMS		SIGNA EXCITE	SE	2D	5	666.664	17	0	63.86313	1.5	7.5	90	1.04814	20	256	192	0.9375\0.9375	TCGA-HT-7874/1.3.6.1.4.1.14519.5.2.1.2531.4003.253968930679883438764511461816/1.3.6.1.4.1.14519.5.2.1.2531.4003.172208561596137136210902304486
TCGA-LGG	TCGA-HT	TCGA-HT-7874	t2	F	47	62	3-pl_T2*_FGRE_S	1	2.64153E+15	9/2/1995	GE MEDICAL SYSTEMS		SIGNA EXCITE	RM	2D	5	5.104	1.46	0	63.86308	1.5	10	30	0.231476	9	256	128	0.9375\0.9375	TCGA-HT-7874/1.3.6.1.4.1.14519.5.2.1.2531.4003.253968930679883438764511461816/1.3.6.1.4.1.14519.5.2.1.2531.4003.108904271616553624903958841695
TCGA-LGG	TCGA-HT	TCGA-HT-7879	flair	M	31	74.843	Ax_T2_Flair	5	1.40238E+15	10/9/1998	GE MEDICAL SYSTEMS		SIGNA EXCITE	SE\IR	2D	5	9002	150.528	2250	127.7265	3	5	90	0.7351	28	512	192	0.4688\0.4688	TCGA-HT-7879/1.3.6.1.4.1.14519.5.2.1.2531.4003.208179366605438767398250387116/1.3.6.1.4.1.14519.5.2.1.2531.4003.390124297613614320003204742124
TCGA-LGG	TCGA-HT	TCGA-HT-7879	t1Gd	M	31	74.843	POST_Ax_T1_MP_SPGR	12	1.40238E+15	10/9/1998	GE MEDICAL SYSTEMS		SIGNA EXCITE	GR	3D	2	6.773	2.772	0	127.7265	3	1	13	0.473884	212	320	192	0.5078\0.5078	TCGA-HT-7879/1.3.6.1.4.1.14519.5.2.1.2531.4003.208179366605438767398250387116/1.3.6.1.4.1.14519.5.2.1.2531.4003.287469028595190429124363364353
TCGA-LGG	TCGA-HT	TCGA-HT-7879	t1	M	31	74.843	Ax_T1_MP_SPGR	6	1.40238E+15	10/9/1998	GE MEDICAL SYSTEMS		SIGNA EXCITE	GR	3D	2	6.773	2.772	0	127.7265	3	1	13	0.473884	212	320	192	0.5078\0.5078	TCGA-HT-7879/1.3.6.1.4.1.14519.5.2.1.2531.4003.208179366605438767398250387116/1.3.6.1.4.1.14519.5.2.1.2531.4003.218683428988186850780269340361
TCGA-LGG	TCGA-HT	TCGA-HT-7879	t2	M	31	74.843	(OPTIONAL)Ax_T2_FSE_INTER	7	1.40238E+15	10/9/1998	GE MEDICAL SYSTEMS		SIGNA EXCITE	SE	2D	2	4200	82.752	0	127.7265	3	2	90	1.6163	78	320	192	0.5078\0.5078	TCGA-HT-7879/1.3.6.1.4.1.14519.5.2.1.2531.4003.208179366605438767398250387116/1.3.6.1.4.1.14519.5.2.1.2531.4003.723937203870259397937020184430
TCGA-LGG	TCGA-HT	TCGA-HT-7882	flair	M	66	67.132	Ax_Flair_irFSE_H	5	2.48389E+15	1/25/1997	GE MEDICAL SYSTEMS		SIGNA EXCITE	SE\IR	2D	5	9002	121.752	2200	63.863	1.5	5	90	0.943	32	256	224	0.9375\0.9375	TCGA-HT-7882/1.3.6.1.4.1.14519.5.2.1.2531.4003.553374666251335749299587381378/1.3.6.1.4.1.14519.5.2.1.2531.4003.111610015608806980339206664631
TCGA-LGG	TCGA-HT	TCGA-HT-7882	t1Gd	M	66	67.132	POSTCOR_T1_SE	14	2.48389E+15	1/25/1997	GE MEDICAL SYSTEMS		SIGNA EXCITE	SE	2D	5	500	16	0	63.863	1.5	5	90	1.12453	37	256	160	0.9375\0.9375	TCGA-HT-7882/1.3.6.1.4.1.14519.5.2.1.2531.4003.553374666251335749299587381378/1.3.6.1.4.1.14519.5.2.1.2531.4003.143978018567514063128225625202
TCGA-LGG	TCGA-HT	TCGA-HT-7882	t1	M	66	67.132	REPEAT_AX_3D_SPGR	9	2.48389E+15	1/25/1997	GE MEDICAL SYSTEMS		SIGNA EXCITE	GR	3D	2	9.344	4.476	450	63.863	1.5	2	20	0.293396	106	320	192	0.5078\0.5078	TCGA-HT-7882/1.3.6.1.4.1.14519.5.2.1.2531.4003.553374666251335749299587381378/1.3.6.1.4.1.14519.5.2.1.2531.4003.131861920354865041057455029355
TCGA-LGG	TCGA-HT	TCGA-HT-7882	t2	M	66	67.132	COR_GRE_T2	6	2.48389E+15	1/25/1997	GE MEDICAL SYSTEMS		SIGNA EXCITE	GR	2D	5	567	35	0	63.863	1.5	7.5	20	0.00559802	50	256	192	0.9375\0.9375	TCGA-HT-7882/1.3.6.1.4.1.14519.5.2.1.2531.4003.553374666251335749299587381378/1.3.6.1.4.1.14519.5.2.1.2531.4003.110745866062969069187735984363
TCGA-LGG	TCGA-HT	TCGA-HT-7884	flair	F	44	68.039	Ax_Flair_irFSE_H	7	3.32409E+15	9/13/1998	GE MEDICAL SYSTEMS		SIGNA EXCITE	SE\IR	2D	5	9002	131.328	2200	63.8609	1.5	5	90	0.8304	28	288	224	0.4688\0.4688	TCGA-HT-7884/1.3.6.1.4.1.14519.5.2.1.2531.4003.213534942777481053514809821174/1.3.6.1.4.1.14519.5.2.1.2531.4003.321207014837740778483316363283
TCGA-LGG	TCGA-HT	TCGA-HT-7884	t1Gd	F	44	68.039	AX_3D_SPGR+C	11	3.32409E+15	9/13/1998	GE MEDICAL SYSTEMS		SIGNA EXCITE	GR	3D	2	9.344	4.476	450	63.8609	1.5	1	20	0.295292	212	320	192	0.5078\0.5078	TCGA-HT-7884/1.3.6.1.4.1.14519.5.2.1.2531.4003.213534942777481053514809821174/1.3.6.1.4.1.14519.5.2.1.2531.4003.318439416511462348230053052662
TCGA-LGG	TCGA-HT	TCGA-HT-7884	t1	F	44	68.039	AX_T1	5	3.32409E+15	9/13/1998	GE MEDICAL SYSTEMS		SIGNA EXCITE	SE	2D	5	666.664	17	0	63.8609	1.5	7	90	1.09596	20	288	192	0.4688\0.4688	TCGA-HT-7884/1.3.6.1.4.1.14519.5.2.1.2531.4003.213534942777481053514809821174/1.3.6.1.4.1.14519.5.2.1.2531.4003.207800235363459841669208898660
TCGA-LGG	TCGA-HT	TCGA-HT-7884	t2	F	44	68.039	(OPT)_AX_T2_FSE_inter	12	3.32409E+15	9/13/1998	GE MEDICAL SYSTEMS		SIGNA EXCITE	SE	2D	2	4550	93.248	0	63.8609	1.5	2	90	1.8596	80	256	160	1.0156\1.0156	TCGA-HT-7884/1.3.6.1.4.1.14519.5.2.1.2531.4003.213534942777481053514809821174/1.3.6.1.4.1.14519.5.2.1.2531.4003.333883177788958445968782847214
TCGA-LGG	TCGA-HT	TCGA-HT-8018	flair	F	40	92.986	Ax_Flair_irFSE_H	6	1.76778E+15	4/11/1997	GE MEDICAL SYSTEMS		SIGNA EXCITE	SE\IR	2D	5	9002	126.588	2200	63.86295	1.5	5	90	0.0531	32	256	224	0.9375\0.9375	TCGA-HT-8018/1.3.6.1.4.1.14519.5.2.1.2531.4003.112684169989572671934693953563/1.3.6.1.4.1.14519.5.2.1.2531.4003.750153295669091838671413843337
TCGA-LGG	TCGA-HT	TCGA-HT-8018	t1Gd	F	40	92.986	AX_3D_SPGR_+_C	9	1.76778E+15	4/11/1997	GE MEDICAL SYSTEMS		SIGNA EXCITE	GR	3D	2	9.336	4.472	450	63.86295	1.5	2	20	0.0118572	106	320	192	0.5078\0.5078	TCGA-HT-8018/1.3.6.1.4.1.14519.5.2.1.2531.4003.112684169989572671934693953563/1.3.6.1.4.1.14519.5.2.1.2531.4003.314673445079965605329585246765
TCGA-LGG	TCGA-HT	TCGA-HT-8018	t1	F	40	92.986	AX_T1	4	1.76778E+15	4/11/1997	GE MEDICAL SYSTEMS		SIGNA EXCITE	SE	2D	5	666.664	17	0	63.86295	1.5	7.5	90	0.0430794	20	256	192	0.9375\0.9375	TCGA-HT-8018/1.3.6.1.4.1.14519.5.2.1.2531.4003.112684169989572671934693953563/1.3.6.1.4.1.14519.5.2.1.2531.4003.626802034424465313475732328979
TCGA-LGG	TCGA-HT	TCGA-HT-8018	t2	F	40	92.986	(OPTIONAL)_AX_FSE_T2_inter	5	1.76778E+15	4/11/1997	GE MEDICAL SYSTEMS		SIGNA EXCITE	SE	2D	2	4100	87.424	0	63.86295	1.5	2	90	0.1135	84	256	160	1.0156\1.0156	TCGA-HT-8018/1.3.6.1.4.1.14519.5.2.1.2531.4003.112684169989572671934693953563/1.3.6.1.4.1.14519.5.2.1.2531.4003.757368868058675693748842299676
TCGA-LGG	TCGA-HT	TCGA-HT-8105	flair	M	54	149.685	Ax_Flair_irFSE_H	6	1.40311E+15	8/26/1998	GE MEDICAL SYSTEMS		SIGNA EXCITE	SE\IR	2D	5	9002	120.156	2200	63.86083	1.5	5	90	1.1596	32	288	224	0.4688\0.4688	TCGA-HT-8105/1.3.6.1.4.1.14519.5.2.1.2531.4003.666089736017948642080519950202/1.3.6.1.4.1.14519.5.2.1.2531.4003.521767595065028541607661249239
TCGA-LGG	TCGA-HT	TCGA-HT-8105	t1Gd	M	54	149.685	=+C_AX_3D_SPGR	10	1.40311E+15	8/26/1998	GE MEDICAL SYSTEMS		SIGNA EXCITE	GR	3D	2	9.456	4.504	450	63.86083	1.5	1	20	0.332902	212	320	192	0.5078\0.5078	TCGA-HT-8105/1.3.6.1.4.1.14519.5.2.1.2531.4003.666089736017948642080519950202/1.3.6.1.4.1.14519.5.2.1.2531.4003.261424643169894361999089010789
TCGA-LGG	TCGA-HT	TCGA-HT-8105	t1	M	54	149.685	SAG_T1	4	1.40311E+15	8/26/1998	GE MEDICAL SYSTEMS		SIGNA EXCITE	SE	2D	4	800	17	0	63.86083	1.5	6	90	1.37125	24	256	192	0.9375\0.9375	TCGA-HT-8105/1.3.6.1.4.1.14519.5.2.1.2531.4003.666089736017948642080519950202/1.3.6.1.4.1.14519.5.2.1.2531.4003.359499344977834153037880376188
TCGA-LGG	TCGA-HT	TCGA-HT-8105	t2	M	54	149.685	COR_GRE_T2	7	1.40311E+15	8/26/1998	GE MEDICAL SYSTEMS		SIGNA EXCITE	GR	2D	5	517	15	0	63.86083	1.5	7.5	20	0.00763495	44	288	192	0.4688\0.4688	TCGA-HT-8105/1.3.6.1.4.1.14519.5.2.1.2531.4003.666089736017948642080519950202/1.3.6.1.4.1.14519.5.2.1.2531.4003.290098899120495776459456484100
TCGA-LGG	TCGA-HT	TCGA-HT-8106	flair	M	53	79.379	Ax_Flair_irFSE_H	6	3.00856E+15	7/27/1997	GE MEDICAL SYSTEMS		SIGNA EXCITE	SE\IR	2D	5	9002	121.6	2200	63.86295	1.5	5	90	1.022	32	256	224	0.9375\0.9375	TCGA-HT-8106/1.3.6.1.4.1.14519.5.2.1.2531.4003.193992540899316552400927467522/1.3.6.1.4.1.14519.5.2.1.2531.4003.223047379880676549838224939528
TCGA-LGG	TCGA-HT	TCGA-HT-8106	t1Gd	M	53	79.379	C+AX_3D_SPGR	12	3.00856E+15	7/27/1997	GE MEDICAL SYSTEMS		SIGNA EXCITE	GR	3D	2	9.36	4.48	450	63.86295	1.5	1	20	0.305234	212	320	192	0.5078\0.5078	TCGA-HT-8106/1.3.6.1.4.1.14519.5.2.1.2531.4003.193992540899316552400927467522/1.3.6.1.4.1.14519.5.2.1.2531.4003.208453668054401410366698146928
TCGA-LGG	TCGA-HT	TCGA-HT-8106	t1	M	53	79.379	AX_T1	5	3.00856E+15	7/27/1997	GE MEDICAL SYSTEMS		SIGNA EXCITE	SE	2D	5	716.664	17	0	63.86295	1.5	7.5	90	1.20734	22	256	192	0.9375\0.9375	TCGA-HT-8106/1.3.6.1.4.1.14519.5.2.1.2531.4003.193992540899316552400927467522/1.3.6.1.4.1.14519.5.2.1.2531.4003.227475575209664270665791110082
TCGA-LGG	TCGA-HT	TCGA-HT-8106	t2	M	53	79.379	Prop_T2_TRF	8	3.00856E+15	7/27/1997	GE MEDICAL SYSTEMS		SIGNA EXCITE	SE	2D	5	5300	112.736		63.86295	1.5	7.5	90	1.273803	22	384	384	0.468750\0.468750	TCGA-HT-8106/1.3.6.1.4.1.14519.5.2.1.2531.4003.193992540899316552400927467522/1.3.6.1.4.1.14519.5.2.1.2531.4003.268245415182347752143774434147
TCGA-LGG	TCGA-HT	TCGA-HT-8107	flair	M	62	86	Ax_T2_Flair	7	2.31066E+15	7/8/1998	GE MEDICAL SYSTEMS		SIGNA EXCITE	SE\IR	2D	5	9002	150.864	2250	127.7266	3	5	90	0.7109	28	512	192	0.4688\0.4688	TCGA-HT-8107/1.3.6.1.4.1.14519.5.2.1.2531.4003.126549621125671515633918730177/1.3.6.1.4.1.14519.5.2.1.2531.4003.742126079765319223078547725684
TCGA-LGG	TCGA-HT	TCGA-HT-8107	t1Gd	M	62	86	Ax_T1_MP_SPGR_+C	13	2.31066E+15	7/8/1998	GE MEDICAL SYSTEMS		SIGNA EXCITE	GR	3D	2	6.773	2.772	0	127.7266	3	1	13	0.484134	212	320	192	0.5078\0.5078	TCGA-HT-8107/1.3.6.1.4.1.14519.5.2.1.2531.4003.126549621125671515633918730177/1.3.6.1.4.1.14519.5.2.1.2531.4003.140725228801837632444136864481
TCGA-LGG	TCGA-HT	TCGA-HT-8107	t1	M	62	86	Ax_T1_SE	9	2.31066E+15	7/8/1998	GE MEDICAL SYSTEMS		SIGNA EXCITE	SE	2D	5	550	18	0	127.7266	3	7.5	90	2.06216	20	320	224	0.4688\0.4688	TCGA-HT-8107/1.3.6.1.4.1.14519.5.2.1.2531.4003.126549621125671515633918730177/1.3.6.1.4.1.14519.5.2.1.2531.4003.339572109113075228569870448997
TCGA-LGG	TCGA-HT	TCGA-HT-8107	t2	M	62	86	(OPTIONAL)Ax_T2_FSE_INTER	6	2.31066E+15	7/8/1998	GE MEDICAL SYSTEMS		SIGNA EXCITE	SE	2D	2	4500	82.752	0	127.7266	3	2	90	1.6202	82	320	192	0.5078\0.5078	TCGA-HT-8107/1.3.6.1.4.1.14519.5.2.1.2531.4003.126549621125671515633918730177/1.3.6.1.4.1.14519.5.2.1.2531.4003.175934860067825647876040050083
TCGA-LGG	TCGA-HT	TCGA-HT-8111	flair	M	32	89.811	Ax_Flair_irFSE_H	6	1.48464E+15	3/30/1998	GE MEDICAL SYSTEMS		SIGNA EXCITE	SE\IR	2D	5	9002	131.48	2200	63.8609	1.5	5	90	0.9488	28	288	224	0.4688\0.4688	TCGA-HT-8111/1.3.6.1.4.1.14519.5.2.1.2531.4003.242435921414571703883231168651/1.3.6.1.4.1.14519.5.2.1.2531.4003.207964003557221916122969204644
TCGA-LGG	TCGA-HT	TCGA-HT-8111	t1Gd	M	32	89.811	AX_3D_SPGR+C	11	1.48464E+15	3/30/1998	GE MEDICAL SYSTEMS		SIGNA EXCITE	GR	3D	2	9.364	4.48	450	63.8609	1.5	1	20	0.31767	212	320	192	0.5078\0.5078	TCGA-HT-8111/1.3.6.1.4.1.14519.5.2.1.2531.4003.242435921414571703883231168651/1.3.6.1.4.1.14519.5.2.1.2531.4003.320743579510829717547301347886
TCGA-LGG	TCGA-HT	TCGA-HT-8111	t1	M	32	89.811	AX_T1	5	1.48464E+15	3/30/1998	GE MEDICAL SYSTEMS		SIGNA EXCITE	SE	2D	5	750	17	0	63.8609	1.5	7	90	1.22394	22	288	192	0.4688\0.4688	TCGA-HT-8111/1.3.6.1.4.1.14519.5.2.1.2531.4003.242435921414571703883231168651/1.3.6.1.4.1.14519.5.2.1.2531.4003.908347256312619710522332501164
TCGA-LGG	TCGA-HT	TCGA-HT-8111	t2	M	32	89.811	3-pl_T2*_FGRE_S	1	1.48464E+15	3/30/1998	GE MEDICAL SYSTEMS		SIGNA EXCITE	RM	2D	5	5.104	1.46	0	63.86089	1.5	10	30	0.276539	15	256	128	0.9375\0.9375	TCGA-HT-8111/1.3.6.1.4.1.14519.5.2.1.2531.4003.242435921414571703883231168651/1.3.6.1.4.1.14519.5.2.1.2531.4003.269592914235352345887545312963
TCGA-LGG	TCGA-HT	TCGA-HT-8113	flair	F	49	79.379	Ax_Flair_irFSE_H	5	1.58118E+15	8/9/1993	GE MEDICAL SYSTEMS		SIGNA EXCITE	IR	2D	5	9002	126.252	2200	63.86342	1.5	5	90	0.0544	32	256	224	0.937513\0.937488	TCGA-HT-8113/1.3.6.1.4.1.14519.5.2.1.2531.4003.119195295177370323580160059884/1.3.6.1.4.1.14519.5.2.1.2531.4003.875941858876296194253422981944
TCGA-LGG	TCGA-HT	TCGA-HT-8113	t1Gd	F	49	79.379	AX_3D_SPGR	9	1.58118E+15	8/9/1993	GE MEDICAL SYSTEMS		SIGNA EXCITE	GR	3D	2	23	4.6	0	63.86342	1.5	2	26	0.0162443	86	256	160	1.01562\1.01562	TCGA-HT-8113/1.3.6.1.4.1.14519.5.2.1.2531.4003.119195295177370323580160059884/1.3.6.1.4.1.14519.5.2.1.2531.4003.107596026517862198471059681510
TCGA-LGG	TCGA-HT	TCGA-HT-8113	t1	F	49	79.379	AX_T1	3	1.58118E+15	8/9/1993	GE MEDICAL SYSTEMS		SIGNA EXCITE	SE	2D	5	683.332	17	0	63.86342	1.5	7.5	90	0.0452583	21	256	192	0.937488\0.937495	TCGA-HT-8113/1.3.6.1.4.1.14519.5.2.1.2531.4003.119195295177370323580160059884/1.3.6.1.4.1.14519.5.2.1.2531.4003.255471461929192148244064707039
TCGA-LGG	TCGA-HT	TCGA-HT-8113	t2	F	49	79.379	COR_T2__FSE	7	1.58118E+15	8/9/1993	GE MEDICAL SYSTEMS		SIGNA EXCITE	SE	2D	3	3850	82.56	0	63.86341	1.5	3.5	90	0.1288	22	320	256	0.351561\0.351562	TCGA-HT-8113/1.3.6.1.4.1.14519.5.2.1.2531.4003.119195295177370323580160059884/1.3.6.1.4.1.14519.5.2.1.2531.4003.213902046445134007901271843900
TCGA-LGG	TCGA-HT	TCGA-HT-8114	flair	M	36	77.111	Ax_Flair_irFSE_H	7	3.2038E+15	10/30/1998	GE MEDICAL SYSTEMS		SIGNA EXCITE	SE\IR	2D	5	9002	127.176	2200	63.86084	1.5	5	90	1.1059	32	288	224	0.4688\0.4688	TCGA-HT-8114/1.3.6.1.4.1.14519.5.2.1.2531.4003.558276508192500356115090222947/1.3.6.1.4.1.14519.5.2.1.2531.4003.108490552093857556325409386866
TCGA-LGG	TCGA-HT	TCGA-HT-8114	t1Gd	M	36	77.111	AX_3D_SPGR	11	3.2038E+15	10/30/1998	GE MEDICAL SYSTEMS		SIGNA EXCITE	GR	3D	2	9.352	4.476	450	63.86084	1.5	1	20	0.30714	212	320	192	0.5078\0.5078	TCGA-HT-8114/1.3.6.1.4.1.14519.5.2.1.2531.4003.558276508192500356115090222947/1.3.6.1.4.1.14519.5.2.1.2531.4003.342300001999257561480858599436
TCGA-LGG	TCGA-HT	TCGA-HT-8114	t2	M	36	77.111	3-pl_T2*_FGRE_S	6	3.2038E+15	10/30/1998	GE MEDICAL SYSTEMS		SIGNA EXCITE	RM	2D	5	5.104	1.46	0	63.86081	1.5	10	30	0.257024	15	256	128	0.9375\0.9375	TCGA-HT-8114/1.3.6.1.4.1.14519.5.2.1.2531.4003.558276508192500356115090222947/1.3.6.1.4.1.14519.5.2.1.2531.4003.262039158313498745895460022053
TCGA-LGG	TCGA-HT	TCGA-HT-8563	flair	F	30	77.111	Ax_Flair_irFSE_H	6	4.02868E+15	12/9/1998	GE MEDICAL SYSTEMS		SIGNA EXCITE	SE\IR	2D	5	9002	126.924	2200	63.86088	1.5	5	90	1.1059	32	288	224	0.4688\0.4688	TCGA-HT-8563/1.3.6.1.4.1.14519.5.2.1.2531.4003.104336479544128599145573158636/1.3.6.1.4.1.14519.5.2.1.2531.4003.147276557455303984775492047930
TCGA-LGG	TCGA-HT	TCGA-HT-8563	t1Gd	F	30	77.111	AX_3D_SPGR	10	4.02868E+15	12/9/1998	GE MEDICAL SYSTEMS		SIGNA EXCITE	GR	3D	2	9.352	4.476	450	63.86088	1.5	1	20	0.30714	212	320	192	0.5078\0.5078	TCGA-HT-8563/1.3.6.1.4.1.14519.5.2.1.2531.4003.104336479544128599145573158636/1.3.6.1.4.1.14519.5.2.1.2531.4003.315705360410181524285206535180
TCGA-LGG	TCGA-HT	TCGA-HT-8563	t1	F	30	77.111	AX_T1	5	4.02868E+15	12/9/1998	GE MEDICAL SYSTEMS		SIGNA EXCITE	SE	2D	5	766.664	17	0	63.86088	1.5	7	90	1.16336	23	288	192	0.4688\0.4688	TCGA-HT-8563/1.3.6.1.4.1.14519.5.2.1.2531.4003.104336479544128599145573158636/1.3.6.1.4.1.14519.5.2.1.2531.4003.146200342358276156961672468450
TCGA-LGG	TCGA-HT	TCGA-HT-8563	t2	F	30	77.111	3-pl_T2*_FGRE_S	1	4.02868E+15	12/9/1998	GE MEDICAL SYSTEMS		SIGNA EXCITE	RM	2D	5	5.104	1.46	0	63.86082	1.5	10	30	0.257024	15	256	128	0.9375\0.9375	TCGA-HT-8563/1.3.6.1.4.1.14519.5.2.1.2531.4003.104336479544128599145573158636/1.3.6.1.4.1.14519.5.2.1.2531.4003.491822277939696706830446210807
TCGA-LGG	TCGA-HT	TCGA-HT-A5RC	flair	F	70	74.843	Ax_Flair_irFSE_H	5	1.89125E+15	8/31/1999	GE MEDICAL SYSTEMS		Signa HDxt	SE\IR	2D	5	9002	127.176	2200	63.86087	1.5	5	90	1.0902	32	288	224	0.4688\0.4688	TCGA-HT-A5RC/1.3.6.1.4.1.14519.5.2.1.2531.4003.231610681115360230926274787101/1.3.6.1.4.1.14519.5.2.1.2531.4003.106462291730893974080562229741
TCGA-LGG	TCGA-HT	TCGA-HT-A5RC	t1Gd	F	70	74.843	AX_3D_SPGR	3	1.89125E+15	8/31/1999	GE MEDICAL SYSTEMS		Signa HDxt	GR	3D	2	9.344	4.476	450	63.86086	1.5	1	20	0.304583	212	320	192	0.5078\0.5078	TCGA-HT-A5RC/1.3.6.1.4.1.14519.5.2.1.2531.4003.231610681115360230926274787101/1.3.6.1.4.1.14519.5.2.1.2531.4003.324452218716694155037396009138
TCGA-LGG	TCGA-HT	TCGA-HT-A5RC	t1	F	70	74.843	SAG_T1	4	1.89125E+15	8/31/1999	GE MEDICAL SYSTEMS		Signa HDxt	SE	2D	4	766.668	17	0	63.86087	1.5	6	90	1.14681	23	288	192	0.4688\0.4688	TCGA-HT-A5RC/1.3.6.1.4.1.14519.5.2.1.2531.4003.231610681115360230926274787101/1.3.6.1.4.1.14519.5.2.1.2531.4003.109664598871282097695886250609
TCGA-LGG	TCGA-HT	TCGA-HT-A5RC	t2	F	70	74.843	3-pl_T2*_FGRE_S	1	1.89125E+15	8/31/1999	GE MEDICAL SYSTEMS		Signa HDxt	GR	2D	5	5.512	1.692	0	63.86085	1.5	10	30	0.231173	15	256	128	1.0156\1.0156	TCGA-HT-A5RC/1.3.6.1.4.1.14519.5.2.1.2531.4003.231610681115360230926274787101/1.3.6.1.4.1.14519.5.2.1.2531.4003.121974323729426673427319472220
TCGA-LGG	TCGA-HT	TCGA-HT-A614	flair	M	47	120.202	Ax_T2_Flair	5	6.74674E+15	12/24/1999	GE MEDICAL SYSTEMS		Signa HDxt	SE\IR	2D	5	9002	145.008	2250	127.7263	3	5	90	0.5385	28	512	192	0.4688\0.4688	TCGA-HT-A614/1.3.6.1.4.1.14519.5.2.1.2531.4003.176367534254999887804695059404/1.3.6.1.4.1.14519.5.2.1.2531.4003.918114974441949509180480975934
TCGA-LGG	TCGA-HT	TCGA-HT-A614	t1Gd	M	47	120.202	=+C_Ax_T1_MP_SPGR	11	6.74674E+15	12/24/1999	GE MEDICAL SYSTEMS		Signa HDxt	GR	3D	2	6.792	2.772	0	127.7263	3	1	13	0.488775	212	320	192	0.5078\0.5078	TCGA-HT-A614/1.3.6.1.4.1.14519.5.2.1.2531.4003.176367534254999887804695059404/1.3.6.1.4.1.14519.5.2.1.2531.4003.657538458835042249023564628808
TCGA-LGG	TCGA-HT	TCGA-HT-A614	t1	M	47	120.202	Ax_T1_MP_SPGR	9	6.74674E+15	12/24/1999	GE MEDICAL SYSTEMS		Signa HDxt	GR	3D	2	6.792	2.772	0	127.7263	3	1	13	0.488775	212	320	192	0.5078\0.5078	TCGA-HT-A614/1.3.6.1.4.1.14519.5.2.1.2531.4003.176367534254999887804695059404/1.3.6.1.4.1.14519.5.2.1.2531.4003.925945533711458317260480099964
TCGA-LGG	TCGA-HT	TCGA-HT-A614	t2	M	47	120.202	Sag_T2_FSE	4	6.74674E+15	12/24/1999	GE MEDICAL SYSTEMS		Signa HDxt	SE	2D	4	3583.34	80.208	0	127.7263	3	6	90	1.0185	24	512	224	0.4688\0.4688	TCGA-HT-A614/1.3.6.1.4.1.14519.5.2.1.2531.4003.176367534254999887804695059404/1.3.6.1.4.1.14519.5.2.1.2531.4003.556634184158803243157182204153
TCGA-LGG	TCGA-HT	TCGA-HT-A61A	flair	F	20	73.936	(OPT)_FLAIR_AX	14	2.14144E+15	1/27/2000	GE MEDICAL SYSTEMS		SIGNA HDx	SE\IR	2D	2	9002	127.7	2200	127.7243	3	2	90	1.2709	88	256	224	1.0156\1.0156	TCGA-HT-A61A/1.3.6.1.4.1.14519.5.2.1.2531.4003.176077465885263584703190609361/1.3.6.1.4.1.14519.5.2.1.2531.4003.548888566112287264452476674687
TCGA-LGG	TCGA-HT	TCGA-HT-A61A	t1Gd	F	20	73.936	=+C_3D_AXIAL IRSPGR Fast	18	2.14144E+15	1/27/2000	GE MEDICAL SYSTEMS		SIGNA HDx	GR	3D	1.2	6.392	2.524	800	127.7243	3	1.2			148	224	192	1.0156\1.0156	TCGA-HT-A61A/1.3.6.1.4.1.14519.5.2.1.2531.4003.176077465885263584703190609361/1.3.6.1.4.1.14519.5.2.1.2531.4003.102441147751659163225639430837
TCGA-LGG	TCGA-HT	TCGA-HT-A61A	t1	F	20	73.936	3D_AXIAL IRSPGR Fast	16	2.14144E+15	1/27/2000	GE MEDICAL SYSTEMS		SIGNA HDx	GR	3D	1.2	6.392	2.524	800	127.7243	3	1.2			148	224	192	1.0156\1.0156	TCGA-HT-A61A/1.3.6.1.4.1.14519.5.2.1.2531.4003.176077465885263584703190609361/1.3.6.1.4.1.14519.5.2.1.2531.4003.325665435231731333959547617273
TCGA-LGG	TCGA-HT	TCGA-HT-A61A	t2	F	20	73.936	(OPTIONAL)Ax_T2_FSE_INTER	15	2.14144E+15	1/27/2000	GE MEDICAL SYSTEMS		SIGNA HDx	SE	2D	2	4500	81.072	0	127.7244	3	2	90	1.3554	83	256	192	1.0156\1.0156	TCGA-HT-A61A/1.3.6.1.4.1.14519.5.2.1.2531.4003.176077465885263584703190609361/1.3.6.1.4.1.14519.5.2.1.2531.4003.225099482324673279939207452331

**Table 4:** List of TCG-GBM and TCGA-LGG identifiers included in BraTS’15 and this study

**Feature Label**	**Type of Feature**	**Description**
ID	Subject identifier	The TCGA subject's identifier.
Date	Date of scan	The date of the scan considered.
VOLUME_ET	Volumetric	The volume of the enhancing (ET) part of the tumor core (TC).
VOLUME_NET	Volumetric	The volume of the non-enhancing (NET) parts of the tumor core (TC).
VOLUME_ED	Volumetric	The volume of the peritumoral edematous region (ED).
VOLUME_TC	Volumetric	The volume of the tumor core (TC), comprising the ET and NET.
VOLUME_WT	Volumetric	The volume of the whole tumor (WT), comprising ET, NET, and ED.
VOLUME_BRAIN	Volumetric	The volume of the complete brain, excluding skull structures.
VOLUME_ET_OVER_NET	Volumetric	The volume of the ET relative to the volume of the NET.
VOLUME_ET_OVER_ED	Volumetric	The volume of the ET relative to the volume of the ED.
VOLUME_NET_OVER_ED	Volumetric	The volume of the NET relative to the volume of the ED.
VOLUME_ET_over_TC	Volumetric	The volume of the ET relative to the volume of the TC.
VOLUME_NET_over_TC	Volumetric	The volume of the NET relative to the volume of the TC.
VOLUME_ED_over_TC	Volumetric	The volume of the ED relative to the volume of the TC.
VOLUME_ET_OVER_WT	Volumetric	The volume of the ET relative to the volume of the WT.
VOLUME_NET_OVER_WT	Volumetric	The volume of the NET relative to the volume of the WT.
VOLUME_ED_OVER_WT	Volumetric	The volume of the ED relative to the volume of the WT.
VOLUME_TC_OVER_WT	Volumetric	The volume of the TC relative to the volume of the WT.
VOLUME_ET_OVER_BRAIN	Volumetric	The volume of the ET relative to the volume of the complete brain.
VOLUME_NET_OVER_BRAIN	Volumetric	The volume of the NET relative to the volume of the complete brain.
VOLUME_ED_over_BRAIN	Volumetric	The volume of the ED relative to the volume of the complete brain.
VOLUME_TC_over_BRAIN	Volumetric	The volume of the TC relative to the volume of the complete brain.
VOLUME_WT_OVER_BRAIN	Volumetric	The volume of the WT relative to the volume of the complete brain.
DIST_Vent_TC	Spatial	The closest distance of the TC from the ventricles.
DIST_Vent_ED	Spatial	The closest distance of the ED from the ventricles.
INTENSITY_Mean_ET_T1Gd	Intensity	The raw average intensity of the ET in the post-contrast T1 (T1-Gd) volume.
INTENSITY_STD_ET_T1Gd	Intensity	The standard deviation of the raw intensities of the ET in the T1-Gd volume.
INTENSITY_Mean_ET_T1	Intensity	The raw average intensity of the ET in the pre-contrast T1 (T1) volume.
INTENSITY_STD_ET_T1	Intensity	The standard deviation of the raw intensities of the ET in the T1 volume.
INTENSITY_Mean_ET_T2	Intensity	The raw average intensity of the ET in the T2-weighted (T2) volume.
INTENSITY_STD_ET_T2	Intensity	The standard deviation of the raw intensities of the ET in the T2 volume.
INTENSITY_Mean_ET_FLAIR	Intensity	The raw average intensity of the ET in the T2-weighted Fluid Attenuated Inversion Recovery (FLAIR) volume.
INTENSITY_STD_ET_FLAIR	Intensity	The standard deviation of the raw intensities of the ET in the FLAIR volume.
INTENSITY_Mean_NET_T1Gd	Intensity	The raw average intensity of the NET in the T1-Gd volume.
INTENSITY_STD_NET_T1Gd	Intensity	The standard deviation of the raw intensities of the NET in the T1-Gd volume.
INTENSITY_Mean_NET_T1	Intensity	The raw average intensity of the NET in the T1 volume.
INTENSITY_STD_NET_T1	Intensity	The standard deviation of the raw intensities of the NET in the T1 volume.
INTENSITY_Mean_NET_T2	Intensity	The raw average intensity of the NET in the T2 volume.
INTENSITY_STD_NET_T2	Intensity	The standard deviation of the raw intensities of the NET in the T2 volume.
INTENSITY_Mean_NET_FLAIR	Intensity	The raw average intensity of the NET in the FLAIR volume.
INTENSITY_STD_NET_FLAIR	Intensity	The standard deviation of the raw intensities of the NET in the FLAIR volume.
INTENSITY_Mean_ED_T1Gd	Intensity	The raw average intensity of the ED in the T1-Gd volume.
INTENSITY_STD_ED_T1Gd	Intensity	The standard deviation of the raw intensities of the ED in the T1-Gd volume.
INTENSITY_Mean_ED_T1	Intensity	The raw average intensity of the ED in the T1 volume.
INTENSITY_STD_ED_T1	Intensity	The standard deviation of the raw intensities of the ED in the T1 volume.
INTENSITY_Mean_ED_T2	Intensity	The raw average intensity of the ED in the T2 volume.
INTENSITY_STD_ED_T2	Intensity	The standard deviation of the raw intensities of the ED in the T2 volume.
INTENSITY_Mean_ED_FLAIR	Intensity	The raw average intensity of the ED in the FLAIR volume.
INTENSITY_STD_ED_FLAIR	Intensity	The standard deviation of the raw intensities of the ED in the FLAIR volume.
HISTO_ET_T1Gd_Bin1	Histogram-based	Binned histogram of the raw intensity values of the ET in the T1-Gd volume (Bin 1).
HISTO_ET_T1Gd_Bin2	Histogram-based	Binned histogram of the raw intensity values of the ET in the T1-Gd volume (Bin 2).
HISTO_ET_T1Gd_Bin3	Histogram-based	Binned histogram of the raw intensity values of the ET in the T1-Gd volume (Bin 3).
HISTO_ET_T1Gd_Bin4	Histogram-based	Binned histogram of the raw intensity values of the ET in the T1-Gd volume (Bin 4).
HISTO_ET_T1Gd_Bin5	Histogram-based	Binned histogram of the raw intensity values of the ET in the T1-Gd volume (Bin 5).
HISTO_ET_T1Gd_Bin6	Histogram-based	Binned histogram of the raw intensity values of the ET in the T1-Gd volume (Bin 6).
HISTO_ET_T1Gd_Bin7	Histogram-based	Binned histogram of the raw intensity values of the ET in the T1-Gd volume (Bin 7).
HISTO_ET_T1Gd_Bin8	Histogram-based	Binned histogram of the raw intensity values of the ET in the T1-Gd volume (Bin 8).
HISTO_ET_T1Gd_Bin9	Histogram-based	Binned histogram of the raw intensity values of the ET in the T1-Gd volume (Bin 9).
HISTO_ET_T1Gd_Bin10	Histogram-based	Binned histogram of the raw intensity values of the ET in the T1-Gd volume (Bin 10).
HISTO_ED_T1Gd_Bin1	Histogram-based	Binned histogram of the raw intensity values of the ED in the T1-Gd volume (Bin 1).
HISTO_ED_T1Gd_Bin2	Histogram-based	Binned histogram of the raw intensity values of the ED in the T1-Gd volume (Bin 2).
HISTO_ED_T1Gd_Bin3	Histogram-based	Binned histogram of the raw intensity values of the ED in the T1-Gd volume (Bin 3).
HISTO_ED_T1Gd_Bin4	Histogram-based	Binned histogram of the raw intensity values of the ED in the T1-Gd volume (Bin 4).
HISTO_ED_T1Gd_Bin5	Histogram-based	Binned histogram of the raw intensity values of the ED in the T1-Gd volume (Bin 5).
HISTO_ED_T1Gd_Bin6	Histogram-based	Binned histogram of the raw intensity values of the ED in the T1-Gd volume (Bin 6).
HISTO_ED_T1Gd_Bin7	Histogram-based	Binned histogram of the raw intensity values of the ED in the T1-Gd volume (Bin 7).
HISTO_ED_T1Gd_Bin8	Histogram-based	Binned histogram of the raw intensity values of the ED in the T1-Gd volume (Bin 8).
HISTO_ED_T1Gd_Bin9	Histogram-based	Binned histogram of the raw intensity values of the ED in the T1-Gd volume (Bin 9).
HISTO_ED_T1Gd_Bin10	Histogram-based	Binned histogram of the raw intensity values of the ED in the T1-Gd volume (Bin 10).
HISTO_NET_T1Gd_Bin1	Histogram-based	Binned histogram of the raw intensity values of the NET in the T1-Gd volume (Bin 1).
HISTO_NET_T1Gd_Bin2	Histogram-based	Binned histogram of the raw intensity values of the NET in the T1-Gd volume (Bin 2).
HISTO_NET_T1Gd_Bin3	Histogram-based	Binned histogram of the raw intensity values of the NET in the T1-Gd volume (Bin 3).
HISTO_NET_T1Gd_Bin4	Histogram-based	Binned histogram of the raw intensity values of the NET in the T1-Gd volume (Bin 4).
HISTO_NET_T1Gd_Bin5	Histogram-based	Binned histogram of the raw intensity values of the NET in the T1-Gd volume (Bin 5).
HISTO_NET_T1Gd_Bin6	Histogram-based	Binned histogram of the raw intensity values of the NET in the T1-Gd volume (Bin 6).
HISTO_NET_T1Gd_Bin7	Histogram-based	Binned histogram of the raw intensity values of the NET in the T1-Gd volume (Bin 7).
HISTO_NET_T1Gd_Bin8	Histogram-based	Binned histogram of the raw intensity values of the NET in the T1-Gd volume (Bin 8).
HISTO_NET_T1Gd_Bin9	Histogram-based	Binned histogram of the raw intensity values of the NET in the T1-Gd volume (Bin 9).
HISTO_NET_T1Gd_Bin10	Histogram-based	Binned histogram of the raw intensity values of the NET in the T1-Gd volume (Bin 10).
HISTO_ET_T1_Bin1	Histogram-based	Binned histogram of the raw intensity values of the ET in the T1 volume (Bin 1).
HISTO_ET_T1_Bin2	Histogram-based	Binned histogram of the raw intensity values of the ET in the T1 volume (Bin 2).
HISTO_ET_T1_Bin3	Histogram-based	Binned histogram of the raw intensity values of the ET in the T1 volume (Bin 3).
HISTO_ET_T1_Bin4	Histogram-based	Binned histogram of the raw intensity values of the ET in the T1 volume (Bin 4).
HISTO_ET_T1_Bin5	Histogram-based	Binned histogram of the raw intensity values of the ET in the T1 volume (Bin 5).
HISTO_ET_T1_Bin6	Histogram-based	Binned histogram of the raw intensity values of the ET in the T1 volume (Bin 6).
HISTO_ET_T1_Bin7	Histogram-based	Binned histogram of the raw intensity values of the ET in the T1 volume (Bin 7).
HISTO_ET_T1_Bin8	Histogram-based	Binned histogram of the raw intensity values of the ET in the T1 volume (Bin 8).
HISTO_ET_T1_Bin9	Histogram-based	Binned histogram of the raw intensity values of the ET in the T1 volume (Bin 9).
HISTO_ET_T1_Bin10	Histogram-based	Binned histogram of the raw intensity values of the ET in the T1 volume (Bin 10).
HISTO_ED_T1_Bin1	Histogram-based	Binned histogram of the raw intensity values of the ED in the T1 volume (Bin 1).
HISTO_ED_T1_Bin2	Histogram-based	Binned histogram of the raw intensity values of the ED in the T1 volume (Bin 2).
HISTO_ED_T1_Bin3	Histogram-based	Binned histogram of the raw intensity values of the ED in the T1 volume (Bin 3).
HISTO_ED_T1_Bin4	Histogram-based	Binned histogram of the raw intensity values of the ED in the T1 volume (Bin 4).
HISTO_ED_T1_Bin5	Histogram-based	Binned histogram of the raw intensity values of the ED in the T1 volume (Bin 5).
HISTO_ED_T1_Bin6	Histogram-based	Binned histogram of the raw intensity values of the ED in the T1 volume (Bin 6).
HISTO_ED_T1_Bin7	Histogram-based	Binned histogram of the raw intensity values of the ED in the T1 volume (Bin 7).
HISTO_ED_T1_Bin8	Histogram-based	Binned histogram of the raw intensity values of the ED in the T1 volume (Bin 8).
HISTO_ED_T1_Bin9	Histogram-based	Binned histogram of the raw intensity values of the ED in the T1 volume (Bin 9).
HISTO_ED_T1_Bin10	Histogram-based	Binned histogram of the raw intensity values of the ED in the T1 volume (Bin 10).
HISTO_NET_T1_Bin1	Histogram-based	Binned histogram of the raw intensity values of the NET in the T1 volume (Bin 1).
HISTO_NET_T1_Bin2	Histogram-based	Binned histogram of the raw intensity values of the NET in the T1 volume (Bin 2).
HISTO_NET_T1_Bin3	Histogram-based	Binned histogram of the raw intensity values of the NET in the T1 volume (Bin 3).
HISTO_NET_T1_Bin4	Histogram-based	Binned histogram of the raw intensity values of the NET in the T1 volume (Bin 4).
HISTO_NET_T1_Bin5	Histogram-based	Binned histogram of the raw intensity values of the NET in the T1 volume (Bin 5).
HISTO_NET_T1_Bin6	Histogram-based	Binned histogram of the raw intensity values of the NET in the T1 volume (Bin 6).
HISTO_NET_T1_Bin7	Histogram-based	Binned histogram of the raw intensity values of the NET in the T1 volume (Bin 7).
HISTO_NET_T1_Bin8	Histogram-based	Binned histogram of the raw intensity values of the NET in the T1 volume (Bin 8).
HISTO_NET_T1_Bin9	Histogram-based	Binned histogram of the raw intensity values of the NET in the T1 volume (Bin 9).
HISTO_NET_T1_Bin10	Histogram-based	Binned histogram of the raw intensity values of the NET in the T1 volume (Bin 10).
HISTO_ET_T2_Bin1	Histogram-based	Binned histogram of the raw intensity values of the ET in the T2 volume (Bin 1).
HISTO_ET_T2_Bin2	Histogram-based	Binned histogram of the raw intensity values of the ET in the T2 volume (Bin 2).
HISTO_ET_T2_Bin3	Histogram-based	Binned histogram of the raw intensity values of the ET in the T2 volume (Bin 3).
HISTO_ET_T2_Bin4	Histogram-based	Binned histogram of the raw intensity values of the ET in the T2 volume (Bin 4).
HISTO_ET_T2_Bin5	Histogram-based	Binned histogram of the raw intensity values of the ET in the T2 volume (Bin 5).
HISTO_ET_T2_Bin6	Histogram-based	Binned histogram of the raw intensity values of the ET in the T2 volume (Bin 6).
HISTO_ET_T2_Bin7	Histogram-based	Binned histogram of the raw intensity values of the ET in the T2 volume (Bin 7).
HISTO_ET_T2_Bin8	Histogram-based	Binned histogram of the raw intensity values of the ET in the T2 volume (Bin 8).
HISTO_ET_T2_Bin9	Histogram-based	Binned histogram of the raw intensity values of the ET in the T2 volume (Bin 9).
HISTO_ET_T2_Bin10	Histogram-based	Binned histogram of the raw intensity values of the ET in the T2 volume (Bin 10).
HISTO_ED_T2_Bin1	Histogram-based	Binned histogram of the raw intensity values of the ED in the T2 volume (Bin 1).
HISTO_ED_T2_Bin2	Histogram-based	Binned histogram of the raw intensity values of the ED in the T2 volume (Bin 2).
HISTO_ED_T2_Bin3	Histogram-based	Binned histogram of the raw intensity values of the ED in the T2 volume (Bin 3).
HISTO_ED_T2_Bin4	Histogram-based	Binned histogram of the raw intensity values of the ED in the T2 volume (Bin 4).
HISTO_ED_T2_Bin5	Histogram-based	Binned histogram of the raw intensity values of the ED in the T2 volume (Bin 5).
HISTO_ED_T2_Bin6	Histogram-based	Binned histogram of the raw intensity values of the ED in the T2 volume (Bin 6).
HISTO_ED_T2_Bin7	Histogram-based	Binned histogram of the raw intensity values of the ED in the T2 volume (Bin 7).
HISTO_ED_T2_Bin8	Histogram-based	Binned histogram of the raw intensity values of the ED in the T2 volume (Bin 8).
HISTO_ED_T2_Bin9	Histogram-based	Binned histogram of the raw intensity values of the ED in the T2 volume (Bin 9).
HISTO_ED_T2_Bin10	Histogram-based	Binned histogram of the raw intensity values of the ED in the T2 volume (Bin 10).
HISTO_NET_T2_Bin1	Histogram-based	Binned histogram of the raw intensity values of the NET in the T2 volume (Bin 1).
HISTO_NET_T2_Bin2	Histogram-based	Binned histogram of the raw intensity values of the NET in the T2 volume (Bin 2).
HISTO_NET_T2_Bin3	Histogram-based	Binned histogram of the raw intensity values of the NET in the T2 volume (Bin 3).
HISTO_NET_T2_Bin4	Histogram-based	Binned histogram of the raw intensity values of the NET in the T2 volume (Bin 4).
HISTO_NET_T2_Bin5	Histogram-based	Binned histogram of the raw intensity values of the NET in the T2 volume (Bin 5).
HISTO_NET_T2_Bin6	Histogram-based	Binned histogram of the raw intensity values of the NET in the T2 volume (Bin 6).
HISTO_NET_T2_Bin7	Histogram-based	Binned histogram of the raw intensity values of the NET in the T2 volume (Bin 7).
HISTO_NET_T2_Bin8	Histogram-based	Binned histogram of the raw intensity values of the NET in the T2 volume (Bin 8).
HISTO_NET_T2_Bin9	Histogram-based	Binned histogram of the raw intensity values of the NET in the T2 volume (Bin 9).
HISTO_NET_T2_Bin10	Histogram-based	Binned histogram of the raw intensity values of the NET in the T2 volume (Bin 10).
HISTO_ET_FLAIR_Bin1	Histogram-based	Binned histogram of the raw intensity values of the ET in the FLAIR volume (Bin 1).
HISTO_ET_FLAIR_Bin2	Histogram-based	Binned histogram of the raw intensity values of the ET in the FLAIR volume (Bin 2).
HISTO_ET_FLAIR_Bin3	Histogram-based	Binned histogram of the raw intensity values of the ET in the FLAIR volume (Bin 3).
HISTO_ET_FLAIR_Bin4	Histogram-based	Binned histogram of the raw intensity values of the ET in the FLAIR volume (Bin 4).
HISTO_ET_FLAIR_Bin5	Histogram-based	Binned histogram of the raw intensity values of the ET in the FLAIR volume (Bin 5).
HISTO_ET_FLAIR_Bin6	Histogram-based	Binned histogram of the raw intensity values of the ET in the FLAIR volume (Bin 6).
HISTO_ET_FLAIR_Bin7	Histogram-based	Binned histogram of the raw intensity values of the ET in the FLAIR volume (Bin 7).
HISTO_ET_FLAIR_Bin8	Histogram-based	Binned histogram of the raw intensity values of the ET in the FLAIR volume (Bin 8).
HISTO_ET_FLAIR_Bin9	Histogram-based	Binned histogram of the raw intensity values of the ET in the FLAIR volume (Bin 9).
HISTO_ET_FLAIR_Bin10	Histogram-based	Binned histogram of the raw intensity values of the ET in the FLAIR volume (Bin 10).
HISTO_ED_FLAIR_Bin1	Histogram-based	Binned histogram of the raw intensity values of the ED in the FLAIR volume (Bin 1).
HISTO_ED_FLAIR_Bin2	Histogram-based	Binned histogram of the raw intensity values of the ED in the FLAIR volume (Bin 2).
HISTO_ED_FLAIR_Bin3	Histogram-based	Binned histogram of the raw intensity values of the ED in the FLAIR volume (Bin 3).
HISTO_ED_FLAIR_Bin4	Histogram-based	Binned histogram of the raw intensity values of the ED in the FLAIR volume (Bin 4).
HISTO_ED_FLAIR_Bin5	Histogram-based	Binned histogram of the raw intensity values of the ED in the FLAIR volume (Bin 5).
HISTO_ED_FLAIR_Bin6	Histogram-based	Binned histogram of the raw intensity values of the ED in the FLAIR volume (Bin 6).
HISTO_ED_FLAIR_Bin7	Histogram-based	Binned histogram of the raw intensity values of the ED in the FLAIR volume (Bin 7).
HISTO_ED_FLAIR_Bin8	Histogram-based	Binned histogram of the raw intensity values of the ED in the FLAIR volume (Bin 8).
HISTO_ED_FLAIR_Bin9	Histogram-based	Binned histogram of the raw intensity values of the ED in the FLAIR volume (Bin 9).
HISTO_ED_FLAIR_Bin10	Histogram-based	Binned histogram of the raw intensity values of the ED in the FLAIR volume (Bin 10).
HISTO_NET_FLAIR_Bin1	Histogram-based	Binned histogram of the raw intensity values of the NET in the FLAIR volume (Bin 1).
HISTO_NET_FLAIR_Bin2	Histogram-based	Binned histogram of the raw intensity values of the NET in the FLAIR volume (Bin 2).
HISTO_NET_FLAIR_Bin3	Histogram-based	Binned histogram of the raw intensity values of the NET in the FLAIR volume (Bin 3).
HISTO_NET_FLAIR_Bin4	Histogram-based	Binned histogram of the raw intensity values of the NET in the FLAIR volume (Bin 4).
HISTO_NET_FLAIR_Bin5	Histogram-based	Binned histogram of the raw intensity values of the NET in the FLAIR volume (Bin 5).
HISTO_NET_FLAIR_Bin6	Histogram-based	Binned histogram of the raw intensity values of the NET in the FLAIR volume (Bin 6).
HISTO_NET_FLAIR_Bin7	Histogram-based	Binned histogram of the raw intensity values of the NET in the FLAIR volume (Bin 7).
HISTO_NET_FLAIR_Bin8	Histogram-based	Binned histogram of the raw intensity values of the NET in the FLAIR volume (Bin 8).
HISTO_NET_FLAIR_Bin9	Histogram-based	Binned histogram of the raw intensity values of the NET in the FLAIR volume (Bin 9).
HISTO_NET_FLAIR_Bin10	Histogram-based	Binned histogram of the raw intensity values of the NET in the FLAIR volume (Bin 10).
SPATIAL_Frontal	Spatial	Percentage of TC volume in the frontal lobe.
SPATIAL_Temporal	Spatial	Percentage of TC volume in the temporal lobe.
SPATIAL_Parietal	Spatial	Percentage of TC volume in the parietal lobe.
SPATIAL_Basal_G	Spatial	Percentage of TC volume in the regions of putamen, caudate nucleus, globus pallidus, subthalamic nucleus, nucleus accumbens, internal capsule, and thalamus.
SPATIAL_Insula	Spatial	Percentage of TC volume in the region of the insula.
SPATIAL_CC_Fornix	Spatial	Percentage of TC volume in the regions of corpus callosum, fornix, and cingulate.
SPATIAL_Occipital	Spatial	Percentage of TC volume in the occipital lobe.
SPATIAL_Cere	Spatial	Percentage of TC volume in the region of the cerebellum.
SPATIAL_Brain_stem	Spatial	Percentage of TC volume in the region of brain stem.
ECCENTRICITY_ET	Volumetric	Eccentricity metric for ET: sqrt[1 - a*b/c^2], where c is the longest semi-principal axes of an ellipsoid fitted on the ET, and a and b are the 2nd and 3rd longest semi-principal axes of the ellipsoid.
ECCENTRICITY_ED	Volumetric	Eccentricity metric for ED: sqrt[1 - a*b/c^2], where c is the longest semi-principal axes of an ellipsoid fitted on the ED, and a and b are the 2nd and 3rd longest semi-principal axes of the ellipsoid.
ECCENTRICITY_NET	Volumetric	Eccentricity metric for NET: sqrt[1 - a*b/c^2], where c is the longest semi-principal axes of an ellipsoid fitted on the NET, and a and b are the 2nd and 3rd longest semi-principal axes of the ellipsoid.
SOLIDITY_ET	Volumetric	Ratio of the number of voxels in the ET to the number of voxels in the 3D convex hull of the ET.
SOLIDITY_ED	Volumetric	Ratio of the number of voxels in the ED to the number of voxels in the 3D convex hull of the ED.
SOLIDITY_NET	Volumetric	Ratio of the number of voxels in the NET to the number of voxels in the 3D convex hull of the NET.
TEXTURE_GLOBAL_ET_T1Gd_Variance	Textural (Global)	Variance of the histogram of the ET in the T1-Gd volume.
TEXTURE_GLOBAL_ET_T1Gd_Skewness	Textural (Global)	Skewness of the histogram of the ET in the T1-Gd volume.
TEXTURE_GLOBAL_ET_T1Gd_Kurtosis	Textural (Global)	Kurtosis of the histogram of the ET in the T1-Gd volume.
TEXTURE_GLOBAL_ET_T1_Variance	Textural (Global)	Variance of the histogram of the ET in the T1 volume.
TEXTURE_GLOBAL_ET_T1_Skewness	Textural (Global)	Skewness of the histogram of the ET in the T1 volume.
TEXTURE_GLOBAL_ET_T1_Kurtosis	Textural (Global)	Kurtosis of the histogram of the ET in the T1 volume.
TEXTURE_GLOBAL_ET_T2_Variance	Textural (Global)	Variance of the histogram of the ET in the T2 volume.
TEXTURE_GLOBAL_ET_T2_Skewness	Textural (Global)	Skewness of the histogram of the ET in the T2 volume.
TEXTURE_GLOBAL_ET_T2_Kurtosis	Textural (Global)	Kurtosis of the histogram of the ET in the T2 volume.
TEXTURE_GLOBAL_ET_FLAIR_Variance	Textural (Global)	Variance of the histogram of the ET in the FLAIR volume.
TEXTURE_GLOBAL_ET_FLAIR_Skewness	Textural (Global)	Skewness of the histogram of the ET in the FLAIR volume.
TEXTURE_GLOBAL_ET_FLAIR_Kurtosis	Textural (Global)	Kurtosis of the histogram of the ET in the FLAIR volume.
TEXTURE_GLOBAL_ED_T1Gd_Variance	Textural (Global)	Variance of the histogram of the ED in the T1-Gd volume.
TEXTURE_GLOBAL_ED_T1Gd_Skewness	Textural (Global)	Skewness of the histogram of the ED in the T1-Gd volume.
TEXTURE_GLOBAL_ED_T1Gd_Kurtosis	Textural (Global)	Kurtosis of the histogram of the ED in the T1-Gd volume.
TEXTURE_GLOBAL_ED_T1_Variance	Textural (Global)	Variance of the histogram of the ED in the T1 volume.
TEXTURE_GLOBAL_ED_T1_Skewness	Textural (Global)	Skewness of the histogram of the ED in the T1 volume.
TEXTURE_GLOBAL_ED_T1_Kurtosis	Textural (Global)	Kurtosis of the histogram of the ED in the T1 volume.
TEXTURE_GLOBAL_ED_T2_Variance	Textural (Global)	Variance of the histogram of the ED in the T2 volume.
TEXTURE_GLOBAL_ED_T2_Skewness	Textural (Global)	Skewness of the histogram of the ED in the T2 volume.
TEXTURE_GLOBAL_ED_T2_Kurtosis	Textural (Global)	Kurtosis of the histogram of the ED in the T2 volume.
TEXTURE_GLOBAL_ED_FLAIR_Variance	Textural (Global)	Variance of the histogram of the ED in the FLAIR volume.
TEXTURE_GLOBAL_ED_FLAIR_Skewness	Textural (Global)	Skewness of the histogram of the ED in the FLAIR volume.
TEXTURE_GLOBAL_ED_FLAIR_Kurtosis	Textural (Global)	Kurtosis of the histogram of the ED in the FLAIR volume.
TEXTURE_GLOBAL_NET_T1Gd_Variance	Textural (Global)	Variance of the histogram of the NET in the T1-Gd volume.
TEXTURE_GLOBAL_NET_T1Gd_Skewness	Textural (Global)	Skewness of the histogram of the NET in the T1-Gd volume.
TEXTURE_GLOBAL_NET_T1Gd_Kurtosis	Textural (Global)	Kurtosis of the histogram of the NET in the T1-Gd volume.
TEXTURE_GLOBAL_NET_T1_Variance	Textural (Global)	Variance of the histogram of the NET in the T1 volume.
TEXTURE_GLOBAL_NET_T1_Skewness	Textural (Global)	Skewness of the histogram of the NET in the T1 volume.
TEXTURE_GLOBAL_NET_T1_Kurtosis	Textural (Global)	Kurtosis of the histogram of the NET in the T1 volume.
TEXTURE_GLOBAL_NET_T2_Variance	Textural (Global)	Variance of the histogram of the NET in the T2 volume.
TEXTURE_GLOBAL_NET_T2_Skewness	Textural (Global)	Skewness of the histogram of the NET in the T2 volume.
TEXTURE_GLOBAL_NET_T2_Kurtosis	Textural (Global)	Kurtosis of the histogram of the NET in the T2 volume.
TEXTURE_GLOBAL_NET_FLAIR_Variance	Textural (Global)	Variance of the histogram of the NET in the FLAIR volume.
TEXTURE_GLOBAL_NET_FLAIR_Skewness	Textural (Global)	Skewness of the histogram of the NET in the FLAIR volume.
TEXTURE_GLOBAL_NET_FLAIR_Kurtosis	Textural (Global)	Kurtosis of the histogram of the NET in the FLAIR volume.
TEXTURE_GLCM_ET_T1Gd_Energy	Textural (Grey-Level Co-occurrence Matrix)	The Energy within the ET in the T1-Gd volume, considering 26-connected neighboring voxels in the 3D volume.
TEXTURE_GLCM_ET_T1Gd_Contrast	Textural (Grey-Level Co-occurrence Matrix)	The Contrast within the ET in the T1-Gd volume, considering 26-connected neighboring voxels in the 3D volume.
TEXTURE_GLCM_ET_T1Gd_Entropy	Textural (Grey-Level Co-occurrence Matrix)	The Entropy within the ET in the T1-Gd volume, considering 26-connected neighboring voxels in the 3D volume.
TEXTURE_GLCM_ET_T1Gd_Homogeneity	Textural (Grey-Level Co-occurrence Matrix)	The Homogeneity within the ET in the T1-Gd volume, considering 26-connected neighboring voxels in the 3D volume.
TEXTURE_GLCM_ET_T1Gd_Correlation	Textural (Grey-Level Co-occurrence Matrix)	The Correlation within the ET in the T1-Gd volume, considering 26-connected neighboring voxels in the 3D volume.
TEXTURE_GLCM_ET_T1Gd_SumAverage	Textural (Grey-Level Co-occurrence Matrix)	The SumAverage within the ET in the T1-Gd volume, considering 26-connected neighboring voxels in the 3D volume.
TEXTURE_GLCM_ET_T1Gd_Variance	Textural (Grey-Level Co-occurrence Matrix)	The Variance within the ET in the T1-Gd volume, considering 26-connected neighboring voxels in the 3D volume.
TEXTURE_GLCM_ET_T1Gd_Dissimilarity	Textural (Grey-Level Co-occurrence Matrix)	The Dissimilarity within the ET in the T1-Gd volume, considering 26-connected neighboring voxels in the 3D volume.
TEXTURE_GLCM_ET_T1Gd_AutoCorrelation	Textural (Grey-Level Co-occurrence Matrix)	The Autocorrelation within the ET in the T1-Gd volume, considering 26-connected neighboring voxels in the 3D volume.
TEXTURE_GLCM_ET_T1_Energy	Textural (Grey-Level Co-occurrence Matrix)	The Energy within the ET in the T1 volume, considering 26-connected neighboring voxels in the 3D volume.
TEXTURE_GLCM_ET_T1_Contrast	Textural (Grey-Level Co-occurrence Matrix)	The Contrast within the ET in the T1 volume, considering 26-connected neighboring voxels in the 3D volume.
TEXTURE_GLCM_ET_T1_Entropy	Textural (Grey-Level Co-occurrence Matrix)	The Entropy within the ET in the T1 volume, considering 26-connected neighboring voxels in the 3D volume.
TEXTURE_GLCM_ET_T1_Homogeneity	Textural (Grey-Level Co-occurrence Matrix)	The Homogeneity within the ET in the T1 volume, considering 26-connected neighboring voxels in the 3D volume.
TEXTURE_GLCM_ET_T1_Correlation	Textural (Grey-Level Co-occurrence Matrix)	The Correlation within the ET in the T1 volume, considering 26-connected neighboring voxels in the 3D volume.
TEXTURE_GLCM_ET_T1_SumAverage	Textural (Grey-Level Co-occurrence Matrix)	The SumAverage within the ET in the T1 volume, considering 26-connected neighboring voxels in the 3D volume.
TEXTURE_GLCM_ET_T1_Variance	Textural (Grey-Level Co-occurrence Matrix)	The Variance within the ET in the T1 volume, considering 26-connected neighboring voxels in the 3D volume.
TEXTURE_GLCM_ET_T1_Dissimilarity	Textural (Grey-Level Co-occurrence Matrix)	The Dissimilarity within the ET in the T1 volume, considering 26-connected neighboring voxels in the 3D volume.
TEXTURE_GLCM_ET_T1_AutoCorrelation	Textural (Grey-Level Co-occurrence Matrix)	The Autocorrelation within the ET in the T1 volume, considering 26-connected neighboring voxels in the 3D volume.
TEXTURE_GLCM_ET_T2_Energy	Textural (Grey-Level Co-occurrence Matrix)	The Energy within the ET in the T2 volume, considering 26-connected neighboring voxels in the 3D volume.
TEXTURE_GLCM_ET_T2_Contrast	Textural (Grey-Level Co-occurrence Matrix)	The Contrast within the ET in the T2 volume, considering 26-connected neighboring voxels in the 3D volume.
TEXTURE_GLCM_ET_T2_Entropy	Textural (Grey-Level Co-occurrence Matrix)	The Entropy within the ET in the T2 volume, considering 26-connected neighboring voxels in the 3D volume.
TEXTURE_GLCM_ET_T2_Homogeneity	Textural (Grey-Level Co-occurrence Matrix)	The Homogeneity within the ET in the T2 volume, considering 26-connected neighboring voxels in the 3D volume.
TEXTURE_GLCM_ET_T2_Correlation	Textural (Grey-Level Co-occurrence Matrix)	The Correlation within the ET in the T2 volume, considering 26-connected neighboring voxels in the 3D volume.
TEXTURE_GLCM_ET_T2_SumAverage	Textural (Grey-Level Co-occurrence Matrix)	The SumAverage within the ET in the T2 volume, considering 26-connected neighboring voxels in the 3D volume.
TEXTURE_GLCM_ET_T2_Variance	Textural (Grey-Level Co-occurrence Matrix)	The Variance within the ET in the T2 volume, considering 26-connected neighboring voxels in the 3D volume.
TEXTURE_GLCM_ET_T2_Dissimilarity	Textural (Grey-Level Co-occurrence Matrix)	The Dissimilarity within the ET in the T2 volume, considering 26-connected neighboring voxels in the 3D volume.
TEXTURE_GLCM_ET_T2_AutoCorrelation	Textural (Grey-Level Co-occurrence Matrix)	The Autocorrelation within the ET in the T2 volume, considering 26-connected neighboring voxels in the 3D volume.
TEXTURE_GLCM_ET_FLAIR_Energy	Textural (Grey-Level Co-occurrence Matrix)	The Energy within the ET in the FLAIR volume, considering 26-connected neighboring voxels in the 3D volume.
TEXTURE_GLCM_ET_FLAIR_Contrast	Textural (Grey-Level Co-occurrence Matrix)	The Contrast within the ET in the FLAIR volume, considering 26-connected neighboring voxels in the 3D volume.
TEXTURE_GLCM_ET_FLAIR_Entropy	Textural (Grey-Level Co-occurrence Matrix)	The Entropy within the ET in the FLAIR volume, considering 26-connected neighboring voxels in the 3D volume.
TEXTURE_GLCM_ET_FLAIR_Homogeneity	Textural (Grey-Level Co-occurrence Matrix)	The Homogeneity within the ET in the FLAIR volume, considering 26-connected neighboring voxels in the 3D volume.
TEXTURE_GLCM_ET_FLAIR_Correlation	Textural (Grey-Level Co-occurrence Matrix)	The Correlation within the ET in the FLAIR volume, considering 26-connected neighboring voxels in the 3D volume.
TEXTURE_GLCM_ET_FLAIR_SumAverage	Textural (Grey-Level Co-occurrence Matrix)	The SumAverage within the ET in the FLAIR volume, considering 26-connected neighboring voxels in the 3D volume.
TEXTURE_GLCM_ET_FLAIR_Variance	Textural (Grey-Level Co-occurrence Matrix)	The Variance within the ET in the FLAIR volume, considering 26-connected neighboring voxels in the 3D volume.
TEXTURE_GLCM_ET_FLAIR_Dissimilarity	Textural (Grey-Level Co-occurrence Matrix)	The Dissimilarity within the ET in the FLAIR volume, considering 26-connected neighboring voxels in the 3D volume.
TEXTURE_GLCM_ET_FLAIR_AutoCorrelation	Textural (Grey-Level Co-occurrence Matrix)	The Autocorrelation within the ET in the FLAIR volume, considering 26-connected neighboring voxels in the 3D volume.
TEXTURE_GLCM_ED_T1Gd_Energy	Textural (Grey-Level Co-occurrence Matrix)	The Energy within the ED in the T1-Gd volume, considering 26-connected neighboring voxels in the 3D volume.
TEXTURE_GLCM_ED_T1Gd_Contrast	Textural (Grey-Level Co-occurrence Matrix)	The Contrast within the ED in the T1-Gd volume, considering 26-connected neighboring voxels in the 3D volume.
TEXTURE_GLCM_ED_T1Gd_Entropy	Textural (Grey-Level Co-occurrence Matrix)	The Entropy within the ED in the T1-Gd volume, considering 26-connected neighboring voxels in the 3D volume.
TEXTURE_GLCM_ED_T1Gd_Homogeneity	Textural (Grey-Level Co-occurrence Matrix)	The Homogeneity within the ED in the T1-Gd volume, considering 26-connected neighboring voxels in the 3D volume.
TEXTURE_GLCM_ED_T1Gd_Correlation	Textural (Grey-Level Co-occurrence Matrix)	The Correlation within the ED in the T1-Gd volume, considering 26-connected neighboring voxels in the 3D volume.
TEXTURE_GLCM_ED_T1Gd_SumAverage	Textural (Grey-Level Co-occurrence Matrix)	The SumAverage within the ED in the T1-Gd volume, considering 26-connected neighboring voxels in the 3D volume.
TEXTURE_GLCM_ED_T1Gd_Variance	Textural (Grey-Level Co-occurrence Matrix)	The Variance within the ED in the T1-Gd volume, considering 26-connected neighboring voxels in the 3D volume.
TEXTURE_GLCM_ED_T1Gd_Dissimilarity	Textural (Grey-Level Co-occurrence Matrix)	The Dissimilarity within the ED in the T1-Gd volume, considering 26-connected neighboring voxels in the 3D volume.
TEXTURE_GLCM_ED_T1Gd_AutoCorrelation	Textural (Grey-Level Co-occurrence Matrix)	The Autocorrelation within the ED in the T1-Gd volume, considering 26-connected neighboring voxels in the 3D volume.
TEXTURE_GLCM_ED_T1_Energy	Textural (Grey-Level Co-occurrence Matrix)	The Energy within the ED in the T1 volume, considering 26-connected neighboring voxels in the 3D volume.
TEXTURE_GLCM_ED_T1_Contrast	Textural (Grey-Level Co-occurrence Matrix)	The Contrast within the ED in the T1 volume, considering 26-connected neighboring voxels in the 3D volume.
TEXTURE_GLCM_ED_T1_Entropy	Textural (Grey-Level Co-occurrence Matrix)	The Entropy within the ED in the T1 volume, considering 26-connected neighboring voxels in the 3D volume.
TEXTURE_GLCM_ED_T1_Homogeneity	Textural (Grey-Level Co-occurrence Matrix)	The Homogeneity within the ED in the T1 volume, considering 26-connected neighboring voxels in the 3D volume.
TEXTURE_GLCM_ED_T1_Correlation	Textural (Grey-Level Co-occurrence Matrix)	The Correlation within the ED in the T1 volume, considering 26-connected neighboring voxels in the 3D volume.
TEXTURE_GLCM_ED_T1_SumAverage	Textural (Grey-Level Co-occurrence Matrix)	The SumAverage within the ED in the T1 volume, considering 26-connected neighboring voxels in the 3D volume.
TEXTURE_GLCM_ED_T1_Variance	Textural (Grey-Level Co-occurrence Matrix)	The Variance within the ED in the T1 volume, considering 26-connected neighboring voxels in the 3D volume.
TEXTURE_GLCM_ED_T1_Dissimilarity	Textural (Grey-Level Co-occurrence Matrix)	The Dissimilarity within the ED in the T1 volume, considering 26-connected neighboring voxels in the 3D volume.
TEXTURE_GLCM_ED_T1_AutoCorrelation	Textural (Grey-Level Co-occurrence Matrix)	The Autocorrelation within the ED in the T1 volume, considering 26-connected neighboring voxels in the 3D volume.
TEXTURE_GLCM_ED_T2_Energy	Textural (Grey-Level Co-occurrence Matrix)	The Energy within the ED in the T2 volume, considering 26-connected neighboring voxels in the 3D volume.
TEXTURE_GLCM_ED_T2_Contrast	Textural (Grey-Level Co-occurrence Matrix)	The Contrast within the ED in the T2 volume, considering 26-connected neighboring voxels in the 3D volume.
TEXTURE_GLCM_ED_T2_Entropy	Textural (Grey-Level Co-occurrence Matrix)	The Entropy within the ED in the T2 volume, considering 26-connected neighboring voxels in the 3D volume.
TEXTURE_GLCM_ED_T2_Homogeneity	Textural (Grey-Level Co-occurrence Matrix)	The Homogeneity within the ED in the T2 volume, considering 26-connected neighboring voxels in the 3D volume.
TEXTURE_GLCM_ED_T2_Correlation	Textural (Grey-Level Co-occurrence Matrix)	The Correlation within the ED in the T2 volume, considering 26-connected neighboring voxels in the 3D volume.
TEXTURE_GLCM_ED_T2_SumAverage	Textural (Grey-Level Co-occurrence Matrix)	The SumAverage within the ED in the T2 volume, considering 26-connected neighboring voxels in the 3D volume.
TEXTURE_GLCM_ED_T2_Variance	Textural (Grey-Level Co-occurrence Matrix)	The Variance within the ED in the T2 volume, considering 26-connected neighboring voxels in the 3D volume.
TEXTURE_GLCM_ED_T2_Dissimilarity	Textural (Grey-Level Co-occurrence Matrix)	The Dissimilarity within the ED in the T2 volume, considering 26-connected neighboring voxels in the 3D volume.
TEXTURE_GLCM_ED_T2_AutoCorrelation	Textural (Grey-Level Co-occurrence Matrix)	The Autocorrelation within the ED in the T2 volume, considering 26-connected neighboring voxels in the 3D volume.
TEXTURE_GLCM_ED_FLAIR_Energy	Textural (Grey-Level Co-occurrence Matrix)	The Energy within the ED in the FLAIR volume, considering 26-connected neighboring voxels in the 3D volume.
TEXTURE_GLCM_ED_FLAIR_Contrast	Textural (Grey-Level Co-occurrence Matrix)	The Contrast within the ED in the FLAIR volume, considering 26-connected neighboring voxels in the 3D volume.
TEXTURE_GLCM_ED_FLAIR_Entropy	Textural (Grey-Level Co-occurrence Matrix)	The Entropy within the ED in the FLAIR volume, considering 26-connected neighboring voxels in the 3D volume.
TEXTURE_GLCM_ED_FLAIR_Homogeneity	Textural (Grey-Level Co-occurrence Matrix)	The Homogeneity within the ED in the FLAIR volume, considering 26-connected neighboring voxels in the 3D volume.
TEXTURE_GLCM_ED_FLAIR_Correlation	Textural (Grey-Level Co-occurrence Matrix)	The Correlation within the ED in the FLAIR volume, considering 26-connected neighboring voxels in the 3D volume.
TEXTURE_GLCM_ED_FLAIR_SumAverage	Textural (Grey-Level Co-occurrence Matrix)	The SumAverage within the ED in the FLAIR volume, considering 26-connected neighboring voxels in the 3D volume.
TEXTURE_GLCM_ED_FLAIR_Variance	Textural (Grey-Level Co-occurrence Matrix)	The Variance within the ED in the FLAIR volume, considering 26-connected neighboring voxels in the 3D volume.
TEXTURE_GLCM_ED_FLAIR_Dissimilarity	Textural (Grey-Level Co-occurrence Matrix)	The Dissimilarity within the ED in the FLAIR volume, considering 26-connected neighboring voxels in the 3D volume.
TEXTURE_GLCM_ED_FLAIR_AutoCorrelation	Textural (Grey-Level Co-occurrence Matrix)	The Autocorrelation within the ED in the FLAIR volume, considering 26-connected neighboring voxels in the 3D volume.
TEXTURE_GLCM_NET_T1Gd_Energy	Textural (Grey-Level Co-occurrence Matrix)	The Energy within the NET in the T1-Gd volume, considering 26-connected neighboring voxels in the 3D volume.
TEXTURE_GLCM_NET_T1Gd_Contrast	Textural (Grey-Level Co-occurrence Matrix)	The Contrast within the NET in the T1-Gd volume, considering 26-connected neighboring voxels in the 3D volume.
TEXTURE_GLCM_NET_T1Gd_Entropy	Textural (Grey-Level Co-occurrence Matrix)	The Entropy within the NET in the T1-Gd volume, considering 26-connected neighboring voxels in the 3D volume.
TEXTURE_GLCM_NET_T1Gd_Homogeneity	Textural (Grey-Level Co-occurrence Matrix)	The Homogeneity within the NET in the T1-Gd volume, considering 26-connected neighboring voxels in the 3D volume.
TEXTURE_GLCM_NET_T1Gd_Correlation	Textural (Grey-Level Co-occurrence Matrix)	The Correlation within the NET in the T1-Gd volume, considering 26-connected neighboring voxels in the 3D volume.
TEXTURE_GLCM_NET_T1Gd_SumAverage	Textural (Grey-Level Co-occurrence Matrix)	The SumAverage within the NET in the T1-Gd volume, considering 26-connected neighboring voxels in the 3D volume.
TEXTURE_GLCM_NET_T1Gd_Variance	Textural (Grey-Level Co-occurrence Matrix)	The Variance within the NET in the T1-Gd volume, considering 26-connected neighboring voxels in the 3D volume.
TEXTURE_GLCM_NET_T1Gd_Dissimilarity	Textural (Grey-Level Co-occurrence Matrix)	The Dissimilarity within the NET in the T1-Gd volume, considering 26-connected neighboring voxels in the 3D volume.
TEXTURE_GLCM_NET_T1Gd_AutoCorrelation	Textural (Grey-Level Co-occurrence Matrix)	The Autocorrelation within the NET in the T1-Gd volume, considering 26-connected neighboring voxels in the 3D volume.
TEXTURE_GLCM_NET_T1_Energy	Textural (Grey-Level Co-occurrence Matrix)	The Energy within the NET in the T1 volume, considering 26-connected neighboring voxels in the 3D volume.
TEXTURE_GLCM_NET_T1_Contrast	Textural (Grey-Level Co-occurrence Matrix)	The Contrast within the NET in the T1 volume, considering 26-connected neighboring voxels in the 3D volume.
TEXTURE_GLCM_NET_T1_Entropy	Textural (Grey-Level Co-occurrence Matrix)	The Entropy within the NET in the T1 volume, considering 26-connected neighboring voxels in the 3D volume.
TEXTURE_GLCM_NET_T1_Homogeneity	Textural (Grey-Level Co-occurrence Matrix)	The Homogeneity within the NET in the T1 volume, considering 26-connected neighboring voxels in the 3D volume.
TEXTURE_GLCM_NET_T1_Correlation	Textural (Grey-Level Co-occurrence Matrix)	The Correlation within the NET in the T1 volume, considering 26-connected neighboring voxels in the 3D volume.
TEXTURE_GLCM_NET_T1_SumAverage	Textural (Grey-Level Co-occurrence Matrix)	The SumAverage within the NET in the T1 volume, considering 26-connected neighboring voxels in the 3D volume.
TEXTURE_GLCM_NET_T1_Variance	Textural (Grey-Level Co-occurrence Matrix)	The Variance within the NET in the T1 volume, considering 26-connected neighboring voxels in the 3D volume.
TEXTURE_GLCM_NET_T1_Dissimilarity	Textural (Grey-Level Co-occurrence Matrix)	The Dissimilarity within the NET in the T1 volume, considering 26-connected neighboring voxels in the 3D volume.
TEXTURE_GLCM_NET_T1_AutoCorrelation	Textural (Grey-Level Co-occurrence Matrix)	The Autocorrelation within the NET in the T1 volume, considering 26-connected neighboring voxels in the 3D volume.
TEXTURE_GLCM_NET_T2_Energy	Textural (Grey-Level Co-occurrence Matrix)	The Energy within the NET in the T2 volume, considering 26-connected neighboring voxels in the 3D volume.
TEXTURE_GLCM_NET_T2_Contrast	Textural (Grey-Level Co-occurrence Matrix)	The Contrast within the NET in the T2 volume, considering 26-connected neighboring voxels in the 3D volume.
TEXTURE_GLCM_NET_T2_Entropy	Textural (Grey-Level Co-occurrence Matrix)	The Entropy within the NET in the T2 volume, considering 26-connected neighboring voxels in the 3D volume.
TEXTURE_GLCM_NET_T2_Homogeneity	Textural (Grey-Level Co-occurrence Matrix)	The Homogeneity within the NET in the T2 volume, considering 26-connected neighboring voxels in the 3D volume.
TEXTURE_GLCM_NET_T2_Correlation	Textural (Grey-Level Co-occurrence Matrix)	The Correlation within the NET in the T2 volume, considering 26-connected neighboring voxels in the 3D volume.
TEXTURE_GLCM_NET_T2_SumAverage	Textural (Grey-Level Co-occurrence Matrix)	The SumAverage within the NET in the T2 volume, considering 26-connected neighboring voxels in the 3D volume.
TEXTURE_GLCM_NET_T2_Variance	Textural (Grey-Level Co-occurrence Matrix)	The Variance within the NET in the T2 volume, considering 26-connected neighboring voxels in the 3D volume.
TEXTURE_GLCM_NET_T2_Dissimilarity	Textural (Grey-Level Co-occurrence Matrix)	The Dissimilarity within the NET in the T2 volume, considering 26-connected neighboring voxels in the 3D volume.
TEXTURE_GLCM_NET_T2_AutoCorrelation	Textural (Grey-Level Co-occurrence Matrix)	The Autocorrelation within the NET in the T2 volume, considering 26-connected neighboring voxels in the 3D volume.
TEXTURE_GLCM_NET_FLAIR_Energy	Textural (Grey-Level Co-occurrence Matrix)	The Energy within the NET in the FLAIR volume, considering 26-connected neighboring voxels in the 3D volume.
TEXTURE_GLCM_NET_FLAIR_Contrast	Textural (Grey-Level Co-occurrence Matrix)	The Contrast within the NET in the FLAIR volume, considering 26-connected neighboring voxels in the 3D volume.
TEXTURE_GLCM_NET_FLAIR_Entropy	Textural (Grey-Level Co-occurrence Matrix)	The Entropy within the NET in the FLAIR volume, considering 26-connected neighboring voxels in the 3D volume.
TEXTURE_GLCM_NET_FLAIR_Homogeneity	Textural (Grey-Level Co-occurrence Matrix)	The Homogeneity within the NET in the FLAIR volume, considering 26-connected neighboring voxels in the 3D volume.
TEXTURE_GLCM_NET_FLAIR_Correlation	Textural (Grey-Level Co-occurrence Matrix)	The Correlation within the NET in the FLAIR volume, considering 26-connected neighboring voxels in the 3D volume.
TEXTURE_GLCM_NET_FLAIR_SumAverage	Textural (Grey-Level Co-occurrence Matrix)	The SumAverage within the NET in the FLAIR volume, considering 26-connected neighboring voxels in the 3D volume.
TEXTURE_GLCM_NET_FLAIR_Variance	Textural (Grey-Level Co-occurrence Matrix)	The Variance within the NET in the FLAIR volume, considering 26-connected neighboring voxels in the 3D volume.
TEXTURE_GLCM_NET_FLAIR_Dissimilarity	Textural (Grey-Level Co-occurrence Matrix)	The Dissimilarity within the NET in the FLAIR volume, considering 26-connected neighboring voxels in the 3D volume.
TEXTURE_GLCM_NET_FLAIR_AutoCorrelation	Textural (Grey-Level Co-occurrence Matrix)	The Autocorrelation within the NET in the FLAIR volume, considering 26-connected neighboring voxels in the 3D volume.
TEXTURE_GLRLM_ET_T1Gd_SRE	Textural (Gray-Level Run-Length Matrix)	The "Short Run Emphasis" within the ET in the T1-Gd volume, considering the 13 main directions in the 3D volume.
TEXTURE_GLRLM_ET_T1Gd_LRE	Textural (Gray-Level Run-Length Matrix)	The "Long Run Emphasis" within the ET in the T1-Gd volume, considering the 13 main directions in the 3D volume.
TEXTURE_GLRLM_ET_T1Gd_GLN	Textural (Gray-Level Run-Length Matrix)	The "Gray-Level Nonuniformity" within the ET in the T1-Gd volume, considering the 13 main directions in the 3D volume.
TEXTURE_GLRLM_ET_T1Gd_RLN	Textural (Gray-Level Run-Length Matrix)	The "Run-Length Nonuniformity" within the ET in the T1-Gd volume, considering the 13 main directions in the 3D volume.
TEXTURE_GLRLM_ET_T1Gd_RP	Textural (Gray-Level Run-Length Matrix)	The "Run Percentage" within the ET in the T1-Gd volume, considering the 13 main directions in the 3D volume.
TEXTURE_GLRLM_ET_T1Gd_LGRE	Textural (Gray-Level Run-Length Matrix)	The "Low Gray-Level Run Emphasis" within the ET in the T1-Gd volume, considering the 13 main directions in the 3D volume.
TEXTURE_GLRLM_ET_T1Gd_HGRE	Textural (Gray-Level Run-Length Matrix)	The "High Gray-Level Run Emphasis" within the ET in the T1-Gd volume, considering the 13 main directions in the 3D volume.
TEXTURE_GLRLM_ET_T1Gd_SRLGE	Textural (Gray-Level Run-Length Matrix)	The "Short Run Low Gray-Level Emphasis" within the ET in the T1-Gd volume, considering the 13 main directions in the 3D volume.
TEXTURE_GLRLM_ET_T1Gd_SRHGE	Textural (Gray-Level Run-Length Matrix)	The "Short Run High Gray-Level Emphasis" within the ET in the T1-Gd volume, considering the 13 main directions in the 3D volume.
TEXTURE_GLRLM_ET_T1Gd_LRLGE	Textural (Gray-Level Run-Length Matrix)	The "Long Run Low Gray-Level Emphasis" within the ET in the T1-Gd volume, considering the 13 main directions in the 3D volume.
TEXTURE_GLRLM_ET_T1Gd_LRHGE	Textural (Gray-Level Run-Length Matrix)	The "Long Run High Gray-Level Emphasis" within the ET in the T1-Gd volume, considering the 13 main directions in the 3D volume.
TEXTURE_GLRLM_ET_T1Gd_GLV	Textural (Gray-Level Run-Length Matrix)	The "Gray-Level Variance" within the ET in the T1-Gd volume, considering the 13 main directions in the 3D volume.
TEXTURE_GLRLM_ET_T1Gd_RLV	Textural (Gray-Level Run-Length Matrix)	The "Run-Length Variance" within the ET in the T1-Gd volume, considering the 13 main directions in the 3D volume.
TEXTURE_GLRLM_ET_T1_SRE	Textural (Gray-Level Run-Length Matrix)	The "Short Run Emphasis" within the ET in the T1 volume, considering the 13 main directions in the 3D volume.
TEXTURE_GLRLM_ET_T1_LRE	Textural (Gray-Level Run-Length Matrix)	The "Long Run Emphasis" within the ET in the T1 volume, considering the 13 main directions in the 3D volume.
TEXTURE_GLRLM_ET_T1_GLN	Textural (Gray-Level Run-Length Matrix)	The "Gray-Level Nonuniformity" within the ET in the T1 volume, considering the 13 main directions in the 3D volume.
TEXTURE_GLRLM_ET_T1_RLN	Textural (Gray-Level Run-Length Matrix)	The "Run-Length Nonuniformity" within the ET in the T1 volume, considering the 13 main directions in the 3D volume.
TEXTURE_GLRLM_ET_T1_RP	Textural (Gray-Level Run-Length Matrix)	The "Run Percentage" within the ET in the T1 volume, considering the 13 main directions in the 3D volume.
TEXTURE_GLRLM_ET_T1_LGRE	Textural (Gray-Level Run-Length Matrix)	The "Low Gray-Level Run Emphasis" within the ET in the T1 volume, considering the 13 main directions in the 3D volume.
TEXTURE_GLRLM_ET_T1_HGRE	Textural (Gray-Level Run-Length Matrix)	The "High Gray-Level Run Emphasis" within the ET in the T1 volume, considering the 13 main directions in the 3D volume.
TEXTURE_GLRLM_ET_T1_SRLGE	Textural (Gray-Level Run-Length Matrix)	The "Short Run Low Gray-Level Emphasis" within the ET in the T1 volume, considering the 13 main directions in the 3D volume.
TEXTURE_GLRLM_ET_T1_SRHGE	Textural (Gray-Level Run-Length Matrix)	The "Short Run High Gray-Level Emphasis" within the ET in the T1 volume, considering the 13 main directions in the 3D volume.
TEXTURE_GLRLM_ET_T1_LRLGE	Textural (Gray-Level Run-Length Matrix)	The "Long Run Low Gray-Level Emphasis" within the ET in the T1 volume, considering the 13 main directions in the 3D volume.
TEXTURE_GLRLM_ET_T1_LRHGE	Textural (Gray-Level Run-Length Matrix)	The "Long Run High Gray-Level Emphasis" within the ET in the T1 volume, considering the 13 main directions in the 3D volume.
TEXTURE_GLRLM_ET_T1_GLV	Textural (Gray-Level Run-Length Matrix)	The "Gray-Level Variance" within the ET in the T1 volume, considering the 13 main directions in the 3D volume.
TEXTURE_GLRLM_ET_T1_RLV	Textural (Gray-Level Run-Length Matrix)	The "Run-Length Variance" within the ET in the T1 volume, considering the 13 main directions in the 3D volume.
TEXTURE_GLRLM_ET_T2_SRE	Textural (Gray-Level Run-Length Matrix)	The "Short Run Emphasis" within the ET in the T2 volume, considering the 13 main directions in the 3D volume.
TEXTURE_GLRLM_ET_T2_LRE	Textural (Gray-Level Run-Length Matrix)	The "Long Run Emphasis" within the ET in the T2 volume, considering the 13 main directions in the 3D volume.
TEXTURE_GLRLM_ET_T2_GLN	Textural (Gray-Level Run-Length Matrix)	The "Gray-Level Nonuniformity" within the ET in the T2 volume, considering the 13 main directions in the 3D volume.
TEXTURE_GLRLM_ET_T2_RLN	Textural (Gray-Level Run-Length Matrix)	The "Run-Length Nonuniformity" within the ET in the T2 volume, considering the 13 main directions in the 3D volume.
TEXTURE_GLRLM_ET_T2_RP	Textural (Gray-Level Run-Length Matrix)	The "Run Percentage" within the ET in the T2 volume, considering the 13 main directions in the 3D volume.
TEXTURE_GLRLM_ET_T2_LGRE	Textural (Gray-Level Run-Length Matrix)	The "Low Gray-Level Run Emphasis" within the ET in the T2 volume, considering the 13 main directions in the 3D volume.
TEXTURE_GLRLM_ET_T2_HGRE	Textural (Gray-Level Run-Length Matrix)	The "High Gray-Level Run Emphasis" within the ET in the T2 volume, considering the 13 main directions in the 3D volume.
TEXTURE_GLRLM_ET_T2_SRLGE	Textural (Gray-Level Run-Length Matrix)	The "Short Run Low Gray-Level Emphasis" within the ET in the T2 volume, considering the 13 main directions in the 3D volume.
TEXTURE_GLRLM_ET_T2_SRHGE	Textural (Gray-Level Run-Length Matrix)	The "Short Run High Gray-Level Emphasis" within the ET in the T2 volume, considering the 13 main directions in the 3D volume.
TEXTURE_GLRLM_ET_T2_LRLGE	Textural (Gray-Level Run-Length Matrix)	The "Long Run Low Gray-Level Emphasis" within the ET in the T2 volume, considering the 13 main directions in the 3D volume.
TEXTURE_GLRLM_ET_T2_LRHGE	Textural (Gray-Level Run-Length Matrix)	The "Long Run High Gray-Level Emphasis" within the ET in the T2 volume, considering the 13 main directions in the 3D volume.
TEXTURE_GLRLM_ET_T2_GLV	Textural (Gray-Level Run-Length Matrix)	The "Gray-Level Variance" within the ET in the T2 volume, considering the 13 main directions in the 3D volume.
TEXTURE_GLRLM_ET_T2_RLV	Textural (Gray-Level Run-Length Matrix)	The "Run-Length Variance" within the ET in the T2 volume, considering the 13 main directions in the 3D volume.
TEXTURE_GLRLM_ET_FLAIR_SRE	Textural (Gray-Level Run-Length Matrix)	The "Short Run Emphasis" within the ET in the FLAIR volume, considering the 13 main directions in the 3D volume.
TEXTURE_GLRLM_ET_FLAIR_LRE	Textural (Gray-Level Run-Length Matrix)	The "Long Run Emphasis" within the ET in the FLAIR volume, considering the 13 main directions in the 3D volume.
TEXTURE_GLRLM_ET_FLAIR_GLN	Textural (Gray-Level Run-Length Matrix)	The "Gray-Level Nonuniformity" within the ET in the FLAIR volume, considering the 13 main directions in the 3D volume.
TEXTURE_GLRLM_ET_FLAIR_RLN	Textural (Gray-Level Run-Length Matrix)	The "Run-Length Nonuniformity" within the ET in the FLAIR volume, considering the 13 main directions in the 3D volume.
TEXTURE_GLRLM_ET_FLAIR_RP	Textural (Gray-Level Run-Length Matrix)	The "Run Percentage" within the ET in the FLAIR volume, considering the 13 main directions in the 3D volume.
TEXTURE_GLRLM_ET_FLAIR_LGRE	Textural (Gray-Level Run-Length Matrix)	The "Low Gray-Level Run Emphasis" within the ET in the FLAIR volume, considering the 13 main directions in the 3D volume.
TEXTURE_GLRLM_ET_FLAIR_HGRE	Textural (Gray-Level Run-Length Matrix)	The "High Gray-Level Run Emphasis" within the ET in the FLAIR volume, considering the 13 main directions in the 3D volume.
TEXTURE_GLRLM_ET_FLAIR_SRLGE	Textural (Gray-Level Run-Length Matrix)	The "Short Run Low Gray-Level Emphasis" within the ET in the FLAIR volume, considering the 13 main directions in the 3D volume.
TEXTURE_GLRLM_ET_FLAIR_SRHGE	Textural (Gray-Level Run-Length Matrix)	The "Short Run High Gray-Level Emphasis" within the ET in the FLAIR volume, considering the 13 main directions in the 3D volume.
TEXTURE_GLRLM_ET_FLAIR_LRLGE	Textural (Gray-Level Run-Length Matrix)	The "Long Run Low Gray-Level Emphasis" within the ET in the FLAIR volume, considering the 13 main directions in the 3D volume.
TEXTURE_GLRLM_ET_FLAIR_LRHGE	Textural (Gray-Level Run-Length Matrix)	The "Long Run High Gray-Level Emphasis" within the ET in the FLAIR volume, considering the 13 main directions in the 3D volume.
TEXTURE_GLRLM_ET_FLAIR_GLV	Textural (Gray-Level Run-Length Matrix)	The "Gray-Level Variance" within the ET in the FLAIR volume, considering the 13 main directions in the 3D volume.
TEXTURE_GLRLM_ET_FLAIR_RLV	Textural (Gray-Level Run-Length Matrix)	The "Run-Length Variance" within the ET in the FLAIR volume, considering the 13 main directions in the 3D volume.
TEXTURE_GLRLM_ED_T1Gd_SRE	Textural (Gray-Level Run-Length Matrix)	The "Short Run Emphasis" within the ED in the T1-Gd volume, considering the 13 main directions in the 3D volume.
TEXTURE_GLRLM_ED_T1Gd_LRE	Textural (Gray-Level Run-Length Matrix)	The "Long Run Emphasis" within the ED in the T1-Gd volume, considering the 13 main directions in the 3D volume.
TEXTURE_GLRLM_ED_T1Gd_GLN	Textural (Gray-Level Run-Length Matrix)	The "Gray-Level Nonuniformity" within the ED in the T1-Gd volume, considering the 13 main directions in the 3D volume.
TEXTURE_GLRLM_ED_T1Gd_RLN	Textural (Gray-Level Run-Length Matrix)	The "Run-Length Nonuniformity" within the ED in the T1-Gd volume, considering the 13 main directions in the 3D volume.
TEXTURE_GLRLM_ED_T1Gd_RP	Textural (Gray-Level Run-Length Matrix)	The "Run Percentage" within the ED in the T1-Gd volume, considering the 13 main directions in the 3D volume.
TEXTURE_GLRLM_ED_T1Gd_LGRE	Textural (Gray-Level Run-Length Matrix)	The "Low Gray-Level Run Emphasis" within the ED in the T1-Gd volume, considering the 13 main directions in the 3D volume.
TEXTURE_GLRLM_ED_T1Gd_HGRE	Textural (Gray-Level Run-Length Matrix)	The "High Gray-Level Run Emphasis" within the ED in the T1-Gd volume, considering the 13 main directions in the 3D volume.
TEXTURE_GLRLM_ED_T1Gd_SRLGE	Textural (Gray-Level Run-Length Matrix)	The "Short Run Low Gray-Level Emphasis" within the ED in the T1-Gd volume, considering the 13 main directions in the 3D volume.
TEXTURE_GLRLM_ED_T1Gd_SRHGE	Textural (Gray-Level Run-Length Matrix)	The "Short Run High Gray-Level Emphasis" within the ED in the T1-Gd volume, considering the 13 main directions in the 3D volume.
TEXTURE_GLRLM_ED_T1Gd_LRLGE	Textural (Gray-Level Run-Length Matrix)	The "Long Run Low Gray-Level Emphasis" within the ED in the T1-Gd volume, considering the 13 main directions in the 3D volume.
TEXTURE_GLRLM_ED_T1Gd_LRHGE	Textural (Gray-Level Run-Length Matrix)	The "Long Run High Gray-Level Emphasis" within the ED in the T1-Gd volume, considering the 13 main directions in the 3D volume.
TEXTURE_GLRLM_ED_T1Gd_GLV	Textural (Gray-Level Run-Length Matrix)	The "Gray-Level Variance" within the ED in the T1-Gd volume, considering the 13 main directions in the 3D volume.
TEXTURE_GLRLM_ED_T1Gd_RLV	Textural (Gray-Level Run-Length Matrix)	The "Run-Length Variance" within the ED in the T1-Gd volume, considering the 13 main directions in the 3D volume.
TEXTURE_GLRLM_ED_T1_SRE	Textural (Gray-Level Run-Length Matrix)	The "Short Run Emphasis" within the ED in the T1 volume, considering the 13 main directions in the 3D volume.
TEXTURE_GLRLM_ED_T1_LRE	Textural (Gray-Level Run-Length Matrix)	The "Long Run Emphasis" within the ED in the T1 volume, considering the 13 main directions in the 3D volume.
TEXTURE_GLRLM_ED_T1_GLN	Textural (Gray-Level Run-Length Matrix)	The "Gray-Level Nonuniformity" within the ED in the T1 volume, considering the 13 main directions in the 3D volume.
TEXTURE_GLRLM_ED_T1_RLN	Textural (Gray-Level Run-Length Matrix)	The "Run-Length Nonuniformity" within the ED in the T1 volume, considering the 13 main directions in the 3D volume.
TEXTURE_GLRLM_ED_T1_RP	Textural (Gray-Level Run-Length Matrix)	The "Run Percentage" within the ED in the T1 volume, considering the 13 main directions in the 3D volume.
TEXTURE_GLRLM_ED_T1_LGRE	Textural (Gray-Level Run-Length Matrix)	The "Low Gray-Level Run Emphasis" within the ED in the T1 volume, considering the 13 main directions in the 3D volume.
TEXTURE_GLRLM_ED_T1_HGRE	Textural (Gray-Level Run-Length Matrix)	The "High Gray-Level Run Emphasis" within the ED in the T1 volume, considering the 13 main directions in the 3D volume.
TEXTURE_GLRLM_ED_T1_SRLGE	Textural (Gray-Level Run-Length Matrix)	The "Short Run Low Gray-Level Emphasis" within the ED in the T1 volume, considering the 13 main directions in the 3D volume.
TEXTURE_GLRLM_ED_T1_SRHGE	Textural (Gray-Level Run-Length Matrix)	The "Short Run High Gray-Level Emphasis" within the ED in the T1 volume, considering the 13 main directions in the 3D volume.
TEXTURE_GLRLM_ED_T1_LRLGE	Textural (Gray-Level Run-Length Matrix)	The "Long Run Low Gray-Level Emphasis" within the ED in the T1 volume, considering the 13 main directions in the 3D volume.
TEXTURE_GLRLM_ED_T1_LRHGE	Textural (Gray-Level Run-Length Matrix)	The "Long Run High Gray-Level Emphasis" within the ED in the T1 volume, considering the 13 main directions in the 3D volume.
TEXTURE_GLRLM_ED_T1_GLV	Textural (Gray-Level Run-Length Matrix)	The "Gray-Level Variance" within the ED in the T1 volume, considering the 13 main directions in the 3D volume.
TEXTURE_GLRLM_ED_T1_RLV	Textural (Gray-Level Run-Length Matrix)	The "Run-Length Variance" within the ED in the T1 volume, considering the 13 main directions in the 3D volume.
TEXTURE_GLRLM_ED_T2_SRE	Textural (Gray-Level Run-Length Matrix)	The "Short Run Emphasis" within the ED in the T2 volume, considering the 13 main directions in the 3D volume.
TEXTURE_GLRLM_ED_T2_LRE	Textural (Gray-Level Run-Length Matrix)	The "Long Run Emphasis" within the ED in the T2 volume, considering the 13 main directions in the 3D volume.
TEXTURE_GLRLM_ED_T2_GLN	Textural (Gray-Level Run-Length Matrix)	The "Gray-Level Nonuniformity" within the ED in the T2 volume, considering the 13 main directions in the 3D volume.
TEXTURE_GLRLM_ED_T2_RLN	Textural (Gray-Level Run-Length Matrix)	The "Run-Length Nonuniformity" within the ED in the T2 volume, considering the 13 main directions in the 3D volume.
TEXTURE_GLRLM_ED_T2_RP	Textural (Gray-Level Run-Length Matrix)	The "Run Percentage" within the ED in the T2 volume, considering the 13 main directions in the 3D volume.
TEXTURE_GLRLM_ED_T2_LGRE	Textural (Gray-Level Run-Length Matrix)	The "Low Gray-Level Run Emphasis" within the ED in the T2 volume, considering the 13 main directions in the 3D volume.
TEXTURE_GLRLM_ED_T2_HGRE	Textural (Gray-Level Run-Length Matrix)	The "High Gray-Level Run Emphasis" within the ED in the T2 volume, considering the 13 main directions in the 3D volume.
TEXTURE_GLRLM_ED_T2_SRLGE	Textural (Gray-Level Run-Length Matrix)	The "Short Run Low Gray-Level Emphasis" within the ED in the T2 volume, considering the 13 main directions in the 3D volume.
TEXTURE_GLRLM_ED_T2_SRHGE	Textural (Gray-Level Run-Length Matrix)	The "Short Run High Gray-Level Emphasis" within the ED in the T2 volume, considering the 13 main directions in the 3D volume.
TEXTURE_GLRLM_ED_T2_LRLGE	Textural (Gray-Level Run-Length Matrix)	The "Long Run Low Gray-Level Emphasis" within the ED in the T2 volume, considering the 13 main directions in the 3D volume.
TEXTURE_GLRLM_ED_T2_LRHGE	Textural (Gray-Level Run-Length Matrix)	The "Long Run High Gray-Level Emphasis" within the ED in the T2 volume, considering the 13 main directions in the 3D volume.
TEXTURE_GLRLM_ED_T2_GLV	Textural (Gray-Level Run-Length Matrix)	The "Gray-Level Variance" within the ED in the T2 volume, considering the 13 main directions in the 3D volume.
TEXTURE_GLRLM_ED_T2_RLV	Textural (Gray-Level Run-Length Matrix)	The "Run-Length Variance" within the ED in the T2 volume, considering the 13 main directions in the 3D volume.
TEXTURE_GLRLM_ED_FLAIR_SRE	Textural (Gray-Level Run-Length Matrix)	The "Short Run Emphasis" within the ED in the FLAIR volume, considering the 13 main directions in the 3D volume.
TEXTURE_GLRLM_ED_FLAIR_LRE	Textural (Gray-Level Run-Length Matrix)	The "Long Run Emphasis" within the ED in the FLAIR volume, considering the 13 main directions in the 3D volume.
TEXTURE_GLRLM_ED_FLAIR_GLN	Textural (Gray-Level Run-Length Matrix)	The "Gray-Level Nonuniformity" within the ED in the FLAIR volume, considering the 13 main directions in the 3D volume.
TEXTURE_GLRLM_ED_FLAIR_RLN	Textural (Gray-Level Run-Length Matrix)	The "Run-Length Nonuniformity" within the ED in the FLAIR volume, considering the 13 main directions in the 3D volume.
TEXTURE_GLRLM_ED_FLAIR_RP	Textural (Gray-Level Run-Length Matrix)	The "Run Percentage" within the ED in the FLAIR volume, considering the 13 main directions in the 3D volume.
TEXTURE_GLRLM_ED_FLAIR_LGRE	Textural (Gray-Level Run-Length Matrix)	The "Low Gray-Level Run Emphasis" within the ED in the FLAIR volume, considering the 13 main directions in the 3D volume.
TEXTURE_GLRLM_ED_FLAIR_HGRE	Textural (Gray-Level Run-Length Matrix)	The "High Gray-Level Run Emphasis" within the ED in the FLAIR volume, considering the 13 main directions in the 3D volume.
TEXTURE_GLRLM_ED_FLAIR_SRLGE	Textural (Gray-Level Run-Length Matrix)	The "Short Run Low Gray-Level Emphasis" within the ED in the FLAIR volume, considering the 13 main directions in the 3D volume.
TEXTURE_GLRLM_ED_FLAIR_SRHGE	Textural (Gray-Level Run-Length Matrix)	The "Short Run High Gray-Level Emphasis" within the ED in the FLAIR volume, considering the 13 main directions in the 3D volume.
TEXTURE_GLRLM_ED_FLAIR_LRLGE	Textural (Gray-Level Run-Length Matrix)	The "Long Run Low Gray-Level Emphasis" within the ED in the FLAIR volume, considering the 13 main directions in the 3D volume.
TEXTURE_GLRLM_ED_FLAIR_LRHGE	Textural (Gray-Level Run-Length Matrix)	The "Long Run High Gray-Level Emphasis" within the ED in the FLAIR volume, considering the 13 main directions in the 3D volume.
TEXTURE_GLRLM_ED_FLAIR_GLV	Textural (Gray-Level Run-Length Matrix)	The "Gray-Level Variance" within the ED in the FLAIR volume, considering the 13 main directions in the 3D volume.
TEXTURE_GLRLM_ED_FLAIR_RLV	Textural (Gray-Level Run-Length Matrix)	The "Run-Length Variance" within the ED in the FLAIR volume, considering the 13 main directions in the 3D volume.
TEXTURE_GLRLM_NET_T1Gd_SRE	Textural (Gray-Level Run-Length Matrix)	The "Short Run Emphasis" within the NET in the T1-Gd volume, considering the 13 main directions in the 3D volume.
TEXTURE_GLRLM_NET_T1Gd_LRE	Textural (Gray-Level Run-Length Matrix)	The "Long Run Emphasis" within the NET in the T1-Gd volume, considering the 13 main directions in the 3D volume.
TEXTURE_GLRLM_NET_T1Gd_GLN	Textural (Gray-Level Run-Length Matrix)	The "Gray-Level Nonuniformity" within the NET in the T1-Gd volume, considering the 13 main directions in the 3D volume.
TEXTURE_GLRLM_NET_T1Gd_RLN	Textural (Gray-Level Run-Length Matrix)	The "Run-Length Nonuniformity" within the NET in the T1-Gd volume, considering the 13 main directions in the 3D volume.
TEXTURE_GLRLM_NET_T1Gd_RP	Textural (Gray-Level Run-Length Matrix)	The "Run Percentage" within the NET in the T1-Gd volume, considering the 13 main directions in the 3D volume.
TEXTURE_GLRLM_NET_T1Gd_LGRE	Textural (Gray-Level Run-Length Matrix)	The "Low Gray-Level Run Emphasis" within the NET in the T1-Gd volume, considering the 13 main directions in the 3D volume.
TEXTURE_GLRLM_NET_T1Gd_HGRE	Textural (Gray-Level Run-Length Matrix)	The "High Gray-Level Run Emphasis" within the NET in the T1-Gd volume, considering the 13 main directions in the 3D volume.
TEXTURE_GLRLM_NET_T1Gd_SRLGE	Textural (Gray-Level Run-Length Matrix)	The "Short Run Low Gray-Level Emphasis" within the NET in the T1-Gd volume, considering the 13 main directions in the 3D volume.
TEXTURE_GLRLM_NET_T1Gd_SRHGE	Textural (Gray-Level Run-Length Matrix)	The "Short Run High Gray-Level Emphasis" within the NET in the T1-Gd volume, considering the 13 main directions in the 3D volume.
TEXTURE_GLRLM_NET_T1Gd_LRLGE	Textural (Gray-Level Run-Length Matrix)	The "Long Run Low Gray-Level Emphasis" within the NET in the T1-Gd volume, considering the 13 main directions in the 3D volume.
TEXTURE_GLRLM_NET_T1Gd_LRHGE	Textural (Gray-Level Run-Length Matrix)	The "Long Run High Gray-Level Emphasis" within the NET in the T1-Gd volume, considering the 13 main directions in the 3D volume.
TEXTURE_GLRLM_NET_T1Gd_GLV	Textural (Gray-Level Run-Length Matrix)	The "Gray-Level Variance" within the NET in the T1-Gd volume, considering the 13 main directions in the 3D volume.
TEXTURE_GLRLM_NET_T1Gd_RLV	Textural (Gray-Level Run-Length Matrix)	The "Run-Length Variance" within the NET in the T1-Gd volume, considering the 13 main directions in the 3D volume.
TEXTURE_GLRLM_NET_T1_SRE	Textural (Gray-Level Run-Length Matrix)	The "Short Run Emphasis" within the NET in the T1 volume, considering the 13 main directions in the 3D volume.
TEXTURE_GLRLM_NET_T1_LRE	Textural (Gray-Level Run-Length Matrix)	The "Long Run Emphasis" within the NET in the T1 volume, considering the 13 main directions in the 3D volume.
TEXTURE_GLRLM_NET_T1_GLN	Textural (Gray-Level Run-Length Matrix)	The "Gray-Level Nonuniformity" within the NET in the T1 volume, considering the 13 main directions in the 3D volume.
TEXTURE_GLRLM_NET_T1_RLN	Textural (Gray-Level Run-Length Matrix)	The "Run-Length Nonuniformity" within the NET in the T1 volume, considering the 13 main directions in the 3D volume.
TEXTURE_GLRLM_NET_T1_RP	Textural (Gray-Level Run-Length Matrix)	The "Run Percentage" within the NET in the T1 volume, considering the 13 main directions in the 3D volume.
TEXTURE_GLRLM_NET_T1_LGRE	Textural (Gray-Level Run-Length Matrix)	The "Low Gray-Level Run Emphasis" within the NET in the T1 volume, considering the 13 main directions in the 3D volume.
TEXTURE_GLRLM_NET_T1_HGRE	Textural (Gray-Level Run-Length Matrix)	The "High Gray-Level Run Emphasis" within the NET in the T1 volume, considering the 13 main directions in the 3D volume.
TEXTURE_GLRLM_NET_T1_SRLGE	Textural (Gray-Level Run-Length Matrix)	The "Short Run Low Gray-Level Emphasis" within the NET in the T1 volume, considering the 13 main directions in the 3D volume.
TEXTURE_GLRLM_NET_T1_SRHGE	Textural (Gray-Level Run-Length Matrix)	The "Short Run High Gray-Level Emphasis" within the NET in the T1 volume, considering the 13 main directions in the 3D volume.
TEXTURE_GLRLM_NET_T1_LRLGE	Textural (Gray-Level Run-Length Matrix)	The "Long Run Low Gray-Level Emphasis" within the NET in the T1 volume, considering the 13 main directions in the 3D volume.
TEXTURE_GLRLM_NET_T1_LRHGE	Textural (Gray-Level Run-Length Matrix)	The "Long Run High Gray-Level Emphasis" within the NET in the T1 volume, considering the 13 main directions in the 3D volume.
TEXTURE_GLRLM_NET_T1_GLV	Textural (Gray-Level Run-Length Matrix)	The "Gray-Level Variance" within the NET in the T1 volume, considering the 13 main directions in the 3D volume.
TEXTURE_GLRLM_NET_T1_RLV	Textural (Gray-Level Run-Length Matrix)	The "Run-Length Variance" within the NET in the T1 volume, considering the 13 main directions in the 3D volume.
TEXTURE_GLRLM_NET_T2_SRE	Textural (Gray-Level Run-Length Matrix)	The "Short Run Emphasis" within the NET in the T2 volume, considering the 13 main directions in the 3D volume.
TEXTURE_GLRLM_NET_T2_LRE	Textural (Gray-Level Run-Length Matrix)	The "Long Run Emphasis" within the NET in the T2 volume, considering the 13 main directions in the 3D volume.
TEXTURE_GLRLM_NET_T2_GLN	Textural (Gray-Level Run-Length Matrix)	The "Gray-Level Nonuniformity" within the NET in the T2 volume, considering the 13 main directions in the 3D volume.
TEXTURE_GLRLM_NET_T2_RLN	Textural (Gray-Level Run-Length Matrix)	The "Run-Length Nonuniformity" within the NET in the T2 volume, considering the 13 main directions in the 3D volume.
TEXTURE_GLRLM_NET_T2_RP	Textural (Gray-Level Run-Length Matrix)	The "Run Percentage" within the NET in the T2 volume, considering the 13 main directions in the 3D volume.
TEXTURE_GLRLM_NET_T2_LGRE	Textural (Gray-Level Run-Length Matrix)	The "Low Gray-Level Run Emphasis" within the NET in the T2 volume, considering the 13 main directions in the 3D volume.
TEXTURE_GLRLM_NET_T2_HGRE	Textural (Gray-Level Run-Length Matrix)	The "High Gray-Level Run Emphasis" within the NET in the T2 volume, considering the 13 main directions in the 3D volume.
TEXTURE_GLRLM_NET_T2_SRLGE	Textural (Gray-Level Run-Length Matrix)	The "Short Run Low Gray-Level Emphasis" within the NET in the T2 volume, considering the 13 main directions in the 3D volume.
TEXTURE_GLRLM_NET_T2_SRHGE	Textural (Gray-Level Run-Length Matrix)	The "Short Run High Gray-Level Emphasis" within the NET in the T2 volume, considering the 13 main directions in the 3D volume.
TEXTURE_GLRLM_NET_T2_LRLGE	Textural (Gray-Level Run-Length Matrix)	The "Long Run Low Gray-Level Emphasis" within the NET in the T2 volume, considering the 13 main directions in the 3D volume.
TEXTURE_GLRLM_NET_T2_LRHGE	Textural (Gray-Level Run-Length Matrix)	The "Long Run High Gray-Level Emphasis" within the NET in the T2 volume, considering the 13 main directions in the 3D volume.
TEXTURE_GLRLM_NET_T2_GLV	Textural (Gray-Level Run-Length Matrix)	The "Gray-Level Variance" within the NET in the T2 volume, considering the 13 main directions in the 3D volume.
TEXTURE_GLRLM_NET_T2_RLV	Textural (Gray-Level Run-Length Matrix)	The "Run-Length Variance" within the NET in the T2 volume, considering the 13 main directions in the 3D volume.
TEXTURE_GLRLM_NET_FLAIR_SRE	Textural (Gray-Level Run-Length Matrix)	The "Short Run Emphasis" within the NET in the FLAIR volume, considering the 13 main directions in the 3D volume.
TEXTURE_GLRLM_NET_FLAIR_LRE	Textural (Gray-Level Run-Length Matrix)	The "Long Run Emphasis" within the NET in the FLAIR volume, considering the 13 main directions in the 3D volume.
TEXTURE_GLRLM_NET_FLAIR_GLN	Textural (Gray-Level Run-Length Matrix)	The "Gray-Level Nonuniformity" within the NET in the FLAIR volume, considering the 13 main directions in the 3D volume.
TEXTURE_GLRLM_NET_FLAIR_RLN	Textural (Gray-Level Run-Length Matrix)	The "Run-Length Nonuniformity" within the NET in the FLAIR volume, considering the 13 main directions in the 3D volume.
TEXTURE_GLRLM_NET_FLAIR_RP	Textural (Gray-Level Run-Length Matrix)	The "Run Percentage" within the NET in the FLAIR volume, considering the 13 main directions in the 3D volume.
TEXTURE_GLRLM_NET_FLAIR_LGRE	Textural (Gray-Level Run-Length Matrix)	The "Low Gray-Level Run Emphasis" within the NET in the FLAIR volume, considering the 13 main directions in the 3D volume.
TEXTURE_GLRLM_NET_FLAIR_HGRE	Textural (Gray-Level Run-Length Matrix)	The "High Gray-Level Run Emphasis" within the NET in the FLAIR volume, considering the 13 main directions in the 3D volume.
TEXTURE_GLRLM_NET_FLAIR_SRLGE	Textural (Gray-Level Run-Length Matrix)	The "Short Run Low Gray-Level Emphasis" within the NET in the FLAIR volume, considering the 13 main directions in the 3D volume.
TEXTURE_GLRLM_NET_FLAIR_SRHGE	Textural (Gray-Level Run-Length Matrix)	The "Short Run High Gray-Level Emphasis" within the NET in the FLAIR volume, considering the 13 main directions in the 3D volume.
TEXTURE_GLRLM_NET_FLAIR_LRLGE	Textural (Gray-Level Run-Length Matrix)	The "Long Run Low Gray-Level Emphasis" within the NET in the FLAIR volume, considering the 13 main directions in the 3D volume.
TEXTURE_GLRLM_NET_FLAIR_LRHGE	Textural (Gray-Level Run-Length Matrix)	The "Long Run High Gray-Level Emphasis" within the NET in the FLAIR volume, considering the 13 main directions in the 3D volume.
TEXTURE_GLRLM_NET_FLAIR_GLV	Textural (Gray-Level Run-Length Matrix)	The "Gray-Level Variance" within the NET in the FLAIR volume, considering the 13 main directions in the 3D volume.
TEXTURE_GLRLM_NET_FLAIR_RLV	Textural (Gray-Level Run-Length Matrix)	The "Run-Length Variance" within the NET in the FLAIR volume, considering the 13 main directions in the 3D volume.
TEXTURE_GLSZM_ET_T1Gd_SZE	Textural (Gray-Level Size Zone Matrix)	The "Small Zone Emphasis" within the ET in the T1-Gd volume, whilst zones of different sizes are computed using a 26-voxel connectivity.
TEXTURE_GLSZM_ET_T1Gd_LZE	Textural (Gray-Level Size Zone Matrix)	The "Large Zone Emphasis" within the ET in the T1-Gd volume, whilst zones of different sizes are computed using a 26-voxel connectivity.
TEXTURE_GLSZM_ET_T1Gd_GLN	Textural (Gray-Level Size Zone Matrix)	The "Gray-Level Nonuniformity" within the ET in the T1-Gd volume, whilst zones of different sizes are computed using a 26-voxel connectivity.
TEXTURE_GLSZM_ET_T1Gd_ZSN	Textural (Gray-Level Size Zone Matrix)	The "Zone-Size Nonuniformity" within the ET in the T1-Gd volume, whilst zones of different sizes are computed using a 26-voxel connectivity.
TEXTURE_GLSZM_ET_T1Gd_ZP	Textural (Gray-Level Size Zone Matrix)	The "Zone Percentage" within the ET in the T1-Gd volume, whilst zones of different sizes are computed using a 26-voxel connectivity.
TEXTURE_GLSZM_ET_T1Gd_LGZE	Textural (Gray-Level Size Zone Matrix)	The "Low Gray-Level Zone Emphasis" within the ET in the T1-Gd volume, whilst zones of different sizes are computed using a 26-voxel connectivity.
TEXTURE_GLSZM_ET_T1Gd_HGZE	Textural (Gray-Level Size Zone Matrix)	The "High Gray-Level Zone Emphasis" within the ET in the T1-Gd volume, whilst zones of different sizes are computed using a 26-voxel connectivity.
TEXTURE_GLSZM_ET_T1Gd_SZLGE	Textural (Gray-Level Size Zone Matrix)	The "Small Zone Low Gray-Level Emphasis" within the ET in the T1-Gd volume, whilst zones of different sizes are computed using a 26-voxel connectivity.
TEXTURE_GLSZM_ET_T1Gd_SZHGE	Textural (Gray-Level Size Zone Matrix)	The "Small Zone High Gray-Level Emphasis" within the ET in the T1-Gd volume, whilst zones of different sizes are computed using a 26-voxel connectivity.
TEXTURE_GLSZM_ET_T1Gd_LZLGE	Textural (Gray-Level Size Zone Matrix)	The "Large Zone Low Gray-Level Emphasis" within the ET in the T1-Gd volume, whilst zones of different sizes are computed using a 26-voxel connectivity.
TEXTURE_GLSZM_ET_T1Gd_LZHGE	Textural (Gray-Level Size Zone Matrix)	The "Large Zone High Gray-Level Emphasis" within the ET in the T1-Gd volume, whilst zones of different sizes are computed using a 26-voxel connectivity.
TEXTURE_GLSZM_ET_T1Gd_GLV	Textural (Gray-Level Size Zone Matrix)	The "Gray-Level Variance" within the ET in the T1-Gd volume, whilst zones of different sizes are computed using a 26-voxel connectivity.
TEXTURE_GLSZM_ET_T1Gd_ZSV	Textural (Gray-Level Size Zone Matrix)	The "Zone-Size Variance" within the ET in the T1-Gd volume, whilst zones of different sizes are computed using a 26-voxel connectivity.
TEXTURE_GLSZM_ET_T1_SZE	Textural (Gray-Level Size Zone Matrix)	The "Small Zone Emphasis" within the ET in the T1 volume, whilst zones of different sizes are computed using a 26-voxel connectivity.
TEXTURE_GLSZM_ET_T1_LZE	Textural (Gray-Level Size Zone Matrix)	The "Large Zone Emphasis" within the ET in the T1 volume, whilst zones of different sizes are computed using a 26-voxel connectivity.
TEXTURE_GLSZM_ET_T1_GLN	Textural (Gray-Level Size Zone Matrix)	The "Gray-Level Nonuniformity" within the ET in the T1 volume, whilst zones of different sizes are computed using a 26-voxel connectivity.
TEXTURE_GLSZM_ET_T1_ZSN	Textural (Gray-Level Size Zone Matrix)	The "Zone-Size Nonuniformity" within the ET in the T1 volume, whilst zones of different sizes are computed using a 26-voxel connectivity.
TEXTURE_GLSZM_ET_T1_ZP	Textural (Gray-Level Size Zone Matrix)	The "Zone Percentage" within the ET in the T1 volume, whilst zones of different sizes are computed using a 26-voxel connectivity.
TEXTURE_GLSZM_ET_T1_LGZE	Textural (Gray-Level Size Zone Matrix)	The "Low Gray-Level Zone Emphasis" within the ET in the T1 volume, whilst zones of different sizes are computed using a 26-voxel connectivity.
TEXTURE_GLSZM_ET_T1_HGZE	Textural (Gray-Level Size Zone Matrix)	The "High Gray-Level Zone Emphasis" within the ET in the T1 volume, whilst zones of different sizes are computed using a 26-voxel connectivity.
TEXTURE_GLSZM_ET_T1_SZLGE	Textural (Gray-Level Size Zone Matrix)	The "Small Zone Low Gray-Level Emphasis" within the ET in the T1 volume, whilst zones of different sizes are computed using a 26-voxel connectivity.
TEXTURE_GLSZM_ET_T1_SZHGE	Textural (Gray-Level Size Zone Matrix)	The "Small Zone High Gray-Level Emphasis" within the ET in the T1 volume, whilst zones of different sizes are computed using a 26-voxel connectivity.
TEXTURE_GLSZM_ET_T1_LZLGE	Textural (Gray-Level Size Zone Matrix)	The "Large Zone Low Gray-Level Emphasis" within the ET in the T1 volume, whilst zones of different sizes are computed using a 26-voxel connectivity.
TEXTURE_GLSZM_ET_T1_LZHGE	Textural (Gray-Level Size Zone Matrix)	The "Large Zone High Gray-Level Emphasis" within the ET in the T1 volume, whilst zones of different sizes are computed using a 26-voxel connectivity.
TEXTURE_GLSZM_ET_T1_GLV	Textural (Gray-Level Size Zone Matrix)	The "Gray-Level Variance" within the ET in the T1 volume, whilst zones of different sizes are computed using a 26-voxel connectivity.
TEXTURE_GLSZM_ET_T1_ZSV	Textural (Gray-Level Size Zone Matrix)	The "Zone-Size Variance" within the ET in the T1 volume, whilst zones of different sizes are computed using a 26-voxel connectivity.
TEXTURE_GLSZM_ET_T2_SZE	Textural (Gray-Level Size Zone Matrix)	The "Small Zone Emphasis" within the ET in the T2 volume, whilst zones of different sizes are computed using a 26-voxel connectivity.
TEXTURE_GLSZM_ET_T2_LZE	Textural (Gray-Level Size Zone Matrix)	The "Large Zone Emphasis" within the ET in the T2 volume, whilst zones of different sizes are computed using a 26-voxel connectivity.
TEXTURE_GLSZM_ET_T2_GLN	Textural (Gray-Level Size Zone Matrix)	The "Gray-Level Nonuniformity" within the ET in the T2 volume, whilst zones of different sizes are computed using a 26-voxel connectivity.
TEXTURE_GLSZM_ET_T2_ZSN	Textural (Gray-Level Size Zone Matrix)	The "Zone-Size Nonuniformity" within the ET in the T2 volume, whilst zones of different sizes are computed using a 26-voxel connectivity.
TEXTURE_GLSZM_ET_T2_ZP	Textural (Gray-Level Size Zone Matrix)	The "Zone Percentage" within the ET in the T2 volume, whilst zones of different sizes are computed using a 26-voxel connectivity.
TEXTURE_GLSZM_ET_T2_LGZE	Textural (Gray-Level Size Zone Matrix)	The "Low Gray-Level Zone Emphasis" within the ET in the T2 volume, whilst zones of different sizes are computed using a 26-voxel connectivity.
TEXTURE_GLSZM_ET_T2_HGZE	Textural (Gray-Level Size Zone Matrix)	The "High Gray-Level Zone Emphasis" within the ET in the T2 volume, whilst zones of different sizes are computed using a 26-voxel connectivity.
TEXTURE_GLSZM_ET_T2_SZLGE	Textural (Gray-Level Size Zone Matrix)	The "Small Zone Low Gray-Level Emphasis" within the ET in the T2 volume, whilst zones of different sizes are computed using a 26-voxel connectivity.
TEXTURE_GLSZM_ET_T2_SZHGE	Textural (Gray-Level Size Zone Matrix)	The "Small Zone High Gray-Level Emphasis" within the ET in the T2 volume, whilst zones of different sizes are computed using a 26-voxel connectivity.
TEXTURE_GLSZM_ET_T2_LZLGE	Textural (Gray-Level Size Zone Matrix)	The "Large Zone Low Gray-Level Emphasis" within the ET in the T2 volume, whilst zones of different sizes are computed using a 26-voxel connectivity.
TEXTURE_GLSZM_ET_T2_LZHGE	Textural (Gray-Level Size Zone Matrix)	The "Large Zone High Gray-Level Emphasis" within the ET in the T2 volume, whilst zones of different sizes are computed using a 26-voxel connectivity.
TEXTURE_GLSZM_ET_T2_GLV	Textural (Gray-Level Size Zone Matrix)	The "Gray-Level Variance" within the ET in the T2 volume, whilst zones of different sizes are computed using a 26-voxel connectivity.
TEXTURE_GLSZM_ET_T2_ZSV	Textural (Gray-Level Size Zone Matrix)	The "Zone-Size Variance" within the ET in the T2 volume, whilst zones of different sizes are computed using a 26-voxel connectivity.
TEXTURE_GLSZM_ET_FLAIR_SZE	Textural (Gray-Level Size Zone Matrix)	The "Small Zone Emphasis" within the ET in the FLAIR volume, whilst zones of different sizes are computed using a 26-voxel connectivity.
TEXTURE_GLSZM_ET_FLAIR_LZE	Textural (Gray-Level Size Zone Matrix)	The "Large Zone Emphasis" within the ET in the FLAIR volume, whilst zones of different sizes are computed using a 26-voxel connectivity.
TEXTURE_GLSZM_ET_FLAIR_GLN	Textural (Gray-Level Size Zone Matrix)	The "Gray-Level Nonuniformity" within the ET in the FLAIR volume, whilst zones of different sizes are computed using a 26-voxel connectivity.
TEXTURE_GLSZM_ET_FLAIR_ZSN	Textural (Gray-Level Size Zone Matrix)	The "Zone-Size Nonuniformity" within the ET in the FLAIR volume, whilst zones of different sizes are computed using a 26-voxel connectivity.
TEXTURE_GLSZM_ET_FLAIR_ZP	Textural (Gray-Level Size Zone Matrix)	The "Zone Percentage" within the ET in the FLAIR volume, whilst zones of different sizes are computed using a 26-voxel connectivity.
TEXTURE_GLSZM_ET_FLAIR_LGZE	Textural (Gray-Level Size Zone Matrix)	The "Low Gray-Level Zone Emphasis" within the ET in the FLAIR volume, whilst zones of different sizes are computed using a 26-voxel connectivity.
TEXTURE_GLSZM_ET_FLAIR_HGZE	Textural (Gray-Level Size Zone Matrix)	The "High Gray-Level Zone Emphasis" within the ET in the FLAIR volume, whilst zones of different sizes are computed using a 26-voxel connectivity.
TEXTURE_GLSZM_ET_FLAIR_SZLGE	Textural (Gray-Level Size Zone Matrix)	The "Small Zone Low Gray-Level Emphasis" within the ET in the FLAIR volume, whilst zones of different sizes are computed using a 26-voxel connectivity.
TEXTURE_GLSZM_ET_FLAIR_SZHGE	Textural (Gray-Level Size Zone Matrix)	The "Small Zone High Gray-Level Emphasis" within the ET in the FLAIR volume, whilst zones of different sizes are computed using a 26-voxel connectivity.
TEXTURE_GLSZM_ET_FLAIR_LZLGE	Textural (Gray-Level Size Zone Matrix)	The "Large Zone Low Gray-Level Emphasis" within the ET in the FLAIR volume, whilst zones of different sizes are computed using a 26-voxel connectivity.
TEXTURE_GLSZM_ET_FLAIR_LZHGE	Textural (Gray-Level Size Zone Matrix)	The "Large Zone High Gray-Level Emphasis" within the ET in the FLAIR volume, whilst zones of different sizes are computed using a 26-voxel connectivity.
TEXTURE_GLSZM_ET_FLAIR_GLV	Textural (Gray-Level Size Zone Matrix)	The "Gray-Level Variance" within the ET in the FLAIR volume, whilst zones of different sizes are computed using a 26-voxel connectivity.
TEXTURE_GLSZM_ET_FLAIR_ZSV	Textural (Gray-Level Size Zone Matrix)	The "Zone-Size Variance" within the ET in the FLAIR volume, whilst zones of different sizes are computed using a 26-voxel connectivity.
TEXTURE_GLSZM_ED_T1Gd_SZE	Textural (Gray-Level Size Zone Matrix)	The "Small Zone Emphasis" within the ED in the T1-Gd volume, whilst zones of different sizes are computed using a 26-voxel connectivity.
TEXTURE_GLSZM_ED_T1Gd_LZE	Textural (Gray-Level Size Zone Matrix)	The "Large Zone Emphasis" within the ED in the T1-Gd volume, whilst zones of different sizes are computed using a 26-voxel connectivity.
TEXTURE_GLSZM_ED_T1Gd_GLN	Textural (Gray-Level Size Zone Matrix)	The "Gray-Level Nonuniformity" within the ED in the T1-Gd volume, whilst zones of different sizes are computed using a 26-voxel connectivity.
TEXTURE_GLSZM_ED_T1Gd_ZSN	Textural (Gray-Level Size Zone Matrix)	The "Zone-Size Nonuniformity" within the ED in the T1-Gd volume, whilst zones of different sizes are computed using a 26-voxel connectivity.
TEXTURE_GLSZM_ED_T1Gd_ZP	Textural (Gray-Level Size Zone Matrix)	The "Zone Percentage" within the ED in the T1-Gd volume, whilst zones of different sizes are computed using a 26-voxel connectivity.
TEXTURE_GLSZM_ED_T1Gd_LGZE	Textural (Gray-Level Size Zone Matrix)	The "Low Gray-Level Zone Emphasis" within the ED in the T1-Gd volume, whilst zones of different sizes are computed using a 26-voxel connectivity.
TEXTURE_GLSZM_ED_T1Gd_HGZE	Textural (Gray-Level Size Zone Matrix)	The "High Gray-Level Zone Emphasis" within the ED in the T1-Gd volume, whilst zones of different sizes are computed using a 26-voxel connectivity.
TEXTURE_GLSZM_ED_T1Gd_SZLGE	Textural (Gray-Level Size Zone Matrix)	The "Small Zone Low Gray-Level Emphasis" within the ED in the T1-Gd volume, whilst zones of different sizes are computed using a 26-voxel connectivity.
TEXTURE_GLSZM_ED_T1Gd_SZHGE	Textural (Gray-Level Size Zone Matrix)	The "Small Zone High Gray-Level Emphasis" within the ED in the T1-Gd volume, whilst zones of different sizes are computed using a 26-voxel connectivity.
TEXTURE_GLSZM_ED_T1Gd_LZLGE	Textural (Gray-Level Size Zone Matrix)	The "Large Zone Low Gray-Level Emphasis" within the ED in the T1-Gd volume, whilst zones of different sizes are computed using a 26-voxel connectivity.
TEXTURE_GLSZM_ED_T1Gd_LZHGE	Textural (Gray-Level Size Zone Matrix)	The "Large Zone High Gray-Level Emphasis" within the ED in the T1-Gd volume, whilst zones of different sizes are computed using a 26-voxel connectivity.
TEXTURE_GLSZM_ED_T1Gd_GLV	Textural (Gray-Level Size Zone Matrix)	The "Gray-Level Variance" within the ED in the T1-Gd volume, whilst zones of different sizes are computed using a 26-voxel connectivity.
TEXTURE_GLSZM_ED_T1Gd_ZSV	Textural (Gray-Level Size Zone Matrix)	The "Zone-Size Variance" within the ED in the T1-Gd volume, whilst zones of different sizes are computed using a 26-voxel connectivity.
TEXTURE_GLSZM_ED_T1_SZE	Textural (Gray-Level Size Zone Matrix)	The "Small Zone Emphasis" within the ED in the T1 volume, whilst zones of different sizes are computed using a 26-voxel connectivity.
TEXTURE_GLSZM_ED_T1_LZE	Textural (Gray-Level Size Zone Matrix)	The "Large Zone Emphasis" within the ED in the T1 volume, whilst zones of different sizes are computed using a 26-voxel connectivity.
TEXTURE_GLSZM_ED_T1_GLN	Textural (Gray-Level Size Zone Matrix)	The "Gray-Level Nonuniformity" within the ED in the T1 volume, whilst zones of different sizes are computed using a 26-voxel connectivity.
TEXTURE_GLSZM_ED_T1_ZSN	Textural (Gray-Level Size Zone Matrix)	The "Zone-Size Nonuniformity" within the ED in the T1 volume, whilst zones of different sizes are computed using a 26-voxel connectivity.
TEXTURE_GLSZM_ED_T1_ZP	Textural (Gray-Level Size Zone Matrix)	The "Zone Percentage" within the ED in the T1 volume, whilst zones of different sizes are computed using a 26-voxel connectivity.
TEXTURE_GLSZM_ED_T1_LGZE	Textural (Gray-Level Size Zone Matrix)	The "Low Gray-Level Zone Emphasis" within the ED in the T1 volume, whilst zones of different sizes are computed using a 26-voxel connectivity.
TEXTURE_GLSZM_ED_T1_HGZE	Textural (Gray-Level Size Zone Matrix)	The "High Gray-Level Zone Emphasis" within the ED in the T1 volume, whilst zones of different sizes are computed using a 26-voxel connectivity.
TEXTURE_GLSZM_ED_T1_SZLGE	Textural (Gray-Level Size Zone Matrix)	The "Small Zone Low Gray-Level Emphasis" within the ED in the T1 volume, whilst zones of different sizes are computed using a 26-voxel connectivity.
TEXTURE_GLSZM_ED_T1_SZHGE	Textural (Gray-Level Size Zone Matrix)	The "Small Zone High Gray-Level Emphasis" within the ED in the T1 volume, whilst zones of different sizes are computed using a 26-voxel connectivity.
TEXTURE_GLSZM_ED_T1_LZLGE	Textural (Gray-Level Size Zone Matrix)	The "Large Zone Low Gray-Level Emphasis" within the ED in the T1 volume, whilst zones of different sizes are computed using a 26-voxel connectivity.
TEXTURE_GLSZM_ED_T1_LZHGE	Textural (Gray-Level Size Zone Matrix)	The "Large Zone High Gray-Level Emphasis" within the ED in the T1 volume, whilst zones of different sizes are computed using a 26-voxel connectivity.
TEXTURE_GLSZM_ED_T1_GLV	Textural (Gray-Level Size Zone Matrix)	The "Gray-Level Variance" within the ED in the T1 volume, whilst zones of different sizes are computed using a 26-voxel connectivity.
TEXTURE_GLSZM_ED_T1_ZSV	Textural (Gray-Level Size Zone Matrix)	The "Zone-Size Variance" within the ED in the T1 volume, whilst zones of different sizes are computed using a 26-voxel connectivity.
TEXTURE_GLSZM_ED_T2_SZE	Textural (Gray-Level Size Zone Matrix)	The "Small Zone Emphasis" within the ED in the T2 volume, whilst zones of different sizes are computed using a 26-voxel connectivity.
TEXTURE_GLSZM_ED_T2_LZE	Textural (Gray-Level Size Zone Matrix)	The "Large Zone Emphasis" within the ED in the T2 volume, whilst zones of different sizes are computed using a 26-voxel connectivity.
TEXTURE_GLSZM_ED_T2_GLN	Textural (Gray-Level Size Zone Matrix)	The "Gray-Level Nonuniformity" within the ED in the T2 volume, whilst zones of different sizes are computed using a 26-voxel connectivity.
TEXTURE_GLSZM_ED_T2_ZSN	Textural (Gray-Level Size Zone Matrix)	The "Zone-Size Nonuniformity" within the ED in the T2 volume, whilst zones of different sizes are computed using a 26-voxel connectivity.
TEXTURE_GLSZM_ED_T2_ZP	Textural (Gray-Level Size Zone Matrix)	The "Zone Percentage" within the ED in the T2 volume, whilst zones of different sizes are computed using a 26-voxel connectivity.
TEXTURE_GLSZM_ED_T2_LGZE	Textural (Gray-Level Size Zone Matrix)	The "Low Gray-Level Zone Emphasis" within the ED in the T2 volume, whilst zones of different sizes are computed using a 26-voxel connectivity.
TEXTURE_GLSZM_ED_T2_HGZE	Textural (Gray-Level Size Zone Matrix)	The "High Gray-Level Zone Emphasis" within the ED in the T2 volume, whilst zones of different sizes are computed using a 26-voxel connectivity.
TEXTURE_GLSZM_ED_T2_SZLGE	Textural (Gray-Level Size Zone Matrix)	The "Small Zone Low Gray-Level Emphasis" within the ED in the T2 volume, whilst zones of different sizes are computed using a 26-voxel connectivity.
TEXTURE_GLSZM_ED_T2_SZHGE	Textural (Gray-Level Size Zone Matrix)	The "Small Zone High Gray-Level Emphasis" within the ED in the T2 volume, whilst zones of different sizes are computed using a 26-voxel connectivity.
TEXTURE_GLSZM_ED_T2_LZLGE	Textural (Gray-Level Size Zone Matrix)	The "Large Zone Low Gray-Level Emphasis" within the ED in the T2 volume, whilst zones of different sizes are computed using a 26-voxel connectivity.
TEXTURE_GLSZM_ED_T2_LZHGE	Textural (Gray-Level Size Zone Matrix)	The "Large Zone High Gray-Level Emphasis" within the ED in the T2 volume, whilst zones of different sizes are computed using a 26-voxel connectivity.
TEXTURE_GLSZM_ED_T2_GLV	Textural (Gray-Level Size Zone Matrix)	The "Gray-Level Variance" within the ED in the T2 volume, whilst zones of different sizes are computed using a 26-voxel connectivity.
TEXTURE_GLSZM_ED_T2_ZSV	Textural (Gray-Level Size Zone Matrix)	The "Zone-Size Variance" within the ED in the T2 volume, whilst zones of different sizes are computed using a 26-voxel connectivity.
TEXTURE_GLSZM_ED_FLAIR_SZE	Textural (Gray-Level Size Zone Matrix)	The "Small Zone Emphasis" within the ED in the FLAIR volume, whilst zones of different sizes are computed using a 26-voxel connectivity.
TEXTURE_GLSZM_ED_FLAIR_LZE	Textural (Gray-Level Size Zone Matrix)	The "Large Zone Emphasis" within the ED in the FLAIR volume, whilst zones of different sizes are computed using a 26-voxel connectivity.
TEXTURE_GLSZM_ED_FLAIR_GLN	Textural (Gray-Level Size Zone Matrix)	The "Gray-Level Nonuniformity" within the ED in the FLAIR volume, whilst zones of different sizes are computed using a 26-voxel connectivity.
TEXTURE_GLSZM_ED_FLAIR_ZSN	Textural (Gray-Level Size Zone Matrix)	The "Zone-Size Nonuniformity" within the ED in the FLAIR volume, whilst zones of different sizes are computed using a 26-voxel connectivity.
TEXTURE_GLSZM_ED_FLAIR_ZP	Textural (Gray-Level Size Zone Matrix)	The "Zone Percentage" within the ED in the FLAIR volume, whilst zones of different sizes are computed using a 26-voxel connectivity.
TEXTURE_GLSZM_ED_FLAIR_LGZE	Textural (Gray-Level Size Zone Matrix)	The "Low Gray-Level Zone Emphasis" within the ED in the FLAIR volume, whilst zones of different sizes are computed using a 26-voxel connectivity.
TEXTURE_GLSZM_ED_FLAIR_HGZE	Textural (Gray-Level Size Zone Matrix)	The "High Gray-Level Zone Emphasis" within the ED in the FLAIR volume, whilst zones of different sizes are computed using a 26-voxel connectivity.
TEXTURE_GLSZM_ED_FLAIR_SZLGE	Textural (Gray-Level Size Zone Matrix)	The "Small Zone Low Gray-Level Emphasis" within the ED in the FLAIR volume, whilst zones of different sizes are computed using a 26-voxel connectivity.
TEXTURE_GLSZM_ED_FLAIR_SZHGE	Textural (Gray-Level Size Zone Matrix)	The "Small Zone High Gray-Level Emphasis" within the ED in the FLAIR volume, whilst zones of different sizes are computed using a 26-voxel connectivity.
TEXTURE_GLSZM_ED_FLAIR_LZLGE	Textural (Gray-Level Size Zone Matrix)	The "Large Zone Low Gray-Level Emphasis" within the ED in the FLAIR volume, whilst zones of different sizes are computed using a 26-voxel connectivity.
TEXTURE_GLSZM_ED_FLAIR_LZHGE	Textural (Gray-Level Size Zone Matrix)	The "Large Zone High Gray-Level Emphasis" within the ED in the FLAIR volume, whilst zones of different sizes are computed using a 26-voxel connectivity.
TEXTURE_GLSZM_ED_FLAIR_GLV	Textural (Gray-Level Size Zone Matrix)	The "Gray-Level Variance" within the ED in the FLAIR volume, whilst zones of different sizes are computed using a 26-voxel connectivity.
TEXTURE_GLSZM_ED_FLAIR_ZSV	Textural (Gray-Level Size Zone Matrix)	The "Zone-Size Variance" within the ED in the FLAIR volume, whilst zones of different sizes are computed using a 26-voxel connectivity.
TEXTURE_GLSZM_NET_T1Gd_SZE	Textural (Gray-Level Size Zone Matrix)	The "Small Zone Emphasis" within the NET in the T1-Gd volume, whilst zones of different sizes are computed using a 26-voxel connectivity.
TEXTURE_GLSZM_NET_T1Gd_LZE	Textural (Gray-Level Size Zone Matrix)	The "Large Zone Emphasis" within the NET in the T1-Gd volume, whilst zones of different sizes are computed using a 26-voxel connectivity.
TEXTURE_GLSZM_NET_T1Gd_GLN	Textural (Gray-Level Size Zone Matrix)	The "Gray-Level Nonuniformity" within the NET in the T1-Gd volume, whilst zones of different sizes are computed using a 26-voxel connectivity.
TEXTURE_GLSZM_NET_T1Gd_ZSN	Textural (Gray-Level Size Zone Matrix)	The "Zone-Size Nonuniformity" within the NET in the T1-Gd volume, whilst zones of different sizes are computed using a 26-voxel connectivity.
TEXTURE_GLSZM_NET_T1Gd_ZP	Textural (Gray-Level Size Zone Matrix)	The "Zone Percentage" within the NET in the T1-Gd volume, whilst zones of different sizes are computed using a 26-voxel connectivity.
TEXTURE_GLSZM_NET_T1Gd_LGZE	Textural (Gray-Level Size Zone Matrix)	The "Low Gray-Level Zone Emphasis" within the NET in the T1-Gd volume, whilst zones of different sizes are computed using a 26-voxel connectivity.
TEXTURE_GLSZM_NET_T1Gd_HGZE	Textural (Gray-Level Size Zone Matrix)	The "High Gray-Level Zone Emphasis" within the NET in the T1-Gd volume, whilst zones of different sizes are computed using a 26-voxel connectivity.
TEXTURE_GLSZM_NET_T1Gd_SZLGE	Textural (Gray-Level Size Zone Matrix)	The "Small Zone Low Gray-Level Emphasis" within the NET in the T1-Gd volume, whilst zones of different sizes are computed using a 26-voxel connectivity.
TEXTURE_GLSZM_NET_T1Gd_SZHGE	Textural (Gray-Level Size Zone Matrix)	The "Small Zone High Gray-Level Emphasis" within the NET in the T1-Gd volume, whilst zones of different sizes are computed using a 26-voxel connectivity.
TEXTURE_GLSZM_NET_T1Gd_LZLGE	Textural (Gray-Level Size Zone Matrix)	The "Large Zone Low Gray-Level Emphasis" within the NET in the T1-Gd volume, whilst zones of different sizes are computed using a 26-voxel connectivity.
TEXTURE_GLSZM_NET_T1Gd_LZHGE	Textural (Gray-Level Size Zone Matrix)	The "Large Zone High Gray-Level Emphasis" within the NET in the T1-Gd volume, whilst zones of different sizes are computed using a 26-voxel connectivity.
TEXTURE_GLSZM_NET_T1Gd_GLV	Textural (Gray-Level Size Zone Matrix)	The "Gray-Level Variance" within the NET in the T1-Gd volume, whilst zones of different sizes are computed using a 26-voxel connectivity.
TEXTURE_GLSZM_NET_T1Gd_ZSV	Textural (Gray-Level Size Zone Matrix)	The "Zone-Size Variance" within the NET in the T1-Gd volume, whilst zones of different sizes are computed using a 26-voxel connectivity.
TEXTURE_GLSZM_NET_T1_SZE	Textural (Gray-Level Size Zone Matrix)	The "Small Zone Emphasis" within the NET in the T1 volume, whilst zones of different sizes are computed using a 26-voxel connectivity.
TEXTURE_GLSZM_NET_T1_LZE	Textural (Gray-Level Size Zone Matrix)	The "Large Zone Emphasis" within the NET in the T1 volume, whilst zones of different sizes are computed using a 26-voxel connectivity.
TEXTURE_GLSZM_NET_T1_GLN	Textural (Gray-Level Size Zone Matrix)	The "Gray-Level Nonuniformity" within the NET in the T1 volume, whilst zones of different sizes are computed using a 26-voxel connectivity.
TEXTURE_GLSZM_NET_T1_ZSN	Textural (Gray-Level Size Zone Matrix)	The "Zone-Size Nonuniformity" within the NET in the T1 volume, whilst zones of different sizes are computed using a 26-voxel connectivity.
TEXTURE_GLSZM_NET_T1_ZP	Textural (Gray-Level Size Zone Matrix)	The "Zone Percentage" within the NET in the T1 volume, whilst zones of different sizes are computed using a 26-voxel connectivity.
TEXTURE_GLSZM_NET_T1_LGZE	Textural (Gray-Level Size Zone Matrix)	The "Low Gray-Level Zone Emphasis" within the NET in the T1 volume, whilst zones of different sizes are computed using a 26-voxel connectivity.
TEXTURE_GLSZM_NET_T1_HGZE	Textural (Gray-Level Size Zone Matrix)	The "High Gray-Level Zone Emphasis" within the NET in the T1 volume, whilst zones of different sizes are computed using a 26-voxel connectivity.
TEXTURE_GLSZM_NET_T1_SZLGE	Textural (Gray-Level Size Zone Matrix)	The "Small Zone Low Gray-Level Emphasis" within the NET in the T1 volume, whilst zones of different sizes are computed using a 26-voxel connectivity.
TEXTURE_GLSZM_NET_T1_SZHGE	Textural (Gray-Level Size Zone Matrix)	The "Small Zone High Gray-Level Emphasis" within the NET in the T1 volume, whilst zones of different sizes are computed using a 26-voxel connectivity.
TEXTURE_GLSZM_NET_T1_LZLGE	Textural (Gray-Level Size Zone Matrix)	The "Large Zone Low Gray-Level Emphasis" within the NET in the T1 volume, whilst zones of different sizes are computed using a 26-voxel connectivity.
TEXTURE_GLSZM_NET_T1_LZHGE	Textural (Gray-Level Size Zone Matrix)	The "Large Zone High Gray-Level Emphasis" within the NET in the T1 volume, whilst zones of different sizes are computed using a 26-voxel connectivity.
TEXTURE_GLSZM_NET_T1_GLV	Textural (Gray-Level Size Zone Matrix)	The "Gray-Level Variance" within the NET in the T1 volume, whilst zones of different sizes are computed using a 26-voxel connectivity.
TEXTURE_GLSZM_NET_T1_ZSV	Textural (Gray-Level Size Zone Matrix)	The "Zone-Size Variance" within the NET in the T1 volume, whilst zones of different sizes are computed using a 26-voxel connectivity.
TEXTURE_GLSZM_NET_T2_SZE	Textural (Gray-Level Size Zone Matrix)	The "Small Zone Emphasis" within the NET in the T2 volume, whilst zones of different sizes are computed using a 26-voxel connectivity.
TEXTURE_GLSZM_NET_T2_LZE	Textural (Gray-Level Size Zone Matrix)	The "Large Zone Emphasis" within the NET in the T2 volume, whilst zones of different sizes are computed using a 26-voxel connectivity.
TEXTURE_GLSZM_NET_T2_GLN	Textural (Gray-Level Size Zone Matrix)	The "Gray-Level Nonuniformity" within the NET in the T2 volume, whilst zones of different sizes are computed using a 26-voxel connectivity.
TEXTURE_GLSZM_NET_T2_ZSN	Textural (Gray-Level Size Zone Matrix)	The "Zone-Size Nonuniformity" within the NET in the T2 volume, whilst zones of different sizes are computed using a 26-voxel connectivity.
TEXTURE_GLSZM_NET_T2_ZP	Textural (Gray-Level Size Zone Matrix)	The "Zone Percentage" within the NET in the T2 volume, whilst zones of different sizes are computed using a 26-voxel connectivity.
TEXTURE_GLSZM_NET_T2_LGZE	Textural (Gray-Level Size Zone Matrix)	The "Low Gray-Level Zone Emphasis" within the NET in the T2 volume, whilst zones of different sizes are computed using a 26-voxel connectivity.
TEXTURE_GLSZM_NET_T2_HGZE	Textural (Gray-Level Size Zone Matrix)	The "High Gray-Level Zone Emphasis" within the NET in the T2 volume, whilst zones of different sizes are computed using a 26-voxel connectivity.
TEXTURE_GLSZM_NET_T2_SZLGE	Textural (Gray-Level Size Zone Matrix)	The "Small Zone Low Gray-Level Emphasis" within the NET in the T2 volume, whilst zones of different sizes are computed using a 26-voxel connectivity.
TEXTURE_GLSZM_NET_T2_SZHGE	Textural (Gray-Level Size Zone Matrix)	The "Small Zone High Gray-Level Emphasis" within the NET in the T2 volume, whilst zones of different sizes are computed using a 26-voxel connectivity.
TEXTURE_GLSZM_NET_T2_LZLGE	Textural (Gray-Level Size Zone Matrix)	The "Large Zone Low Gray-Level Emphasis" within the NET in the T2 volume, whilst zones of different sizes are computed using a 26-voxel connectivity.
TEXTURE_GLSZM_NET_T2_LZHGE	Textural (Gray-Level Size Zone Matrix)	The "Large Zone High Gray-Level Emphasis" within the NET in the T2 volume, whilst zones of different sizes are computed using a 26-voxel connectivity.
TEXTURE_GLSZM_NET_T2_GLV	Textural (Gray-Level Size Zone Matrix)	The "Gray-Level Variance" within the NET in the T2 volume, whilst zones of different sizes are computed using a 26-voxel connectivity.
TEXTURE_GLSZM_NET_T2_ZSV	Textural (Gray-Level Size Zone Matrix)	The "Zone-Size Variance" within the NET in the T2 volume, whilst zones of different sizes are computed using a 26-voxel connectivity.
TEXTURE_GLSZM_NET_FLAIR_SZE	Textural (Gray-Level Size Zone Matrix)	The "Small Zone Emphasis" within the NET in the FLAIR volume, whilst zones of different sizes are computed using a 26-voxel connectivity.
TEXTURE_GLSZM_NET_FLAIR_LZE	Textural (Gray-Level Size Zone Matrix)	The "Large Zone Emphasis" within the NET in the FLAIR volume, whilst zones of different sizes are computed using a 26-voxel connectivity.
TEXTURE_GLSZM_NET_FLAIR_GLN	Textural (Gray-Level Size Zone Matrix)	The "Gray-Level Nonuniformity" within the NET in the FLAIR volume, whilst zones of different sizes are computed using a 26-voxel connectivity.
TEXTURE_GLSZM_NET_FLAIR_ZSN	Textural (Gray-Level Size Zone Matrix)	The "Zone-Size Nonuniformity" within the NET in the FLAIR volume, whilst zones of different sizes are computed using a 26-voxel connectivity.
TEXTURE_GLSZM_NET_FLAIR_ZP	Textural (Gray-Level Size Zone Matrix)	The "Zone Percentage" within the NET in the FLAIR volume, whilst zones of different sizes are computed using a 26-voxel connectivity.
TEXTURE_GLSZM_NET_FLAIR_LGZE	Textural (Gray-Level Size Zone Matrix)	The "Low Gray-Level Zone Emphasis" within the NET in the FLAIR volume, whilst zones of different sizes are computed using a 26-voxel connectivity.
TEXTURE_GLSZM_NET_FLAIR_HGZE	Textural (Gray-Level Size Zone Matrix)	The "High Gray-Level Zone Emphasis" within the NET in the FLAIR volume, whilst zones of different sizes are computed using a 26-voxel connectivity.
TEXTURE_GLSZM_NET_FLAIR_SZLGE	Textural (Gray-Level Size Zone Matrix)	The "Small Zone Low Gray-Level Emphasis" within the NET in the FLAIR volume, whilst zones of different sizes are computed using a 26-voxel connectivity.
TEXTURE_GLSZM_NET_FLAIR_SZHGE	Textural (Gray-Level Size Zone Matrix)	The "Small Zone High Gray-Level Emphasis" within the NET in the FLAIR volume, whilst zones of different sizes are computed using a 26-voxel connectivity.
TEXTURE_GLSZM_NET_FLAIR_LZLGE	Textural (Gray-Level Size Zone Matrix)	The "Large Zone Low Gray-Level Emphasis" within the NET in the FLAIR volume, whilst zones of different sizes are computed using a 26-voxel connectivity.
TEXTURE_GLSZM_NET_FLAIR_LZHGE	Textural (Gray-Level Size Zone Matrix)	The "Large Zone High Gray-Level Emphasis" within the NET in the FLAIR volume, whilst zones of different sizes are computed using a 26-voxel connectivity.
TEXTURE_GLSZM_NET_FLAIR_GLV	Textural (Gray-Level Size Zone Matrix)	The "Gray-Level Variance" within the NET in the FLAIR volume, whilst zones of different sizes are computed using a 26-voxel connectivity.
TEXTURE_GLSZM_NET_FLAIR_ZSV	Textural (Gray-Level Size Zone Matrix)	The "Zone-Size Variance" within the NET in the FLAIR volume, whilst zones of different sizes are computed using a 26-voxel connectivity.
TEXTURE_NGTDM_ET_T1Gd_Coarseness	Textural (Neighborhood Gray-Tone Difference Matrix)	The Coarseness within the ET in the T1-Gd volume, considering a 26-voxel connectivity.
TEXTURE_NGTDM_ET_T1Gd_Contrast	Textural (Neighborhood Gray-Tone Difference Matrix)	The Contrast within the ET in the T1-Gd volume, considering a 26-voxel connectivity.
TEXTURE_NGTDM_ET_T1Gd_Busyness	Textural (Neighborhood Gray-Tone Difference Matrix)	The Busyness within the ET in the T1-Gd volume, considering a 26-voxel connectivity.
TEXTURE_NGTDM_ET_T1Gd_Complexity	Textural (Neighborhood Gray-Tone Difference Matrix)	The Complexity within the ET in the T1-Gd volume, considering a 26-voxel connectivity.
TEXTURE_NGTDM_ET_T1Gd_Strength	Textural (Neighborhood Gray-Tone Difference Matrix)	The Strength within the ET in the T1-Gd volume, considering a 26-voxel connectivity.
TEXTURE_NGTDM_ET_T1_Coarseness	Textural (Neighborhood Gray-Tone Difference Matrix)	The Coarseness within the ET in the T1 volume, considering a 26-voxel connectivity.
TEXTURE_NGTDM_ET_T1_Contrast	Textural (Neighborhood Gray-Tone Difference Matrix)	The Contrast within the ET in the T1 volume, considering a 26-voxel connectivity.
TEXTURE_NGTDM_ET_T1_Busyness	Textural (Neighborhood Gray-Tone Difference Matrix)	The Busyness within the ET in the T1 volume, considering a 26-voxel connectivity.
TEXTURE_NGTDM_ET_T1_Complexity	Textural (Neighborhood Gray-Tone Difference Matrix)	The Complexity within the ET in the T1 volume, considering a 26-voxel connectivity.
TEXTURE_NGTDM_ET_T1_Strength	Textural (Neighborhood Gray-Tone Difference Matrix)	The Strength within the ET in the T1 volume, considering a 26-voxel connectivity.
TEXTURE_NGTDM_ET_T2_Coarseness	Textural (Neighborhood Gray-Tone Difference Matrix)	The Coarseness within the ET in the T2 volume, considering a 26-voxel connectivity.
TEXTURE_NGTDM_ET_T2_Contrast	Textural (Neighborhood Gray-Tone Difference Matrix)	The Contrast within the ET in the T2 volume, considering a 26-voxel connectivity.
TEXTURE_NGTDM_ET_T2_Busyness	Textural (Neighborhood Gray-Tone Difference Matrix)	The Busyness within the ET in the T2 volume, considering a 26-voxel connectivity.
TEXTURE_NGTDM_ET_T2_Complexity	Textural (Neighborhood Gray-Tone Difference Matrix)	The Complexity within the ET in the T2 volume, considering a 26-voxel connectivity.
TEXTURE_NGTDM_ET_T2_Strength	Textural (Neighborhood Gray-Tone Difference Matrix)	The Strength within the ET in the T2 volume, considering a 26-voxel connectivity.
TEXTURE_NGTDM_ET_FLAIR_Coarseness	Textural (Neighborhood Gray-Tone Difference Matrix)	The Coarseness within the ET in the FLAIR volume, considering a 26-voxel connectivity.
TEXTURE_NGTDM_ET_FLAIR_Contrast	Textural (Neighborhood Gray-Tone Difference Matrix)	The Contrast within the ET in the FLAIR volume, considering a 26-voxel connectivity.
TEXTURE_NGTDM_ET_FLAIR_Busyness	Textural (Neighborhood Gray-Tone Difference Matrix)	The Busyness within the ET in the FLAIR volume, considering a 26-voxel connectivity.
TEXTURE_NGTDM_ET_FLAIR_Complexity	Textural (Neighborhood Gray-Tone Difference Matrix)	The Complexity within the ET in the FLAIR volume, considering a 26-voxel connectivity.
TEXTURE_NGTDM_ET_FLAIR_Strength	Textural (Neighborhood Gray-Tone Difference Matrix)	The Strength within the ET in the FLAIR volume, considering a 26-voxel connectivity.
TEXTURE_NGTDM_ED_T1Gd_Coarseness	Textural (Neighborhood Gray-Tone Difference Matrix)	The Coarseness within the ED in the T1-Gd volume, considering a 26-voxel connectivity.
TEXTURE_NGTDM_ED_T1Gd_Contrast	Textural (Neighborhood Gray-Tone Difference Matrix)	The Contrast within the ED in the T1-Gd volume, considering a 26-voxel connectivity.
TEXTURE_NGTDM_ED_T1Gd_Busyness	Textural (Neighborhood Gray-Tone Difference Matrix)	The Busyness within the ED in the T1-Gd volume, considering a 26-voxel connectivity.
TEXTURE_NGTDM_ED_T1Gd_Complexity	Textural (Neighborhood Gray-Tone Difference Matrix)	The Complexity within the ED in the T1-Gd volume, considering a 26-voxel connectivity.
TEXTURE_NGTDM_ED_T1Gd_Strength	Textural (Neighborhood Gray-Tone Difference Matrix)	The Strength within the ED in the T1-Gd volume, considering a 26-voxel connectivity.
TEXTURE_NGTDM_ED_T1_Coarseness	Textural (Neighborhood Gray-Tone Difference Matrix)	The Coarseness within the ED in the T1 volume, considering a 26-voxel connectivity.
TEXTURE_NGTDM_ED_T1_Contrast	Textural (Neighborhood Gray-Tone Difference Matrix)	The Contrast within the ED in the T1 volume, considering a 26-voxel connectivity.
TEXTURE_NGTDM_ED_T1_Busyness	Textural (Neighborhood Gray-Tone Difference Matrix)	The Busyness within the ED in the T1 volume, considering a 26-voxel connectivity.
TEXTURE_NGTDM_ED_T1_Complexity	Textural (Neighborhood Gray-Tone Difference Matrix)	The Complexity within the ED in the T1 volume, considering a 26-voxel connectivity.
TEXTURE_NGTDM_ED_T1_Strength	Textural (Neighborhood Gray-Tone Difference Matrix)	The Strength within the ED in the T1 volume, considering a 26-voxel connectivity.
TEXTURE_NGTDM_ED_T2_Coarseness	Textural (Neighborhood Gray-Tone Difference Matrix)	The Coarseness within the ED in the T2 volume, considering a 26-voxel connectivity.
TEXTURE_NGTDM_ED_T2_Contrast	Textural (Neighborhood Gray-Tone Difference Matrix)	The Contrast within the ED in the T2 volume, considering a 26-voxel connectivity.
TEXTURE_NGTDM_ED_T2_Busyness	Textural (Neighborhood Gray-Tone Difference Matrix)	The Busyness within the ED in the T2 volume, considering a 26-voxel connectivity.
TEXTURE_NGTDM_ED_T2_Complexity	Textural (Neighborhood Gray-Tone Difference Matrix)	The Complexity within the ED in the T2 volume, considering a 26-voxel connectivity.
TEXTURE_NGTDM_ED_T2_Strength	Textural (Neighborhood Gray-Tone Difference Matrix)	The Strength within the ED in the T2 volume, considering a 26-voxel connectivity.
TEXTURE_NGTDM_ED_FLAIR_Coarseness	Textural (Neighborhood Gray-Tone Difference Matrix)	The Coarseness within the ED in the FLAIR volume, considering a 26-voxel connectivity.
TEXTURE_NGTDM_ED_FLAIR_Contrast	Textural (Neighborhood Gray-Tone Difference Matrix)	The Contrast within the ED in the FLAIR volume, considering a 26-voxel connectivity.
TEXTURE_NGTDM_ED_FLAIR_Busyness	Textural (Neighborhood Gray-Tone Difference Matrix)	The Busyness within the ED in the FLAIR volume, considering a 26-voxel connectivity.
TEXTURE_NGTDM_ED_FLAIR_Complexity	Textural (Neighborhood Gray-Tone Difference Matrix)	The Complexity within the ED in the FLAIR volume, considering a 26-voxel connectivity.
TEXTURE_NGTDM_ED_FLAIR_Strength	Textural (Neighborhood Gray-Tone Difference Matrix)	The Strength within the ED in the FLAIR volume, considering a 26-voxel connectivity.
TEXTURE_NGTDM_NET_T1Gd_Coarseness	Textural (Neighborhood Gray-Tone Difference Matrix)	The Coarseness within the NET in the T1-Gd volume, considering a 26-voxel connectivity.
TEXTURE_NGTDM_NET_T1Gd_Contrast	Textural (Neighborhood Gray-Tone Difference Matrix)	The Contrast within the NET in the T1-Gd volume, considering a 26-voxel connectivity.
TEXTURE_NGTDM_NET_T1Gd_Busyness	Textural (Neighborhood Gray-Tone Difference Matrix)	The Busyness within the NET in the T1-Gd volume, considering a 26-voxel connectivity.
TEXTURE_NGTDM_NET_T1Gd_Complexity	Textural (Neighborhood Gray-Tone Difference Matrix)	The Complexity within the NET in the T1-Gd volume, considering a 26-voxel connectivity.
TEXTURE_NGTDM_NET_T1Gd_Strength	Textural (Neighborhood Gray-Tone Difference Matrix)	The Strength within the NET in the T1-Gd volume, considering a 26-voxel connectivity.
TEXTURE_NGTDM_NET_T1_Coarseness	Textural (Neighborhood Gray-Tone Difference Matrix)	The Coarseness within the NET in the T1 volume, considering a 26-voxel connectivity.
TEXTURE_NGTDM_NET_T1_Contrast	Textural (Neighborhood Gray-Tone Difference Matrix)	The Contrast within the NET in the T1 volume, considering a 26-voxel connectivity.
TEXTURE_NGTDM_NET_T1_Busyness	Textural (Neighborhood Gray-Tone Difference Matrix)	The Busyness within the NET in the T1 volume, considering a 26-voxel connectivity.
TEXTURE_NGTDM_NET_T1_Complexity	Textural (Neighborhood Gray-Tone Difference Matrix)	The Complexity within the NET in the T1 volume, considering a 26-voxel connectivity.
TEXTURE_NGTDM_NET_T1_Strength	Textural (Neighborhood Gray-Tone Difference Matrix)	The Strength within the NET in the T1 volume, considering a 26-voxel connectivity.
TEXTURE_NGTDM_NET_T2_Coarseness	Textural (Neighborhood Gray-Tone Difference Matrix)	The Coarseness within the NET in the T2 volume, considering a 26-voxel connectivity.
TEXTURE_NGTDM_NET_T2_Contrast	Textural (Neighborhood Gray-Tone Difference Matrix)	The Contrast within the NET in the T2 volume, considering a 26-voxel connectivity.
TEXTURE_NGTDM_NET_T2_Busyness	Textural (Neighborhood Gray-Tone Difference Matrix)	The Busyness within the NET in the T2 volume, considering a 26-voxel connectivity.
TEXTURE_NGTDM_NET_T2_Complexity	Textural (Neighborhood Gray-Tone Difference Matrix)	The Complexity within the NET in the T2 volume, considering a 26-voxel connectivity.
TEXTURE_NGTDM_NET_T2_Strength	Textural (Neighborhood Gray-Tone Difference Matrix)	The Strength within the NET in the T2 volume, considering a 26-voxel connectivity.
TEXTURE_NGTDM_NET_FLAIR_Coarseness	Textural (Neighborhood Gray-Tone Difference Matrix)	The Coarseness within the NET in the FLAIR volume, considering a 26-voxel connectivity.
TEXTURE_NGTDM_NET_FLAIR_Contrast	Textural (Neighborhood Gray-Tone Difference Matrix)	The Contrast within the NET in the FLAIR volume, considering a 26-voxel connectivity.
TEXTURE_NGTDM_NET_FLAIR_Busyness	Textural (Neighborhood Gray-Tone Difference Matrix)	The Busyness within the NET in the FLAIR volume, considering a 26-voxel connectivity.
TEXTURE_NGTDM_NET_FLAIR_Complexity	Textural (Neighborhood Gray-Tone Difference Matrix)	The Complexity within the NET in the FLAIR volume, considering a 26-voxel connectivity.
TEXTURE_NGTDM_NET_FLAIR_Strength	Textural (Neighborhood Gray-Tone Difference Matrix)	The Strength within the NET in the FLAIR volume, considering a 26-voxel connectivity.
TGM_p1	Tumor Growth Model Parameter	The mass-effect parameters considering all apparent tumors.
TGM_dw	Tumor Growth Model Parameter	The estimated diffusion coefficient of white matter, considering all apparent tumors.
TGM_Cog_X_1	Tumor Growth Model Parameter	The 3D coordinates (in Sagital plane) of the apparent tumor's center of gravity.
TGM_Cog_Y_1	Tumor Growth Model Parameter	The 3D coordinates (in Coronal plane) of the apparent tumor's center of gravity.
TGM_Cog_Z_1	Tumor Growth Model Parameter	The 3D coordinates (in Axial plane) of the apparent tumor's center of gravity.
TGM_T_1	Tumor Growth Model Parameter	The estimated growth time of the apparent tumor in this location.
TGM_Cog_X_2	Tumor Growth Model Parameter	The 3D coordinates (in Sagital plane) of the apparent tumor's center of gravity.
TGM_Cog_Y_2	Tumor Growth Model Parameter	The 3D coordinates (in Coronal plane) of the apparent tumor's center of gravity.
TGM_Cog_Z_2	Tumor Growth Model Parameter	The 3D coordinates (in Axial plane) of the apparent tumor's center of gravity.
TGM_T_2	Tumor Growth Model Parameter	The estimated growth time of the apparent tumor in this location.
TGM_Cog_X_3	Tumor Growth Model Parameter	The 3D coordinates (in Sagital plane) of the apparent tumor's center of gravity.
TGM_Cog_Y_3	Tumor Growth Model Parameter	The 3D coordinates (in Coronal plane) of the apparent tumor's center of gravity.
TGM_Cog_Z_3	Tumor Growth Model Parameter	The 3D coordinates (in Axial plane) of the apparent tumor's center of gravity.
TGM_T_3	Tumor Growth Model Parameter	The estimated growth time of the apparent tumor in this location.
TGM_Cog_X_4	Tumor Growth Model Parameter	The 3D coordinates (in Sagital plane) of the apparent tumor's center of gravity.
TGM_Cog_Y_4	Tumor Growth Model Parameter	The 3D coordinates (in Coronal plane) of the apparent tumor's center of gravity.
TGM_Cog_Z_4	Tumor Growth Model Parameter	The 3D coordinates (in Axial plane) of the apparent tumor's center of gravity.
TGM_T_4	Tumor Growth Model Parameter	The estimated growth time of the apparent tumor in this location.
TGM_Cog_X_5	Tumor Growth Model Parameter	The 3D coordinates (in Sagital plane) of the apparent tumor's center of gravity.
TGM_Cog_Y_5	Tumor Growth Model Parameter	The 3D coordinates (in Coronal plane) of the apparent tumor's center of gravity.
TGM_Cog_Z_5	Tumor Growth Model Parameter	The 3D coordinates (in Axial plane) of the apparent tumor's center of gravity.
TGM_T_5	Tumor Growth Model Parameter	The estimated growth time of the apparent tumor in this location.
TGM_Cog_X_6	Tumor Growth Model Parameter	The 3D coordinates (in Sagital plane) of the apparent tumor's center of gravity.
TGM_Cog_Y_6	Tumor Growth Model Parameter	The 3D coordinates (in Coronal plane) of the apparent tumor's center of gravity.
TGM_Cog_Z_6	Tumor Growth Model Parameter	The 3D coordinates (in Axial plane) of the apparent tumor's center of gravity.
TGM_T_6	Tumor Growth Model Parameter	The estimated growth time of the apparent tumor in this location.
